# Developmental contexts of gene-regulatory evolution following whole genome duplication in salmonids

**DOI:** 10.1038/s41559-026-03171-6

**Published:** 2026-09-29

**Authors:** Marie-Odile Baudement, Diego Perojil Morata, Gareth B. Gillard, Pooran S. Dewari, Manu Kumar Gundappa, Tomasz Podgorniak, Lars Grønvold, Damir Baranasic, Audrey Laurent, François Giudicelli, Bojan Zunar, Erika Carrera-García, Aline Perquis, Aurélien Brionne, Tan Thi Nguyen, Rose Ruiz Daniels, Gabriela A. Merino, David Thybert, Garth R. Ilsley, Alexandra Louis, Torgeir R. Hvidsten, Camille Berthelot, Peter W. Harrison, Hugues Roest Crollius, Yann Guiguen, Boris Lenhard, Simen R. Sandve, Julien Bobe, Matthew P. Kent, Sigbjørn Lien, Daniel J. Macqueen

**Affiliations:** 1 Centre for Integrative Genetics, Faculty of BioSciences, Norwegian University of Life Sciences, Ås, Norway; 2 The Roslin Institute, University of Edinburgh; Easter Bush Campus, Edinburgh, UK; 3 MRC London Institute of Medical Sciences, London, UK; 4 Institute of Clinical Sciences, Imperial College London, London, UK; 5 Animal Breeding and Genomics, Wageningen University and Research, Wageningen, the Netherlands; 6 Ruđer Bošković Institute, Zagreb, Croatia; 7 Fish Physiology and Genomics, INRAE, Rennes, France; 8 Institut de Biologie de l’ENS, Département de Biologie, Ecole normale supérieure, CNRS, Inserm, Université PSL, Paris, France; 9 Faculty of Food Technology and Biotechnology, University of Zagreb, Zagreb, Croatia; 10 Institute of Aquaculture, University of Stirling, Stirling, UK; 11 European Molecular Biology Laboratory, European Bioinformatics Institute, Cambridge, UK; 12 Faculty of Chemistry, Biotechnology and Food Science, Norwegian University of Life Sciences, Ås, Norway; 13 Institut Pasteur, Université Paris Cité, CNRS UMR 3525, INSERM UA12, Paris, France

**Keywords:** Molecular evolution, Functional genomics

## Abstract

Whole genome duplication (WGD) events during early vertebrate evolution may have promoted phenotypic innovation by expanding the genetic material for functional diversification. However, their ancient origins obscure how gene regulation evolved during rediploidization, the process that generates duplicate genes and regulatory elements following WGD. Salmonids provide a unique window to these mechanisms, because they experienced a more recent autotetraploid WGD and remain at an earlier stage of rediploidization. Here we generated 771 multiomics datasets spanning embryonic and adult tissues in Atlantic salmon and rainbow trout to investigate the evolution of gene expression and regulatory elements. Our analyses show that regulatory evolution following the salmonid WGD was shaped by developmental and tissue-specific constraints known to act across vertebrate species. We identify a peak of within-genome regulatory constraint at the vertebrate phylotypic period, associated with the functional retention of duplicated enhancer pairs harbouring deeply conserved non-coding elements enriched in motifs for master developmental transcription factors. We further identify roles for enhancers and transposable elements in the expression divergence of duplicated genes. This study advances the understanding of regulatory evolution following WGD and offers an open resource supporting vertebrate comparative genomics and research applications in salmonid aquaculture and conservation.

## Abstract (web summary)

A comparative analysis of multiomics data from embryonic and adult tissues of Atlantic salmon and rainbow trout reveals developmental and tissue-specific constraints shaping regulatory evolution following whole genome duplication.

## Main

Whole genome duplication (WGD) occurred at many stages of eukaryotic evolution^1–3^, including sequential rounds in the vertebrate (1R), jawed vertebrate (2R)^4–8^ and teleost fish (3R) ancestors^9,10^. Although duplicate genes created by WGD (ohnologues) are often lost, those retained are enriched for roles in development, signalling and transcriptional regulation^2,11^. Vertebrate ohnologues have gained more complex regulation and specialized in expression during evolution^12,13^. The vertebrate 1R (~530 million years ago (Ma))^5–8^ and teleost 3R (~275 Ma)^14,15^ are proposed autotetraploidizations, where a diploid genome doubled within-species, leading to four homologous chromosome sets that paired non-preferentially during meiosis, resulting in tetrasomic inheritance^1^. Rediploidization follows such WGD events and involves a return to stable preferential bivalent pairing, resulting in the disomic inheritance of diploid ohnologue pairs that can diverge in sequence and function^1,16,17^.

The salmonid ancestor underwent autotetraploidization 80–100 Ma, representing a 4R WGD^18–21^. Around half of salmonid genes are retained as functional 4R ohnologue pairs^21,22^, located in large collinear regions that have diverged in sequence to a highly variable extent^19,21^. This signature results from asynchronous rediploidization^17,19^, which also followed the teleost 3R^15^, and WGDs ancestral to acipenseriforms^23^ and snow carp (Schizothoracine)^24^. Asynchronous rediploidization creates a stratification of ohnologue divergence times within affected genomes, providing a system to explore the functional divergence of these genes at distinct stages of evolution. A large fraction of ohnologue pairs created by the salmonid 4R have evolved divergent expression^21,25^. However, the mechanistic underpinnings of this regulatory evolution remain poorly understood.

In this study, we generated large-scale multiomics data for two salmonids to investigate gene regulatory evolution following the 4R WGD.

## Results

### Functional and comparative annotation of salmonid genomes

**Fig. 1:**
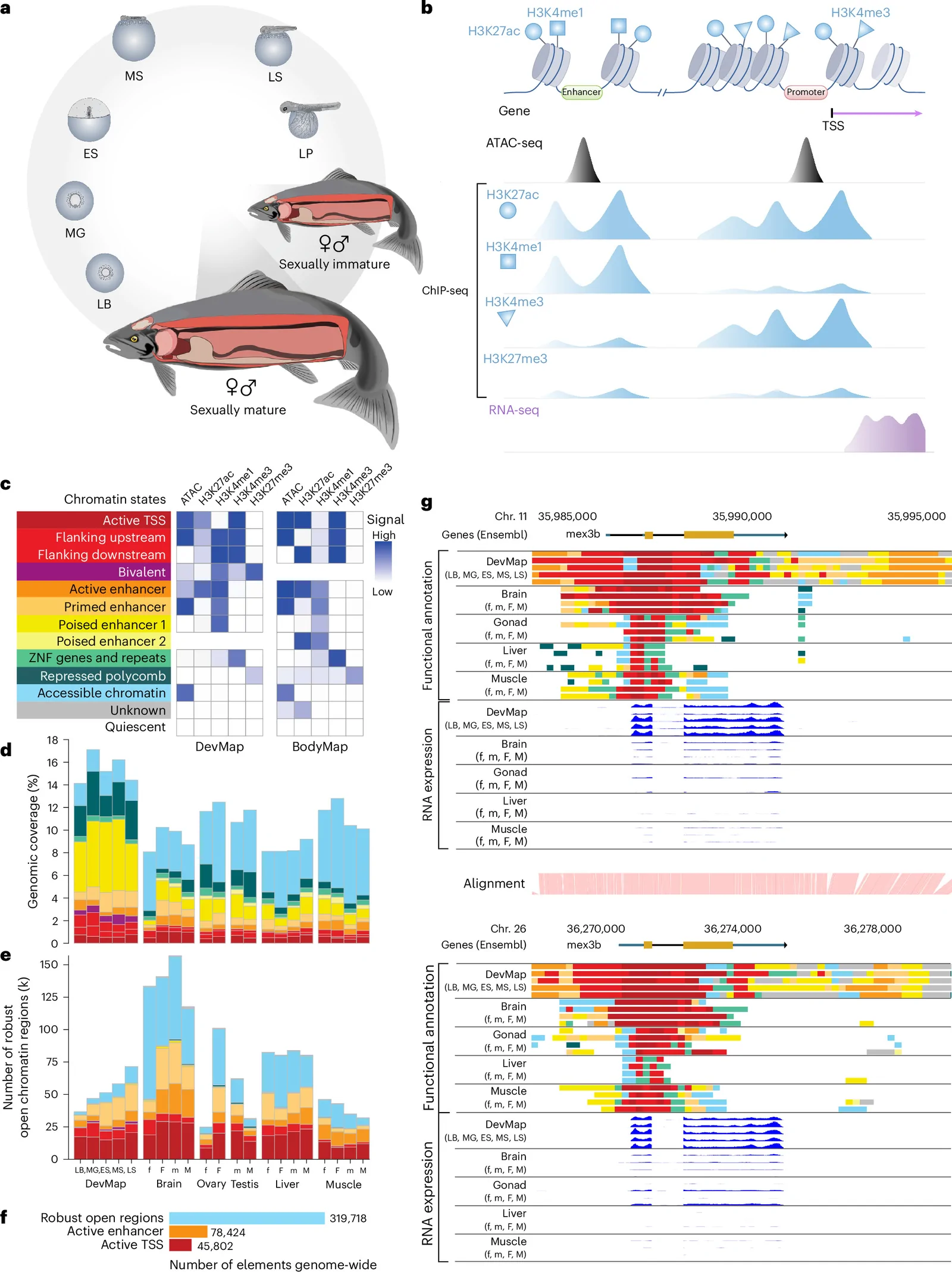
Regulatory annotation of salmonid genomes. **a**, Data shown for salmon (equivalent data for trout shown in Extended Data Fig. 1); an overview of sampling spanning embryogenesis (DevMap) and adult tissues at two stages of sexual maturation (BodyMap). **b**, Regulatory elements were profiled using ATAC-seq and ChIP-seq and gene expression using RNA-seq. **c**, The 12-state ChromHMM models for DevMap and BodyMap datasets, including signal intensity for individual functional assays per chromatin state. **d**,**e**, Coverage of the genome (**d**) and number of robust open chromatin regions (**e**) per chromatin state across samples (DevMap, BodyMap with tissue samples separated into immature (lowercase ‘f’ or ‘m’) and mature (uppercase ‘F’ and ‘M’) for females and males, respectively). **f**, The number of robust open chromatin regions across the salmon genome, including those comprising active promoters and enhancers according to our ChromHMM annotation. **g**, The integration of functional annotation data for a pair of *mex3b* ohnologues (Ensembl identifiers: ENSSSAG0000008892 and ENSSSAG00000085168) encoding a conserved RNA-binding protein. Predicted chromatin states (coloured as in **c**) and corresponding gene expression are visualized across samples. This example illustrates conservation of regulatory elements between ohnologues that correlate with conserved gene expression. Equivalent data for trout *mex3b* ohnologues is provided in Extended Data Fig. 3. LB, late blastulation; MG, mid gastrulation; ES, early somitogenesis; MS, mid somitogenesis; LS, late somitogenesis; LP, late pharyngula. Images of embryos in **a** adapted from ref. ^111^, Springer Nature. Image of salmonid fish in **a** from Madhusudhan Gundappa. Images in **b** from Ashita Singh.

Following ENCODE^26^ and within the Functional Annotation of Animal Genomes (FAANG) initiative^27,28^, AQUA-FAANG generated genome functional annotations for farmed fish species of global economic importance^29^. In the current study, we obtained species-matched samples from Atlantic salmon and rainbow trout (hereafter salmon and trout), among the world’s most commercially valuable fishes^30^, with wild populations of great conservation importance^31^. Sampling spanned key stages of embryogenesis (‘DevMap’) and a tissue panel from males and females at sexually immature and mature life stages (‘BodyMap’) (Fig. 1a). After developing robust protocols (Supplementary Fig. 1 and Supplementary Texts 1–4) we used RNA sequencing (RNA-seq) to quantify gene expression alongside the assay for transposase-accessible chromatin using sequencing (ATAC-seq)^32^ and chromatin immunoprecipitation sequencing (ChIP-seq) (histone marks: H3K27ac, H3K4me1, H3K4me3 and H3K27me3) to assess chromatin openness and epigenetic state (Fig. 1b). Altogether, we generated 771 datasets (Supplementary Tables 1–3 and Supplementary Figs. 2–9) summing to 77.7 billion read pairs, with raw data shared on the FAANG data portal (https://data.faang.org/projects/AQUA-FAANG)^33^ and processed datasets via https://salmobase.org/ (ref. ^34^).

**Extended Data Fig. 1:**
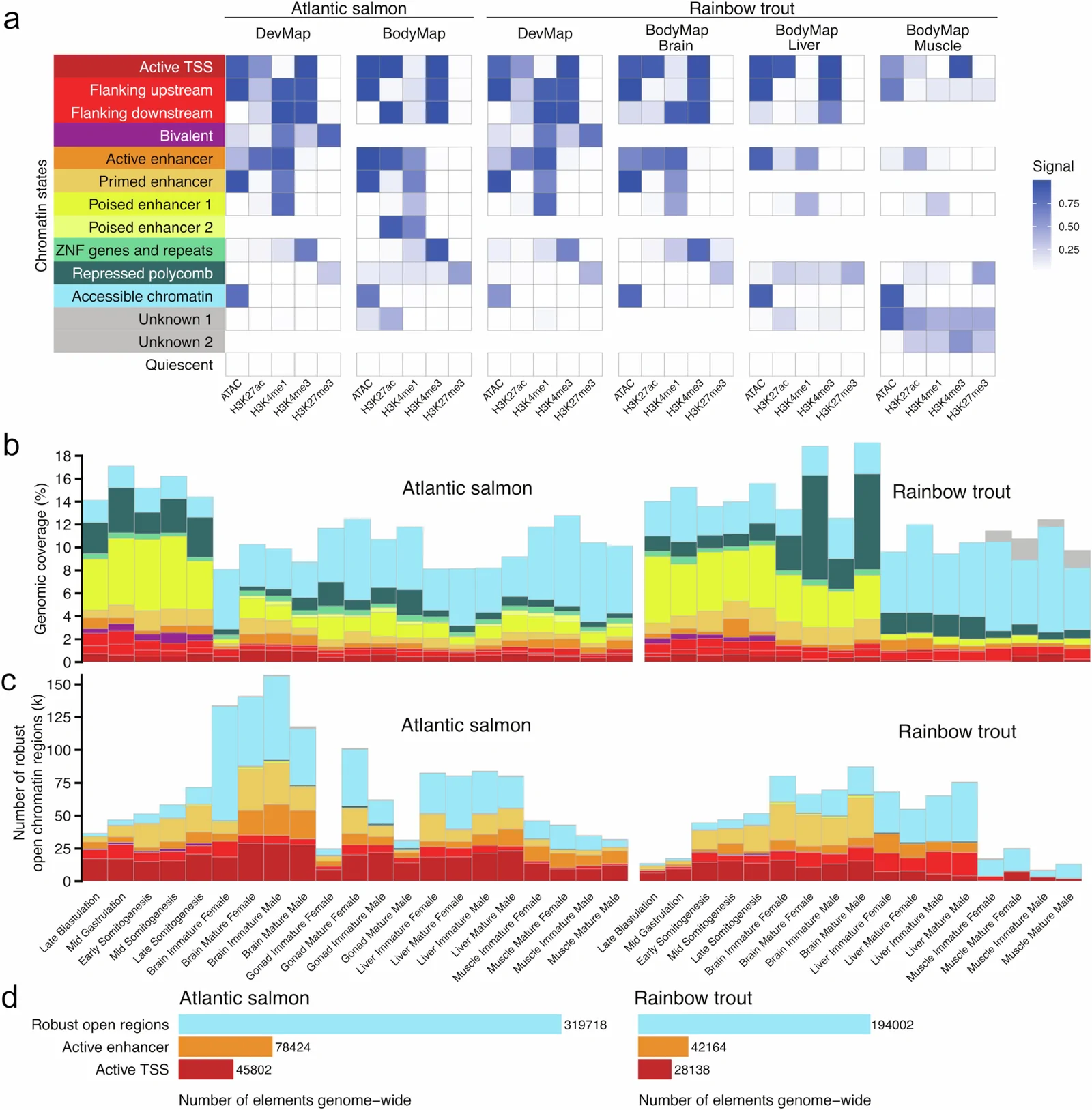
ChromHMM models and robust open chromatin regions defined across two salmonid genomes to predict regulatory elements. (**a**) Comparison of selected ChromHMM chromatin state models for salmon and trout DevMap and BodyMap datasets. (**b**) Sample-specific proportions (values shown are means of biological replicates) of each salmonid genome covered by different ChromHMM chromatin states, colored according to part **a**. (**c**) Sample-specific counts (values shown are means of biological replicates) of robust chromatin regions representing different chromatin states for each salmonid species. (**d**) Counts of genome-wide robust open chromatin regions, including those comprising active enhancer and active promoters (that is, active TSS) for salmon and trout, combined across DevMap and BodyMap.

**Extended Data Fig. 2:**
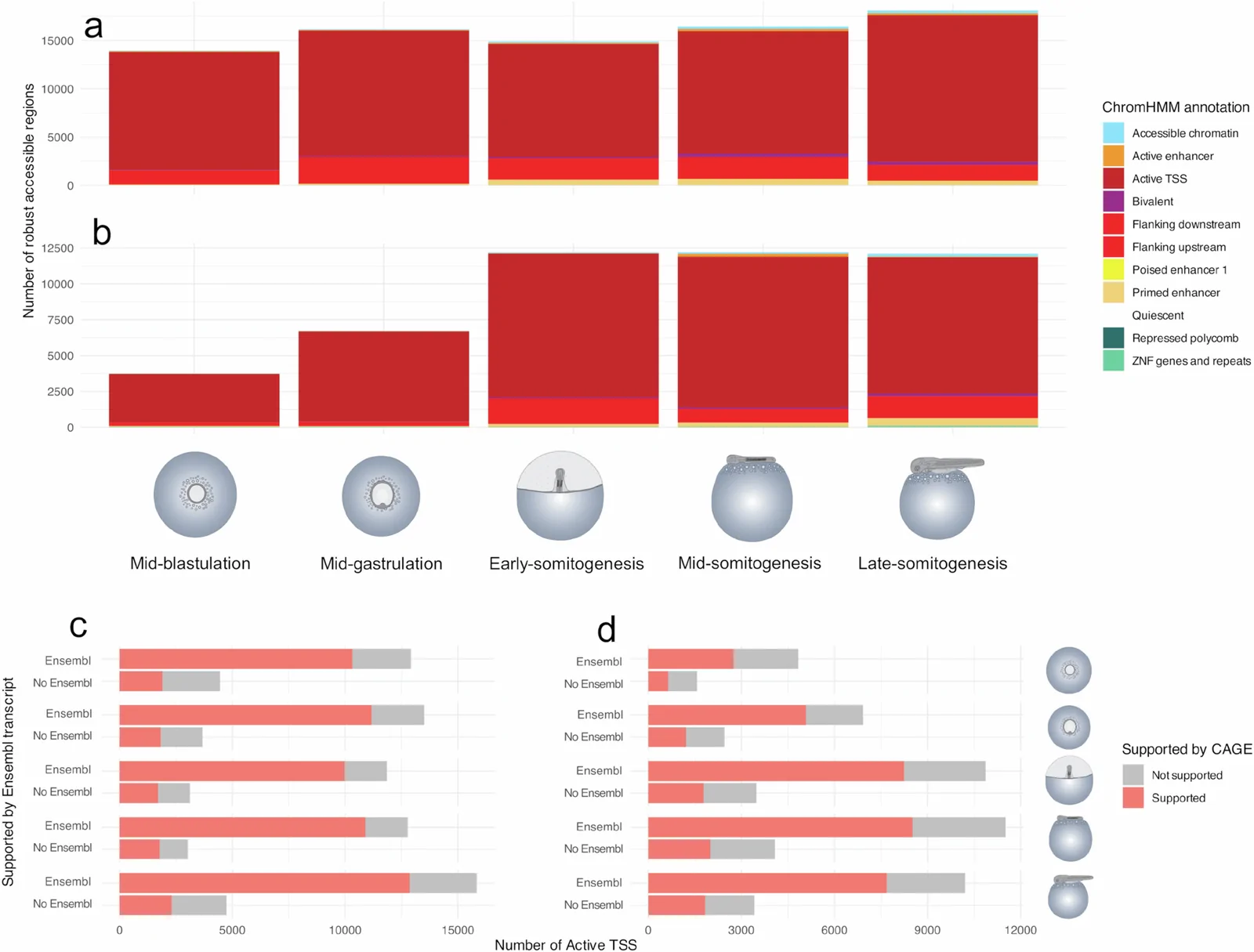
Validation of annotated regulatory elements using CAGE data with DevMap samples. (**a, b**) Overlap between ChromHMM annotated regulatory elements and CAGE promoters in salmon (**a**) and trout (**b**). The majority of overlapping peaks are associated with promoter or promoter-proximal functions, confirming the specificity and accuracy of ChromHMM annotations when independently validated with CAGE. (**c, d**) Bar charts depicting the number of regulatory elements with Ensembl transcript annotation support and without Ensembl transcript annotation support, colored by their CAGE support status, in salmon (**c**) and trout (**d**). While most Ensembl-supported elements are also supported by CAGE data, a subset of elements without Ensembl support show CAGE support, indicating the potential presence of novel, previously unidentified transcripts. Images of embryos adapted from ref. ^109^, Springer Nature.

We identified 319,718 ‘robust’ open chromatin regions in salmon and 194,002 in trout, defined as biologically reproducible ATAC-seq peaks using the irreproducible discovery rate (IDR)^35^ (Supplementary Table 4). These regions harbour regulatory elements bound by transcription factors (TFs) and protein complexes associated with RNA polymerase II^32^. To enrich their functional context, we produced chromatin state models using ChromHMM^36^, integrating ATAC-and ChIP-seq. This identified 45,802 and 28,138 unique active promoters and 78,424 and 42,164 unique active enhancers, in the salmon and trout genomes, respectively (Fig. 1c–f and Extended Data Fig. 1). Cap Analysis Gene Expression (CAGE)^37^ showed that most regions annotated as ‘Active TSS’ were supported by CAGE-defined promoters, with support observed for 83–91% in salmon and 53–70% in trout (Extended Data Fig. 2). Active promoters and enhancers were enriched in TF motifs matching sample-type expectations (Supplementary Figs. 10 and 11 and Supplementary Text 5). For example, the motif targeted by the Pou5f1(Oct4):Sox2 complex, essential for zebrafish zygotic genome activation and gastrulation^38^, was enriched in regulatory elements highly active at the same stages in salmon and trout (Supplementary Fig. 10). We also identified the enrichment of canonical tissue-specific TF motifs in both species and extensive between-species sharing of enriched motifs (Supplementary Text 5 and Supplementary Fig. 12).

**Extended Data Fig. 3:**
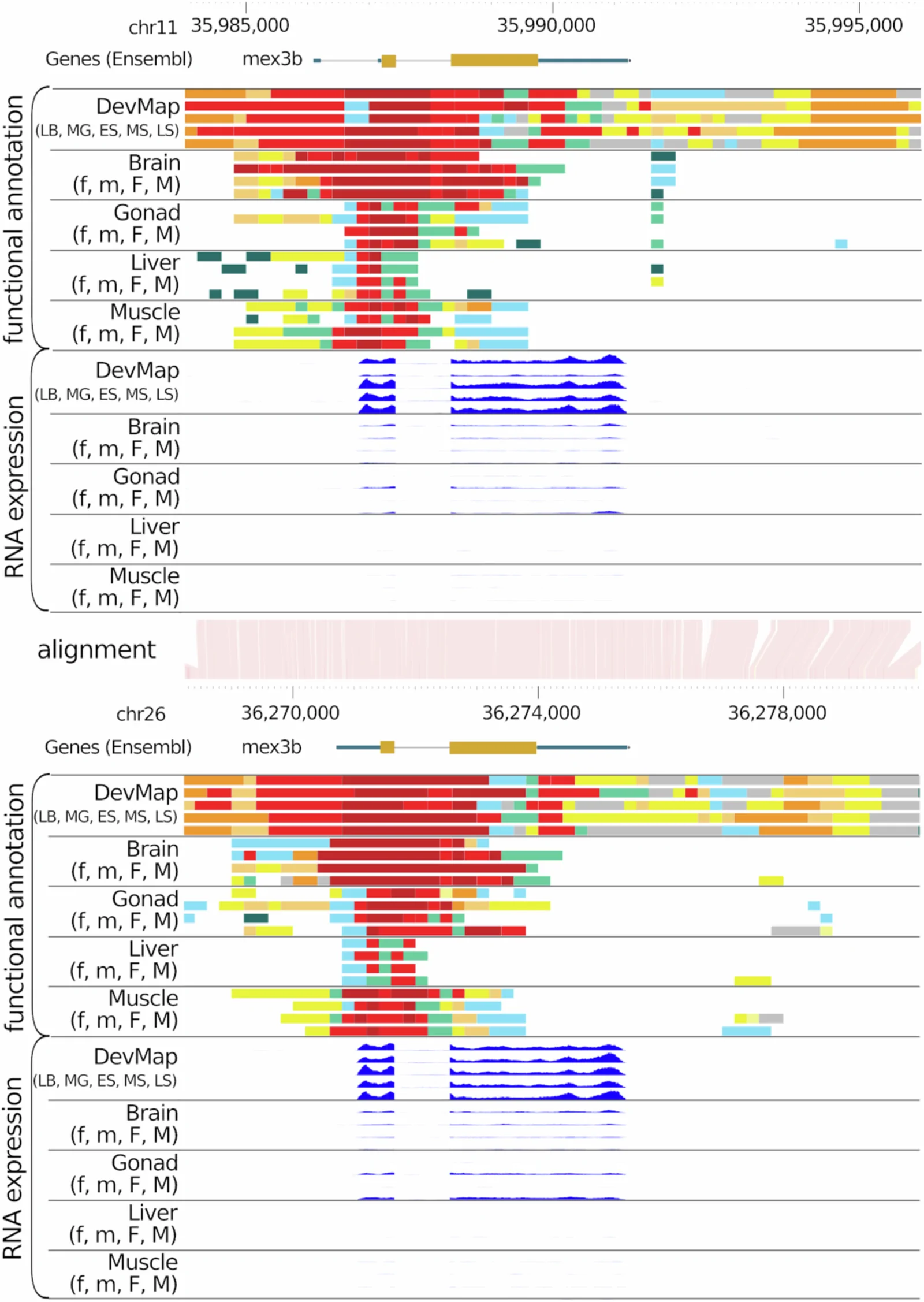
Integration of functional annotation data for a pair of trout ohnologs for *mex3b*. The genes shown are found on Chromosome 6 (ENSOMYG00000040028) and 26 (ENSOMYG00000077707). Predicted chromatin states (colours as per Fig. 1 and Extended Data Fig. 1) and corresponding gene expression is visualized for both DevMap and BodyMap annotations. Equivalent figure for salmon is provided in Fig. 1g.

For comparative analyses, we identified 10,521 ohnologue pairs conserved in both species and 5,832 ‘singletons’, where one ohnologue was lost, using a phylogenomic pipeline leveraging collinearity retained following WGD (Supplementary Tables 5 and 6 and Supplementary Fig. 13). To compare non-coding regulatory regions, we used Cactus^39^ to align duplicated and orthologous sequences within and between the salmon and trout genomes (Methods; Supplementary Fig. 14 and Supplementary Table 7), alongside orthologous regions from northern pike (from a sister lineage that did not undergo 4R^18^). Our comparative dataset captured 26,157 genes and 194,528 unique regulatory elements and is used in later sections to investigate gene regulatory evolution following the 4R WGD (see Fig. 1g and Extended Data Fig. 3 for example utilities).

### Dynamic genome regulation across ontogeny and tissues

**Fig. 2:**
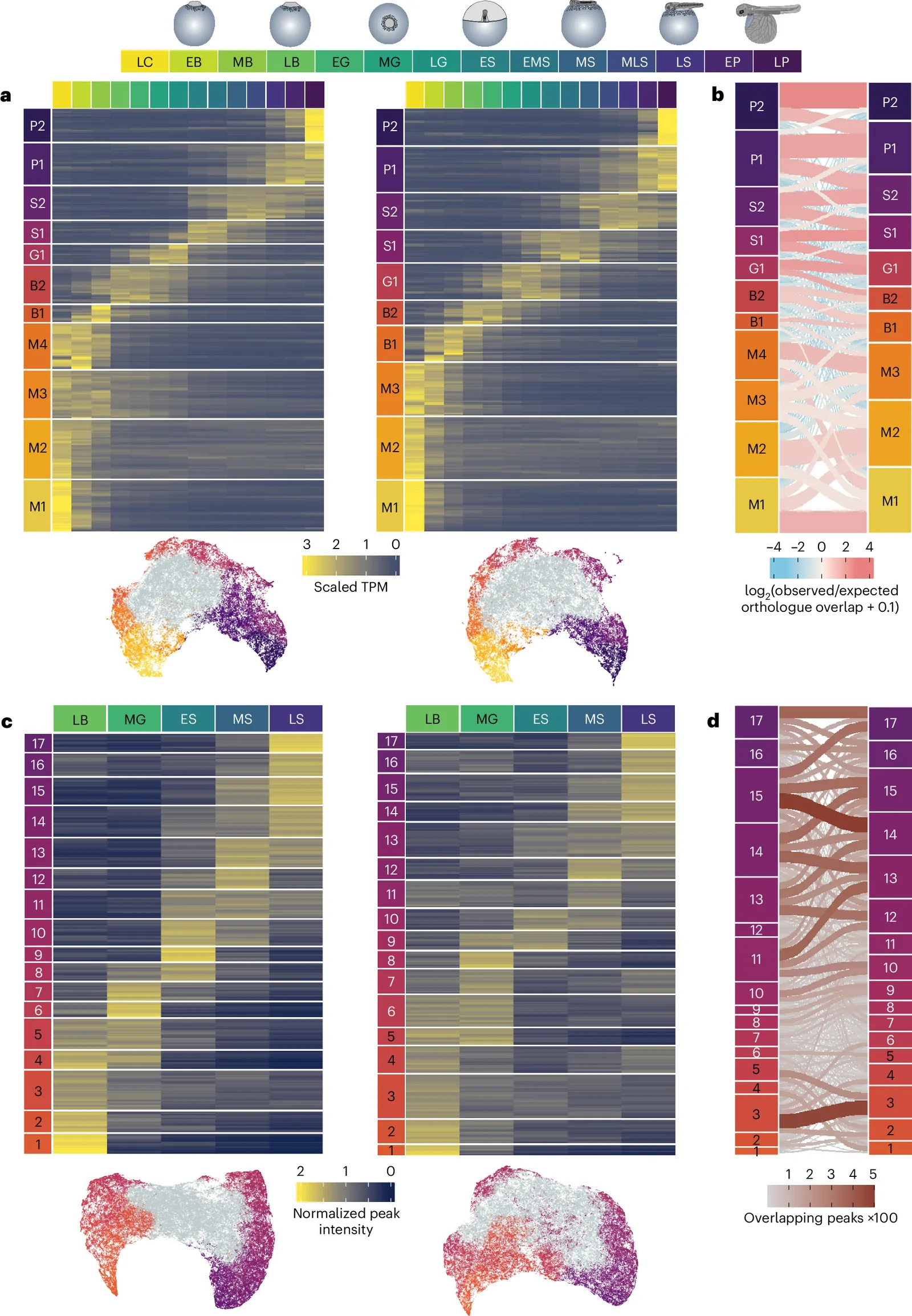
Functional activity of salmonid genomes during embryogenesis. **a**, Transcriptome dynamics during embryogenesis. SOM cluster heat maps for salmon (left) and trout (right) show mean normalized TPM values across developmental stages, with rows split by stage-specific SOM clusters (full data shown in Supplementary Figs. 15 and 16). Cluster labels (M1–M4, B1–B2, G1, S1–S2, P1–P2) indicate maternal (M), blastulation (B), gastrulation (G), somitogenesis (S) and pharyngula (P) expression. Below each heat map, UMAP plots show dimensionality reduction of the multivariate normalized expression profiles across all 14 embryonic stages, where each point represents an individual gene positioned according to its expression profile similarity to other genes. Points are coloured by SOM cluster assignment; grey points indicate genes assigned to constitutive SOM clusters (Supplementary Figs. 15 and 16). A schematic (top) illustrates embryonic stages, coloured by developmental stage: LC, late cleavage; EB, MB, LB, early, mid and late B; EG, MG, LG, early, mid and late G; ES, EMS, MS, MLS, LS, early, early-to-mid, mid, mid-to-late and late S; EP, LP, early and late P. **b**, Chromatin openness dynamics during embryogenesis. SOM cluster heat maps for salmon (left) and trout (right) show mean normalized ATAC-seq read intensity values in robust open chromatin regions across five developmental stages (LB, MG, ES, MS, LS). Rows are split by stage-specific SOM clusters (full data shown in Supplementary Figs. 17 and 18). UMAP plots below show dimensionality reduction of multivariate normalized profiles, where each point represents a single robust open chromatin region. Point colouring as per **a**. **c**, Cross-species comparison of transcriptomic SOM clusters. Sankey diagram shows the number of shared 1-to-1 orthologous genes between salmon (left) and trout (right) stage-specific SOM clusters. The band width is proportional to the number of shared orthologues between each pair of clusters. The band colour represents the log_2_ observed-to-expected ratio of orthologous gene sharing (colour scale; blue, fewer shared orthologues than chance; red, more shared orthologues than chance). Expected values were estimated using 10,000 random permutations of cluster assignments (Methods). **d**, Cross-species comparison of chromatin openness SOM clusters. The Sankey diagram shows the number of syntenic open chromatin regions shared between salmon and trout ATAC-seq SOM clusters, on the basis of whole genome alignment. The band width is proportional to the number of overlapping syntenic open chromatin regions between each cluster pair. The band colour represents the number of shared regions. Images of embryos shown above **a** adapted from ref. ^111^, Springer Nature.

We first classified our global datasets using dimensionality reduction approaches^40^. For DevMap, self-organizing maps (SOM) identified clusters of genes (Fig. 2a and Supplementary Figs. 15 and 16) or genomic regions (Fig. 2b and Supplementary Figs. 17 and 18) showing similar stage-specific changes in expression or chromatin openness across embryogenesis. The maternal-to-zygotic transition (MZT) was captured by clusters of maternal mRNAs showing highest levels during late cleavage or early blastulation, with declining levels thereafter (Fig. 2a, M1–M4 in salmon and M1–M3 in trout), along with genes upregulated (Fig. 2a, B1–B2 and G1, both species) and chromatin regions showing maximal openness (Fig. 2b, clusters 1–7 for salmon and 1–11 for trout) at blastulation and gastrulation stages. Maternal clusters were enriched for Gene Ontology (GO) terms including ‘cell cycle’ and ‘chromosome segregation’, whereas segmentation and pharyngula stages (S1–S2 and P1–P2, both species) were enriched for terms linked to organogenesis and energy production (Fig. 2a, Supplementary Figs. 19 and 20, Supplementary Text 6 and Supplementary Tables 8 and 9). A developmental trajectory of embryogenesis was evident, with topologically related clusters co-localizing on uniform manifold approximation and projection (UMAP) graphs (Fig. 2a,b). Comparing the two species, closely related clusters were enriched in orthologous genes and syntenic regions (Fig. 2c,d and Supplementary Fig. 21).

These analyses were repeated using our BodyMap datasets, capturing changes in gene expression and chromatin openness across tissues, sexes and maturation stages that were similar in both species (Extended Data Fig. 4, Supplementary Figs. 22–29, Supplementary Tables 8 and 9 and Supplementary Text 7).

Overall, the AQUA-FAANG datasets capture dynamic changes in genome activity across development and tissues, providing a powerful framework to dissect gene regulatory evolution following WGD.

### Ohnologue expression divergence mirrors constraints observed across species

**Fig. 3:**
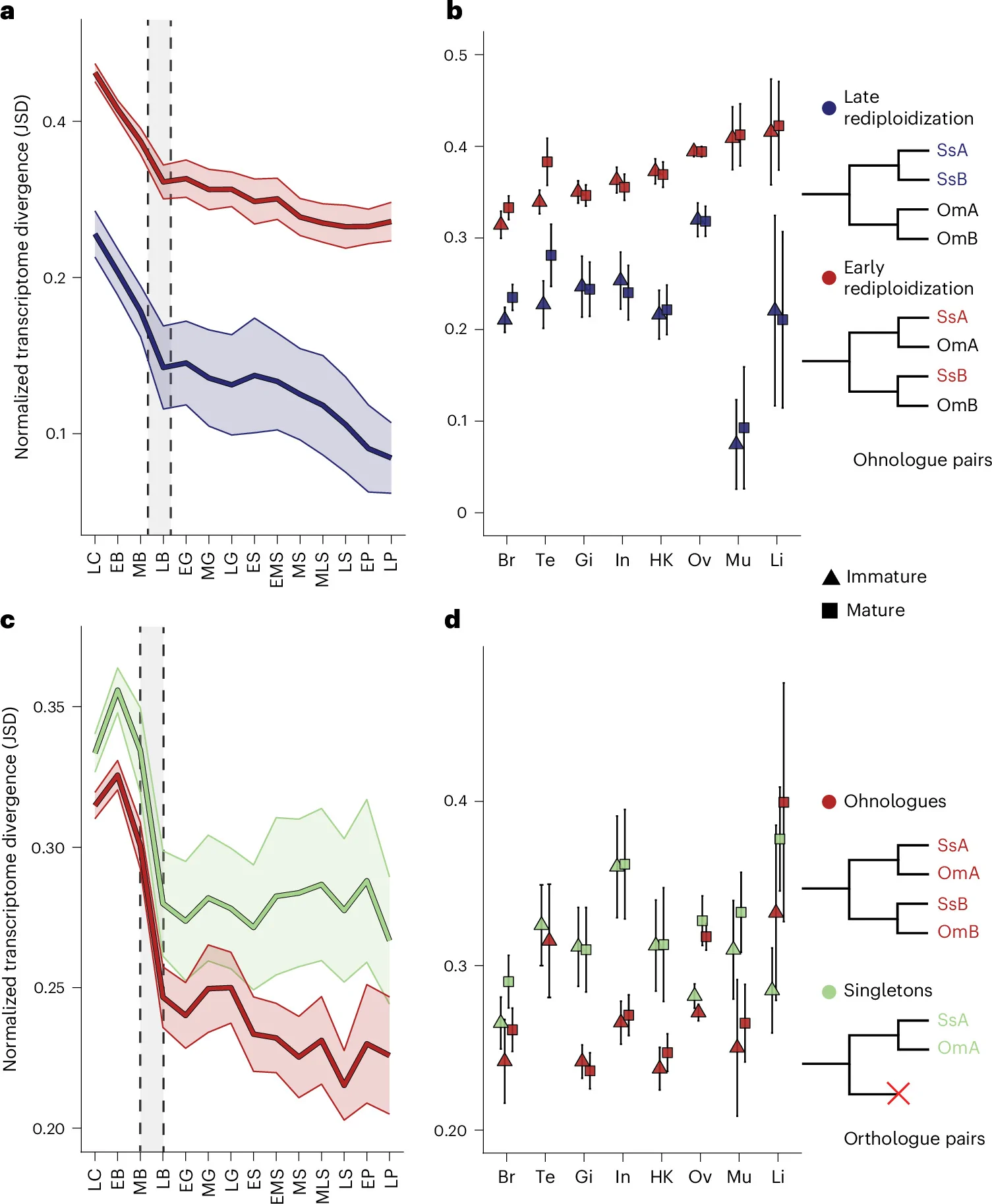
Gene expression evolution following the salmonid 4R autotetraploidization. **a**,**b**, Mean normalized JSD ± 1 s.d. (100 bootstrap samples) between salmon ohnologue pairs across 14 stages of embryonic development (DevMap) (**a**) (grey area between dashed lines represents the MZT period) or adult tissues of immature and mature fish (triangles and squares, respectively) (BodyMap) (**b**). Lines are coloured by early (red) and late (blue) rediploidization categories. **c**,**d**, Mean JSD ± 1 s.d. for orthologue pairs from salmon and trout for two groups representing ohnologues (retained as pairs in both species) or singletons (one gene lost in same ohnologue pair in the salmon–trout ancestor) for the same DevMap (**c**) and BodyMap (**d**) samples. JSD was calculated from quantile-normalized TPM values. Higher normalized mean JSD indicates greater expression divergence between gene pairs. Centre: mean. Error bars and bands, ±1 s.d. across 100 bootstrap resamples of gene pairs. The unit of analysis is the ohnologue or orthologue gene pair, and per-sample expression is the mean of the biological replicates before pairing. *n* (gene pairs): *n* = 8,757 early rediploidization ohnologue pairs and *n* = 1,103 late rediploidization ohnologue pairs (**a** and **b**); *n* = 8,661 ohnologue group orthologue pairs and *n* = 5,099 singleton group orthologue pairs (**c** and **d**). DevMap stage abbreviations are as per the Fig. 1 legend. BodyMap tissue abbreviations: Br, brain; Te, testis; Gi, gill; In, distal intestine; HK, head kidney; Ov, ovary; Mu, skeletal muscle; Li, liver.

Ontogeny and tissue-specific context influence the rate of gene expression evolution across vertebrates^12,41–47^. To ask if similar constraints follow rediploidization, we quantified salmon ohnologue pair expression divergence using a published strategy^12^ (Fig. 3a,b). To account for asynchronous rediploidization^17,19^, we subclassified our data into ‘early rediploidization’ ohnologue pairs (*n* = 8,757), where ohnologue divergence started before the salmon and trout lineages split-off ~25 Ma^17^ and ‘late rediploidization’ ohnologue pairs (*n* = 1,103), where ohnologue divergence occurred independently in salmon and trout^17^.

As expected, rediploidization was the dominant factor explaining ohnologue expression divergence^17^, with early pairs showing greater divergence for all sample types (Fig. 3a,b). Ohnologue expression divergence decreased across embryogenesis, being highest before the MZT and lowest at pharyngula stages (Fig. 3a), thought to represent the vertebrate phylotypic period^44^, where transcriptomic profiles across species show the highest degree of conservation across ontogeny^41,44,46^. The pattern of decreasing expression divergence across embryogenesis was highly correlated between early and late rediploidization ohnologue pairs (Pearson’s *R* = 0.97, *P* = 1.1 × 10^−8^; Supplementary Fig. 29), albeit with a more pronounced drop in divergence for late pairs at advanced segmentation and pharyngula stages (Fig. 3a).

Adult tissue types differed extensively in ohnologue expression divergence, with brain showing the lowest divergence for early ohnologue pairs (Fig. 3b), consistent with past work^47^. Sexual maturation had little effect on ohnologue expression divergence for most tissues, including ovaries (Fig. 3b). However, ohnologue expression divergence was higher in mature versus immature testis (Fig. 3b). Expression divergence across tissues was not correlated between early and late ohnologue pairs (Supplementary Fig. 29), explained by increased divergence in ovaries and reduced divergence in muscle for late pairs, indicative of the weakest and strongest respective levels of purifying selection on the dosage of young ohnologues among the profiled tissues.

**Extended Data Fig. 5:**
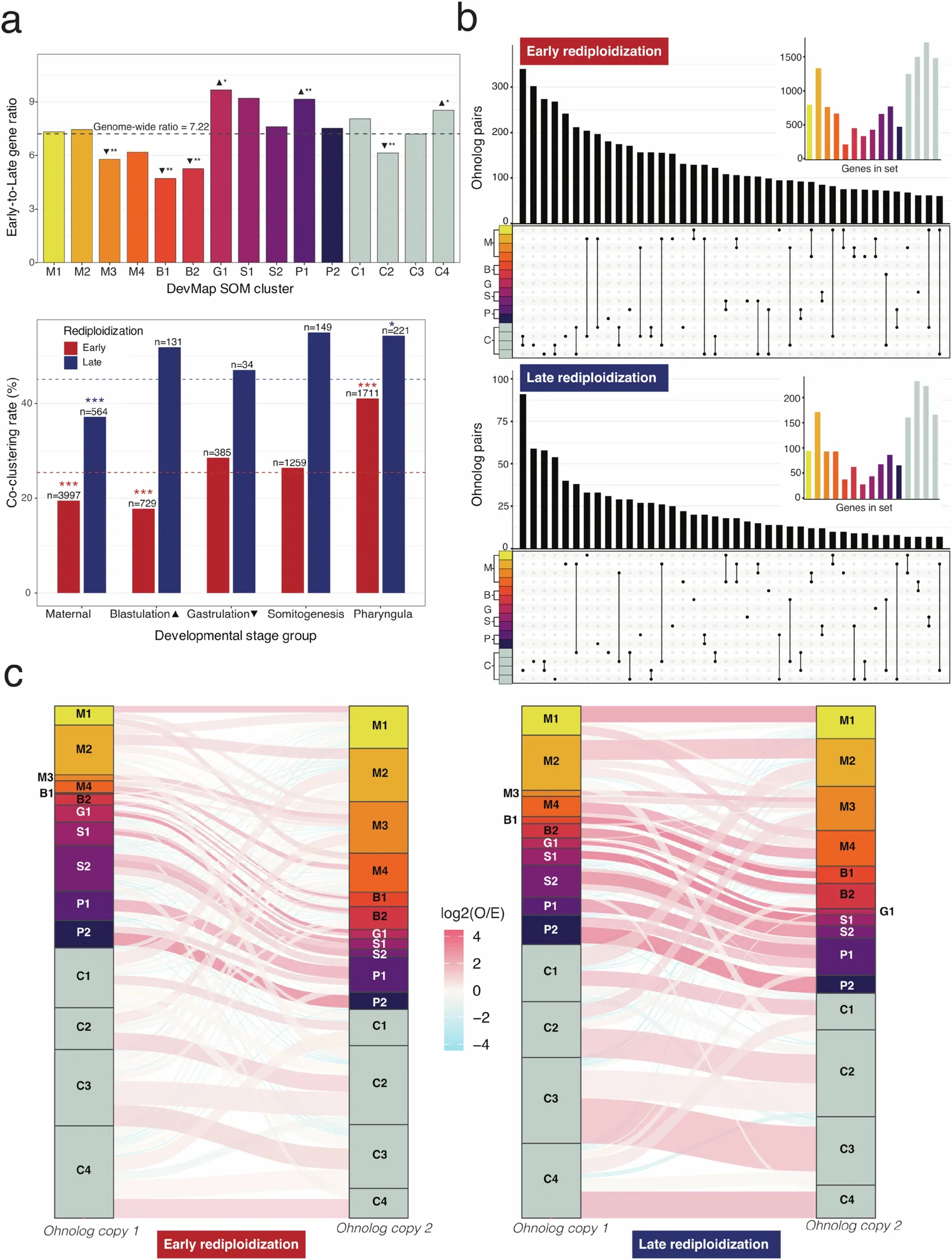
Ohnolog partitioning across salmon DevMap transcriptomic SOM clusters. SOM clusters are labelled by developmental stages (M1 to M4: maternal; B1 to B2: blastulation; G1: gastrulation; S1 to S2: somitogenesis; P1 to P2: pharyngula; C1 to C4: constitutive). (**a**) Within-cluster composition and co-clustering of Early and Late rediploidization ohnolog pairs. Top: per-cluster relative proportion of Early and Late rediploidization genes across the 15 DevMap SOM clusters. Bar height gives the Early/Late rediploidization gene-count ratio for each cluster. The dashed horizontal line indicates the genome-wide Early-to-Late ratio (=7.22). Filled triangles and asterisks above bars indicate per SOM cluster significant enrichment (up triangles) or depletion (down triangles) of Early ohnologs relative to this baseline (binomial test, one, two and three asterisks denote p-adj <0.05, 0.01 and 0.001, respectively). Bottom: per ohnolog pair co-clustering rate aggregated across developmental stage groups. Bar height indicates the percentage of co-clustered ohnolog pairs in each rediploidization class. Dashed horizontal lines indicate the class-wide mean ohnolog pair co-clustering rate across non-constitutive clusters. Asterisks above bars mark developmental stage groups where the co-clustering rate deviates significantly from its group mean (proportion test, asterisks as per top panel). (**b**) UpSet plots of the most common SOM-cluster membership for Early (top) and Late (bottom) rediploidization ohnolog pairs, with SOM cluster identity collapsed to dominant developmental stages (M, B, G, S, P, C). Each vertical bar represents a unique combination of stages across which the two genes forming an ohnolog pair are distributed, with bar heights giving the number of pairs for shown combinations. Dots in the matrix below each bar indicate which stages contribute to that combination: a single dot marks pairs with both ohnolog copies in clusters of the same dominant stage, and two connected dots mark cross-stage ohnolog pairs. Inset bars at the top-right give the number of ohnolog gene copies contributing to each developmental stage group. (**c**) Alluvial (Sankey) diagrams of ohnolog pair-level SOM cluster transitions, shown separately for Early (left) and Late (right) rediploidization ohnolog pairs. Ribbons connecting two SOM clusters represent the proportion of ohnolog pairs with one copy in each cluster. Ribbon colour indicates pair counts higher (red) or lower (blue) than expected under random reassignment of pairs to cluster combinations. Images of embryos in **a** adapted from ref. ^111^, Springer Nature.

**Extended Data Fig. 6:**
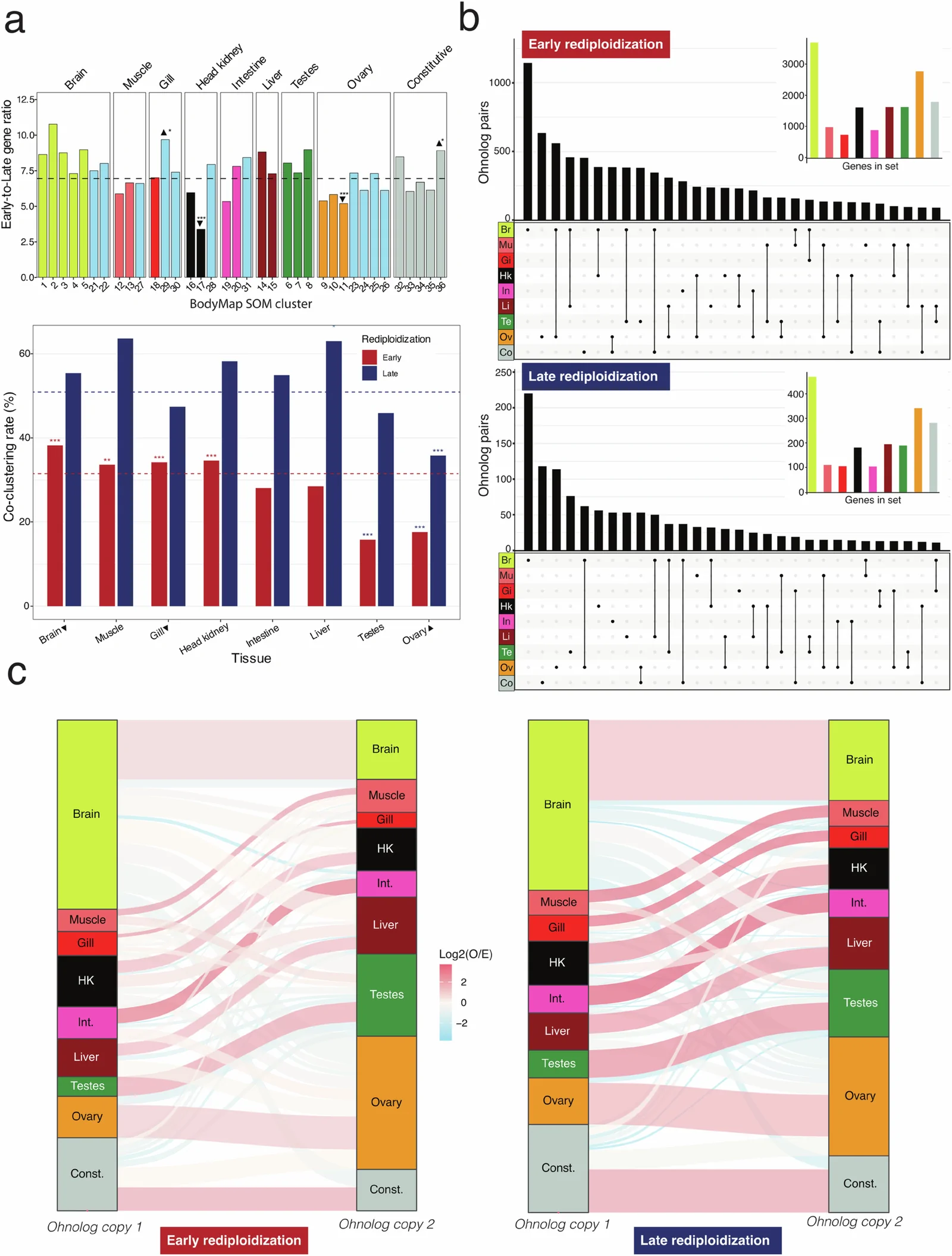
Ohnolog partitioning across salmon BodyMap transcriptomic SOM clusters. SOM clusters are labelled by tissue (brain, muscle, gill, head kidney, intestine, liver, testes, ovary, constitutive). All other details match directly to the legends for equivalent panels (**a-c**) in Extended Data Fig. 5. Statistics were computed as in Extended Data Fig. 5, on the BodyMap data (n = 10,817 genes in two-copy ohnolog pairs with both copies assigned to a SOM cluster): per-cluster enrichment or depletion of Early ohnologs relative to the genome-wide Early:Late ratio (=6.96) using a two-sided binomial test with Benjamini-Hochberg adjustment, and per-tissue co-clustering rate versus the class mean by two-sided proportion test with Benjamini-Hochberg adjustment (one, two and three asterisks denote P-adj < 0.05, 0.01, 0.001). Across the panel, the Early:Late gene composition differed among SOM clusters (chi-squared test, P = 2.9 × 10^−26^); Early and Late ohnolog pairs differed in overall co-clustering rate (two-sided Fisher’s exact test, P < 10^−35^, via the chi-squared approximation as in Extended Data Fig. 5); and Early-pair co-clustering differed across tissues (chi-squared test of co-clustering-rate homogeneity, P = 2 × 10^−94^).

The proportion of early versus late rediploidization pairs assigned to different SOM clusters varied markedly for DevMap (Extended Data Fig. 5a) and BodyMap (Extended Data Fig. 6a). For DevMap, we observed an enrichment of early rediploidization genes in gastrulation and pharyngula clusters but depletion in maternal and blastulation clusters (binomial tests, adjusted *P*(*P*_adj_) < 0.05). Early pairs co-occurred in the same clusters less frequently than late pairs (DevMap, 26.0 versus 44.9%; BodyMap, 28.7 versus 49.1%; Fisher’s exact test: both *P* < 10^−35^). Co-clustering of early pairs increased across embryogenesis from maternal (19.5%) to pharyngula (41.1%) stages (Cochran–Armitage trend test *χ*^2 ^= 269, *P* = 2 × 10^−60^) and varied 2.4-fold across adult tissues (testes, 15.8%, to brain, 38.2%; differences across tissues; *P* = 2 × 10^−94^). Late pairs showed similar co-clustering patterns but with weaker effect sizes. The ohnologue pairs with copies assigned to different SOM clusters typically involved at least one constitutive cluster (Extended Data Figs. 5b,c and 6b,c). Early pairs were split across stage or tissue SOM cluster groups more often than late pairs (55 versus 36% for DevMap; 63 versus 52% for BodyMap), consistent with the greater opportunity these genes have had to undergo expression divergence during evolution.

We finally asked if salmonid 4R ohnologue expression divergence mirrors across-species expression evolution using 1-to-1 orthologue pairs from salmon and trout (Fig. 3c,d). To capture potential effects of ohnologue retention or loss, we distinguished 8,661 orthologue pairs representing ohnologues retained in salmon and trout from 5,099 orthologue pairs representing singletons in both species. Expression divergence of orthologue pairs from the ohnologue group was highly correlated with that of salmon ohnologue pairs across embryogenesis (Pearson’s *R* = 0.96, *P* = 1.0 × 10^−7^) but weakly correlated across tissues (Pearson’s *R* = 0.54, *P* = 0.037) (Supplementary Fig. 29). Expression divergence of orthologue pairs from both the ohnologue and singleton groups decreased across embryogenesis, with a strong drop around the MZT, matching salmon ohnologue pairs (Fig. 3a-c). However, orthologue pairs from the ohnologue group showed comparatively reduced expression divergence during segmentation stages (Fig. 3c). As observed following many WGD events^48,49^, ohnologue pairs retained from the salmonid 4R are enriched for dosage-sensitive functions^19^. Considering the striking correlation between expression divergence for ohnologue pairs versus orthologues of the same genes, our results suggest that genes expressed from mid-to-late segmentation are not only enriched for dosage-sensitive functions but also that this property selects for ohnologue retention.

Across tissues, we observed variable and often significant differences in how orthologue pairs have evolved across species, depending on whether they represent ohnologues or singletons (Fig. 3d). For gill, head kidney and distal intestine, orthologue pairs from the ohnologue group showed significantly lower expression divergence than singletons (Fig. 3d). However, in brain, expression divergence of orthologue pairs was similar for both groups, requiring a comparatively slower evolution of singleton orthologues (Fig. 3d). Specific to ovaries, the expression divergence of orthologue pairs across species was driven by maturation stage rather than by retention status following WGD (Fig. 3d).

These results reveal that the 4R WGD has played a central role in regulatory evolution in salmonids, shaping variation in gene expression divergence across developmental stages and tissues, both within the same genome and following speciation. In both contexts, a period of maximal constraint is inferred during advanced stages of embryogenesis, where pleiotropic genes are intensively recruited across multiple developing cell and organ types, creating a highly interconnected regulatory environment proposed to limit evolvability^41,50^. Our results suggest these pleiotropic constraints act post-WGD and are a driver for ohnologue retention and regulatory evolution.

### Functional evolution of regulatory elements retained from autotetraploidization

**Fig. 4:**
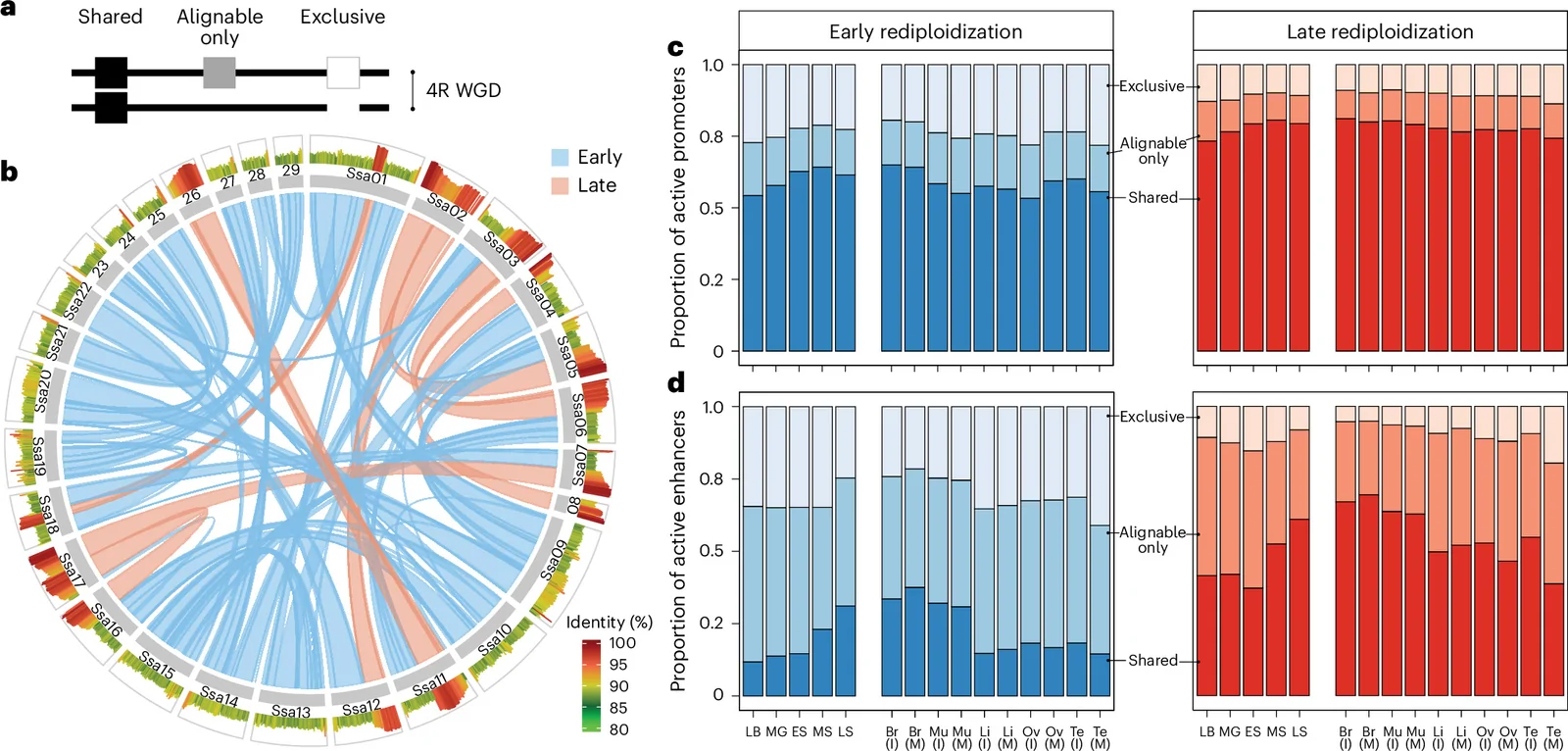
Regulatory element evolution following the salmonid 4R autotetraploidization. **a**, Schematic of shared, alignable only and exclusive regulatory elements, categorized by whole genome alignment. **b**, Circos plot of 29 salmon chromosomes. Ribbons show collinear regions retained from the 4R WGD, with blue and red colours indicating early and late rediploidization regions, where duplicated regions share a respective 88.6% (s.d. 1.51%) and 95.1% (s.d. 2.67%) nucleotide sequence identity. The outermost sections show percentage nucleotide sequence identity (as per key) between collinear blocks at 1-Mb intervals. **c**,**d**, For each alignment (shared, alignable only and exclusive) and rediploidization (early and late) category, we present: the proportion of active promoters (**c**) and enhancers (**d**) in each sample type. Sample name abbreviations are as in Fig. 1–3, except with sexually immature (I) and mature (M) stages separated for adult tissues.

Using whole genome alignment, we divided all promoters and enhancers from salmon into three categories of post-WGD evolution: ‘shared’—where two duplicated sequences retained from WGD are aligned and both shows activity; ‘alignable only’—where, despite being aligned, activity is restricted to one of two duplicated sequences retained from WGD; and ‘exclusive’—where the active element is limited to one chromosome (Fig. 4a). To capture the rediploidization process, we separated these elements on the basis of genomic locations showing early or late rediploidization, which have diverged in sequence to very different levels^48^ (Fig. 4b and Supplementary Tables 10 and 11).

Overall, 53.5%, 21.6% and 24.9% of active promoters belonged to the shared, alignable only and exclusive categories, respectively (Supplementary Table 12). This proportion of shared promoters is consistent with the estimated 55% retention rate for salmonid 4R ohnologue pairs^21^. For active enhancers, 33.3%, 43.8% and 22.9% belonged to the same categories (Supplementary Table 12). Compared with promoters, the reduced proportion from the shared category, alongside higher proportion from the alignable only category (both *P* < 0.0001, Fisher’s exact test) supports a faster comparative evolutionary turnover of enhancers following WGD, as observed across species^51,52^.

Early rediploidization regions harboured proportionately fewer shared promoters and enhancers, alongside more from the exclusive category (Supplementary Text 8). Thus, a longer period of divergence has facilitated the loss of duplicated regulatory elements, while providing more opportunities for the gain of regulatory elements post-WGD.

### Developmental and tissue-specific constraints on regulatory element evolution

We next explored variation in the functional retention of regulatory elements across sample types (Fig. 4c,d and Supplementary Tables 13 and 14), revealing that promoters were active in more sample types than enhancers (Supplementary Fig. 30), consistent with past work^53^. The breadth of regulatory element activity across sample types and extent of sample type-specific activity varied markedly across alignment categories (Supplementary Figs. 30 and 31 and Supplementary Text 8). Mirroring ohnologue expression divergence (Fig. 3a,b), rediploidization was the dominant factor determining the proportion of shared promoters and enhancers across sample types (Fig. 4c,d).

The proportion of active promoters per alignment category showed limited variation across sample types, with shared promoters most common (Fig. 4c and Supplementary Text 8). Greater sample type heterogeneity was observed for enhancers (Fig. 4d). During embryogenesis, the highest proportion of Shared enhancers occurred at late somitogenesis, reaching a proportion similar to brain—the highest among adult tissues (Fig. 4d). Mature testis showed the highest proportion of exclusive enhancers, whereas liver, ovary and testis showed the lowest proportion of Shared enhancers (Fig. 4d).

Thus, the functional retention of enhancers following the salmonid 4R was strongly shaped by development and tissue-specific context, whereas promoter evolution was less dynamic.

### Enhancer evolution is coupled to ohnologue expression regulation

Enhancers shape transcriptional regulation and their conservation is strongly linked to gene expression evolution^51,52,54–56^. Does the loss, retention or gain of enhancer function also explain ohnologue expression evolution following WGD? Although a past study proposed that enhancers are involved in ohnologue expression divergence following the salmonid WGD^57^, this conclusion was based on a single histone mark and tissue, whereas the retention of duplicated enhancer sequences was overlooked. We used our broad sample panel, regulatory atlases and genome alignment to dissect how enhancer activity and sequence conservation shape ohnologue expression evolution.

We first asked if enhancer conservation correlates with ohnologue expression divergence across sample types. As ohnologue expression divergence increased, the proportion of shared enhancers decreased, whereas the proportion of alignable only and exclusive enhancers increased (Supplementary Table 15). Although broadly consistent with enhancer conservation or loss contributing to ohnologue expression divergence, these trends were nonsignificant (Pearson’s *R* = −0.41, 0.39, 0.37 for shared, alignable only and exclusive enhancers, respectively; *P*_adj_ > 0.17). Equivalent tests for promoters were significant in the same directions (Pearson’s *R* = −0.72, 0.78 and 0.59; *P*_adj_ = 0.004, 0.002 and 0.02, respectively), indicating, as expected, a stronger role for promoters in ohnologue expression evolution.

**Fig. 5:**
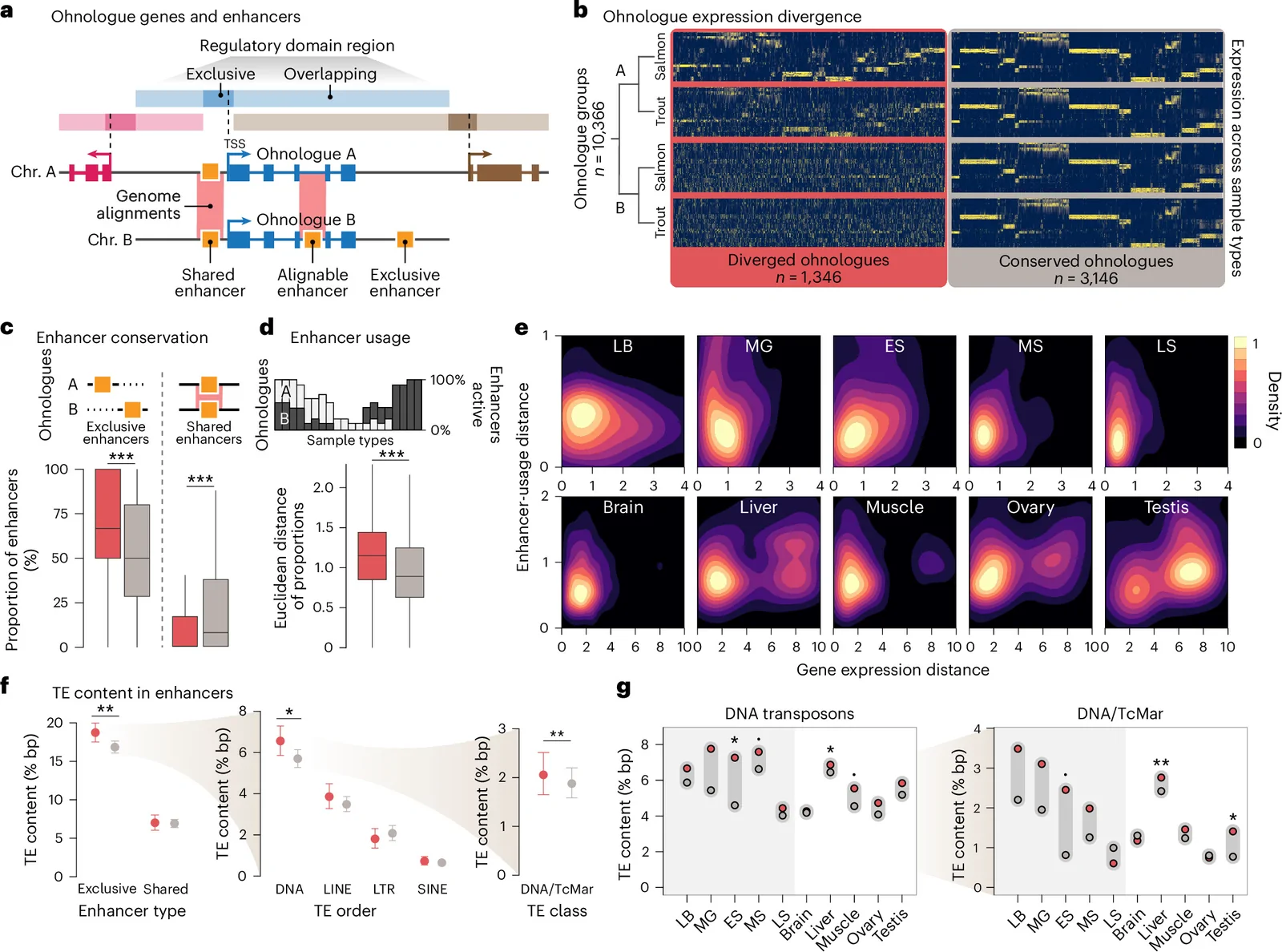
Enhancer evolution is coupled to ohnologue regulatory divergence. **a**, Our definition of gene *cis*-regulatory domains^51^ includes an ‘exclusive’ region −5 to +1 kb surrounding the TSS and an ‘overlapping’ region, extending until a neighbouring exclusive region or maximum distance of 1 Mb from the TSS. Active enhancers were compared within these domains for ohnologue pairs from the shared, alignable only and exclusive categories. **b**, Ohnologue pairs were split into ‘conserved’ (grey) or ‘diverged’ (red) regulation subclasses (Extended Data Fig. 7), which were compared in downstream tests. **c**, Enhancer conservation in diverged (*n* = 1,847) and conserved (*n* = 4,720) ohnologues was compared by contrasting the proportion of enhancers belonging to the exclusive (odds ratio, 1.61, 95% confidence interval 1.50–1.74, *P* = 1.7 × 10^−36^), alignable only (Supplementary Fig. 32) and shared (odds ratio, 0.49, 95% confidence interval 0.43–0.56, *P* = 1.6 × 10^−25^) categories using two-sided Wald tests from binomial generalized linear models (logit link) with robust standard errors clustered by ohnologue (Holm-adjusted across the three alignment categories). **d**, Enhancer-usage distance (Euclidean distance between ohnologue pairs for sample-specific enhancer activity profiles) in diverged (*n* = 707) and conserved (*n* = 1,978) ohnologues (two-sided Wilcoxon rank-sum test, rank-biserial *r* = 0.28, 95% confidence interval 0.23–0.33, *P* = 1.6 × 10^−28^). **e**, Density maps of ohnologue pair enhancer-usage Euclidean distance versus expression Euclidean distance, split by stage of embryogenesis or adult tissue type (note: *x* axis limited to 4 and 10, *y* axis limited to 1 and 2, respectively). **f**, TE content (percentage of enhancer sequence overlapping TEs) in diverged and conserved ohnologue pairs. Left: exclusive enhancers (*r* = 0.063, 95% confidence interval 0.021–0.104, *P* = 0.0031; *n* = 1,043 diverged and *n* = 2,458 conserved ohnologue pairs, respectively) and shared enhancers (*n* = 297 diverged ohnologue pairs, 1,204 conserved ohnologue pairs). Centre: exclusive enhancer content split by different TE orders (for DNA order TEs, *r* = 0.043, 95% confidence interval 0.001–0.085, *P* = 0.038). Right: exclusive enhancer TE content in the DNA/TcMar class (*r* = 0.048, 95% confidence interval 0.006–0.089, *P* = 0.0051). Diverged versus conserved ohnologue regulation classes were compared by two-sided Wilcoxon rank-sum tests; *P* values are unadjusted. The centre and right comparisons used the same exclusive enhancer pairs as the left. **g**, TE content in diverged versus conserved exclusive enhancers across embryogenesis and adult tissue types, as tested in **f**. Left: DNA transposon content was significantly different for early somitogenesis (ES) (*r* = 0.109, 95% confidence interval 0.008–0.223, *P* = 0.030) and liver (*r* = 0.062, 95% confidence interval 0.003–0.120, *P* = 0.023). Right: DNA/TcMar class TE content was significantly different for liver (*r* = 0.062, 95% confidence interval 0.003–0.120, *P* = 0.0024) and testis (*r* = 0.037, 95% confidence interval 0.035–0.109, *P* = 0.037). Additional statistics and *n* values provided in Supplementary Fig. 34. The ohnologue pairs in **d**–**g**, or genes in **c**, are independent biological units; comparisons are observational. The period indicates *P* < 0.1. *P < 0.05, **P < 0.01, ***P < 0.001, unmarked, not significant. Box plots show median (centre line), 25^th^ to 75^th^ percentiles (box), 1.5× interquartile range (whiskers); outliers are not shown. Points on graphs are means. In **f**, error bars show the 95% confidence interval surrounding the mean (percentile method, 10,000 bootstrap resamples); in **g**, grey bars are visual guides only.

**Extended Data Fig. 7:**
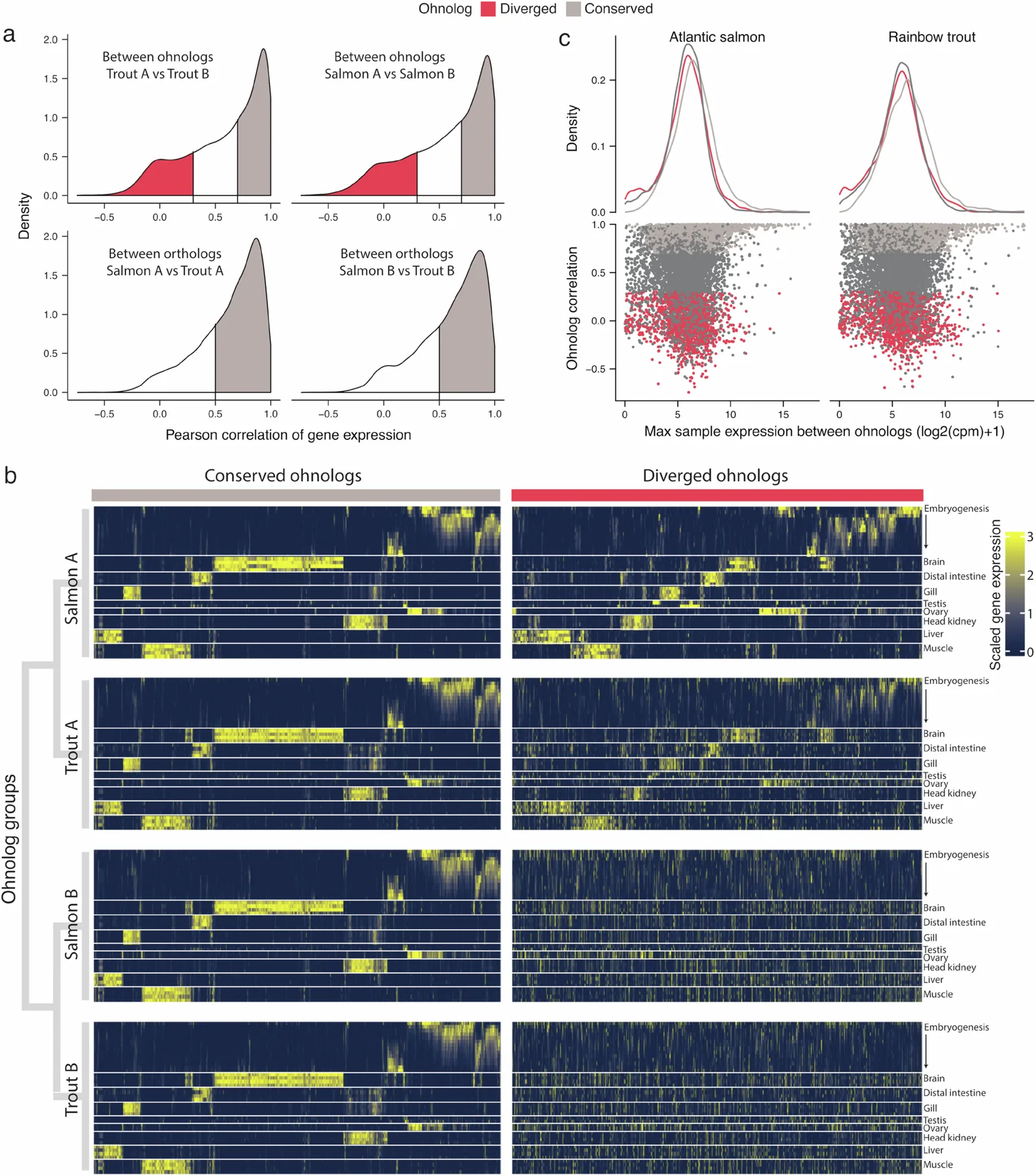
Defining ‘conserved’ and ‘diverged’ expression conservation classes for 2:2 ohnolog pairs. (**a**) Distribution of Pearson’s correlation values calculated between expression profiles between 2:2 ohnolog pairs within species and their matching orthologs from salmon and trout. Threshold for ‘conserved’ ohnologs = R > 0.7 between 2:2 ohnolog pairs (both species) and R > 0.5 between orthologs. Threshold for ‘diverged’ ohnologs = R ≤ 0.3 between ohnolog pairs (both species). (**b**) Heatmap of scaled gene expression across sample types for ‘conserved’ and ‘diverged’ ohnologs in both species. (**c**) Expression level distribution of ohnolog pairs, measured as the maximum read counts per million (CPM) across conditions of both ohnolog pairs (median CPM taken over replicates). The distribution of ohnolog expression levels is shown against the within species ohnolog correlation, as well as the density distribution, for Atlantic salmon and rainbow trout ohnologs.

To directly test if enhancer evolution tracks ohnologue expression divergence, we assigned enhancers to genes using *cis*-regulatory domains^51^ (Methods), before splitting ohnologue pairs into ‘conserved’ and ‘diverged’ regulation classes (Fig. 5a,b and Extended Data Fig. 7a,b). After confirming there was no clear difference in expression levels between regulation classes (Extended Data Fig. 7c), we compared them in terms of enhancer composition and sequence-level conservation following WGD. Although several gene-to-enhancer assignments may be inaccurate owing to long-distance regulation, enhancer proximity to promoters is an accurate predictor for their physical interaction^58^.

Ohnologue pairs with conserved regulation had more active enhancers than pairs with diverged regulation (Wilcoxon test: *P* = 10^−20^; Supplementary Fig. 32), alongside a higher proportion of Shared enhancers (Fig. 5c; *P* = 10^−25^, odds ratio 2.04) and reduced proportion of Exclusive enhancers (*P* = 10^−36^, odds ratio 0.62). Comparing sample type activity, ohnologues with conserved regulation had more shared enhancers active in the same samples (*P* = 10^−6^, Wilcoxon test; Supplementary Fig. 37). Alignable only enhancers were equally present in ohnologues from both regulation classes (Supplementary Fig. 32).

We also quantified differences in total enhancer activity profiles of ohnologue pairs across sample types (Fig. 5d) using Euclidean distance (termed ‘enhancer-usage distance’; Methods). Ohnologue pairs with diverged regulation showed higher enhancer-usage distances than pairs with conserved regulation (medians 1.15 versus 0.89, Wilcoxon test; Fig. 5d).

We finally asked if enhancer-usage distance tracks gene expression distance across sample types on a continuous scale (Methods; Fig. 5e and Supplementary Fig. 33). As embryogenesis proceeds, ohnologue expression distance narrowed (*P* = 10^−7^, *χ*^2 ^= 36.6, d.f. of 4, Kruskal–Wallis tests), consistent with earlier findings (Fig. 3a). Enhancer usage distance, by contrast, showed no systematic decrease across embryogenesis (*P* = 0.685, *χ*^2 ^= 2.26, d.f. of 4). Thus, the total repertoire of enhancers near ohnologue pairs seems unimportant for the extent of gene expression divergence at different embryonic stages, instead suggesting a role for promoters and/or a specific subset of shared enhancers (Fig. 5c).

By contrast, gene expression and enhancer-usage distance were strongly coupled for adult tissues (Fig. 5e and Supplementary Fig. 33). We observed significant differences in ohnologue expression distance (*P* = 10^−35^, *χ*^2 ^= 166.6, d.f. of 4, Kruskal–Wallis tests) and enhancer-usage distance (*P* = 10^−16^, *χ*^2 ^= 77.2, d.f. of 4) across tissues, with brain showing the lowest median values and narrowest interquartile ranges, whereas liver, ovary and testis showed the broadest ranges. Therefore, unlike embryogenesis, tissue-level ohnologue expression divergence is strongly coupled to the remodelling of the total *cis*-proximal enhancer repertoire.

These analyses reveal a coupling between enhancer evolution and ohnologue expression divergence, with clear differences between embryogenesis and adult tissues. Ohnologue expression divergence strongly tracks the loss of shared enhancers and gain of exclusive enhancers. Moreover, the relationship between enhancer evolution and ohnologue expression is consistent with the developmental and tissue-specific constraints shown earlier (Figs. 3 and 4).

### TEs shape enhancer evolution after WGD

How transposable elements (TEs) shape enhancer evolution following WGD remains poorly understood. We overlapped all active enhancers within *cis*-regulatory domains from salmon with a high-quality TE annotation^59^. Exclusive enhancers overlapped TEs >2-fold more than shared enhancers (Fig. 5f), which could be due to TE insertions creating new enhancers^57,59^ or because of reduced purifying selection on exclusive enhancers. To assess the contribution of TEs to ohnologue expression divergence, we contrasted TE-sequence content in enhancers of ohnologue pairs with conserved or diverged regulation (Fig. 5f,g and Supplementary Fig. 34). The proportion of TE sequences in exclusive enhancers was weakly linked to expression divergence (Fig. 5f, *P* = 0.0031; higher in diverged ohnologues), with stronger effects near TSS regions (2–50 Kb) than at distances >100 Kb. However, TE overlap was unlinked to ohnologue expression divergence for shared enhancers (Fig. 5f and Supplementary Fig. 34).

The most active TE group following the salmonid 4R WGD are DNA transposons, specifically the TcMar (DNA/TcMar)^59^. In line with this, the overlap of DNA TEs with exclusive enhancers was increased for diverged ohnologue pairs (Fig. 5f; *P* = 0.038 for all DNA elements; *P* = 0.0051 for DNA/TcMar elements), but not retrotransposons. There was an increased overlap between DNA or DNA/TcMar TEs and exclusive enhancers for ohnologues showing divergent regulation in early somitogenesis, liver and testis (Fig. 5g and Supplementary Fig. 34). Related to this, the contribution of TEs towards gene regulation following the salmonid 4R was proposed to reflect tissue-specific selective constraint^59^. Consistently, we found that enhancers active in brain and late embryogenesis showed the lowest TE overlap across early and late rediploidization categories, whereas increasing TE overlap across sample types was associated with more alignable only/exclusive and fewer shared enhancers (Supplementary Fig. 35 and Supplementary Table 16).

Overall, TEs broadly contributed to regulatory evolution following the salmonid 4R, acting to disable duplicated enhancers and create new enhancers. Moreover, selection against TE insertions was strongest for enhancers retained as duplicates, especially those active in sample types under the most constrained regulatory evolution following WGD (Figs. 3 and 4).

### Ontogeny governs the evolution of enhancer-CNEs following WGD

**Fig. 6:**
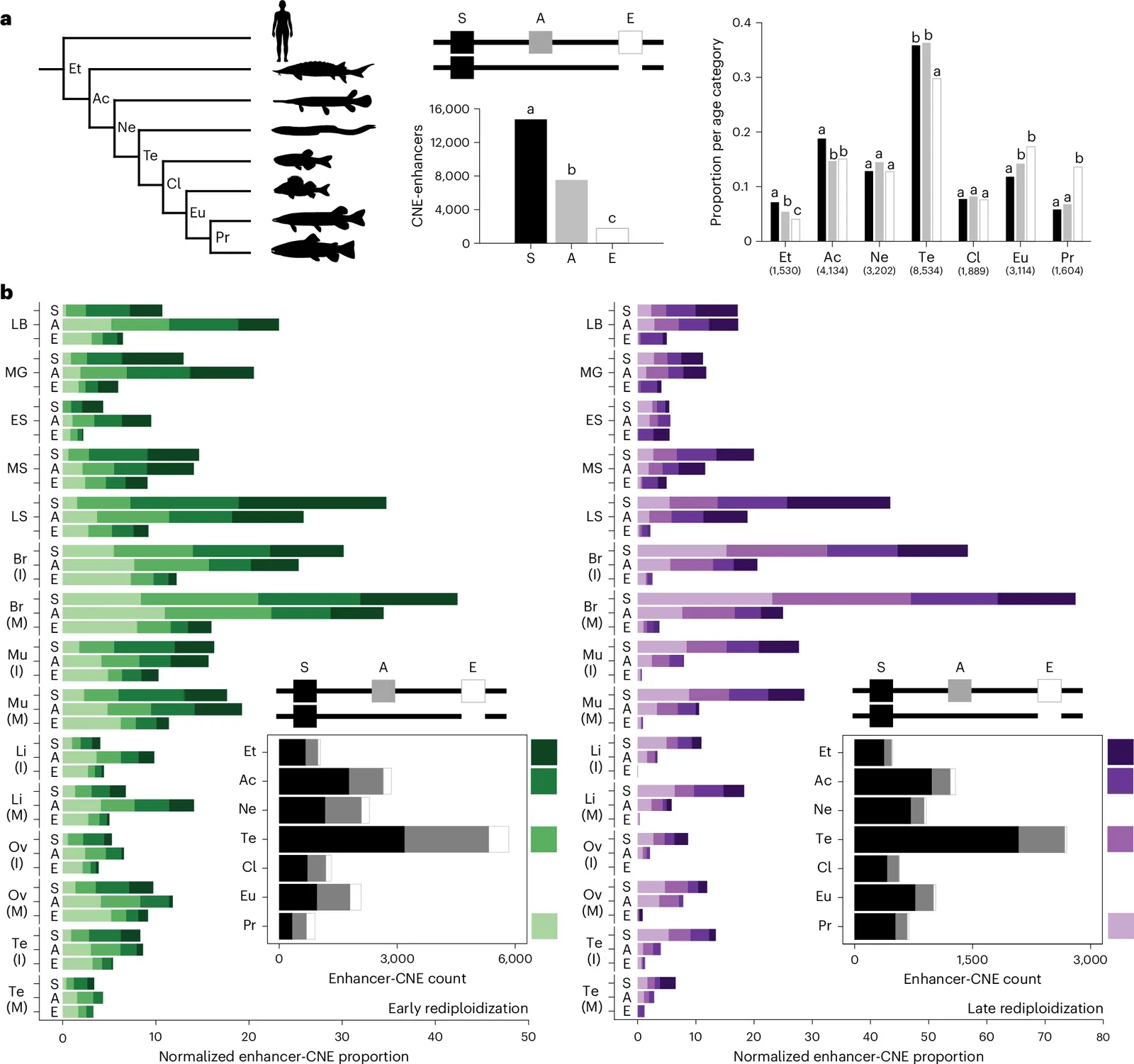
Evolution of enhancer-CNEs following the salmonid 4R autotetraploidization. **a**, Left: the tree illustrates the different phylogenetic ages of enhancer-CNEs: Et, Euteleostomi; Ac, Actinopteri; Ne, Neopterygii; Te, Teleostei; Cl, Clupeocephala; Eu, Euteleostomorpha; Pr, Protacanthopterygii. Also shown is the number of salmon enhancer-CNEs belonging to the shared (S) (*n* = 14,732), alignable only (A) (*n* = 7,507) and exclusive (E) (*n* = 1,768) categories, along with their proportions per phylogenetic age category. On the provided bar plots, different lowercase letters indicate significant differences between alignment categories within each provided comparison. Differences in enhancer-CNE proportions between alignment categories were assessed using two-sided Fisher’s exact tests with Benjamini–Hochberg false discovery rate correction (S versus A, odds ratio 0.51, 95% confidence interval 0.49–0.53, *P*_adj _< 2.2 × 10^−16^; A versus E, odds ratio 4.25, 95% confidence interval 4.00–4.51, *P*_adj _< 2.2 × 10^−16^; E versus S, odds ratio 8.33, 95% confidence interval 7.89–8.80, *P*_adj _< 2.2 × 10^−16^). **b**, Normalized proportions of salmon enhancer-CNEs for each alignment category (S, A and E), stratified by phylogenetic age and different sample types for early (left) and late (right) rediploidization regions. Sample abbreviations are as in Fig. 3. Silhouettes from PhyloPic (https://www.phylopic.org) under a Creative Commons license: *Acipenser oxyrhynchus, Anguilla anguklla*, *Perca* *fluviatilis*, *Esox* *lucius* (PDM 1.0); *Homo* *sapiens*, *Lepisosteus* *oculatus*, *Danio* *rerio*, *Salmo* *trutta* (CC0 1.0).

Conserved non-coding elements (CNEs) often lie within enhancers, marking regulatory sequences under purifying selection across species^60,61^. We overlapped all salmon active enhancers with a novel dataset of northern pike CNEs stratified by phylogenetic age, with the oldest category (Euteleostomi) predating the split of ray- and lobe-finned fish (Fig. 6 and Supplementary Table 17). We explored the post-WGD retention of salmon CNEs lying within active enhancers (hereafter: enhancer-CNEs) by alignment to pike CNEs (Supplementary Table 18). In this context, exclusive enhancer-CNEs are singletons where one in a duplicated pair was lost post-WGD, with the retained CNE conserved with pike. Among 24,007 enhancer-CNEs, 61.4%, 31.2% and 7.3% belonged to the shared, alignable only and exclusive categories, respectively (Fig. 6a).

Enhancer-CNEs from the shared category were enriched for the two oldest CNE categories (Euteleostomi/Actinopteri), whereas exclusive enhancer-CNEs were enriched among the youngest CNEs and underrepresented in the Teleostei category (Fig. 6a). Shared enhancer-CNE pairs showed higher sequence-level conservation than the alignable only category, both overall (median identity 95.0 versus 92.9%; Wilcoxon test, *P* = 2.2 × 10^−16^) and stratified by CNE age (Supplementary Fig. 36). For enhancer-CNEs from the alignable only category, the CNE that lost activity evolved more rapidly, with the youngest CNEs most affected (Supplementary Fig. 36). Thus, the phylogenetic age and functional retention of enhancer-CNEs reflects their ongoing evolution following WGD.

Enhancer-CNEs were strongly overrepresented in Late rediploidization regions, both overall and per age category (Supplementary Table 19). This was explained by shared enhancer-CNEs (Supplementary Fig. 37), whereas enhancer-CNEs from the alignable only and exclusive categories were enriched in early regions (Supplementary Fig. 37 and Supplementary Table 20). Shared enhancer-CNEs within late regions occupied 39.9% of all shared enhancer-CNE base pairs in the genome, twice that expected by chance (Supplementary Table 20), implicating selection against the divergence of these elements during the early stages of post-WGD evolution.

**Extended Data Fig. 8:**
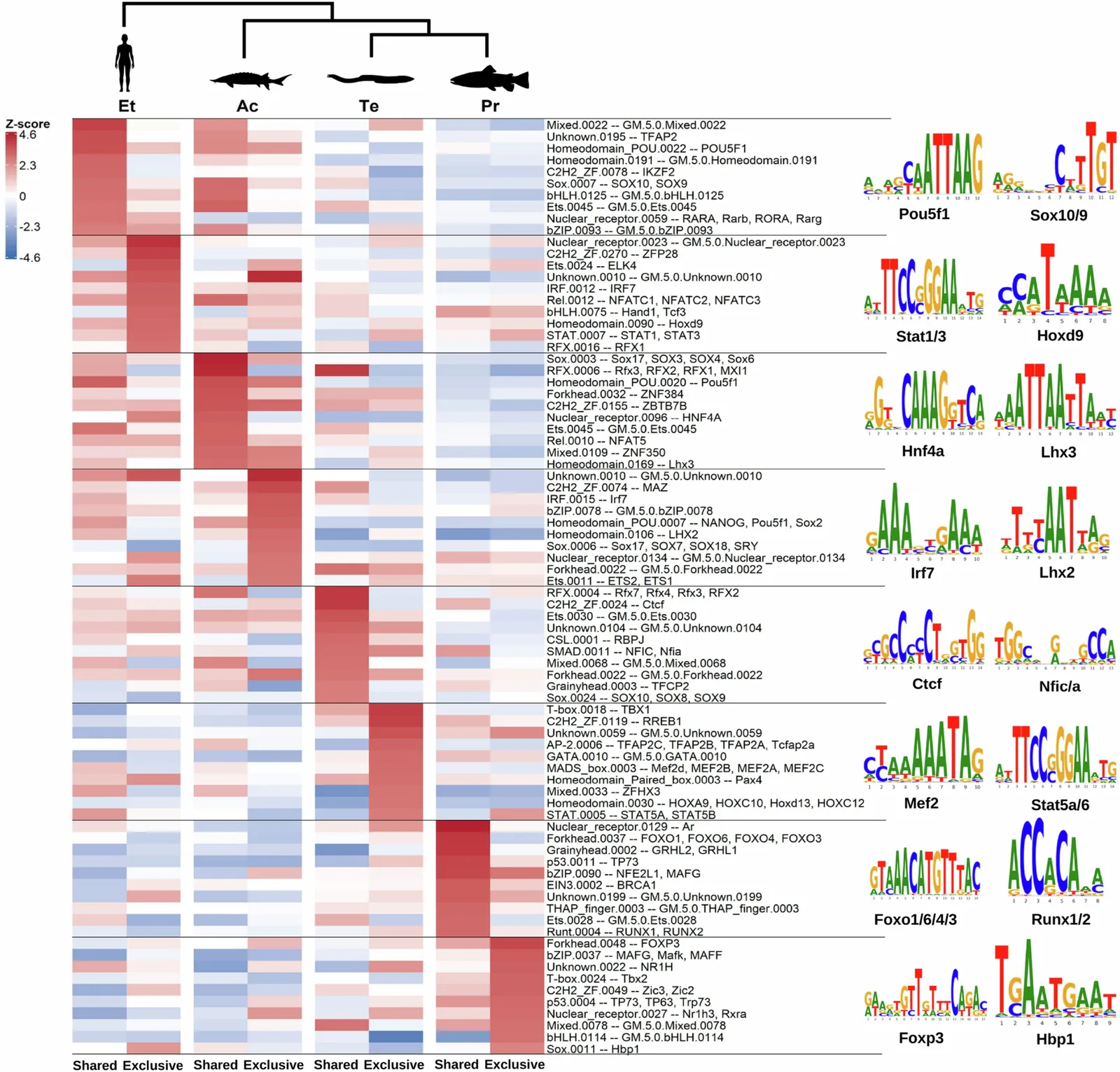
Differential enrichment of DNA motifs in enhancer-CNEs of distinct phylogenetic ages, contrasting functional retention or loss following the salmonid 4R WGD. The heatmap shows differential enrichment of motifs using Z-score values comparing WGD alignment categories (Shared vs. Exclusive; = conserved vs. lost across duplicated regions retained following WGD) for four phylogenetic ages (‘Et’ =Euteleostomi; ‘Ac’ =Actinopteri, ‘Te’ =Teleostei, and ‘Pr’ = Protacanthopterygii). The top ten uniquely enriched motifs (gimme.vertebrate.v5.0 motif database) per category are included, with motif names provided along with predicted TFs (following ‘-’ in each motif title). Example motif logos and their predicted TFs are shown in the right panel. Silhouettes from PhyloPic (https://www.phylopic.org) under a Creative Commons license: *Acipenser oxyrhynchus, Anguilla anguklla*, *Perca* *fluviatilis*, *Esox* *lucius* (PDM 1.0); *Homo* *sapiens*, *Lepisosteus* *oculatus*, *Danio* *rerio*, *Salmo* *trutta* (CC0 1.0).

Euteleostomi and Actinopteri Enhancer-CNEs were enriched in motifs for master developmental regulators of vertebrate cell-fate, pluripotency, embryonic patterning and organogenesis—particularly Homeodomain, Sox and related motifs. This was contrasted by enrichment of C2H2 zinc finger, bZIP and nuclear receptor motifs for regulators of stress and hormone responses, chromatin, metabolism, muscle development, immunity and detoxification responses in the younger CNE categories (Supplementary Fig. 38, Supplementary Table 2 and Supplementary Text 9). Across all phylogenetic ages, there were strong differences in motif enrichment between shared and exclusive enhancer-CNEs (Extended Data Fig. 8 and Supplementary Table 22). Euteleostomi and Actinopteri shared enhancer-CNEs showed several commonly enriched motifs (for example, POU5F1, Sox10/9 and TFAP2) depleted in exclusive enhancer-CNEs of the same age categories. However, Euteleostomi shared enhancer-CNEs were specifically enriched in motifs for Pax3/7, Ikzf2 and RAR family members (Extended Data Fig. 8).

Euteleostomi exclusive enhancer-CNEs were specifically enriched for master regulators of antiviral responses (Irf7), immune functions (Spic, Nfatc1 and Stat1/3) and embryonic or tissue development (Hand1/Tcf3, Zfp28, Elk4 and Etv5). For Teleostei enhancer-CNEs, motifs enriched in the shared category included Rfx and EGR family members, CTCF and CTCFL (Boris), whereas motifs for SRF, Stat5b, Pax4 and Mef2 were enriched among the exclusive teleost enhancer-CNEs (Extended Data Fig. 8 and Supplementary Table 22). Protacanthopterygi shared enhancer-CNEs were enriched in motifs for the glucocorticoid and androgen receptors, immune system TFs (for example Jun, FoxO1 and Ets) and TFs governing circadian rhythms (DBP, HLF and NFIL3), whereas the equivalent set of exclusive enhancer-CNEs was enriched in motifs for regulators of metabolism (for example THRB and NR1H3), tumour suppressors (Hbp1 and TP63/TP73) and regulatory T cell development (Foxp3).

We finally partitioned enhancer-CNEs across alignment categories, rediploidization regions, CNE ages and sample types (Fig. 6b; see Supplementary Tables 23–25 for statistical support). Shared enhancer-CNEs from the two oldest CNE categories were strongly enriched at late somitogenesis for both early and late regions (Fig. 6b and Supplementary Table 25; *P*_adj _*=* 0). The proportion of Euteleostomi Shared enhancer-CNEs at late somitogenesis was in fact higher than for brain (Fig. 6b and Supplementary Table 25; *P*_adj _*=* 0). Comparing adult tissues, brain showed the highest proportion of enhancer-CNEs, with a significantly increased proportion of shared enhancer-CNEs from the Teleostei and Protacanthopterygii age categories compared with embryonic stages and other adult tissues (Fig. 6b).

These findings further support the role of ontogeny in the evolution of genome regulation following the salmonid 4R WGD. The overrepresentation of ancient vertebrate CNEs among shared enhancer-CNEs active during late somitogenesis, enriched in motifs for master regulators of embryonic development, is consistent with these duplicated enhancers contributing to the limited ohnologue expression divergence observed at this stage. More generally, our data highlight distinct fates of vertebrate enhancer-CNEs following WGD, with strong biases across diverse biological functions governed by distinct TF motifs. In this respect, the identified exclusive enhancer-CNEs are candidates for explaining ohnologue regulatory subfunctionalization.

## Discussion

Ohnologue regulatory evolution following the salmonid 4R WGD was governed by developmental and tissue-specific constraints shown elsewhere to be acting across vertebrate species^41,44,46^. Multiple lines of evidence imply a period of high ‘within-genome’ post-WGD constraint on regulatory evolution at the phylotypic period, which may reflect the limited evolvability of pleiotropic regulatory networks governing vertebrate organogenesis^41,50^. Past work in salmon concluded that the evolution of regulatory elements following the 4R WGD was selectively neutral, on the basis of an analysis of a single histone mark in testis^57^. Our atlases revealed extensive variation in the rate of regulatory evolution across tissues, with brain representing the maxima of post-WGD constraint and gonads and liver experiencing rapid regulatory evolution, which may reflect either (or a combination of) relaxed purifying selection and positive selection. Our study also shows that WGD created opportunities for regulatory diversification, via the loss of duplicated enhancer-CNEs. Future studies should further dissect these findings using additional data types to penetrate regulation at the level of three-dimensional chromatin architecture and specific cell types^62^.

Recent work shows that asynchronous rediploidization followed several vertebrate WGD events^15,23,24^ additional to the salmonid 4R. Thus, the stratification of regulatory divergence identified in salmonid genomes probably occurred at earlier stages of vertebrate diversification, where WGD may have facilitated the evolution of lineage-defining traits such as brain complexity^63^. Salmonids provide an excellent system to explore the regulatory basis of such innovations, including lineage-specific traits arising following asynchronous rediploidization^17^.

Finally, this study shares an open genomic resource, which, alongside a tissue regulatory atlas published recently for trout^64^, can support precision breeding goals in salmonid aquaculture^28,29^ by helping to dissect traits with a regulatory basis. Our datasets will also empower studies exploring the role of regulatory elements in the adaptation of wild salmonids and enrich comparative analyses of regulatory evolution across vertebrates.

## Methods

### Ethical considerations

This research complies with relevant ethical regulations. Sampling of fish embryos (DevMap) was done before protected life stages. For tissue sampling in rainbow trout adults (BodyMap), the experiment was conducted at the INRAE Fish Physiology and Genomics Laboratory (LPGP) experimental facilities (IE LPGP, permit number C35-238-6, Rennes, France) and approved by the animal welfare committee for the animal experimentation of LPGP under the authorization number T-2020-16-YG-AL. Tissue sampling in Atlantic salmon adults (BodyMap) was conducted at the Center for Fish Trials (NMBU, Ås, Norway), an approved facility that is registered with the Norwegian Food Safety Authority (Mattilsynet) under locality number 10362. Animal treatment and experiments were conducted according to institutional and national animal guidelines, as well as laws that regulate the scientific use of experimental animals in Norway (EU directive 2010/63/EU, FOR-2017-04-05-451).

### Animal material

Detailed per-sample information describing, as applicable, the strain, age, life-stage and source of animal material, was shared as metadata through the FAANG data portal (https://data.faang.org/projects/AQUA-FAANG). This information can be navigated per dataset via the ENA dataset accessions provided in the ‘Data availability’ statement.

In brief, for DevMap, the sampled salmon embryos were derived from a cross between a single male and female, with the milt and eggs collected in November 2019, provided by AquaGen (Holywood Salmon Farm, Dumfries, Scotland), representing a commercial aquaculture strain. The sampled trout embryos were also derived from a cross between a single male and female, with the milt and eggs collected in February 2020, provided by Northern trout (North Yorkshire, England), representing an anadromous aquaculture strain. For BodyMap, the sampled salmon were sourced from AquaGen, representing a commercial aquaculture strain, aged either 9 months (sexually immature) or 16 months (sexually mature). The sampled trout were sourced from INRAE’s experimental facilities (PEIMA, INRAE, Fish Farming systems Experimental Facility, https://doi.org/10.15454/1.5572329612068406E12, Sizun, France), representing the INRAE autumn strain, aged either 13 months (sexually immature) or 19 months (sexually mature).

### Protocols and sample information

In sections that follow, we overview key steps of our study, including sampling, sequencing library preparation and sequencing for both salmonid species. These descriptions are based on full protocols provided in the Supplementary Text (Supplementary Texts 1–4). Information about sample types, biological replicates and sequencing assays can be found in Supplementary Tables 1 and 2, with a visual summary in Supplementary Fig. 1.

### Sampling

#### DevMap sampling

A full sampling protocol is provided in the Supplementary Text (Supplementary Text 1). In brief, as described above, eggs from a single salmon or trout female were fertilized with milt from a single salmon or trout male, respectively, leading to monoparental batches of fertilized eggs. Fertilized eggs were incubated at 8 °C (salmon) or 9 °C (trout) in tray systems receiving recirculating, filtered water. At each sampling timepoint, eggs were collected then processed and stored for subsequent RNA extraction and library preparation for RNA-seq, ATAC-seq and ChIP-seq (see later sections).

For RNA extraction, three eggs were punctured and submerged in 1,000 µl of TRIzol (Thermo Fisher) in 1.5-ml Safe-Lock Eppendorf tubes with homogenization beads (27 tubes sampled per developmental stage). This was followed by homogenization using a TissueLyser II (Sigma) for 2 min at 30 Hz and storage at −80 °C. For ATAC-seq library preparation, eggs were dechorionated and the embryo separated from the yolk sac using watchmaker forceps. After gentle mincing with a scalpel, the embryo material was digested using 0.25% Trypsin EDTA. Following PBS washes, the embryos were cryopreserved in 300 µl cryopreservation solution (10% dimethylsulfoxide (DMSO), 40% FBS, 50% DMEM) using 2-ml tubes and a Mr Frosty freezing container (Nalgene) with a 1 °C min^−1^ cooling rate at −80 °C. A total of *n* = 3 biological replicates were sampled per developmental stage. The number of embryos required per biological replicate was as follows: blastula stages, 40; gastrula stages, 30; early somitogenesis, 20; mid-to-late somitogenesis, 10; and eyed stage/pharyngula, 5. The volume of 0.25% Trypsin EDTA and duration of trypsinization also varied for each stage, as described within Supplementary Text 1. Embryo material was prepared for ChIP-seq as described above for ATAC-seq before the digestion step, followed by crosslinking using 875 µl of 1% formaldehyde. Crosslinking was stopped using 125 µl of 1 M glycine, followed by resuspension in 1 ml of PBS and storage at −80 °C; *n* = 3 replicates were sampled for each developmental stage. The number of embryos used per ChIP-seq replicate varied by developmental stage as for ATAC-seq.

#### BodyMap sampling

A full sampling protocol is provided in Supplementary Text 1. In total, *n* = 12 salmon and *n* = 12 trout were used. In both cases, this included *n* = 3 fish per sex (that is, male and female) for two stages of ontogeny (sexually immature and sexually mature). The maturation status of all the sampled fish was established by visual inspection of the gonads, followed by histological analysis (images provided within Supplementary Text 1). Fish were fasted for 24 h before a lethal/non-recovery dose of MS222 was administered, followed by killing using a schedule 1 method. Seven tissues were immediately sampled (in order: brain, liver, gonads, distal intestine, gill and muscle), cut in pieces, washed in cold PBS then placed in RNAlater solution at 4 °C overnight (RNA-seq) or snap-frozen on dry ice (ATAC-seq and ChIP-seq) and then stored at −80 °C. The brain was collected following removal of the pituitary gland and brain stem, with remaining regions thoroughly mixed before splitting each sample. A strip from the middle of the liver was extracted, avoiding the vascular network. A slice (maximum 1-cm thick) from the middle of the gonad was placed in Dietrich solution for 10 days and then dehydrated with ethanol washes, embedded in paraffin and cut into 4-μm sections using a microtome. Eosin staining was used to assess gonadal maturation state (images provided within Supplementary Text 1). The distal intestine samples were collected after gentle removal of faecal remains and mucus. The head kidney tissue was carefully removed, washed in PBS and cut into slices for sampling. Gill samples were restricted to the filaments, with rakers discarded. Fast-twitch (‘white’) skeletal muscle was sampled from the anterior epaxial myotomal muscle adjacent to the dorsal fin.

The number of biological replicates used from the above samples for RNA-seq, ATAC-seq and ChIP-seq is provided per species, sex and maturation status in Supplementary Table 1.

### Functional assays and sequencing

#### Total RNA extraction, DevMap

A full protocol is provided within Supplementary Text 2. In brief, total RNA extraction was performed after thawing the homogenized embryo samples (in TRIzol; see above) at room temperature, followed by centrifugation to separate the aqueous phase from the organic phase, debris and oils. Phase separation was achieved by adding 200 µl of 1-bromo-3-chloropropane, followed by vortexing for 15 s and incubation at room temperature for 30 min. After centrifugation, 400 µl of the aqueous layer was extracted. RNA precipitation was achieved by the addition of 200 µl of a 1:1 solution of isopropanol:RNA precipitation solution (1.2 M sodium chloride, 0.8 M potassium citrate sesquihydrate). Following overnight precipitation at −20 °C, samples were centrifuged and pellets washed in 70% ethanol under rotation for 30 min. Three such ethanol washes were performed. Pellets were resuspended in 20–200 µl of nuclease-free water (volume used depending on pellet size) at room temperature for 60 min, flicking the tubes every 10 min. RNA concentration was established using a Qubit 4 fluorimeter (Invitrogen) with a Broad Range RNA kit. RNA purity was confirmed with a Nanodrop 1000 spectrometer (Thermo Fisher Scientific). RNA integrity was confirmed using a Tapestation 4200 (Agilent), recording RNA integrity (RIN) scores for all samples.

#### Total RNA extraction, BodyMap

A full protocol is provided within Supplementary Text 2. In brief, total RNA extraction from tissues of salmon was performed using a column-based miRNeasy Mini kit (Qiagen, no. 217004) following the manufacturer’s instructions. Each tissue was removed from RNAlater solution, placed in 700 µl Qiazol with one 5-mm stainless steel bead (Qiagen, no. 69989), homogenized with a TissueLyser (Qiagen) for 15–40 s (until the solution appeared clear), then mixed with chloroform and centrifuged at 12,000*g* for 15 min. The upper phase was extracted and mixed with 100% ethanol, passed through the RNeasy filter included in the kit, then washed, DNAse-digested (Qiagen, no. 79254) and washed again. An optional column drying (17,000*g*, 1 min) after the washing steps was applied to improve RNA purity. Total RNA samples were eluted in 50 µl water and their quality assessed using a Nanodrop 1000 spectrometer and Bioanalyzer 2100 system (Agilent), before storage at −80 °C.

Total RNA extraction from tissues of trout was performed using a phenol–chloroform extraction method. Tissue samples (100 mg) were submerged in 1 ml Trizol (Euromedex, no. TR118) and homogenized (Precellys, Bertin Instruments, no. P000669-PR240-A) in Eppendorf tubes for 40 s with ceramic beads (1.4 mm, no. P000933-LYSK0-A.0 OZYME). A total of 200 μL of chloroform was added before the tubes were inverted several times and the mixed samples incubated for 5 min at room temperature before centrifugation (12,000*g* at 4°C for 30 min). The upper phase was transferred to a new tube, mixed with isopropanol (500 μl per 1 ml Trizol) by vortexing, incubated for 2 h at −20 °C, before centrifugation at 12,000 g (4°C for 25 min). The resultant pellet was carefully washed twice with 1 ml of 75% ethanol. The washed pellet was dried and resuspended in ultra-pure water before quality assessment, as for salmon above.

### Library preparation and sequencing

#### RNA-seq, DevMap and BodyMap

RNA-seq library preparations were performed by Novogene using total RNA samples described above from both species (Supplementary Tables 1 and 2). In all cases, the NEBNext Ultra Directional RNA Library Prep for Illumina kit (no. E7420S/L) was used with the NEBNext Poly(A) mRNA Magnetic Isolation Module (no. E7490) and NEBNext Multiplex Oligos for Illumina (no. 7600). All libraries were produced following manufacturer’s instructions. In brief, mRNA was isolated from 0.1–1 µg of total RNA using the poly(A) mRNA enrichment step, fragmented and random primed before first and second strand cDNA synthesis step. Next, 3′ ends were end-repaired and dA-tailed, ligated to the adapter and size selected, PCR-amplified and cleaned-up. Libraries were sequenced on the Illumina Novaseq 6000 platform generating paired-end 150-bp reads.

#### ATAC-seq, BodyMap

A full protocol including tissue-specific modifications is provided within Supplementary Text 3. In brief, ATAC-seq was based on Omni-ATAC-seq^65^, a modified version of ATAC-seq^32,66^ optimized for frozen tissues. Omni-ATAC-seq is divided into three main steps: nuclear isolation, transposition and PCR with size selection. Nuclear isolation involves tissue disruption, filtration and homogenization, release of nuclei from cells and then purification and concentration of nuclei suspension. Optimizations of nuclear preparations were applied to different tissues (Supplementary Text 3). For example, in the case of mature testis, additional washes and a second gradient centrifugation improved the ratio of nuclei from testis versus contaminating sperm cells. Alternatively, for muscle, tissue disruption required grinding on dry ice to effectively disrupt the muscle fibres and release the nuclei^67^.

For each processed sample, nuclei were counted and confirmed for quality before transposition, done using Illumina Tagment DNA Enzyme (transposase) and Buffer (Illumina, no. 20034197). The transposition reaction was sensitive to the ratio of transposase to nuclei. We found the transposition to be optimal using 50,000 nuclei per 2.5 µl transposase, except for gill (25,000 nuclei per 2.5 µl transposase) (Supplementary Text 3). Following 30-min transposition at 37 °C, the DNA was purified using a MinElute PCR Purification Kit (Qiagen, no. 28004) and frozen at −20 °C. The above steps were performed in tissue-specific batches; then, once a larger number of eluted DNA was accumulated, the samples were PCR-amplified (8–13 total PCR cycles) and size-selected (180–700 bp). The number of PCR cycles and size selection steps were optimized to fit the best product size distribution for sequencing. The DNA profile (nucleosome banding) was confirmed using a High Sensitivity Bioanalyzer (Agilent). The concentration of the libraries was assessed by using High Sensitivity Qubit 4.0 kit. Libraries were sequenced on the Illumina Novaseq 6000 platform by Novogene.

#### ATAC-seq, DevMap

A full protocol is provided within Supplementary Text 3. In brief, the ATAC-seq method used with embryo samples was an adaptation of the BodyMap Omni-ATAC method, differing in the steps leading up to nuclear isolation, which were based on a published protocol used for single nuclei RNA-seq^68^. Cryopreserved embryo samples (see above section) were thawed on ice by pipetting up and down in 900 µl of ice-cold PBS containing Protease Inhibitor Cocktail (PIC, Sigma) This step was immediately followed by a round of centrifugation and resuspension of the resultant pellet in 1,000 µl of PBS containing PIC. After a further round of centrifugation, nuclei isolation was performed by resuspending the pellet in 1,000 µl of Tween with salts and Tris buffer containing PIC, followed by pipetting up and down on ice for 5 min. The nuclei isolation was stopped by the addition of salts and Tris buffer and centrifuged (Supplementary Text 3). A total of 75,000 nuclei were for the transposition reaction, following the Omni-ATAC-seq protocol described for BodyMaps above. Libraries were sequenced on the Illumina Novaseq 6000 platform by Novogene.

#### ChIP-seq, DevMap

A full protocol is provided within Supplementary Text 4. In brief, we used a ChIPmentation approach with all DevMap samples. ChIPmentation is a tagmentation-based ChIP-seq method that allows one-step library preparation on bead-bound chromatin^69^. We used a μChIPmentation Kit for Histones (Diagenode no. C01011011), optimized for low input samples, such as those from embryos. In brief, crosslinked embryo samples were thawed on ice and washed once with ice-cold PBS before homogenization in 1 ml of homogenization buffer (PBS and 1× PIC) using a 2-ml Dounce homogenizer (20–60 cycles on ice).

We used Chromatin EasyShear Kit—High SDS (Diagenode no. C01020012) for cell lysis and chromatin shearing. In total, 5 × 10^5^ homogenized cells were lysed in 315 μl of tL1 lysis buffer with 1× PIC on ice for 20–30 min. After complete lysis was observed by microscope, 185 μl of Hanks’ Balanced Salt Solution buffer was added, and samples were sonicated using a Bioruptor Pico bath sonicator. Samples were sonicated in 0.65 ml Bioruptor Microtubes no. C30010011 for 3–7 cycles (1 cycle was 30 s ‘on’ followed by 30 s ‘off’) until chromatin fragments, as analysed using a Tapestation 4200 (High-Sensitivity D1000 assays), were in the 200–800-bp size range.

We followed Diagenode’s instructions for μChIPmentation with the following modifications: (1) pull-down was done using 100 ng of sheared chromatin per assay (equivalent to approximately 37,000 cells), and (2) 0.8–1 μg antibody was used (H3K27ac no. C15410196, H3K4me1 no. C15410194, H3K4me3 no. C15410003 and H3K27me3 no. C15410195) per reaction. For library preparation, we used 24 unique dual indexes for tagmented libraries Set I and Set II from Diagenode (no. C0101134 and no. C0101135); samples were tagmented at 37 °C for 12 min and amplified for 14 to 15 PCR cycles. Libraries were sequenced on an Illumina Novaseq 6000 by Novogene, generating 150-bp paired-end reads (targeting 45 million reads per ChIP sample,). For each condition, a fraction of chromatin was extracted before immunoprecipitation for control (6 μl) and underwent the μChIPmentation protocol in a similar way as for the samples, with several adaptations (Supplementary Text 4). An equimolar mix of input controls per developmental stage was created for sequencing (90 M reads per input control sample).

#### ChIP-seq, BodyMap

Full protocols including tissue-specific modifications are provided within Supplementary Text 4. The Diagenode μChIPmentation approach, as used for DevMap, was used for brain tissue in trout. For all other tissues from both species, ChIP-seq was performed using frozen tissues, with an optimized classic protocol involving seven steps: tissue disruption and homogenization, crosslinking, sonication, immunoprecipitation, washing, de-crosslinking and elution of immunoprecipitated DNA. The first step aimed to produce a homogeneous and clean isolation of nuclei, which required additional modifications for muscle and mature male gonads. Counting of nuclei was performed before crosslinking. After crosslinking, multiple washes were immediately performed to clean the nuclei. Next, samples were concentrated to 10 million nuclei ml^−1^ (400 μl total volume) and sonicated with a probe sonicator (EpiShear, ActiveMotif, no. 53052), with all parameters fixed (55% intensity, 1-s ON/1-s OFF cycle rhythm, 30-s cycle) except the number of cycles, which was tissue-specific. After sonication, the sample was centrifuged for 10 min at 8,000*g* to separate sonicated chromatin from debris. To assess the profile of sonicated chromatin using an Agilent Bioanalyser, an aliquot of each sample was de-crosslinked and the DNA purified and quantified. Samples with a size distribution of 100–600 bp (median 350 bp) were diluted fourfold with immunoprecipitation buffer before storage at −80 °C before immunoprecipitation.

Immediately before immunoprecipitation, two separate preparation steps were performed in parallel: (1) chromatin preclearing (30-min incubation of chromatin with Protein A/G beads without antibody) and (2) coupling of antibodies to the Protein A/G beads, which was subsequently used in an overnight IP step (4 °C, in rotation) with precleared chromatin. An aliquot (5 ng) of precleared chromatin from each sample was not immunoprecipitated and used as an input control (each control library was produced using a pool of group-specific input samples). Four antibodies (Diagenode) were used with following amounts per immunoprecipitation: 1 μg for H3K4me1 (no. C15410194) and H3K4me3 (no. C15410003) and 2 μg for H2K27ac (no. C15410196) and H3K27me3 (no. C15410195). Next, immunoprecipitated samples were washed multiple times with graded salinity buffers, allowing the removal of weak bonds between Protein A/G beads–antibody complexes and unspecific/nontargeted protein epitopes, before the immunoprecipitated chromatin was eluted from the beads and de-crosslinked, its DNA purified using a MinElute PCR Purification Kit (Qiagen, no. 28004) and its concentration assessed using a Qubit High Sensitivity DNA Kit (Thermofisher, no. Q32854).

Immunoprecipitated DNA was stored at −20 °C until library preparation, done using a MicroPlex Library Preparation Kit V3 (Diagenode; no. C05010002), following the manufacturer’s recommendations. In brief, a minimum of 0.2 ng of immunoprecipitated DNA was end-repaired and ligated with stem-loop adaptors through blunt-end ligation, then dual-indexed (96 dual indexes for MicroPlex Kit v3 Set I-IV, no. C05010004- C05010007) and PCR-amplified (from 7 to 8 cycles for inputs and from 8 to 13 cycles for immunoprecipitated products), purified (Agencourt AMPure XP Beads, Beckman Coulter, no. A63880) and sequenced on an Illumina Novaseq 6000 platform by Novogene, generating 150-bp paired-end reads (targeting 45 million reads per ChIP sample and 90 million reads per input control sample).

### Primary data analyses

#### Reference genomes

The reference genomes and annotations used for all reported data analyses were downloaded from the Ensembl rapid release database: for salmon: assembly Ssal_v3.1, softmasked fasta file (Salmo_salar-GCA_905237065.2-softmasked.fa) and gene annotation GTF file (Salmo_salar-GCA_905237065.2-2021_07-genes.gtf.gz), and for trout: assembly USDA_OmykA_1.1, softmasked fasta file (Oncorhynchus_mykiss-GCA_013265735.3-softmasked.fa) and gene annotation GTF file (Oncorhynchus_mykiss-GCA_013265735.3-2020_12-genes.gtf).

#### Bioinformatic code

All code described in sections that follow is available through a project GitHub page^70^.

#### RNA-seq alignment and quantification

RNA-seq data was processed using a standardized approach through nf-core pipeline^71,72^ rnaseq v3.8.1^73^ with standard parameters. In brief, the pipeline involves adapter and quality trimming of reads (Trim Galore!)^74^, alignment of reads to the reference genome using STAR^75^ before projection of alignments to the transcriptome for BAM-level quantification of gene and transcript level read counts using RSEM^76^.

#### ATAC-seq alignment and peak calling

ATAC-seq data was processed using a standardized approach through nf-core pipeline atacseq v1.2.1^77^ with standard parameters. In brief, the pipeline involves adapter and quality trimming of reads (Trim Galore!), alignment of reads to the reference genome using the Burrows-Wheeler Alignment (BWA) tool^78^ and (narrow) peak calling using MACS2^79,80^. The estimated genome size used was: 2,756,563,003 for salmon and 2,070,645,207 for trout.

#### ChIP-seq alignment and peak calling

All ChIP-seq data was processed using a standardised approach through the nf-core pipeline chipseq v1.2.2 (ref. ^81^) with standard parameters. In brief, the pipeline involves adapter and quality trimming of reads (Trim Galore!), alignment of reads to the reference genome using BWA and peak calling using MACS2 with representative ChIP input control samples used to establish background signal. The estimated genome size used was as detailed above for ATAC-seq. Broad peaks were called for H3K4me1 and H3K27me3 and narrow peaks for H3K4me3 and H3K27ac.

#### Quality control results for each functional assay

MultiQC^82^ reports for all RNA-seq, ATAC-seq and ChIP-seq samples are shared in Supplementary Data 1.

#### Blacklist regions

To remove signal–artefact regions from the sequencing data for downstream analysis, we generated blacklist files for the salmon and trout genomes. First, uint8 mappability files were created for 75, 100 and 150 k-mers across each chromosome in the genome using Umap^83^, and then, all k-mer mappability results were combined. Finally, blacklist regions were defined using Blacklist^84^ taking in all available ChIP-seq input BAM files (32 and 29 respective input controls for salmon and trout) and combined uint8 files. Blacklists were generated separately for classic ChIP (BodyMap) and μChIPmentation (DevMap) input controls, with the resulting blacklisted regions merged.

#### Chromatin state annotations

Chromatin states were annotated across the salmon and trout genomes using ChromHMM input signal data were BAM alignments for the ATAC-seq and ChIP-seq (H3K27ac, H3K4me1, H3K4me3 and H3K27me3 marks) data generated as described above. Sample-matched input controls were given to estimate background signals for the ChIP-seq data. Before ChromHMM, BAM alignments were converted to BED format and filtered to keep reads only in chromosomes and exclude blacklist regions. The alignments were binarized using the ChromHMM BinarizeBed function with default bin size (200 bp), before running the ChromHMM LearnModel function. After testing models with various state numbers, with the aim to capture the most biologically relevant chromatin states^36^ we proceeded with: two separate 12-state models for salmon DevMaps (that is, embryo stages) and BodyMaps (that is, tissue samples, brain, gonad, liver and muscle), and for trout, a 12-state model for DevMaps and three separate 10-state models for each included tissue (brain, liver and muscle) (Extended Data Fig. 1). Note that each trout tissue was treated separately, as data quality was more variable between tissues compared with salmon. After generating the ChromHMM models, the non-blacklisted BAM files representing ATAC-seq and multiple histone mark ChIP-seq data were binarized using the ChromHMM BinarizeBam function with default bin size (200 bp). The combination of binarized signals from ATAC-seq and ChIP-seq marks were used together with the ChromHMM model for the respective species and condition to annotate regions across the genome representing different chromatin states at 200-bp resolution (Results).

#### Robust open chromatin definition and functional annotation

A set of robust open chromatin regions, defined as those that are highly reproducible across biological replicates per condition, was generated using the ATAC-seq narrow peak files resulting from the analyses described above, post-filtering to remove peaks either not found on chromosome sequences or completely overlapping blacklist regions. The reproducibility of peaks between all possible pairs of biological replicates per condition was determined using the IDR approach^35^ as done by the ENCODE project. IDR measures the reproducibility of the scoring of peaks between replicates to determine an optimal cutoff for peak significance. IDR was run using options -rank = ‘p.value’ and -soft-idr-threshold = 0.1. This gave a set of significantly reproducible peaks between pairs of replicates (Supplementary Table 4). To deal with having three biological replicates per condition, the resulting reproducible peaks from each pair were merged together with bedtools merge (≥1-bp overlap), taking the maximum IDR score for the resulting peak. With one set of reproducible peaks per condition, the summits of those peaks were recalculated using the MACS2 refinePeak function and all replicate ATAC-seq BAM alignments were merged together.

Finally, the reproducible ATAC-seq peaks with summits were assigned with chromatin states per condition by intersecting ChromHMM annotations using bedtools intersect. When a peak overlapped more than one ChromHMM annotation in a given condition, the assigned chromatin state was chosen in order of importance, on the basis of how confidently we could call the state given the combination of assay signals required. This order is the same order of the chromatin states shown in Fig. 1b (Results) from top to bottom. A single unified set of robust open chromatin regions (reproducible ATAC-seq peaks) was generated per species by merging all overlapping peak regions (≥1-bp overlap) across all conditions. Read counts from each ATAC-seq and ChIP-seq assays were counted with featureCounts using the BAM alignments for each biological replicate against the unified peak set of each species. This table of assay counts within robust open chromatin peaks for all samples was used for downstream analyses.

### TFBS enrichment analysis

TF binding site (TFBS) enrichment analysis was carried out with GIMME Maelstrom (GimmeMotifs v0.17.1^85^) separately on the identified active enhancer and active promoter regions of salmon and trout. The input was log-transformed ATAC-seq counts (pooled across biological replicates) for robust open chromatin peaks described above. The analyses were run with default parameters, except with the region size set to 200 bp. The motif database used was JASPAR2022_vertebrates.pfm. By investigating the results, we found that several closely related Jaspar TFBS were associated with different, but closely related TFBS showing an enrichment in matched sample types for salmon and trout. To reconcile this discrepancy for the purpose of comparative analysis, we downloaded a table including pairwise correlation values for all Vertebrate JASPAR TFBS (https://jaspar2020.genereg.net/static/clustering/2020/vertebrates/CORE/interactive_trees/JASPAR_2020_matrix_clustering_vertebrates_tables/pairwise_compa_matrix_descriptions.tab). TFBS that shared a correlation value ≥0.9 were considered a single TFBS for the purpose of comparison between salmon and trout, which led to an increase in the number of shared enriched TFBS.

### Homology prediction

Ensembl Compara^86^ 106 gene trees (downloaded from http://ftp.ensembl.org/pub/misc/aqua-faang/ensembl_trees/gene_trees/Compara.106.protein_default.nhx.emf.gz) were used as the basis to identify: (1) ohnologue pairs retained from the salmonid-specific WGD and (2) orthologues conserved between salmon and trout and with northern pike (*Esox lucius*) as an outgroup species to the WGD. Estimation of homology relationships is obscured by incorrect gene tree estimation, gene loss and lineage-specific rediploidization, where ohnologues independently diverged in sequence after the two salmonid species diverged^17^. To validate that duplicated genes identified in the Ensembl gene trees derived from the salmonid-specific WGD, we leveraged large collinear, syntenic blocks in which the vast majority of these genes reside^21^. Our approach first extracts Protacanthopterygii clades from the gene trees, that is including both salmonids and northern pike, a representative of Esociformes, the sister lineage to salmonids, which did not experience the salmonid-specific WGD^18^. If both ohnologues have been retained in salmon and trout, each clade will contain two genes per species, which we call ‘2:2 clades’. Using the 2:2 clades, we identified syntenic blocks by selecting the most commonly observed combinations of four chromosomes (>20 clades with the same combination of chromosomes) (Supplementary Fig. 15). We assign the orthologue relationship of the four chromosomes using the most commonly observed phylogeny of clades in agreement with ancestral ohnologue resolution (AORe)^17^ that is requiring two salmonid sister clades, each containing one salmon and one trout orthologue. In the main text, we also call these early rediploidization regions. In genomic regions that have undergone lineage-specific rediploidization, we observe two clades showing lineage-specific ohnologue resolution (LORe)^17^ that is where ohnologue pairs from each species are monophyletic, because they diverged after speciation. In the main text, we also call these late rediploidization regions. The syntenic relationship of 2:2 clades was used to verify the synteny of gene tree clades where two ohnologues were not retained in both species, including (among others): 1:1 clades (for example, resulting from one ohnologue in a pair being lost ancestrally to salmon and trout), 2:1 clades (where one ohnologue was lost in one species, and the other species retains both ohnologues) and 2:0 clades (for example, when both ohnologues were lost in one species, and the other species retains both ohnologues). The number of genes belonging to all possible type of clade is shown in Supplementary Fig. 15, and the full homology prediction is shared in Supplementary Table 6 and available on SalmoBase.org^34^.

### Whole genome alignment to explore regulatory element evolution

Whole genome alignments including salmon, trout and northern pike were generated using progressive Cactus^39^. As Cactus was not designed to align genomes affected by recent WGD events, we were concerned that running Cactus without adaptations using salmonid genomes would fail to effectively capture duplicated regions. As part of the European AQUA-FAANG project, a Cactus alignment was produced for six teleost species: salmon, trout, along with turbot, European seabass, Gilthead seabream and common carp (available at http://ftp.ensembl.org/pub/misc/aqua-faang/cactus/). This six-species alignment was run in default mode, where the whole genome of each species was fed into Cactus. The inspection of the results revealed suboptimal capture of duplicated regions, particularly in more divergent early rediploidization (AORe) regions (Supplementary Fig. 14), which comprise much of salmonid genomes^17^.

To confirm the suboptimal capture of duplicated regions, we devised then tested an alternative strategy where collinear, syntenic regions retained from the salmonid WGD were extracted as separate regions for salmon, trout (that is, two ohnologous collinear regions from each species) and northern pike as an outgroup. Genome-wide collinear and syntenic blocks for the salmon, trout and northern pike genomes were identified by aligning the chromosomes of each species to the salmon chromosomes using minimap2 (v 2.18-r1015)^87^. A manual inspection of dot plots from the alignments facilitated the identification of conserved collinear blocks within the duplicated salmon and trout genomes and enabled the determination of their orthologous regions in the northern pike genome. A final table, containing details of all the syntenic or collinear blocks with respect to each salmon chromosome, is presented in Supplementary Table 7. As an initial test system, we extracted two collinear regions associated with salmon chromosome 3, namely Ssa03 and Ssa06 (homologous to regions of Omy12 and Omy13) and Ssa03 and Ssa14 (homologous to regions of Omy28 and Omy08) using the synteny data generated above. We deliberately selected these regions as they represent early rediploidization (Ssa03-14 and Omy28-08) and late rediploidization (Ssa03-06 and Omy12-13) regions of similar sizes^21^, meaning they are markedly more and less diverged as duplicated sequences, respectively^21^. For each syntenic block, the extracted sequences were fed into Cactus as independent sequences, regardless of duplicated sequences originating within each species. Cactus was run with default parameters to generate a HAL alignment output for each syntenic block. The HAL output files for both syntenic blocks (Ssa03-Ssa06 and Ssa03-Ssa14) were converted to MAF alignment format using the hal2maf command (-maxRefGap 20000 -noAncestors -noDupes and rest as default) in the ComparativeGenomicsToolkit^88^, facilitating downstream processing. Salmon chromosome 3 (Ssa03) was set as the reference chromosome for both tested syntenic blocks during the HAL to MAF conversion. The mafStats command within mafTools^89^ was used to generate summary statistics for each MAF file, including the number of base pairs for each non-reference sequence (duplicated sequences) captured in the MAF alignment (Supplementary Fig. 16). We found that the capture of duplicated regions in both species was markedly improved by alignment of the extracted syntenic regions compared with the standard Cactus approach (Supplementary Fig. 16). The gain in recovered sequence was especially striking for the more divergent AORe (early rediploidization) regions and more modest, yet still substantial, for highly similar LORe (late rediploidization) regions (Supplementary Fig. 14).

For our final Cactus alignment strategy, as described above, we segmented the salmon and trout genomes into collinear regions retained from the salmonid WGD and identified orthologous regions in the northern pike genome (Supplementary Table 7). The resulting sequence regions were used for independent Cactus alignments for each salmon chromosome. A custom workflow was developed to automate this process. In brief, the pipeline uses each salmon chromosome as a reference, denoted by the prefix Ssal_A. The duplicated salmon chromosome is assigned the prefix Ssal_B, whereas syntenic trout sequences are labelled Omyk_A and Omyk_B, and the orthologous northern pike sequence is denoted as Eluc. For each reference chromosome (Ssal_A), the syntenic (orthologous) regions are extracted (extractBlocks.R) and aligned in Cactus (run_cactus.job.sh) with the assigned prefixes. The HAL output files were converted to MAF format using the hal2maf command in the Comparative Genomics Toolkit^90^. A custom Python script (fix_maf.py) was used to adjust alignment coordinates from block-level to genome-wide coordinates, producing a final set of MAF alignment files, one per salmon chromosome.

### SOM and UMAP

To determine how chromatin accessibility and gene expression were regulated across ontogeny, we clustered the salmon and trout ATAC-seq and RNA-seq datasets using SOM. For the ATAC-seq data, we first extracted raw read counts for all robust open chromatin regions. To account for differences in sequencing depth, tags per million values were calculated by normalizing read counts to the total number of mapped reads per sample while correcting for region length. To ensure comparability between samples, we calculated the fold change of observed over expected values for each open chromatin region, using the mean tags per million value within the sample as the expected value. The resulting fold change values were then scaled by dividing by the standard error across samples, ensuring comparability of both regions and conditions. For the RNA-seq data, we calculated the mean of biological replicates of transcripts per million (TPM) values for all samples. We independently performed quantile normalization across DevMap and BodyMap samples using the preprocessCore R package^91^ to correct for potential discrepancies in sequencing depth or experimental variability. As with ATAC-seq peaks, the standard error across samples was used to divide the gene expression values.

Following this data preprocessing, SOM clustering was performed using the kohonen R package^92^. SOM grid dimensions were selected to exceed the number of distinct experimental conditions in each dataset, ensuring sufficient resolution to capture dynamic profiles without generating redundant or sparsely populated clusters. The following SOM configurations were used: (1) 4 × 4 for DevMap RNA-seq (16 SOM clusters; exceeding the 14 developmental stages sampled, allowing resolution of overlapping expression dynamics), (2) 6 × 6 for BodyMap RNA-seq (36 SOM clusters; reflecting the higher dimensionality of ~20 unique tissue, sex and maturation combinations), (3) 5 × 5 for DevMap ATAC-seq (25 SOM clusters) and (4) 5 × 5 for BodyMap ATAC-seq (25 SOM clusters). Alternative grid configurations were evaluated by visual inspection of resulting cluster profiles and population sizes, ensuring that no clusters showed highly similar profiles and that biological variability was adequately captured. Cluster profiles were further visualized with heat maps, after ordering clusters within each analysis on the basis of a combination of maximally expressed samples, mean expression across samples and standard deviation across samples. This approach allowed us to segregate clusters into stage-specific and constitutive categories for DevMap samples and tissue-specific, multi-tissue expression and constitutive expression categories for BodyMap samples. Clusters were classified as constitutive when the standard deviation of mean expression (RNA-seq) or accessibility (ATAC-seq) values across all conditions was below 0.4, indicating a flat profile lacking condition-specific activity. Both this threshold and the tissue-specific threshold described below were empirically determined by visual inspection of cluster expression profiles and iteratively adjusted to produce classifications consistent with the observed expression patterns. For BodyMap RNA-seq, non-constitutive clusters were further classified as tissue-specific when the highest per-condition mean normalized expression value across all tissues exceeded 2.165; this threshold was selected as the lowest value that reliably separated clusters with clear single-tissue enrichment from those with broader expression across multiple tissues. Non-constitutive clusters that did not meet the tissue-specific threshold were classified as multi-tissue. These quantitative criteria were supplemented by manual curation to correct edge cases, particularly for clusters with intermediate profiles not unambiguously classified by the automated thresholds. All criteria and manual adjustments are documented in the shared analysis code.

In addition, to visualize the dynamic developmental trajectory and to differentiate samples per tissue, we applied the UMAP algorithm^93^ for dimensionality reduction on each processed matrix using the UMAP implementation in the uwot package^94^ providing a two-dimensional visual representation of the data, highlighting underlying patterns and structures in our high-dimensional dataset.

#### Cross-species SOM cluster comparisons

To assess the conservation of gene expression and chromatin accessibility between salmon and trout, we performed an enrichment analysis of orthologues across SOM clusters in both species. The analysis was conducted separately for the DevMap and BodyMap datasets, using both RNA-seq and ATAC-seq data. For this analysis, we used only 1-to-1 gene orthologues and robust ATAC-seq peaks located in syntenic regions within the same ancestral collinear block, defined by our Cactus whole-genome alignments. For each clustering, we compared all pairs of SOM clusters between salmon and trout. We generated matrices representing the observed and expected number of orthologous genes for RNA-seq and syntenic ATAC-seq peaks for chromatin accessibility. The size of these matrices varied depending on the SOM configuration, reflecting the number of clusters in each dataset. The observed number of orthologues in each cluster pair was counted directly from the data, whereas expected values were estimated using a permutation-based approach, in which cluster assignments were randomized 10,000 times to generate a null distribution of orthologous counts. The mean number of orthologues across these permutations was used as the expected value for each cluster pair. Enrichment was calculated as the ratio of observed-to-expected orthologues, providing a measure of conservation between SOM clusters. To visualize the relationships between clusters, we generated heat maps using the ComplexHeatmap R package^95^, in which rows corresponded to SOM clusters in salmon and columns to SOM clusters in trout, with colour intensity reflecting the observed-to-expected enrichment ratio. We generated Sankey plots using the ggalluvial R package^96^ to illustrate shared orthologous genes and open chromatin region between species, with nodes representing SOM clusters and edges weighted according to the number of orthologues connecting each pair.

#### Gene set enrichment for SOM clusters

For SOM clusters produced using RNA-seq datasets, we investigated enrichment of particular biological functions among gene sets comprising each SOM cluster. This analysis was done separately per species and each major dataset (that is, DevMap and BodyMap) using the GO framework and the package clusterProfiler^97^ restricting to ‘biological process’ terms and using salmon and trout annotations produced by Ensembl, which were loaded into R using AnnotationForge^98^ and biomaRt^99^. For DevMaps, GO enrichment was performed on each SOM cluster. For BodyMaps, SOM clusters were manually curated according to tissue-specific expression, with SOM clusters showing constitutive expression used as an additional category. For each test, the background genes in the test were all genes expressed above 1 TPM in at least one sample for each dataset separately. Enriched GO terms were filtered using a custom script and the package GO.db^100^ to retain only parent terms, therefore reducing the presentation of highly redundant GO terms. Shared GO terms among SOM clusters were visualized using the clusterProfiler function compareCluster.

### Validation of ChromHMM annotations using CAGE

CAGE-seq followed a previously published protocol^101^ with sequencing compatibility optimizations. To improve sequencing efficiency and demultiplexing accuracy on Illumina platforms, the legacy 3-bp barcode at the start of Read 1 was replaced with 8-bp TruSeq Unique Dual Indexes. Adapter ligation and reverse transcription were followed by an additional five to six cycles of PCR amplification using NEBNext Ultra II Q5 Master Mix (New England BioLabs, no. M0544S) to incorporate Illumina P5/P7 flow cell adaptors and I5/I7 indexes. Library purification was performed using 1.4× AMPure XP beads (Beckman Coulter, no. A63881) to remove adapter dimers and short fragments. Final libraries were quality-checked using a High Sensitivity DNA Bioanalyzer (Agilent, no. 5067-4626) and quantified with a Qubit dsDNA High Sensitivity Assay Kit (Thermo Fisher, no. Q32854). Libraries were sequenced on an Illumina NextSeq 2000 with the flow cell P4-50 kit, generating 70-bp single-end reads. Following sequencing, raw reads were demultiplexed using standard Illumina processing pipelines. Adapter sequences and low-quality reads were trimmed and filtered using Trim Galore! with default settings. Quality-filtered reads were aligned to the Salmon and Trout reference genomes using Bowtie2 v2.5.0^102^ in end-to-end alignment mode, ensuring that the 5′ end of each read was correctly mapped to its genomic location. Alignments were filtered to retain only reads with mapping quality greater than 20, ensuring only primary, uniquely mapped reads were retained before being converted to BAM format using SAMtools v1.5.

Mapped data were processed using CAGEr v2.11.4^103^ following the recommended workflow. First, the artificial G nucleotide added during reverse transcription was removed from the 5′ end of each read to correct for CAGE-specific biases. A probabilistic approach was used to distinguish added matching Gs from non-added matching Gs. Signal normalization was performed using power-law normalization, and transcription start site (TSS) clusters were identified with a clustering threshold of 20 bp. Clusters were further filtered to remove artefacts and low-expression peaks, and genomic coordinates were converted to a standardized format for quality validation of ChromHMM annotations.

### Ohnologue expression divergence analysis

#### Jensen–Shannon distance

We calculated pairwise expression distances for defined classes of ohnologue and orthologue pairs (data shown in Fig. 3). We first calculated the mean of biological replicates of TPM values for each gene, separately for different samples. We then independently performed quantile normalization across DevMap and BodyMap samples using the preprocessCore R package^91^. Next, the standard error across samples was divided by the gene expression values. The ohnologues and orthologues were extracted from our homology prediction (Supplementary Table 6) for comparison. Specifically, we compared expression data four non-overlapping gene-pair categories: (1) 8,757 2:2 AORe (early rediploidization) pairs, which started diverging in the salmon and trout ancestor; (2) 1,103 2:2 LORe (late) rediploidization pairs, where two ohnologue pairs are conserved in both salmon and trout but diverged independently in each species; (3) 8,661 orthologue pairs representing AORe 2:2 ohnologues and (4) 5,099 orthologue pairs that represent singletons from AORe regions. Within each category, the orthogroup ID was used to segregate gene pairs into separate columns (named geneA and geneB). Expression divergence between the two members of a pair was measured using Jensen–Shannon distance (JSD). Before the distance calculation, the two normalized TPM profiles were jointly quantile-normalized using the preprocessCore R package and rescaled by dividing each value by the sum of all values. JSD values were then obtained using the JSD function of the phylentropy R package^104^. To capture uncertainty, 100 bootstrap replicates were drawn for every embryonic stage (DevMaps) or tissue/maturity (BodyMaps) combination by resampling gene-pair indices with replacement. Within each replicate, we recalculated JSD for the sampled set and subsequently recorded the mean and standard deviation across replicates. To assess the correlation of divergence patterns between categories, we calculated Pearson’s correlation between the sample-wise mean JSD values; scatter plots, regression lines and 95% confidence intervals were produced with the ggpubr R package^105^.

#### Assignment of ohnologue pair expression to SOM clusters

Separately for our DevMap or BodyMap transcriptomic datasets, we first classified all salmon genes assigned to SOM clusters into early and late rediploidization categories using our homologue prediction (Supplementary Table 6). We then tested if the assignment of these genes to different SOM clusters varied across stages (DevMap) or tissues (BodyMap) and if this was affected by rediploidization category. This was done using chi-squared tests with a SOM cluster × rediploidization category contingency table, and we also tested for per SOM cluster enrichment or depletion of early rediploidization ohnologues against the genome-wide ratio (7.22 for DevMap; 6.96 for BodyMap) using two-sided binomial test with Benjamini–Hochberg correction. Our ohnologue pair SOM co-clustering analysis used all two-copy salmon ohnologue pairs where both genes were assigned to SOM clusters (*n* = 8,827 genes for DevMap analysis; 10,817 genes for BodyMap analysis). Aggregate co-clustering rates of ohnologue pairs from the early versus late rediploidization categories were compared with Fisher’s exact test, and per stage (for DevMap) and per tissue (for BodyMap), with co-clustering rates defined as the proportion of ohnologue genes per group whose partner gene was found in the same SOM cluster. Stage-dependent trends in ohnologue pair co-clustering rates within each rediploidization category were further tested using the Cochran–Armitage trend test. To verify that heterogeneity in co-clustering per SOM cluster for late rediploidization ohnologue pairs (much less numerous than early rediploidization ohnologue pairs) captured real biological signal, we additionally tested per SOM cluster heterogeneity using chi-squared tests and compared Spearman rank correlations for per SOM cluster ohnologue pair co-clustering rates between early and late rediploidization categories (restricted to non-constitutive clusters with at least ten ohnologue genes in each rediploidization category). Sankey diagrams of ohnologue pair SOM cluster transitions were made in ggalluvial^91^. The ohnologue pair SOM cluster assignment intersection diagrams were made using UpSetR^106^.

### Evolution of regulatory elements

Genomic coordinates of the unified set of 319,718 salmon robust open chromatin regions were provided as a BED file to the mafExtractor command within mafTools, allowing us to extract each robust open chromatin region within our whole genome alignment in MAF format. A custom R script was used to process each resulting alignment file to extract the start and end coordinates for sequences (Ssal_A, Ssal_B, Omyk_A, Omyk_B, Eluc) present in the MAF file. Tabular data from individual chromosomes was merged into a single dataset and curated to remove spurious alignments, including nested alignments where Ssal_B coordinates were identical for multiple robust open chromatin region (9,250 regions) and instances where a single robust open chromatin region overlapped two regions on the same chromosome (4,996 regions). Deduplication was performed to eliminate duplicate representation of robust open chromatin regions across the two duplicated chromosomes. The final output captures the genomic coordinates of 225,801 reference robust open chromatin regions (Ssal_A) along with the coordinates of their syntenic, orthologous regions (Ssal_B, Omyk_A, Omyk_B, Eluc) (Supplementary Data 2).

We grouped the robust open chromatin regions into three categories: (1) ‘shared’, representing robust open chromatin regions conserved in syntenic sequences located on two duplicated chromosomes represented in the whole genome alignment; (2) ‘alignable only’, representing syntenic sequences conserved on two duplicated chromosomes represented in the whole genome alignment but with a robust open chromatin region found on only one of these duplicated regions; and (3) ‘exclusive’, representing robust open chromatin regions specific to a single chromosome (that is, no evidence that the region is retained as a duplicated sequence from the salmonid WGD). A schematic of these regions is provided in Fig. 4 of the main text.

To identify ‘shared’ regulatory elements, robust open chromatin regions overlapping both duplicated regions (that is, Ssal_A and Ssal_B) were selected, and the percentage overlap between duplicated robust open chromatin regions calculated. The summit regions for robust open chromatin regions (defined from ATAC-seq peaks) were overlaid onto these overlapping regions to filter for high-confidence regulatory elements, where the summit coordinates fell within the overlapping regions for both duplicates (Ssal_A and Ssal_B). For ‘exclusive’ regulatory elements, the alignments were filtered to retain regions where both the sequence and robust open chromatin regions were confined to Ssal_A or Ssa_B. Similarly, ‘alignable only’ regions were identified by filtering for alignments where sequence conservation was observed across both duplicated chromosomes (Ssal_A and Ssal_B), but a robust open chromatin region was present only in Ssal_A or Ssa_B.

We next overlapped the robust open chromatin regions from each category with our ChromHMM annotation to identify those representing active promoters (45,802/225,801) or active enhancers (78,424/225,801) across the unified dataset spanning all samples. This identified 16,938/14,836, 6,845/19,546 and 7,873/10,232 active promoters/enhancers belonging to the ‘shared’, ‘alignable only’ and ‘exclusive’ categories, respectively (Supplementary Data 3). The promoter and enhancer elements within these three categories were further split into early and late rediploidization categories using the genomic coordinates across the salmon genome (Supplementary Table 10). The activity of categorized regulatory elements (presence or absence), was assessed for individual sample types in the BodyMap and DevMap atlases. Specifically, for active promoters and enhancers, we calculated the proportion of each within the ‘shared’, ‘alignable only’ and ‘exclusive categories’ across each DevMap stage of embryogenesis and across different BodyMap tissue and maturation stage samples, independently for early and late rediploidization regions (results in Fig. 4; Supplementary Table 13). Fisher’s exact tests (with Bonferroni correction) were performed to assess statistical significance across different sample types for all possible combinations within each alignment category and separately for early and late rediploidization regions (Supplementary Table 14). Pearson’s correlation was performed across all sample types and per rediploidization category to compare the proportions of active promoters or enhancers within the ‘shared’, ‘alignable only’ and ‘exclusive’ categories versus JSD values for 2:2 ohnologue pairs in salmon (see previous section) (Supplementary Table 15).

#### TE content within regulatory elements

A curated TE dataset for salmon^59^ was used as input to RepeatMasker to annotate TE regions across the salmon genome. TE annotations were filtered to remove low complexity, satellite and simple repeats. TE content was calculated as the percentage of active enhancer bases overlapping TE sequences out of the total sum of active enhancer bases. This was calculated separately for active enhancers found in each sample type and those classified into the ‘shared’, ‘alignable only’ and ‘exclusive’ categories. Pearson’s correlation was performed across all sample types and per rediploidization region to compare the proportion of active enhancers within each alignment category versus the proportion of TEs overlapping active enhancers (Supplementary Table 16).

#### Evolutionary fate of enhancer-CNEs

CNEs were defined in the northern pike genome (fEsoLuc1.pri; GCA_011004845.1) on the basis of a whole genome alignment of 46 species including human alongside a broad selection of actinopterygian lineages (teleosts and non-teleosts), inclusive of salmon and trout. The approach to call CNEs and identify their phylogenetic age followed Naville et al.^107^. Using the northern pike genome as a reference allowed us to (1) identify northern pike CNEs in our whole genome alignment and (2) leverage their co-orthology to putative salmon CNE ohnologues. The approach in full is as follows.

Starting from a 46-species Multiz alignment with fEsoLuc1 as the reference genome, we used GERP++ gerpcol^108^ to compute the GERP conservation score (that is, number of rejected substitutions) at all positions and then filter out aligned columns to keep only those fulfilling all the following conditions: (1) belong to a conserved element with a significant GERP score (false discovery rate 0.2); (2) do not overlap with any annotated exon for any of the aligned genomes nor with any annotated pike repeat sequence; and (3) still belong to an aligned block of at least ten consecutive bases after filtering steps 1 and 2. For each of these blocks, we attempt to assign putative target gene(s) using synteny conservation: among all pike genes located within a fixed radius around the block, we choose as putative target(s) the one(s) which are most often also found in the vicinity of the orthologous block in other genomes. Then, blocks less than 100 bp away and targeting the same genes in the same species were merged into single units that we consider CNEs. Therefore, one CNE can be made of several conserved non-coding blocks. After this merging process, we obtained 416,589 CNEs in the northern pike genome (Supplementary Data 4), which spanned 21,136,463 bp. Each of these CNEs was assigned a putative age, which corresponds to the most recent ancestor of all the species that possess an orthologous CNE: Euteleostomi (ancestral to ray-finned fish and mammals); Actinopteri (ancestral to ray-finned fish, excluding Polypteriformes); Neopterygii (ancestral to holostean and teleost fish); Teleostei (ancestral to teleost fish); Clupeocephala (ancestral to clupeocephalan teleosts, excluding Osteoglossomorpha and Elopomorpha); Euteleosteomorpha (ancestral to euteleost fish, excluding Otocephala); and Protacanthopterygii (ancestral to protacanthopterygians, including Esociformes and Salmonidae).

We categorized and extracted CNEs captured in our whole genome alignment, requiring that 100% of the northern pike CNE sequence was captured and located within an alignment belonging to the shared, alignable only or exclusive categories, previously categorized as representing active enhancers in salmon. These extractions were performed using Maftools to generate maf files capturing individual CNEs. At this stage, we identified 21,928, 14,040 and 2,565 individual enhancer-CNEs belonging to the shared, alignable only and exclusive categories, respectively. Each MAF alignment was subsequently processed through an R-based filtering pipeline applying the following criteria to retain highly credible CNEs: (1) at least 90% of the CNE sequence length was present among any of the northern pike or salmon sequences within each MAF alignment block; (2) the alignment block contains zero gaps for any northern pike and salmon sequences captured; and (3) at least one salmon co-orthologue must exhibit >80% sequence similarity to the pike orthologue. Final counts and proportions of salmon enhancer-CNEs belonging to each alignment category were provided across the genome and separated by the early and late rediploidization regions (Supplementary Table 18), with information provided for individual CNEs in Supplementary Data 5.

Nucleotide sequence percentage identity was separately calculated between salmon enhancer-CNE ohnologue pairs (salmon A versus B) and compared between the shared and alignable only categories. To test if the rate of sequence evolution in enhancer-CNE ohnologues depends on enhancer-CNE activity, we first calculated nucleotide sequence percentage identity separately for each enhancer-CNE within an ohnologue pair in comparison with their shared pike CNE orthologue (that is, salmon A versus pike and salmon B versus pike). We then compared differences in sequence divergence against pike for A and B ohnologues for the shared (A active, B active) and alignable only (A active, B inactive) categories. Each of the above analyses was done globally, then stratified by each phylogenetic age of CNEs, with differences between groups of interest assessed using Wilcoxon’s rank-sum test, using the Bonferroni–Holm method to obtain *P*_adj_ values accounting for multiple comparisons.

#### Enhancer-CNE motif enrichment analyses

Enhancer-CNE sequences belonging to the different alignment categories were used as the input for TF motif enrichment analysis using GIMME Maelstrom^85^. Separate analyses were performed to compare motif enrichment in enhancer-CNE sequences contrasting: (1) all enhancer-CNEs across the phylogenetic age categories and (2) enhancer-CNEs split into the different alignment categories across the phylogenetic age categories. As GIMME Maelstrom requires input sequences of at least 200 bp, and given that a substantial proportion of enhancer-CNE sequences were shorter than 50 bp, a merging and expansion strategy was applied before each iteration using a custom R script. Within each comparison, adjacent-CNEs with boundaries within 200 bp were merged into a single interval, providing they belonged to the same age/alignment category. CNEs from different categories were kept as separate entries. Any CNE-enhancer interval that remained below 200 bp after merging (or was shorter than 200 bp without merging) was symmetrically expanded from its centre to reach a minimum length of 200 bp. In situations where a CNE-enhancer interval from one category overlapped with another, the overlap was tolerated. Overall, each interval retained its original category identity throughout, ensuring that GIMME Maelstrom received stable, consistently sized windows for motif scoring without distorting the categorical structure of the data.

GIMME Maelstrom was run using the -no-filter flag against the gimme.vertebrate.v5.0 motif database. CNE coordinates were supplied in TSV format for the Atlantic salmon reference genome, with the comparison categories as a separate column for each run. Output results were filtered to retain the top 60 enriched motifs per comparison category using the Z-score as a metric (results shown in Supplementary Tables 21 and 22). Furthermore, for each analysis, we selected the top ten non-redundant enriched motifs per comparison category and generated heat maps to visualize all *Z*-scores using ComplexHeatmap^95^ within R (Extended Data Fig. 8 and Supplementary Fig. 38).

#### Enhancer-CNE activity across ontogeny

To explore the impact of ontogeny, tissue-context and rediploidization on the evolution of enhancer-CNEs following the salmonid WGD, we calculated the number and length of enhancer-CNEs per: (1) each sample type defined within our DevMap and BodyMap atlases (averaged across sex for the BodyMap data), (2) each alignment category (shared, alignable only and exclusive) and (3) each rediploidization region (early and late). The results are shown in Supplementary Data 6. To normalize the above comparisons, CNE counts were divided by the total length of CNEs across the three alignment categories for each phylogenetic age across all sample types. The normalized proportion of enhancer-CNEs in the shared, alignable only and exclusive categories was separately plotted for early and late rediploidization regions per phylogenetic age and sample type. Fisher’s exact test was used to test for enrichment of enhancer-CNEs in early versus late rediploidization regions, comparing the length of enhancer-CNEs overlapping each region, against the total length for early (2,023,914,877 bp) and late rediploidization regions (649,544,050 bp) (Supplementary Table 19). This analysis was repeated after splitting enhancer-CNEs within each age category into the shared, alignable and exclusive alignment categories (Supplementary Table 20).

Fisher’s exact test was used to compare: (1) all possible CNE phylogenetic age combinations per sample type, alignment category and rediploidization region (Supplementary Table 23); (2) all possible alignment categories per sample type, rediploidization region and CNE phylogenetic age category (Supplementary Table 24); and (3) all possible sample types per enhancer-CNE phylogenetic age, rediploidization region and alignment category (Supplementary Table 25).

### Linking enhancer evolution to ohnologue regulatory divergence

A series of steps were done to investigate the extent to which salmon ohnologue pair expression divergence was coupled to changes in *cis*-enhancer elements proximal to these genes.

The first step was to define regulatory domains for ohnologue genes, following ref. ^51^. Each gene’s regulatory domain was defined by an ‘exclusive’ region 5 kb upstream and 1 kb downstream of its TSS. Next, an ‘overlapping’ region extended up and downstream of the exclusive region, until meeting another gene’s exclusive region or a maximum distance of 1 Mb from the TSS. The exclusive and overlapping regions together defined the regulatory domain. Exclusive regions can overlap each other, and overlapping regions can overlap each other, but overlapping regions cannot overlap exclusive regions.

The second step was to annotate *cis*-enhancers by overlapping robust open chromatin regions with active enhancer annotations from our ChromHMM annotation across all sample types. Gene regulatory domain regions were used to assign the enhancers falling within the domains to the respective gene. With this approach, enhancers can be assigned to more than one gene when domains overlap. All enhancer ohnologue pairs were assigned to the shared, alignable only or exclusive category using our whole genome alignment, as described above. When enhancers were located outside a gene’s regulatory domain, yet aligned to an enhancer located within the other ohnologue’s regulatory domain, they were taken forward in the analysis.

The third step was to delineate ohnologue pairs into two extreme categories of expression conservation: ‘conserved’ and ‘diverged’. This was done using Pearson’s correlation with scaled TPM values averaged across biological replicates for all sampled conditions (that is, all DevMap stages of embryogenesis, plus the different BodyMap tissues from the two maturation stages), for both ohnologue pairs of the same species and between 1:1 orthologue pairs between species, using our homology prediction (Supplementary Table 6). We used Pearson correlation value thresholds to classify 10,366 ohnologue groups (2:2 ohnologue pairs from both species) into the conserved and diverged groups. A set of 3,146 ohnologue groups were defined as conserved, on the basis of Pearson correlation values >0.7 for the 2:2 ohnologue pairs of both salmonid species and >0.5 between the equivalent orthologue pairs across species. A set of 1,346 ohnologue groups were defined as diverged, on the basis of Pearson correlation values ≤0.3 for the 2:2 ohnologue pairs of both salmonid species. We further checked if gene expression level was a confounding factor distinguishing the ‘conserved’ and ‘diverged’. We compared Pearson correlation values against expression levels, represented as the maximum counts per million in a given sample type (median across replicates) for either ohnologue gene in the pair (Extended Data Fig. 7c).

Regulatory landscape differences between 2:2 ohnologues were compared between the ‘diverged’ and ‘conserved’ expression groups from salmon. For statistical analysis, unless stated otherwise, the Wilcoxon rank-sum test was chosen for tests of differences between the distribution of values of ‘conserved’ and ‘diverged’ ohnologues, with 95% confidence intervals computed from median values using bootstrapping with 10,000 replicates. The number of enhancers per gene was compared between ‘conserved’ and ‘diverged’ ohnologues. Differences in enhancer membership to the alignment categories (exclusive, shared, alignable only) between ‘conserved’ and ‘diverged’ ohnologue pairs were tested using binomial generalized linear models, fitted for enhancer proportions with ohnologue conservation status as the predictor, reporting odds ratios with 95% confidence intervals. Multiple comparisons were Holm-adjusted, and global differences across categories additionally tested using a 3 × 2 chi-squared test. Similarity in the activity of shared enhancers was compared by dividing the number of times shared enhancers of an ohnologue enhancer group were annotated as active by ChromHMM in the same sample type, over the total unique number of samples the shared enhancers were active. We compared differences between ohnologues in their ‘enhancer-usage’ across conditions. For this purpose, we calculated, per sample type, the proportion of enhancers active as a product of the total number of active enhancers for each gene. At least one active enhancer for each gene was required to test an ohnologue pair. The Euclidean distance between the two vectors of proportions was calculated between ohnologue pairs, termed ‘enhancer-usage distance’. Distances of ‘diverged’ ohnologues were then compared with ‘conserved’ ohnologues.

Next, we sought evidence for a direct relationship between conservation/divergence in gene expression and regulatory landscape differences between ohnologue pairs. For this purpose, both enhancer-usage Euclidean distance and gene expression Euclidean distances were calculated as described above for all 10,366 ohnologue pairs, separately for DevMap and BodyMap samples. SOM cluster identifications, assigned to genes on the basis of gene co-expression maps, were used to subset ohnologue groups into specific developmental stages or tissue types. At least one gene of the ohnologue pair needed to be assigned to a specific stage or tissue. The ohnologue pairs were allowed to have two assignments if each gene belonged to different SOM clusters. Finally, ohnologue pair expression distance was plotted against enhancer-usage distance separately by developmental stage or tissue type, with density levels visualizing clusters of ohnologue pairs showing the relationships between active enhancer-usage and expression divergence. We used a global Kruskal–Wallis rank-sum test to compare overall differences in dispersion of expression and enhancer distances between sample types. To test if ohnologues clustered around a single or multiple peaks (representing regulatory conservation or divergence), we used a two-component mixture model (Mclust R package^109^) to split ohnologues into two clusters and compared between sample types the proportion of ohnologue pairs fitting into the first or second cluster.

Finally, we compared TE content in active enhancer sequences of ‘conserved’ and ‘diverged’ ohnologues. The proportions TE content, calculated as base pairs of TE regions overlapping active enhancers regions, out of total enhancers base pairs, was determined per ohnologue pair separately by enhancer categorization (shared, alignable and exclusive). TE content was also separately measured for enhancers binned by distance to gene TSS. TE content in exclusive enhancers was then broken down by four major TE orders: DNA, LINE, LTR and SINE, and specifically by the Tc1/mariner (TcMar) class of DNA transposons, the most abundant class of TE in the salmon genome. TE content for TcMar transposons were then broken down by sample types, that is, different developmental stages and tissues. Means of values per ohnologue pair were used for bootstrapping 95% confidence intervals and for Wilcoxon tests between ‘conserved’ and ‘diverged’ ohnologues.

### Reporting summary

Further information on research design is available in the Nature Portfolio Reporting Summary linked to this article.

## Supplementary information

Supplementary Information Supplementary Figs. 1–38 and Texts 1–9.

Reporting Summary

Peer Review File

Supplementary Tables 1–25 Supplementary Tables 1–25.

## Figures and Tables

**Extended Data Fig. 4:**
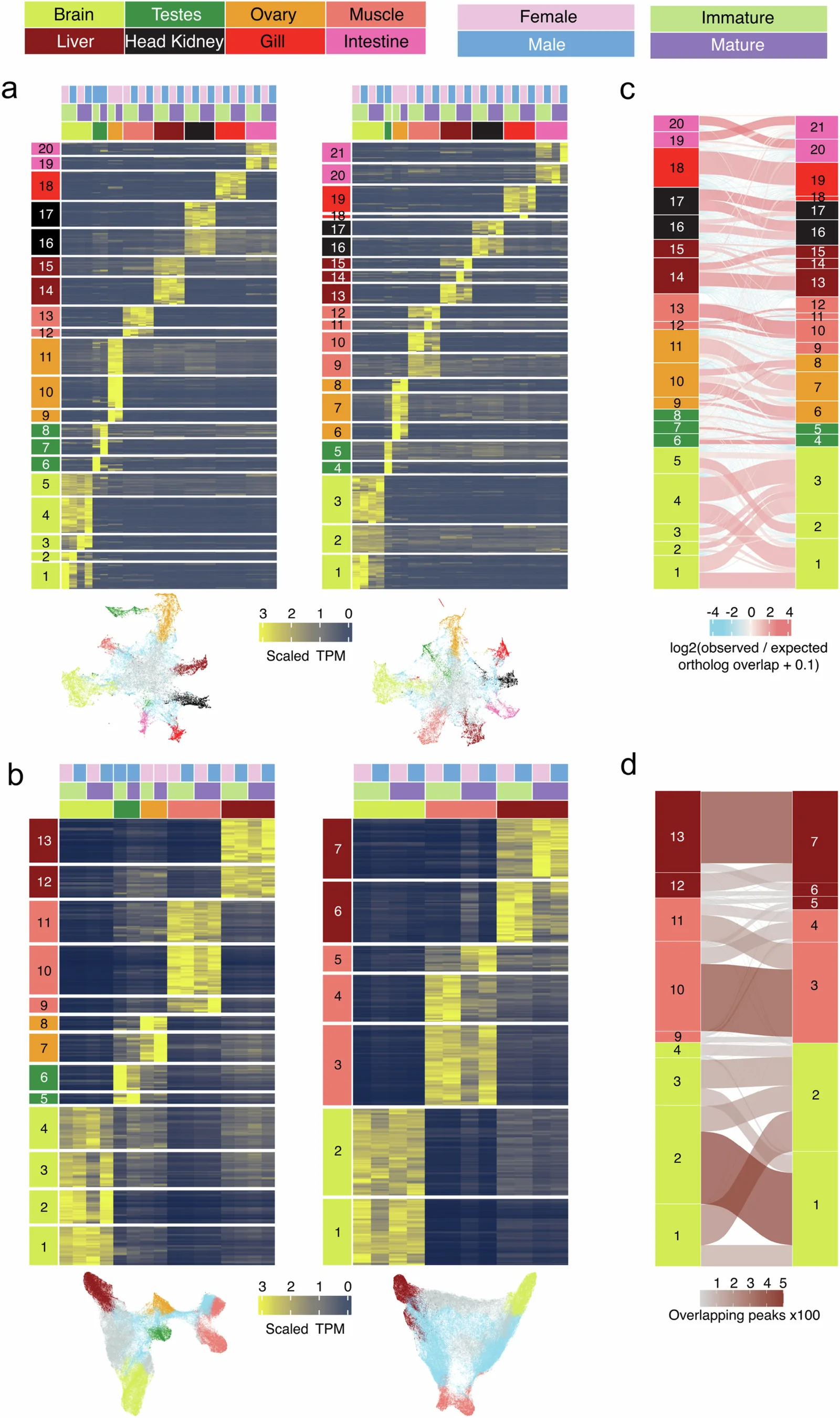
Functional activity of salmonid genomes across different tissues. Schematic at top illustrates colors used for tissues, sex and stage of maturity throughout the figure. (**a**) Transcriptome dynamics across tissues. SOM cluster heatmaps for salmon (left) and trout (right) show mean normalised transcripts per million (TPM) values across tissues, with rows split by tissue-specific SOM clusters (full data shown in Supplementary Figs. 22-23), cluster number were matched across figures. Below each heatmap, UMAP plots show dimensionality reduction of the multivariate normalised expression profiles across all tissues, where each point represents an individual gene positioned according to its expression profile similarity to other genes. Points are coloured by SOM cluster assignment; grey points indicate genes assigned to constitutive SOM clusters; blue points indicate genes assigned to multi-tissue SOM clusters (Supplementary Figs. 22-23). (**b**) Chromatin openness dynamics across tissues. SOM cluster heatmaps for salmon (left) and trout (right) show mean normalised ATAC-seq read intensity values in robust open chromatin regions across three tissues with ATAC-seq data (brain, muscle and liver). Rows are split by stage-specific SOM clusters (full data shown in Supplementary Figs. 24-25). UMAP plots below show dimensionality reduction of multivariate normalised accessibility profiles, where each point represents a single robust open chromatin region. Point colouring as per panel a. (**c**) Cross-species comparison of transcriptomic SOM clusters. Sankey diagram showing the number of shared 1-to-1 orthologous genes between salmon (left) and trout (right) tissue-specific SOM clusters. Band width is proportional to the number of shared orthologs between each pair of clusters. Band colour represents the log_2_ observed-to-expected ratio of orthologous gene sharing (colour scale shown; blue: fewer shared orthologs than expected by chance; red: more shared orthologs than expected). Expected values were estimated from 10,000 random permutations of cluster assignments (see Methods). (**d**) Cross-species comparison of chromatin accessibility SOM clusters. Sankey diagram showing the number of syntenic open chromatin regions shared between salmon and trout ATAC-seq SOM clusters, based on whole genome alignment. Band width is proportional to the number of overlapping syntenic open chromatin regions between each cluster pair. Band colour represents the number of shared regions.

## Data Availability

All data required to reproduce and expand the results reported in this study have been shared. The raw sequencing datasets are available via the FAANG data portal at https://data.faang.org/projects/AQUA-FAANG. ENA accession numbers are shared in Supplementary Table 2 and are as follows: PRJEB51855, PRJEB51854 and PRJEB53399 (salmon DevMap: RNA-seq, ATAC-seq and ChIP-seq, respectively); PRJEB51857, PRJEB51856 and PRJEB55010 (trout DevMap: RNA-seq, ATAC-seq and ChIP-seq, respectively); PRJEB47409, PRJEB47408 and PRJEB55063 (salmon BodyMap: RNA-seq, ATAC-seq and ChIP-seq, respectively); and PRJEB57191, PRJEB57190 and PRJEB57956 (trout BodyMap: RNA-seq, ATAC-seq and ChIP-seq, respectively). Processed datasets are also provided via salmobase.org^34^, including RNA-seq TPM values, ATAC-seq and ChIP-seq peak files, robust open chromatin regions, ChromHMM-annotated robust open chromatin regions, whole genome alignments and our homology prediction. Supplementary data files and source data for all figures and extended data figures are available via Zenodo at https://doi.org/10.5281/zenodo.21415252 (ref. ^110^).

## Code availability

All code required to reproduce the analyses reported is available via GitHub at https://github.com/AQUA-FAANG/AQUA-FAANG-salmonids and via Zenodo at https://doi.org/10.5281/zenodo.21410850(ref. ^70^).
