# Supplementary material for: Developmental contexts of gene-regulatory evolution following whole genome duplication in salmonids
Source: Nat Ecol Evol. 2026 Sep 29;10(10):1959–81. doi: 10.1038/s41559-026-03171-6 (PMC13642036; doi:10.1038/s41559-026-03171-6)
Supplement: Supplementary file 1 [file 41559_2026_3171_MOESM1_ESM.pdf]

nature ecology & evolution

Supplementary information https://doi.org/10.1038/s41559-026-03171-6

Developmental contexts of gene-regulatory evolution following whole genome duplication in salmonids

In the format provided by the authors and unedited

Table of contents

Supplementary Fig. 1. Graphical visualization of protocol development for sequencing assays used to functionally annotate two salmonid genomes. 3

Supplementary Fig. 2. Principal component analysis (PCA) of RNA-Seq and ATAC-seq datasets for DevMap and BodyMap from salmon and trout. 4

Supplementary Fig. 3. PCA of ChIP-seq datasets for DevMap from salmon and trout. 5

Supplementary Fig. 4. PCA of ChIP-seq datasets for BodyMap from salmon and trout. 6

Supplementary Fig. 5. Overlap of robust open chromatin regions with annotated genome features. 7

Supplementary Fig. 6. Overlap of ChIP-seq peaks with annotated genome features for H3K27ac. 8

Supplementary Fig. 7. Overlap of ChIP-seq peaks with annotated genome features for H3K4me1. 9

Supplementary Fig. 8. Overlap of ChIP-seq peaks with annotated genome features for H3K4me3. 10

Supplementary Fig. 9. Overlap of ChIP-seq peaks with annotated genome features for H3K27me3. 11

Supplementary Fig. 10. Transcription factor binding sites (TFBS) enrichment analysis across DevMap stages in salmon and trout. 12

Supplementary Fig. 11. TFBS enrichment analysis across BodyMap tissues in salmon and trout. 13

Supplementary Fig. 12. Overlap between enriched TFBS for salmon and trout in DevMap and BodyMap sample types. 14

Supplementary Fig. 13. Homology prediction capturing ohnologs (within) and orthologs (between) for the salmon and trout genomes (see Methods). 15

Supplementary Fig. 14. Comparison of effectiveness of two whole genome alignment strategies for capture of duplicated regions from salmonid WGD. 16

Supplementary Fig. 15. Salmon DevMap transcriptome SOM clusters. 17

Supplementary Fig. 16. Trout DevMap transcriptome SOM clusters. 18

Supplementary Fig. 17. Salmon DevMap chromatin openness SOM clusters. 19

Supplementary Fig. 18. Trout DevMap chromatin openness SOM clustering. 20

Supplementary Fig. 19. Enriched gene ontology (GO) terms for DevMap transcriptome of salmon. 21

Supplementary Fig. 20. Enriched gene ontology (GO) terms for DevMap transcriptome of trout. 22

Supplementary Fig. 21. Matrix of observed to expected ratio of orthologs shared across all combinations of salmon and trout DevMap SOM clusters. 23

Supplementary Fig. 22. Salmon BodyMap transcriptome clusters 24

Supplementary Fig. 23. Trout BodyMap transcriptome clusters. 25

Supplementary Fig. 24. Salmon BodyMap chromatin openness SOM clusters......................26
Supplementary Fig. 25. Trout BodyMap chromatin openness SOM clusters. 27
Supplementary Fig. 26. Enriched gene ontology (GO) terms for BodyMap transcriptome of salmon..28
Supplementary Fig. 27. Enriched gene ontology (GO) terms for BodyMap transcriptome of trout...29
Supplementary Fig. 28. Matrix of observed to expected ratio of orthologs shared across all combinations of salmon and trout BodyMap SOM clusters...30
Supplementary Fig. 29. Scatterplots comparing mean JSD values between pairs of ohnologs and orthologs across ontogeny and tissues. 31
Supplementary Fig. 35. Ontogeny and tissue context constrain the overlap between TEs and active enhancers..39
Supplementary Fig. 37. Enhancer-CNEs are differentially enriched in the salmon genome dependent on rediploidization history...42
Supplementary Fig. 38. Differential enrichment of DNA motifs in enhancer-CNEs of distinct phylogenetic ages..43
Supplementary Text 1. Sampling Protocols for DevMap and BodyMap 44
Supplementary Text 2: DevMap and BodyMap RNA extraction protocols............................51
Supplementary Text 3: DevMap & BodyMap Omni-ATAC-seq...55
Supplementary Text 4. DevMap & BodyMap ChIP-seq..68
Supplementary Text 5. Transcription factor binding site (TFBS) enrichment results.............81
Supplementary Text 6. Transcriptome dynamics during embryogenesis................................84
Supplementary Text 7. Transcriptome dynamics across tissues at different maturation stages 87
Supplementary Text 8. Additional text on regulatory element evolution (supporting Fig. 4).90
Supplementary Text 9. Differential enrichment of motifs in enhancer-CNEs across different CNE phylogenetic age categories. 91
Supplementary References...91

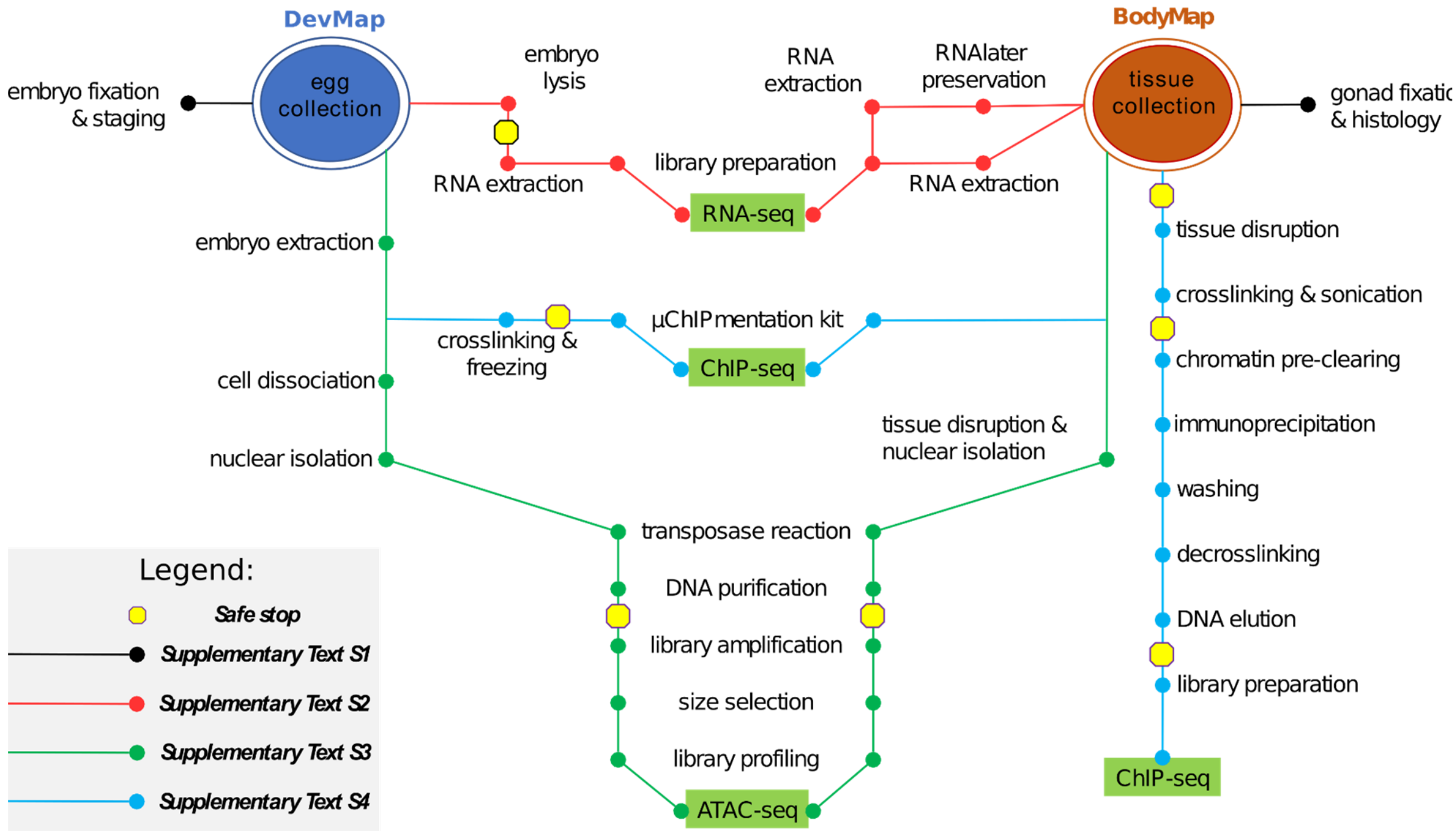

Supplementary Fig. 1. Graphical visualization of protocol development for sequencing assays used to functionally annotate two salmonid genomes. The map illustrates interconnections between different protocols fully outlined in Supplementary Text 1-4.

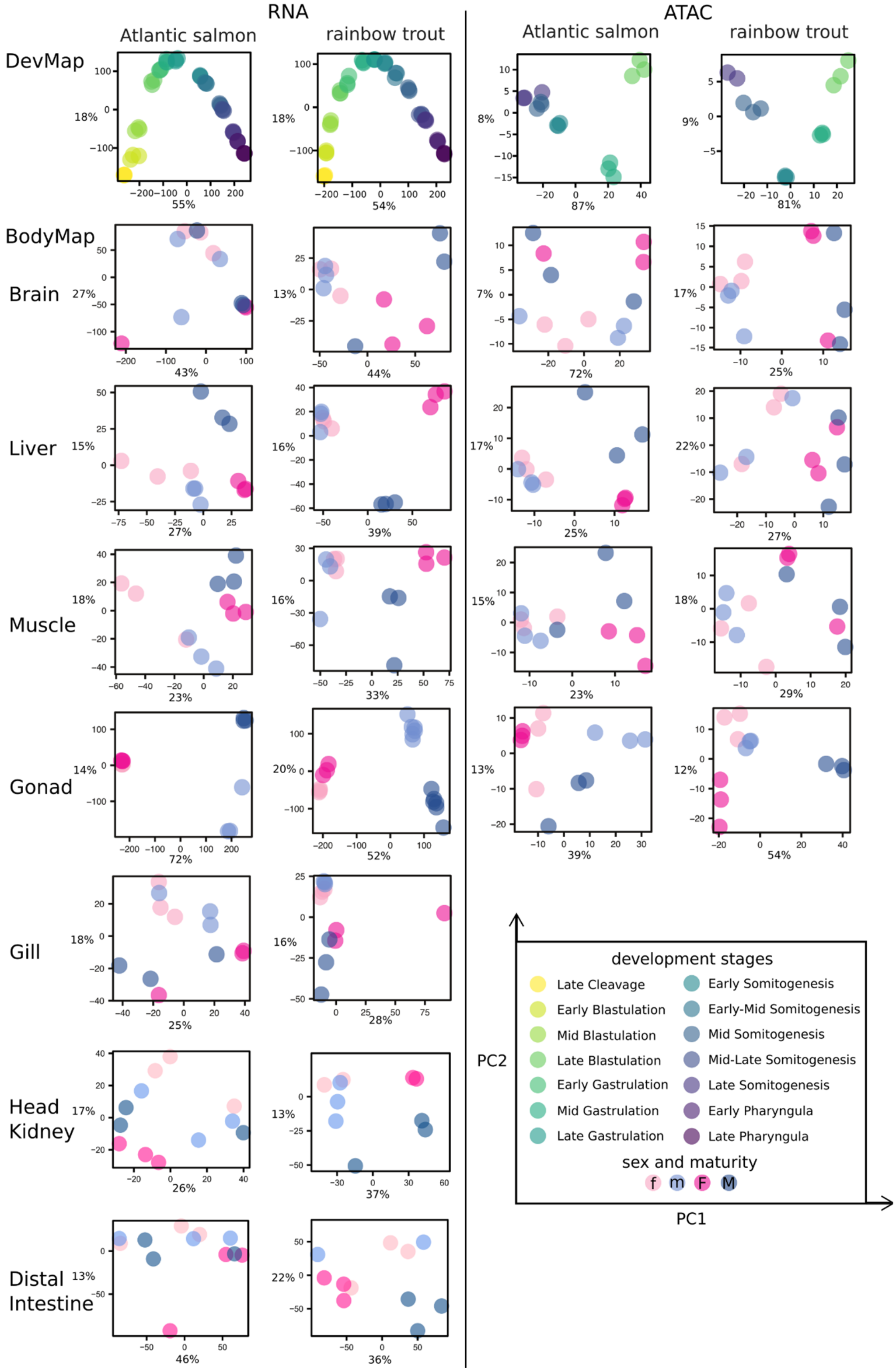

Supplementary Fig. 2. Principal component analysis (PCA) of RNA-Seq and ATAC-seq datasets for DevMap and BodyMap from salmon and trout. The key at the bottom right highlights different DevMap stages or symbols used for sexes in BodyMap (tissues separated into immature = lowercase 'f' or 'm' and mature = uppercase 'F' and 'M'). The proportion of variation explained by PC1 and PC2 is shown on each plot.

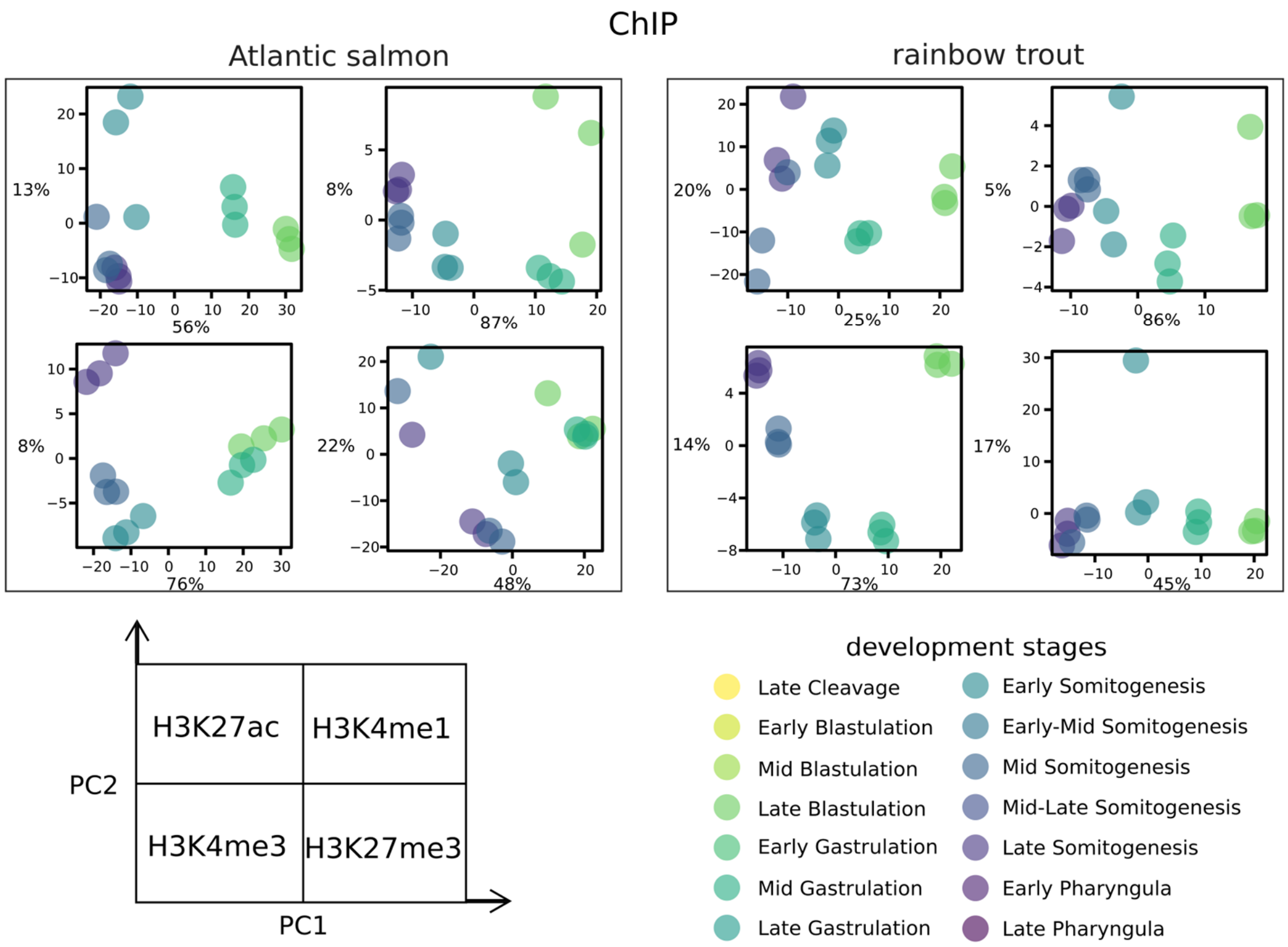

Supplementary Fig. 3. PCA of ChIP-seq datasets for DevMap from salmon and trout. The key at the bottom right highlights different DevMap stages. The proportion of variation explained by PC1 and PC2 is shown on each plot.

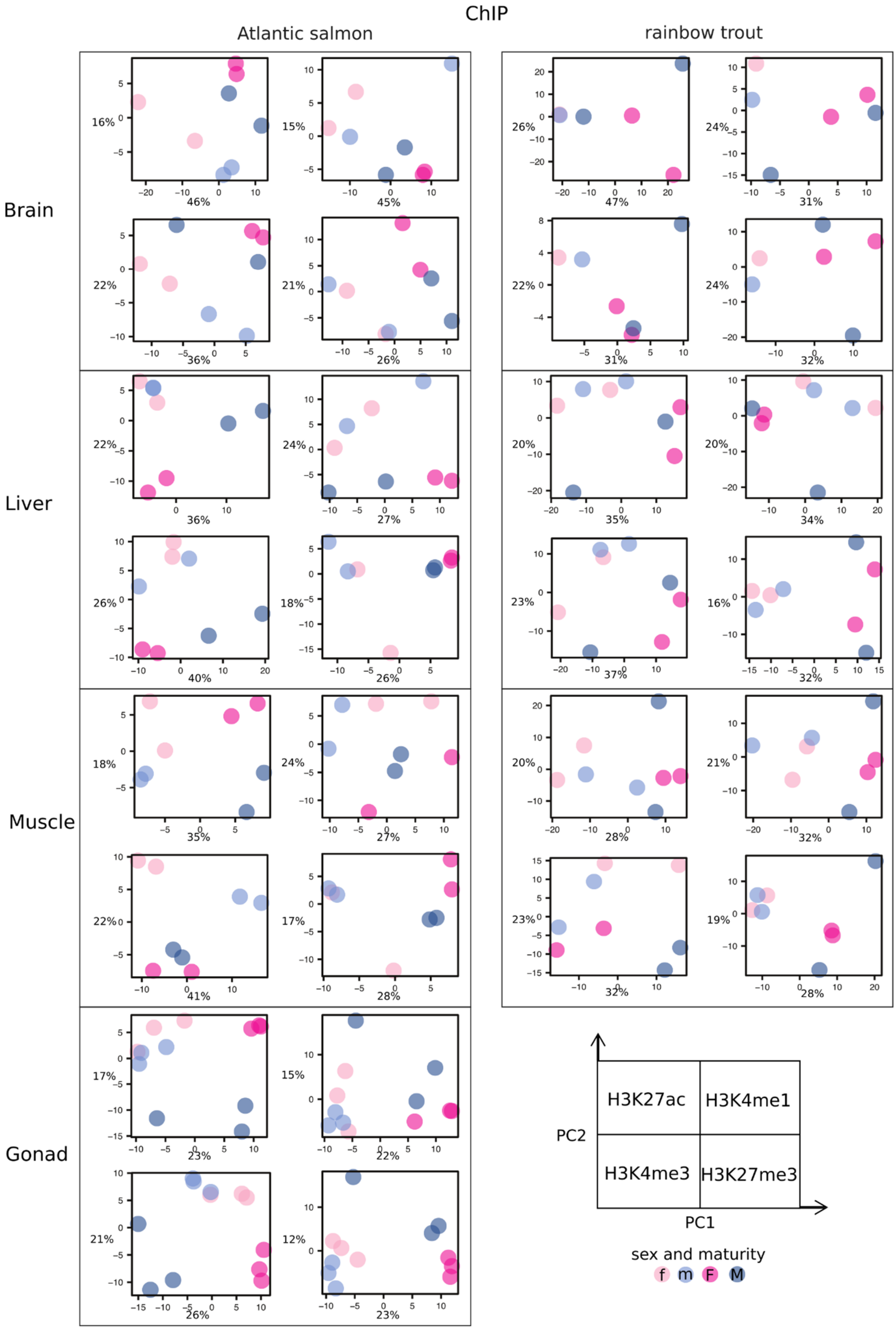

Supplementary Fig. 4. PCA of ChIP-seq datasets for BodyMap from salmon and trout. The key at the bottom right highlights different symbols used for sexes in BodyMap (tissues separated into immature = lowercase 'f' or 'm' and mature = uppercase 'F' and 'M'). The proportion of variation explained by PC1 and PC2 is shown on each plot.

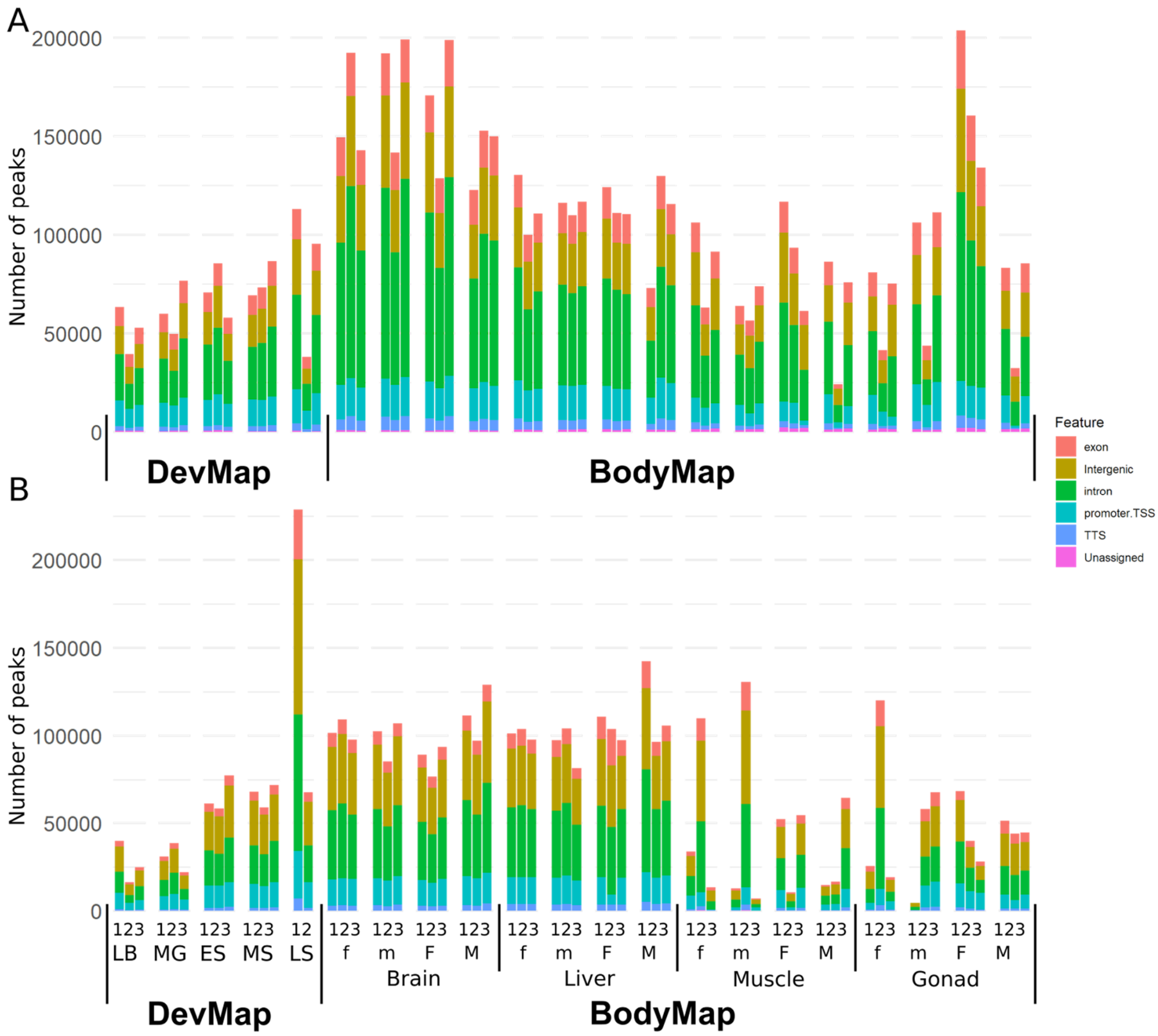

Supplementary Fig. 5. Overlap of robust open chromatin regions with annotated genome features. Data are shown for DevMap (stages: LB = late blastulation; MG = mid gastrulation; ES = early somitogenesis; MS = mid-somitogenesis; LS = late somitogenesis) and BodyMap (tissues separated into immature = lowercase 'm' or 'f' and mature = uppercase 'M' and 'F') samples for females and males, respectively) for salmon (**A**) and trout (**B**).

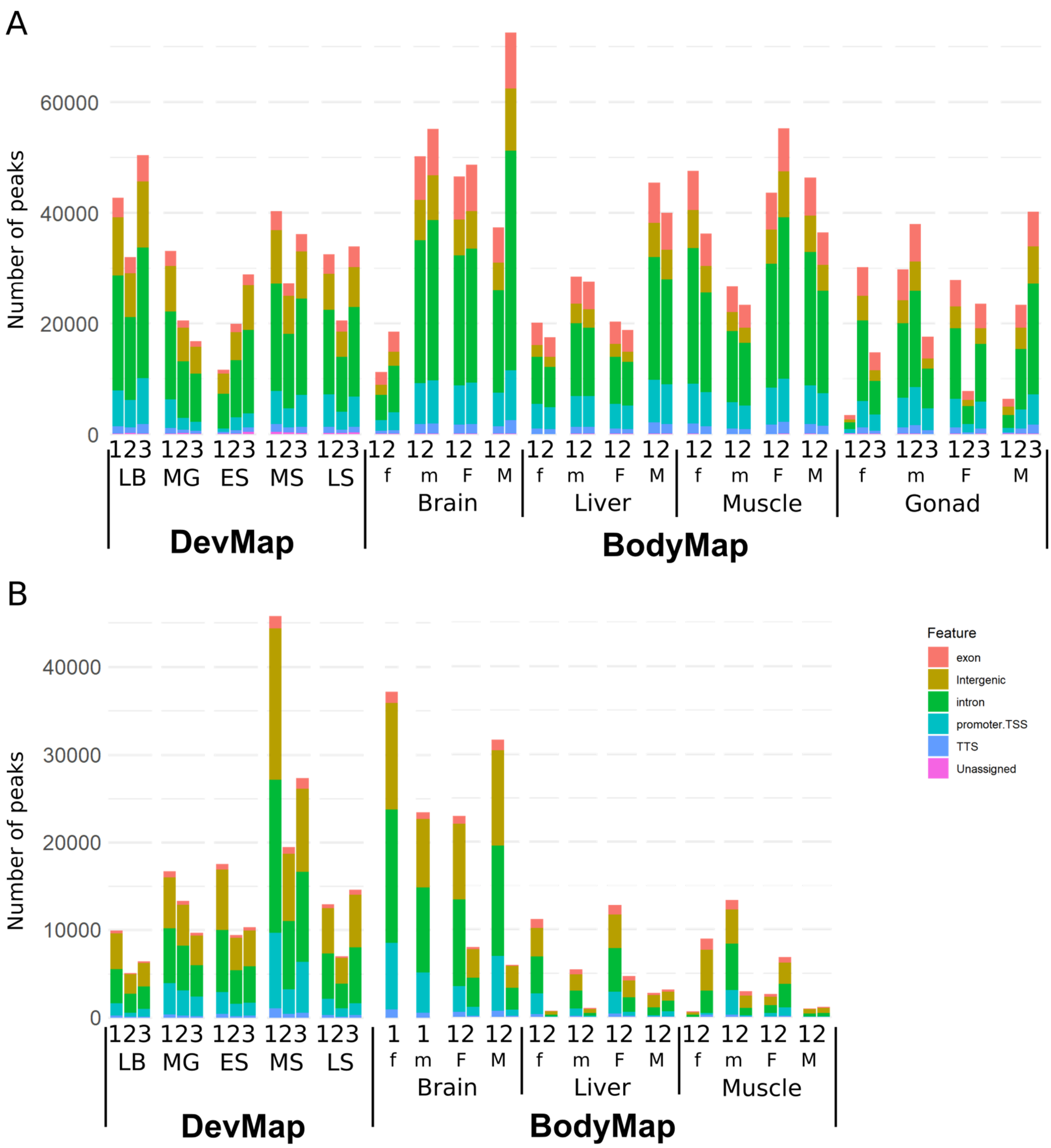

Supplementary Fig. 6. Overlap of ChIP-seq peaks with annotated genome features for H3K27ac. Data are shown for both DevMap (stages: LB = late blastulation; MG = mid gastrulation; ES = early somitogenesis; MS = mid-somitogenesis; LS = late somitogenesis) and BodyMap (tissues separated into immature = lowercase ‘f’ or ‘m’ and mature = uppercase ‘F’ and ‘M’) samples for females and males, respectively) for salmon (**A**) and trout (**B**).

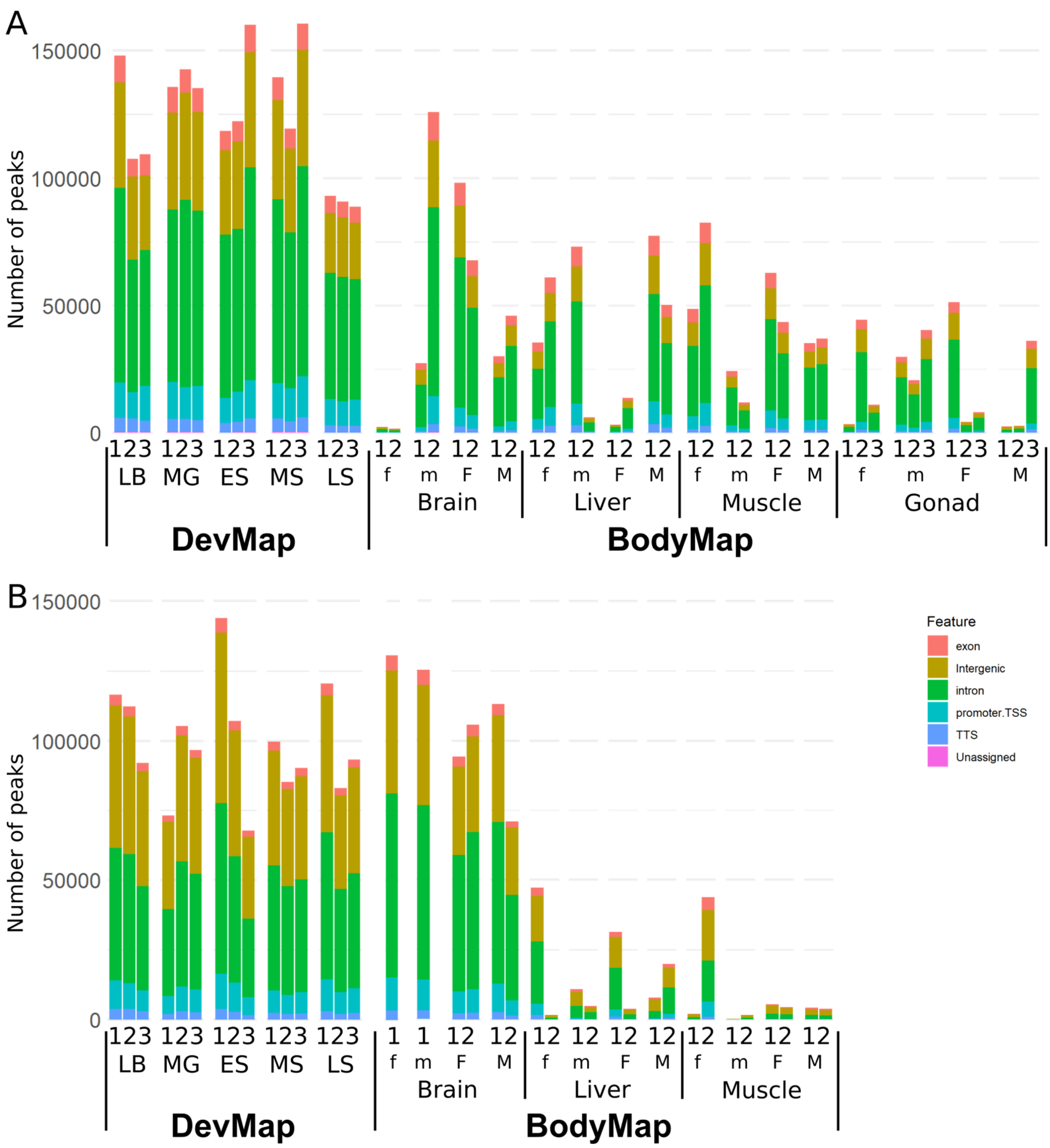

Supplementary Fig. 7. Overlap of ChIP-seq peaks with annotated genome features for H3K4me1. Data are shown for DevMap (stages: LB = late blastulation; MG = mid gastrulation; ES = early somitogenesis; MS = mid-somitogenesis; LS = late somitogenesis) and BodyMap (tissues separated into immature = lowercase ‘f’ or ‘m’ and mature = uppercase ‘F’ and ‘M’) samples for females and males, respectively) for salmon (**A**) and trout (**B**).

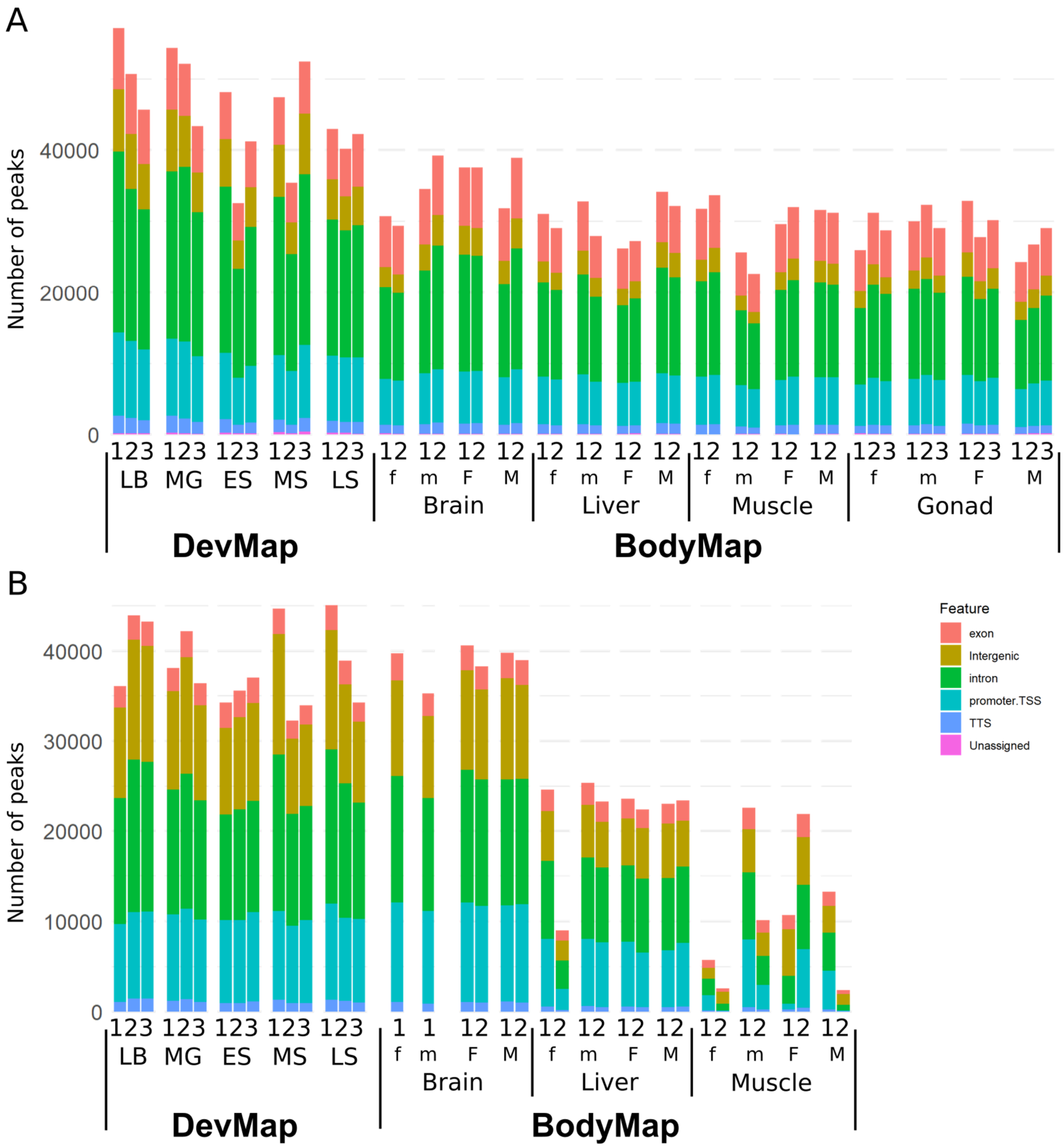

Supplementary Fig. 8. Overlap of ChIP-seq peaks with annotated genome features for H3K4me3. Data are shown for DevMap (stages: LB = late blastulation; MG = mid gastrulation; ES = early somitogenesis; MS = mid-somitogenesis; LS = late somitogenesis) and BodyMap (tissues separated into immature = lowercase 'f' or 'm' and mature = uppercase 'F' and 'M') samples for females and males, respectively) for salmon (**A**) and trout (**B**).

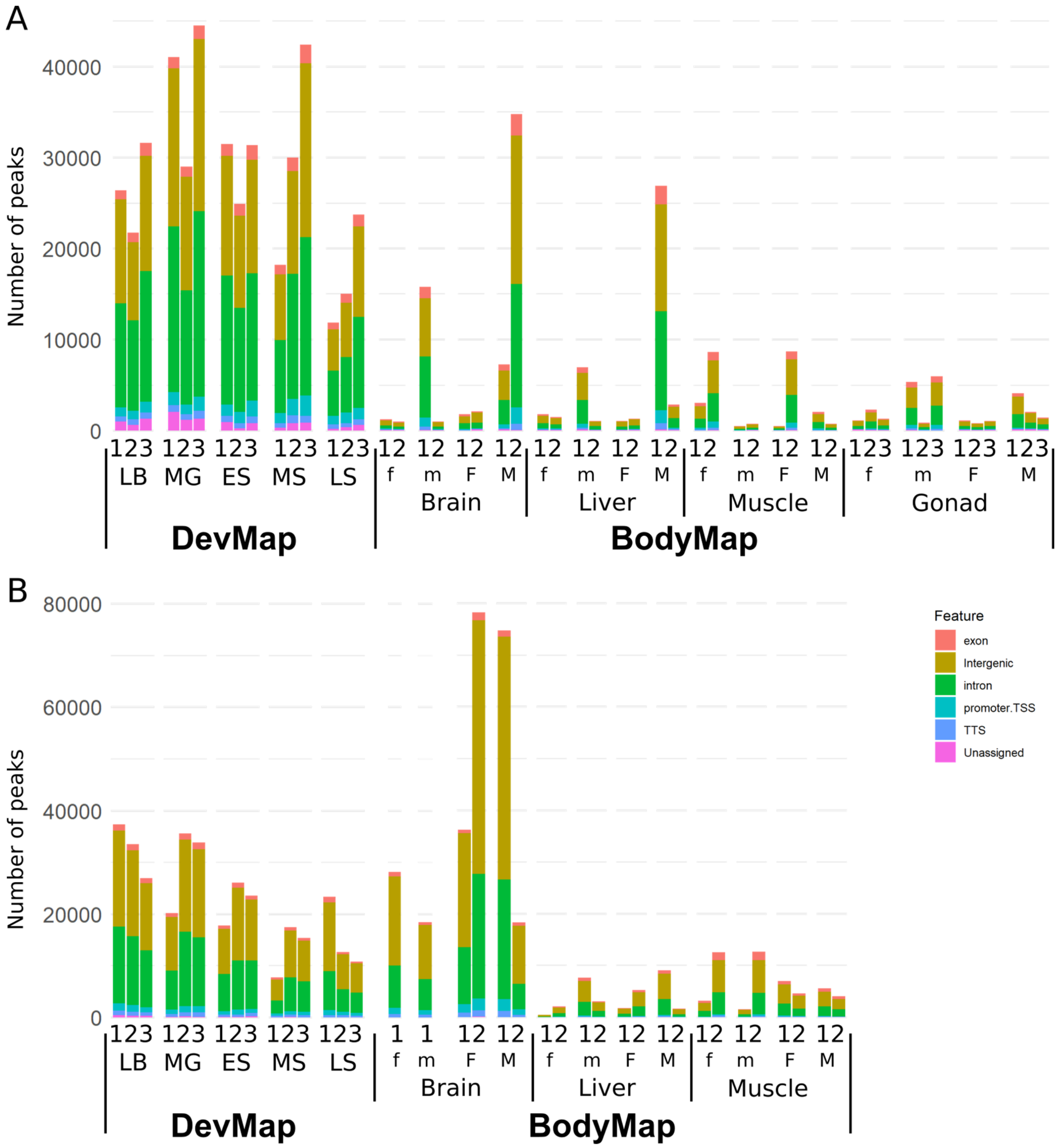

Supplementary Fig. 9. Overlap of ChIP-seq peaks with annotated genome features for H3K27me3. Data are shown for DevMap (stages: LB = late blastulation; MG = mid gastrulation; ES = early somitogenesis; MS = mid-somitogenesis; LS = late somitogenesis) and BodyMap (tissues separated into immature = lowercase ‘f’ or ‘m’ and mature = uppercase ‘F’ and ‘M’) samples for females and males, respectively) for salmon (**A**) and trout (**B**).

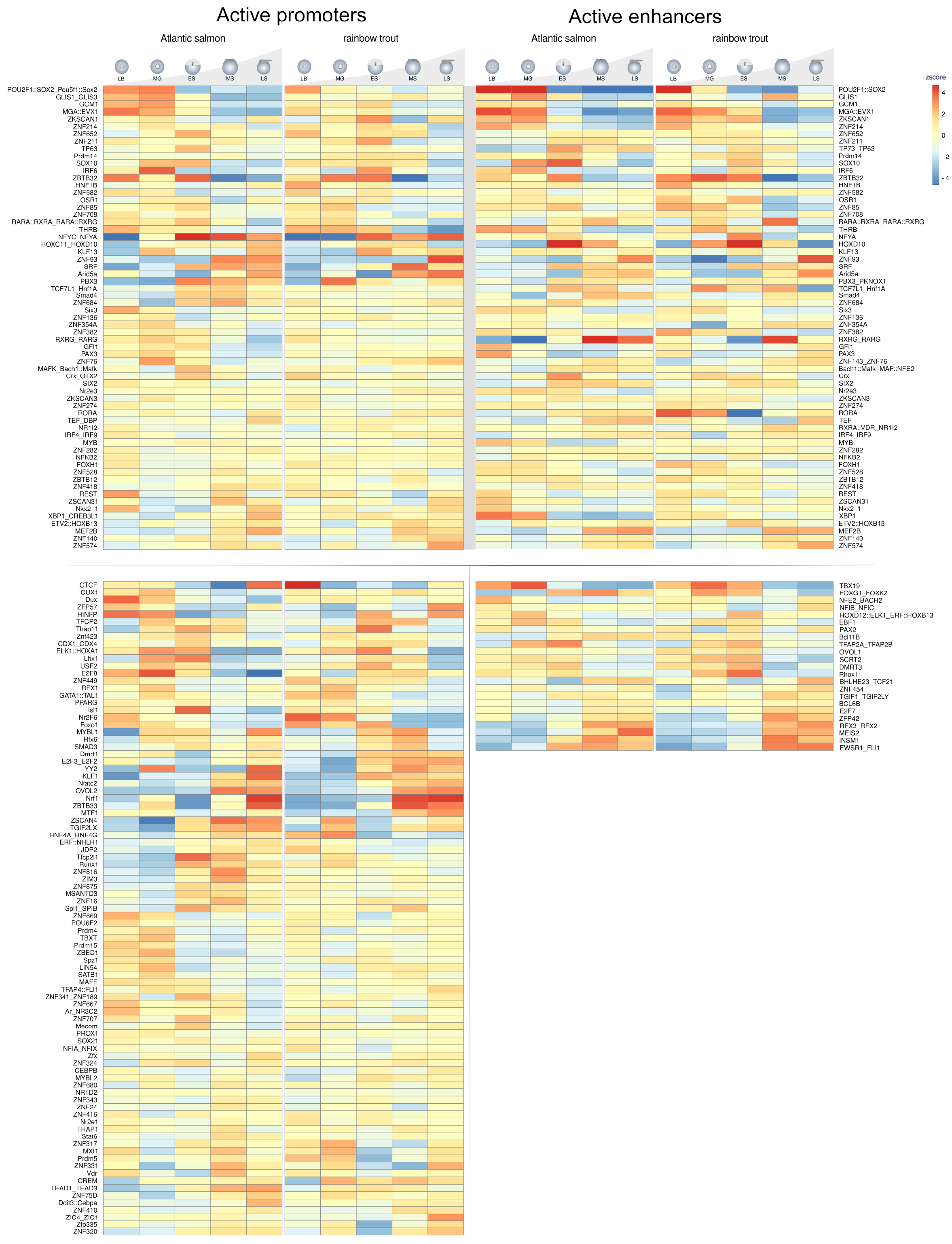

Supplementary Fig. 10. Transcription factor binding sites (TFBS) enrichment analysis across DevMap stages in salmon and trout. Enrichment of TFBSs within robust open chromatin regions annotated by ChromHMM as active promoters (left panel) or enhancers (right panel). TFBSs showing significant differences across stages are shown on a heatmap, with colours representing z-score values to visualize relative overrepresentation or underrepresentation according to the key on the right side. Supplementary Text 5 includes a summary of key results.

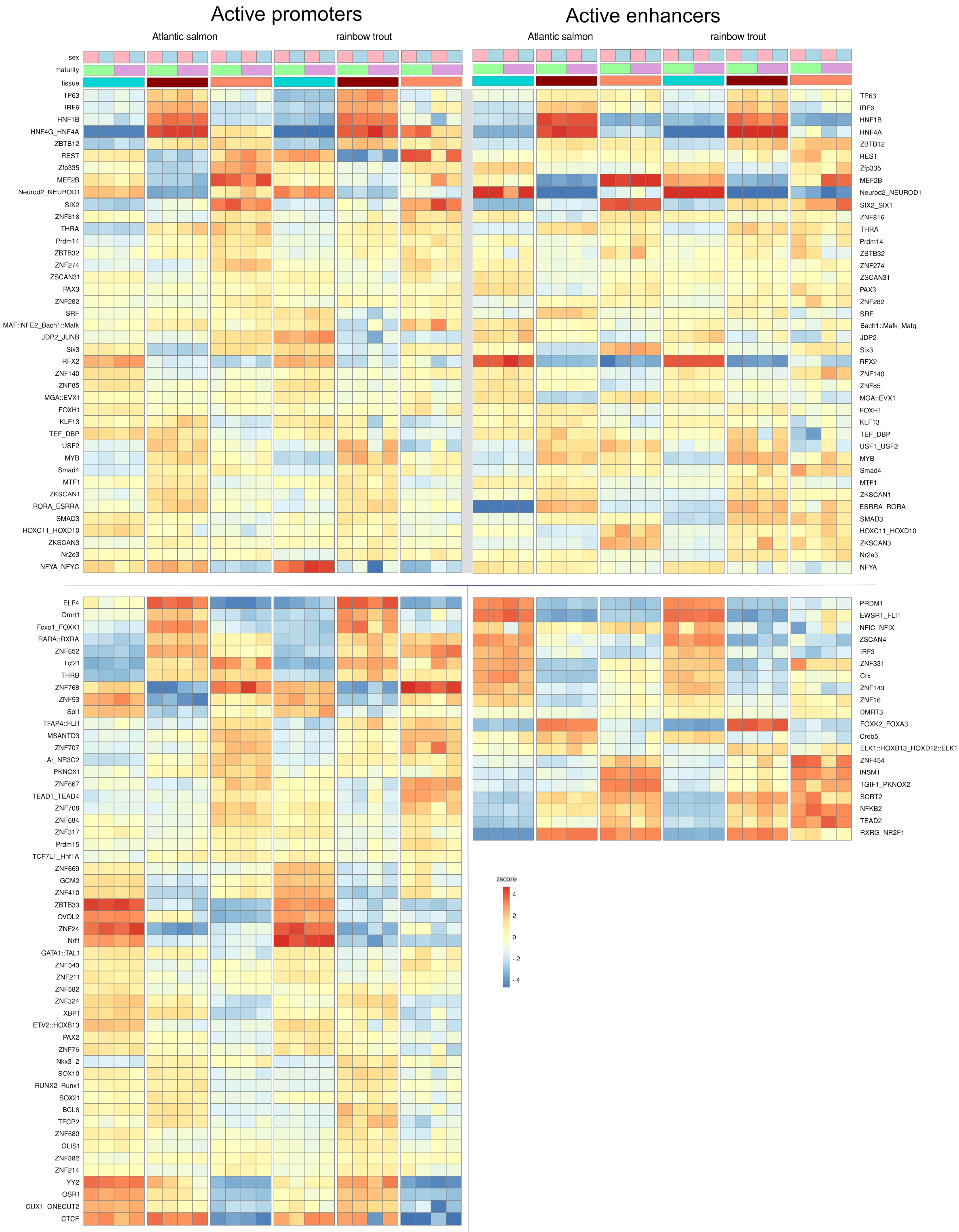

Supplementary Fig. 11. TFBS enrichment analysis across BodyMap tissues in salmon and trout. Enrichment of TFBSs within robust open chromatin regions annotated by ChromHMM as active promoters (left panel) or enhancers (right panel). TFBSs showing significant differences across stages are shown on a heatmap, with colours representing z-score values to visualize relative overrepresentation or underrepresentation according to the key on the right side. Supplementary Text 5 includes a summary of key results.

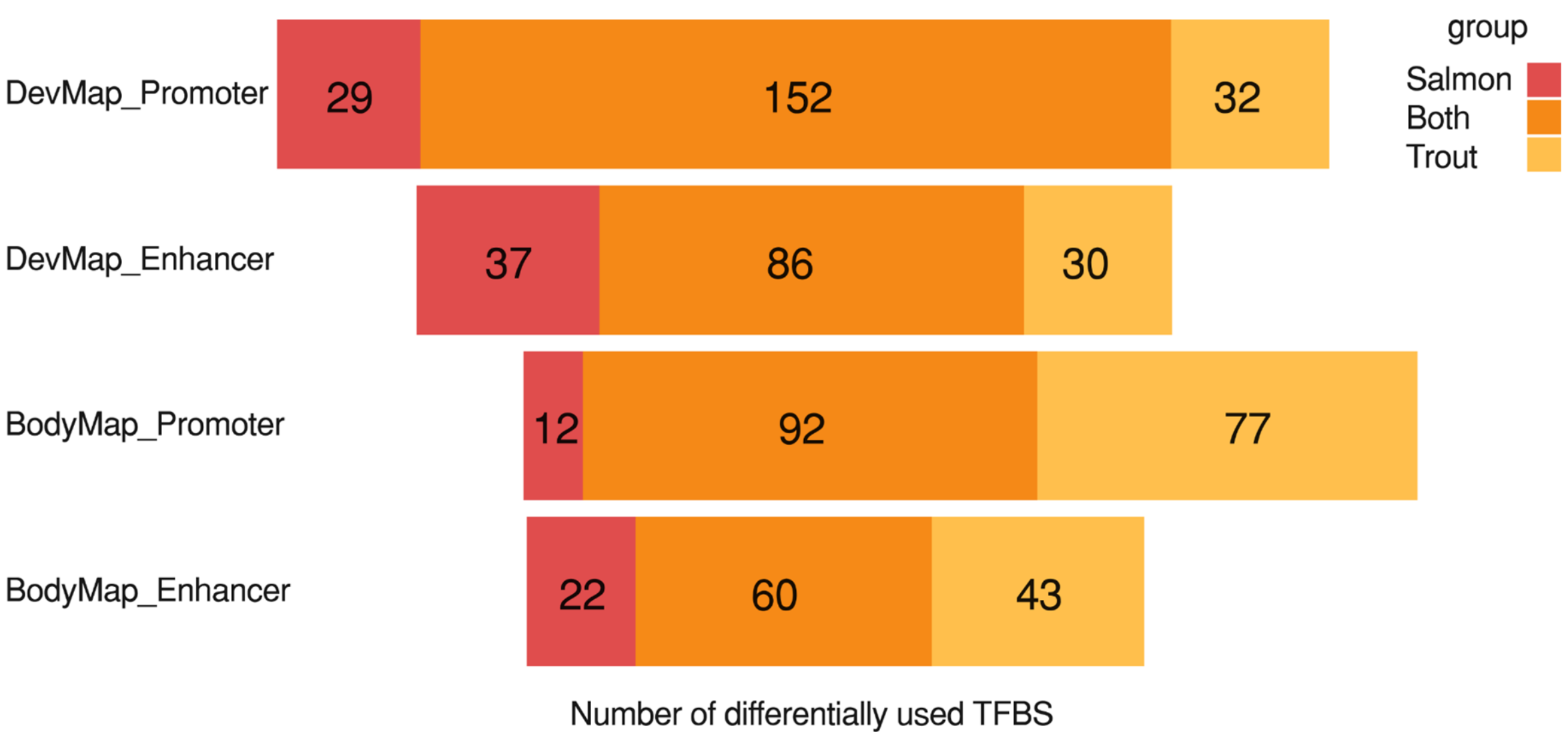

Supplementary Fig. 12. Overlap between enriched TFBS for salmon and trout in DevMap and BodyMap sample types. For DevMap or BodyMap, the number of overlapping TFBS found enriched within robust open chromatin regions annotated by ChromHMM as active TSS or active enhancers for both species is presented in orange, while the number of unique TFBS is depicted in red for salmon and in yellow for trout. Supplementary Text 5 includes a summary of key results.

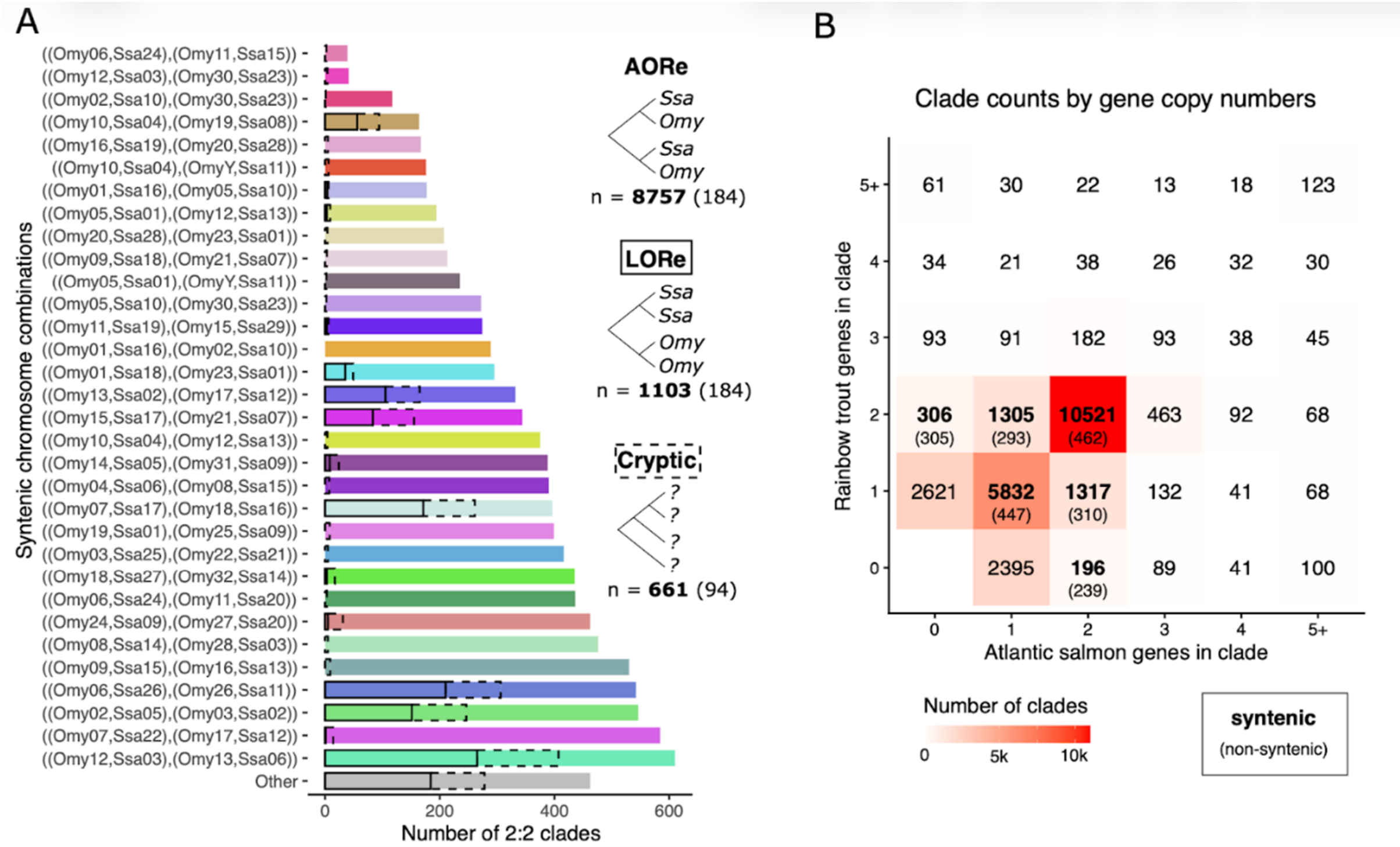

Supplementary Fig. 13. Homology prediction capturing ohnologs (within) and orthologs (between) for the salmon and trout genomes (see Methods). (**A**) Identification of homologous/syntenic chromosome regions represented by genes captured within 2:2 clades in phylogenetic analysis (2:2 clades are those where both salmonid species retain two duplicated genes). Inserts show the number of AORe (i.e., Early rediploidization; ohnolog divergence shared by both salmonid species), LORe (lineage-specific rediploidization; ohnolog divergence post-speciation of salmon and trout; also referred to in this study as Late rediploidization) and other (cryptic) phylogenetic topologies. Numbers in parenthesis represent clades not matching syntenic chromosomes, i.e "Other". (**B**) Number of clades capturing different numbers of genes in the two salmonid species. The bold numbers show how many of the clades capture genes located within syntenic chromosomes, as expected by a WGD event, i.e., representing our definition of high-confidence ohnologs (numbers in parenthesis represent clades not in syntenic chromosomes). The plot highlights the dominance of 2:2 clades (i.e., ohnolog pairs retained in both species), 1:1 clades (e.g., resulting from one ohnolog in a pair being lost ancestrally to salmon and trout), as well as 2:1 clades (e.g., where one ohnolog was lost in one species, and the other species retains both ohnologs).

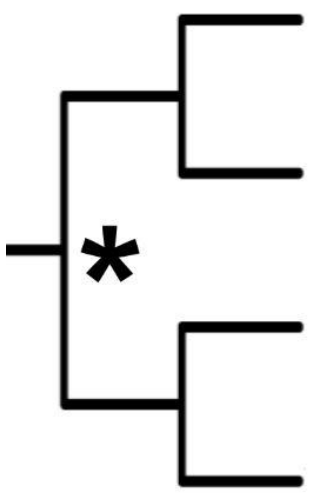

Ssa03:53,495,240 – 100,966,100			Ssa03:2,160,013 – 45,141,280		
**LORe**	**Ensembl**	**AQUA-FAANG**	**AORe**	**Ensembl**	**AQUA-FAANG**
Ssa03	47,623,004	47,470,893	*Ssa03*	43,083,082	42,981,345
Ssa06	26,638,611	25,604,383	*Omyk28*	26,638,736	26,837,948
Omyk12	23,530,163	26,143,366	*Ssa14*	9,597,080	15,716,672
Omyk13	23,137,724	25,833,768	*Omyk08*	9,204,883	14,845,254

Supplementary Fig. 14. Comparison of effectiveness of two whole genome alignment strategies for capture of duplicated regions from salmonid WGD. The table shows data derived from the alignment of a chromosome selected from the salmon genome that contains two distinct rediploidization histories [1,2] including a large LORe (Late rediploidization) region (Ssa03-06) that underwent rediploidization after the split of *Salmo* and *Oncorhynchus* and a large AORe (Early rediploidization) region that underwent rediploidization early in salmonid evolution (Ssa03-14). We compared the capture of these regions, along with their homologous regions in trout (Omyk12-13 and Omyk28-08) in two Cactus alignments (see Methods). The first was an alignment that was prepared by the Ensembl Genome Browser ("Ensembl") run using a standard approach that is not aware of WGD, and the second the alignment prepared in this study ("AQUA-FAANG") with our WGD-aware approach. The WGD-aware approach recovered around 37-38% more aligned sequence in AORe regions for both species, and 10-11% in LORe (Late rediploidization) regions.

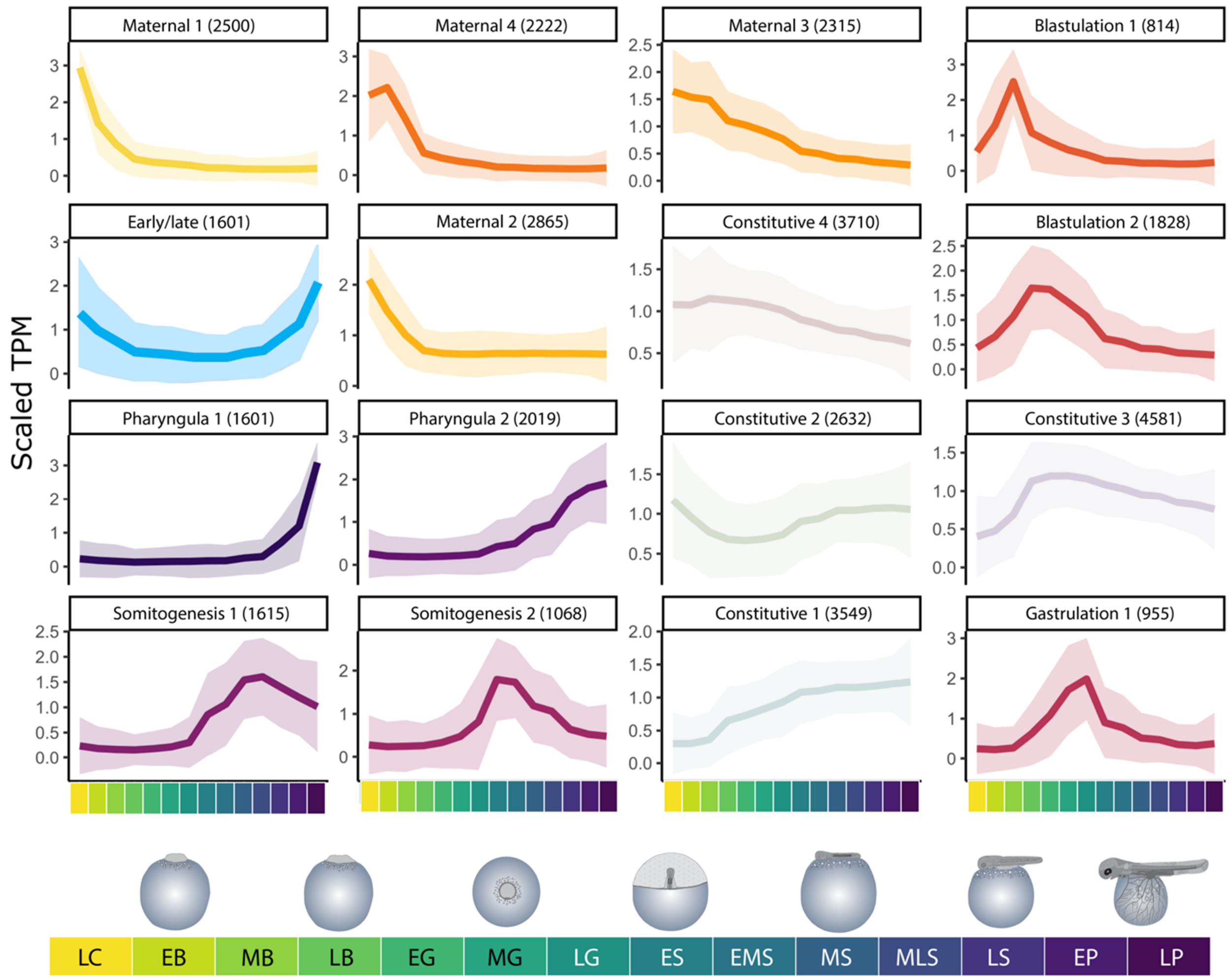

Supplementary Fig. 15. Salmon DevMap transcriptome SOM clusters. Each individual line plot represents one SOM cluster and shows average normalised gene expression, with the position on the panel assigned by the SOM algorithm based on the inferred topology of the data. The colours indicate the order of expression throughout embryogenesis, based on colours used in Fig. 2 in the main text. Colored areas around each line plot show 95% confidence intervals. The top of each line plot indicates the name assigned to each SOM cluster based on gene expression pattern, along with the number of genes captured per cluster. Stages are indicated using the colours shown in the bottom annotation. Images of embryos were adapted from [3], with permission from Springer Nature under License Number 6307121285453.

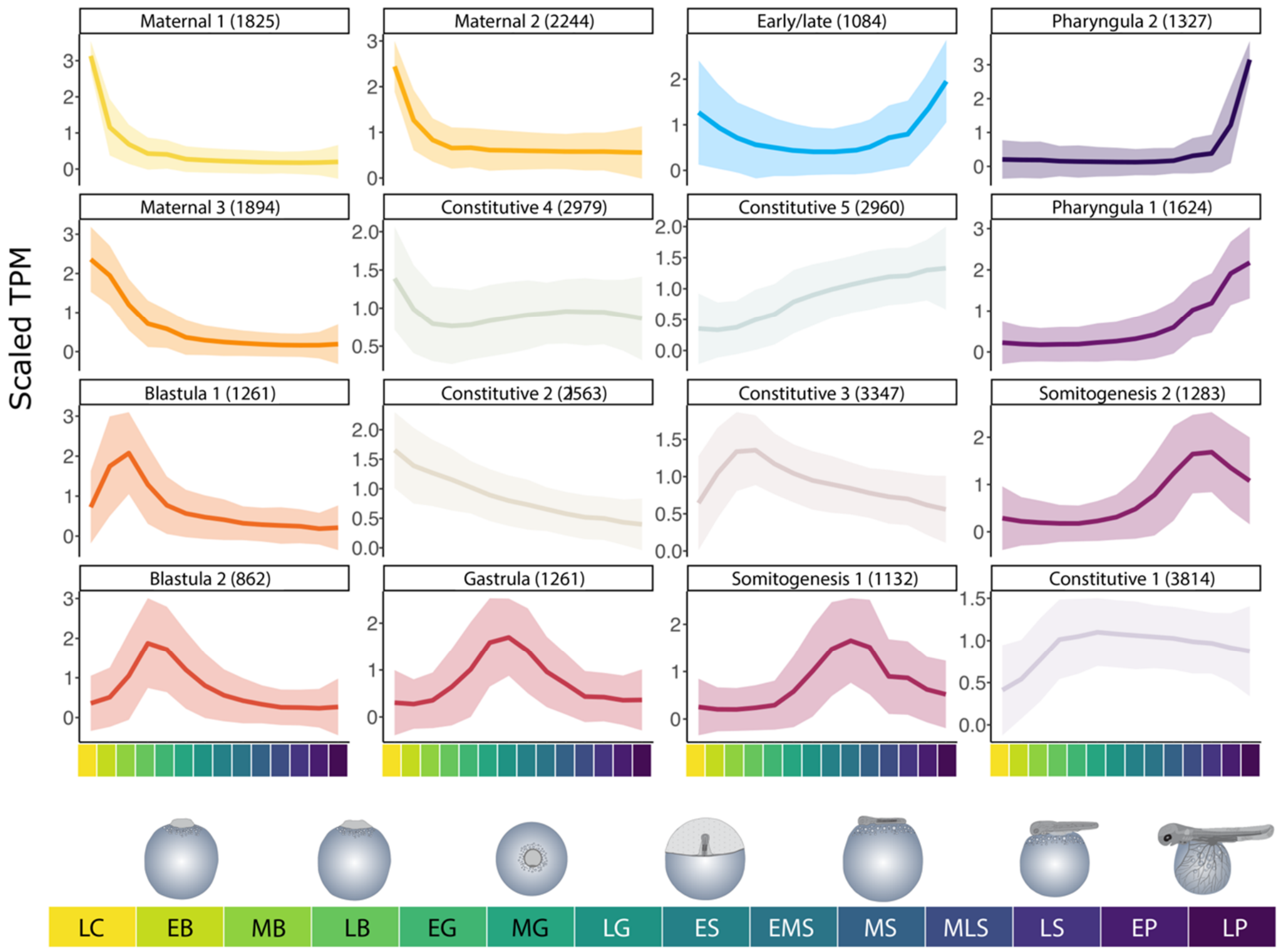

Supplementary Fig. 16. Trout DevMap transcriptome SOM clusters. Each individual line plot represents one SOM cluster and shows average normalised gene expression, with the position on the panel assigned by the SOM algorithm based on the inferred topology of the data. The colours indicate the order of expression throughout embryogenesis, based on colours used in Fig. 2 in the main text. Colored areas around each line plot show 95% confidence intervals. The top of each line plot indicates the name assigned to each SOM cluster based on gene expression pattern, along with the number of genes captured per cluster. Stages are indicated using the colours shown in the bottom annotation. Images of embryos were adapted from [3], with permission from Springer Nature under License Number 6307121285453.

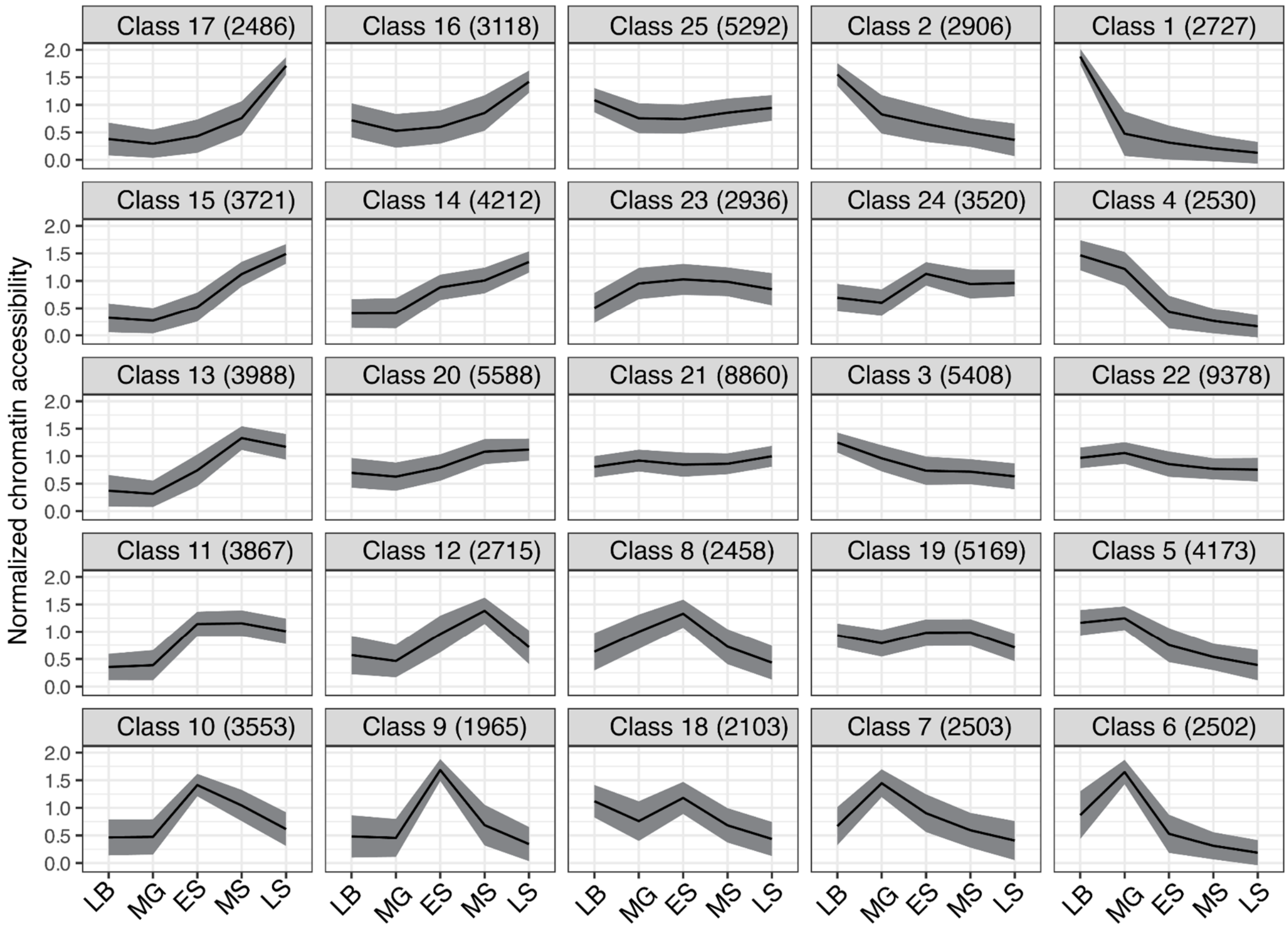

Supplementary Fig. 17. Salmon DevMap chromatin openness SOM clusters. Each individual line plot represents one SOM cluster and shows average normalised chromatin openness/accessibility, with the position on the panel assigned by the SOM algorithm based on the inferred topology of the data. Shaded areas around each line plot show 95% confidence intervals. The top of each line plot indicates the SOM class number, along with the number of robust open chromatin regions captured per class. Stages are indicated using abbreviations as x-axis labels: LB, Late blastula; MG, Mid gastrula; ES, Early somitogenesis; MS, Mid somitogenesis; LS, Late somitogenesis.

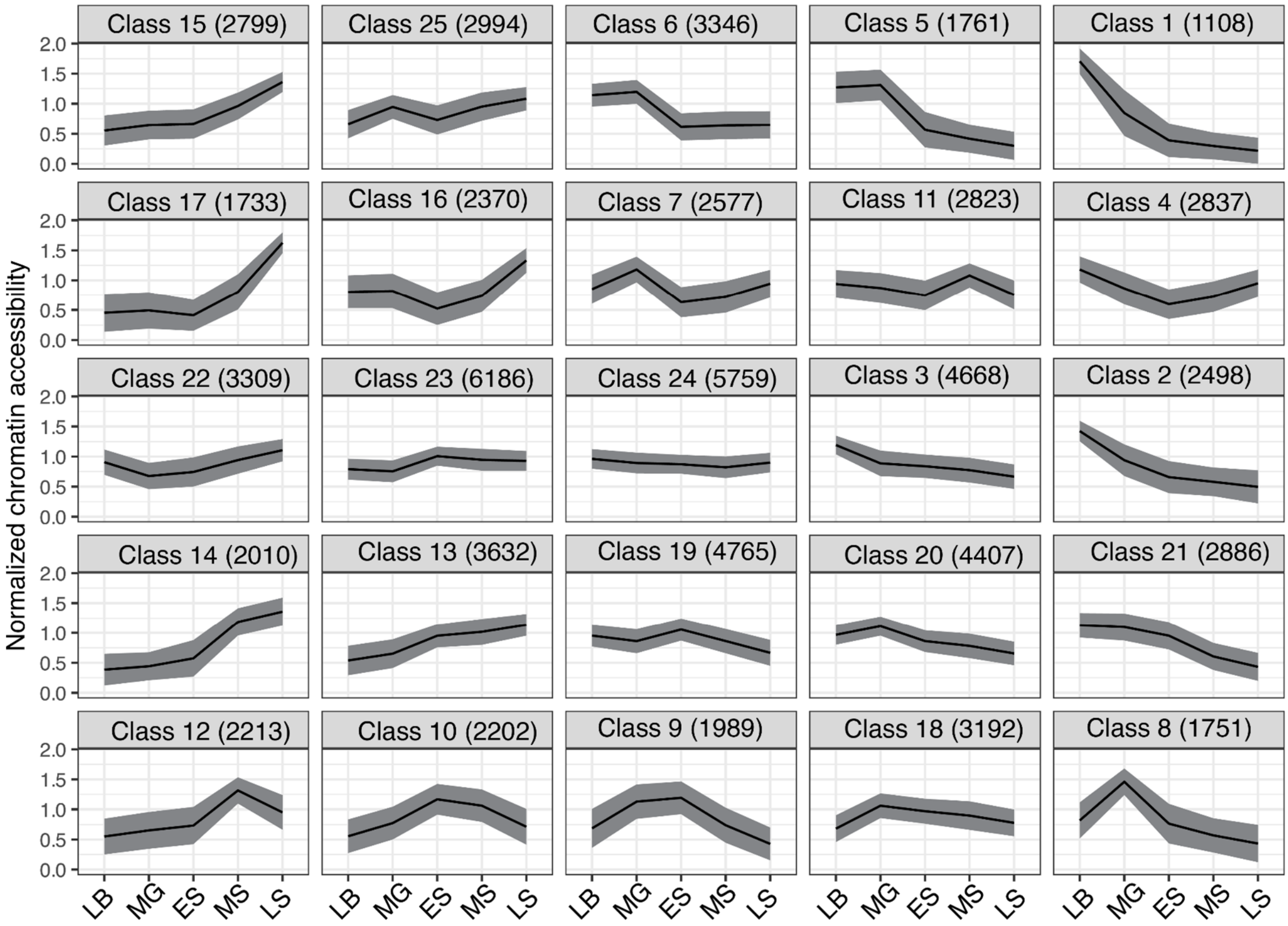

Supplementary Fig. 18. Trout DevMap chromatin openness SOM clustering. Other details are as per the Supplementary Fig. 17 legend.

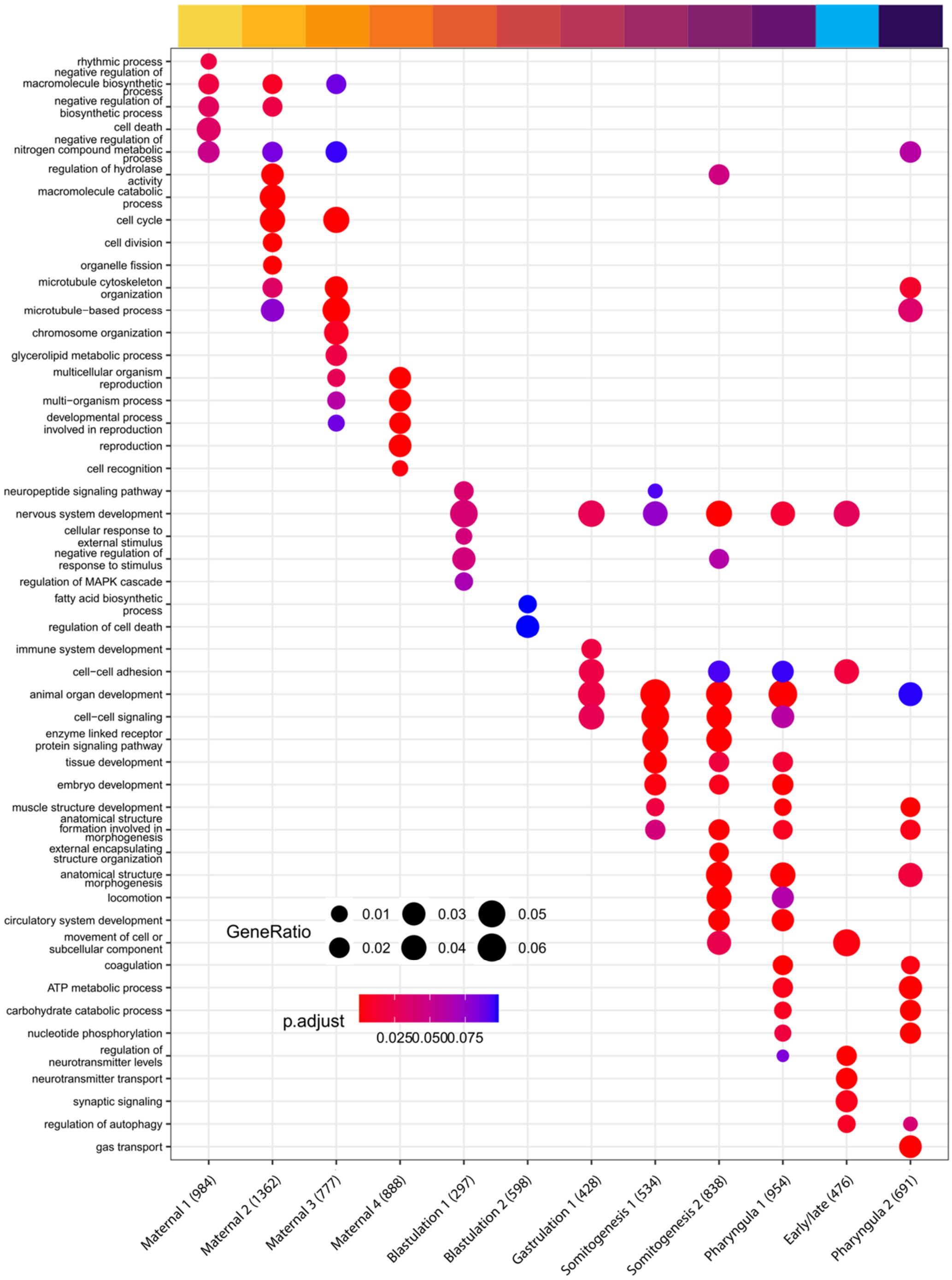

Supplementary Fig. 19. Enriched gene ontology (GO) terms for DevMap transcriptome of salmon. The dot plot compares the most enriched GO terms across SOM clusters shown on the x-axis (shown in Supplementary Fig. 15), restricted to 'Biological Process' terms. Each cluster shows a maximum of five distinct terms, based on FDR adjusted *p*-value, and additional terms also enriched in other clusters. The y-axis provides GO terms, with the size of each dot indicating gene ratio and colours indicating FDR adjusted *p*-value according to the shown scales. Numbers in labels indicate the number of genes in the SOM cluster matched to at least one GO term. The cluster named "early/late" was not included in Fig. 2 (main text). GO term enrichment was tested by one-sided over-representation analysis (hypergeometric test, clusterProfiler enrichGO), with *p* values adjusted across terms by the Benjamini-Hochberg method.

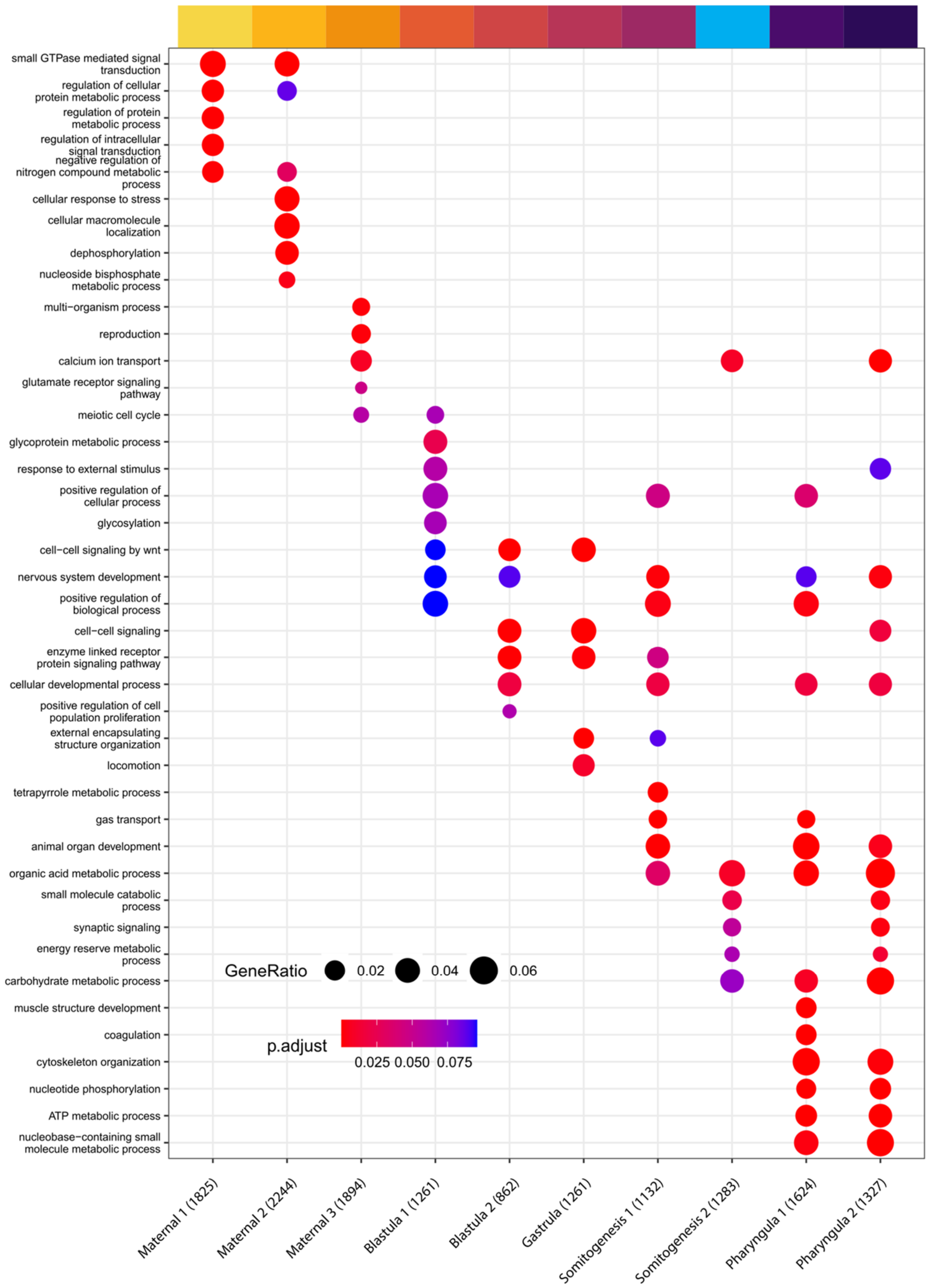

Supplementary Fig. 20. Enriched gene ontology (GO) terms for DevMap transcriptome of trout. The dot plot compares the most enriched GO terms across SOM clusters shown on the x-axis (shown in Supplementary Fig. 16), restricted to 'Biological Process' terms. Each cluster shows a maximum of five distinct terms, based on FDR adjusted *p*-value, and additional terms also enriched in other clusters. The y-axis provides GO terms, with the size of each dot indicating gene ratio and colours indicating FDR adjusted *p*-value according to the shown scales. Numbers in labels indicate the number of genes in the SOM cluster matched to at least one GO term. GO term enrichment was tested by one-sided over-representation analysis (hypergeometric test, clusterProfiler enrichGO), with *p* values adjusted across terms by the Benjamini-Hochberg method.

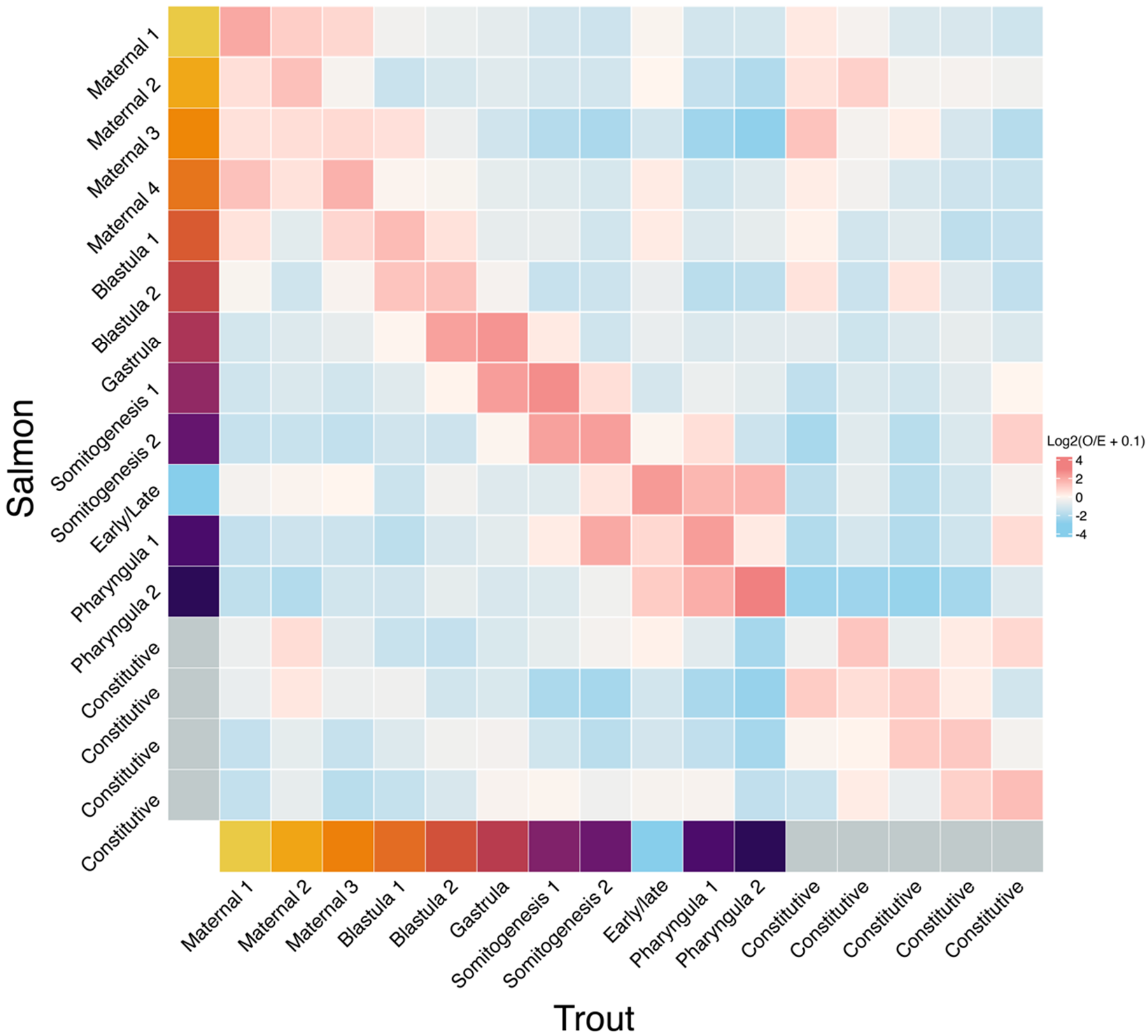

Supplementary Fig. 21. Matrix of observed to expected ratio of orthologs shared across all combinations of salmon and trout DevMap SOM clusters. Expected values were calculated as the mean of 10,000 random distributions of trout genes across SOM clusters. Colours represent log-2 of observed to expected ratio, such that positive and negative values represent ortholog enrichment and depletion, respectively. Axis labels indicate SOM cluster names, as shown in Supplementary Fig. 15 (salmon) and Supplementary Fig. 16 (trout).

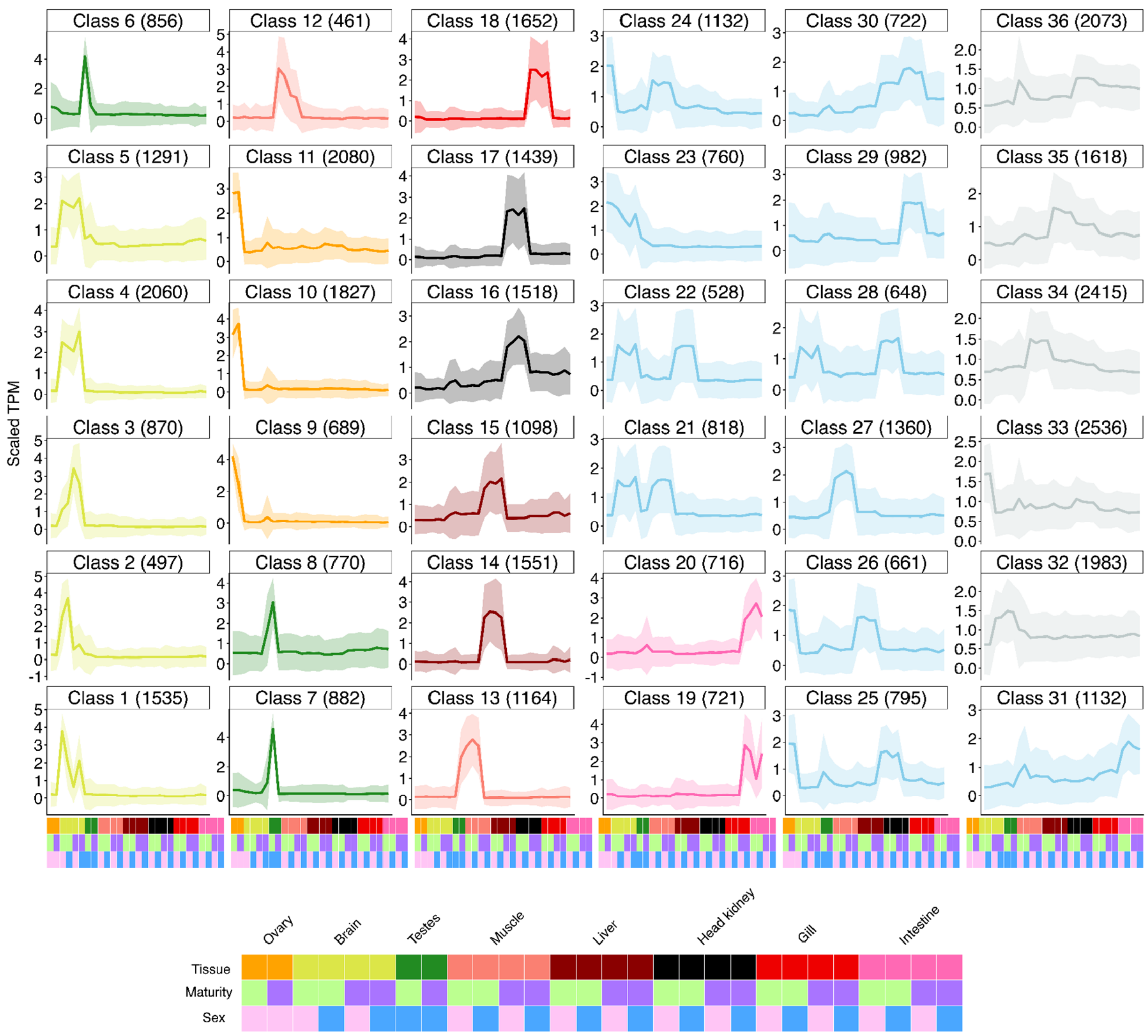

Supplementary Fig. 22. Salmon BodyMap transcriptome clusters. Each individual line plot represents one SOM cluster and shows average normalised gene expression, with the position on the panel assigned by the SOM algorithm based on the inferred topology of the data. The colours indicate the tissue-specific expression of each cluster (based on colours used in Extended Data Fig. 4), with additional clusters presented here (In grey, constitutive clusters, and in light blue, clusters expressed across several tissues). Coloured areas around each line plot show 95% confidence intervals. The top of each line plot indicates the number of genes captured per cluster. Each sample is indicated using the colours shown in the bottom annotation.

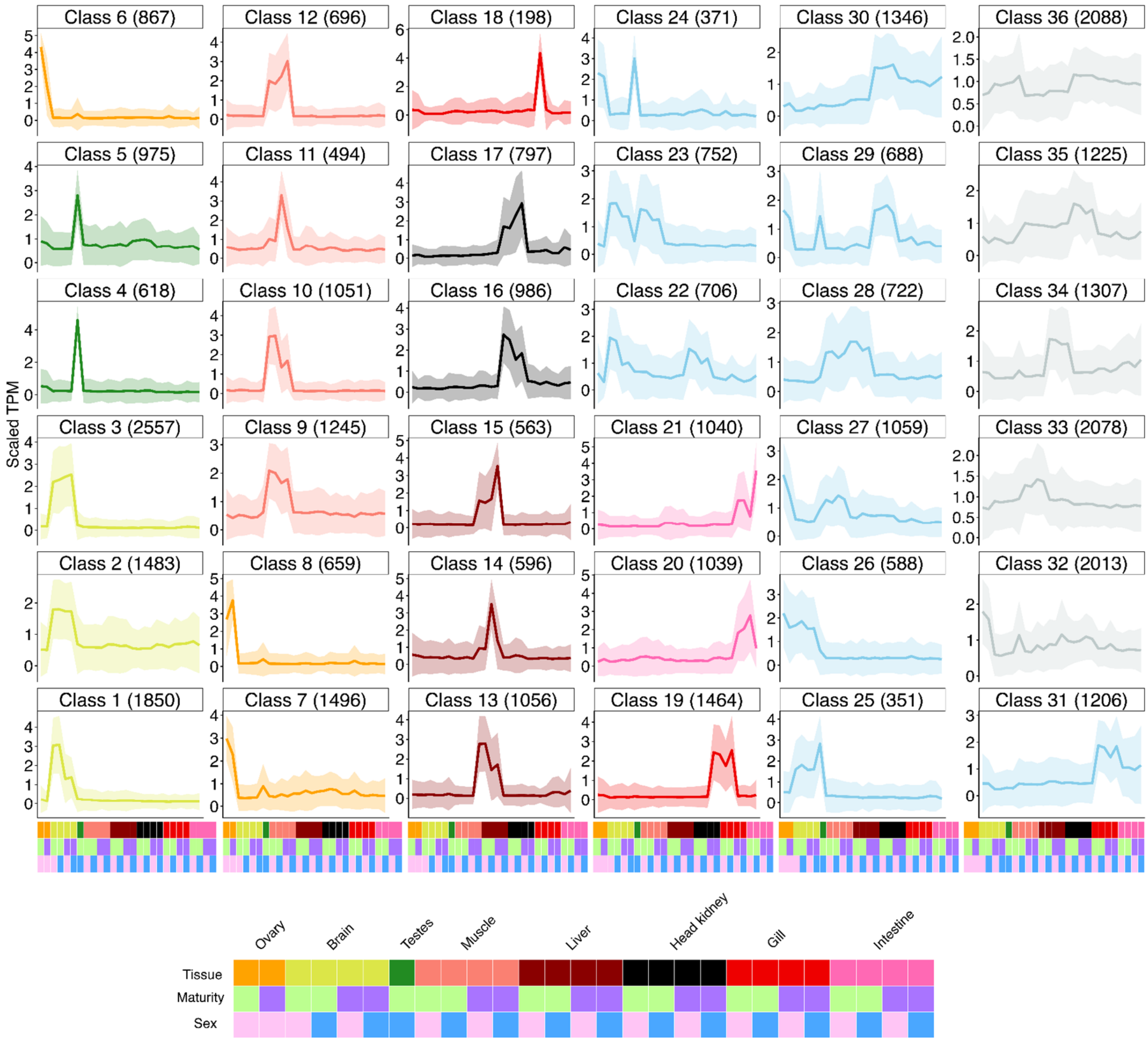

Supplementary Fig. 23. Trout BodyMap transcriptome clusters. Other details are as per the Supplementary Fig. 22 legend.

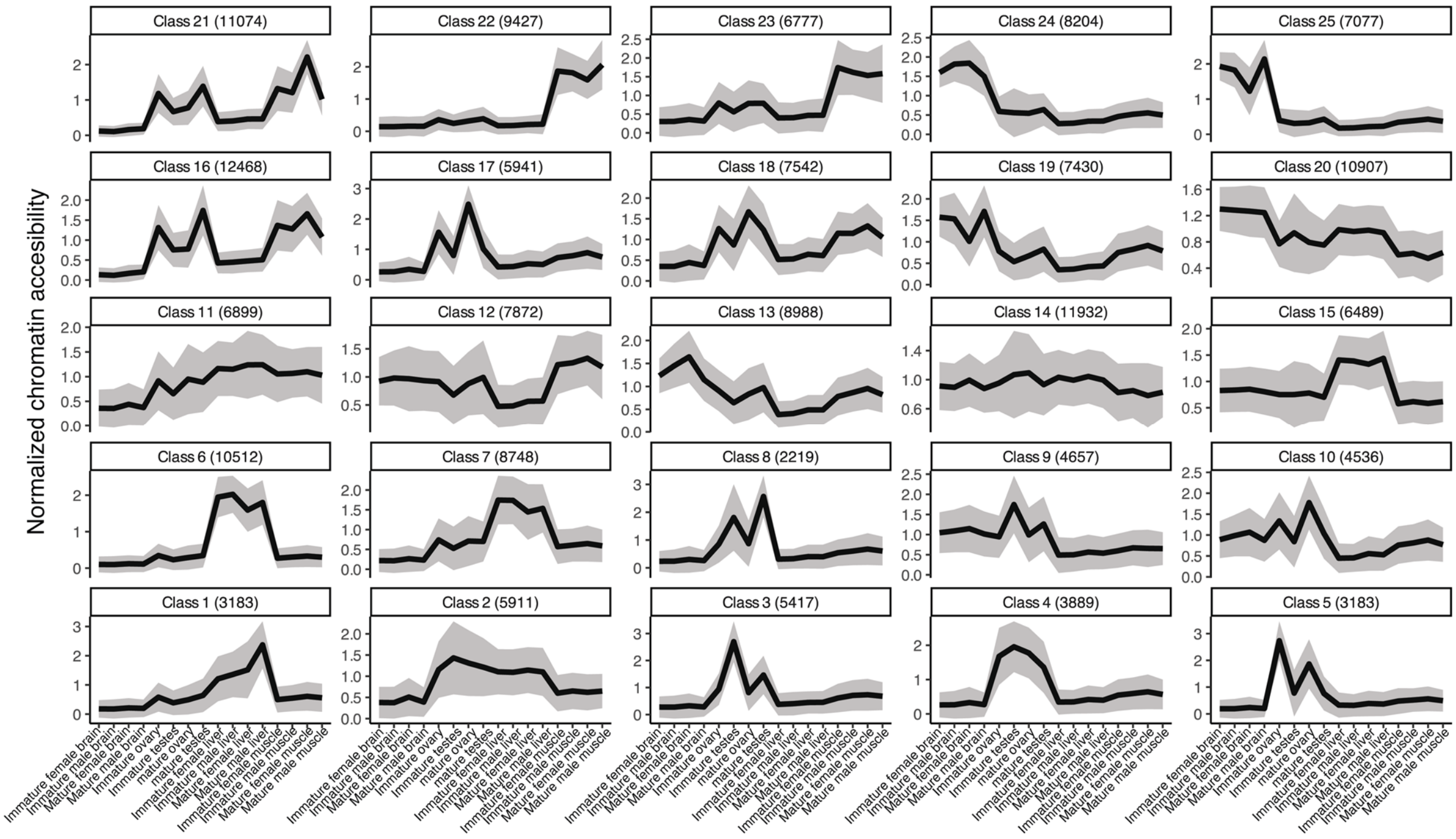

Supplementary Fig. 24. Salmon BodyMap chromatin openness SOM clusters. Each individual line plot represents one SOM cluster and shows average normalised chromatin openness, with the position on the panel assigned by the SOM algorithm based on the inferred topology of the data. Shaded areas around each line plot show 95% confidence intervals. The top of each line plot indicates the SOM class number, along with the number of robust open chromatin regions captured per class. The x-axis labels represent sample information, including tissue type, sex and stage of maturity

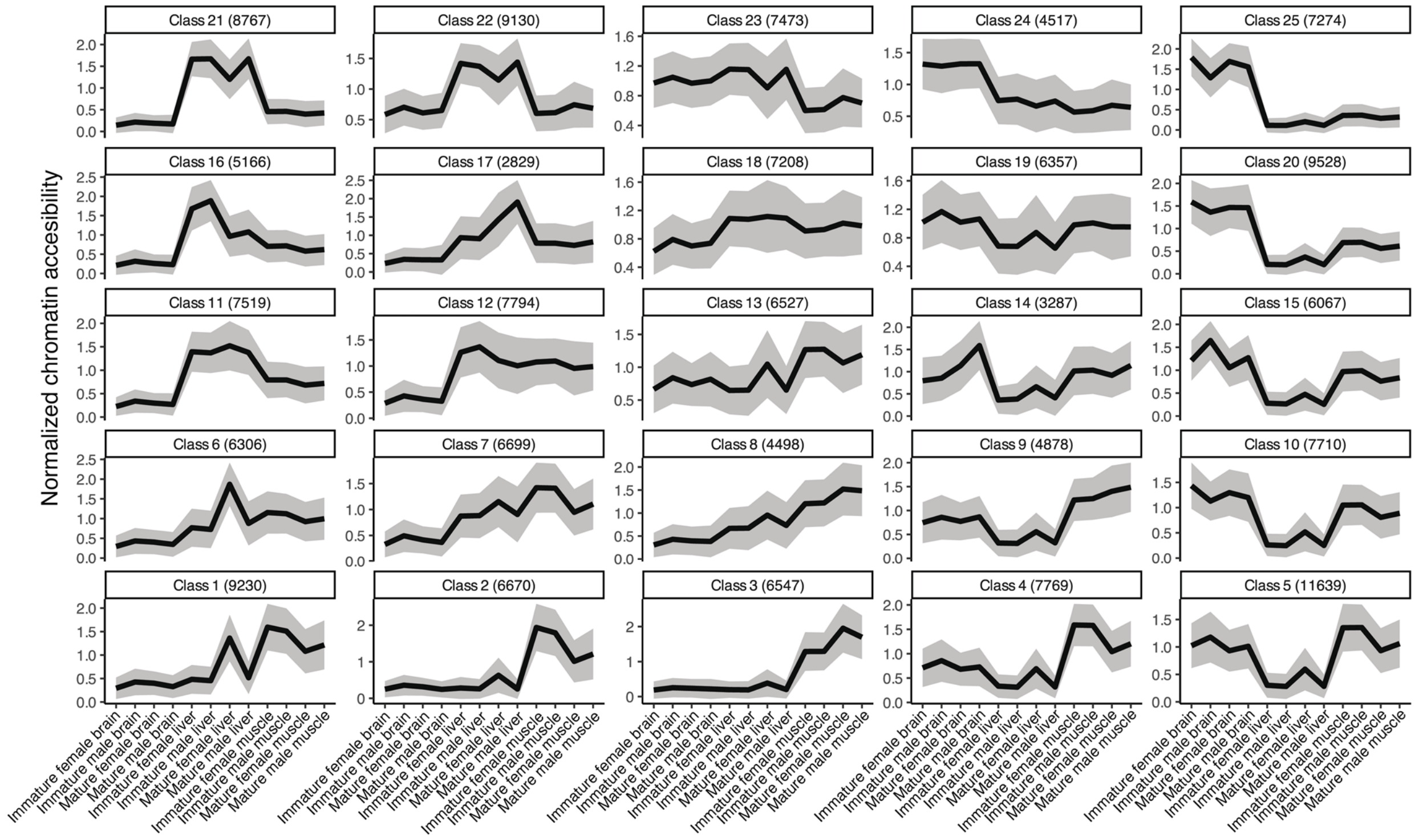

Supplementary Fig. 25. Trout BodyMap chromatin openness SOM clusters. Other details are as per the Supplementary Fig. 24 legend.

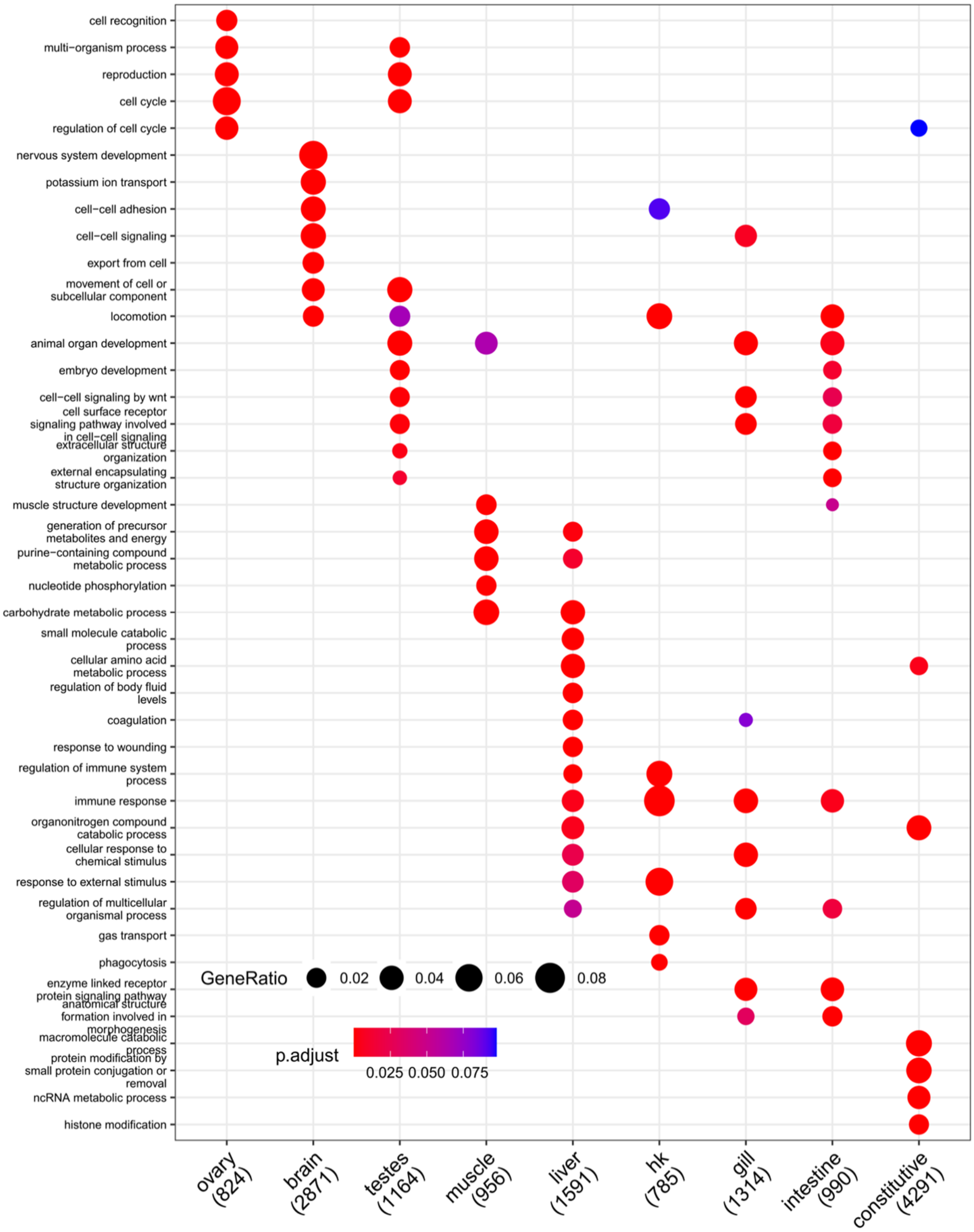

Supplementary Fig. 26. Enriched gene ontology (GO) terms for BodyMap transcriptome of salmon. The dot plot compares the most enriched GO terms across SOM clusters shown on the x-axis (shown in Supplementary Fig. 22), restricted to 'Biological Process' terms. Each cluster shows a maximum of five distinct terms, based on FDR adjusted p-value, and additional terms also enriched in other clusters. The y-axis provides GO terms, with the size of each dot indicating gene ratio and colours indicating FDR adjusted p-value according to the shown scales. Numbers in labels indicate the number of genes in the SOM cluster matched to at least one GO term. 'hk' = head kidney, 'intestine' = distal intestine. GO term enrichment was tested by one-sided over-representation analysis (hypergeometric test, clusterProfiler enrichGO), with *p* values adjusted across terms by the Benjamini-Hochberg method.

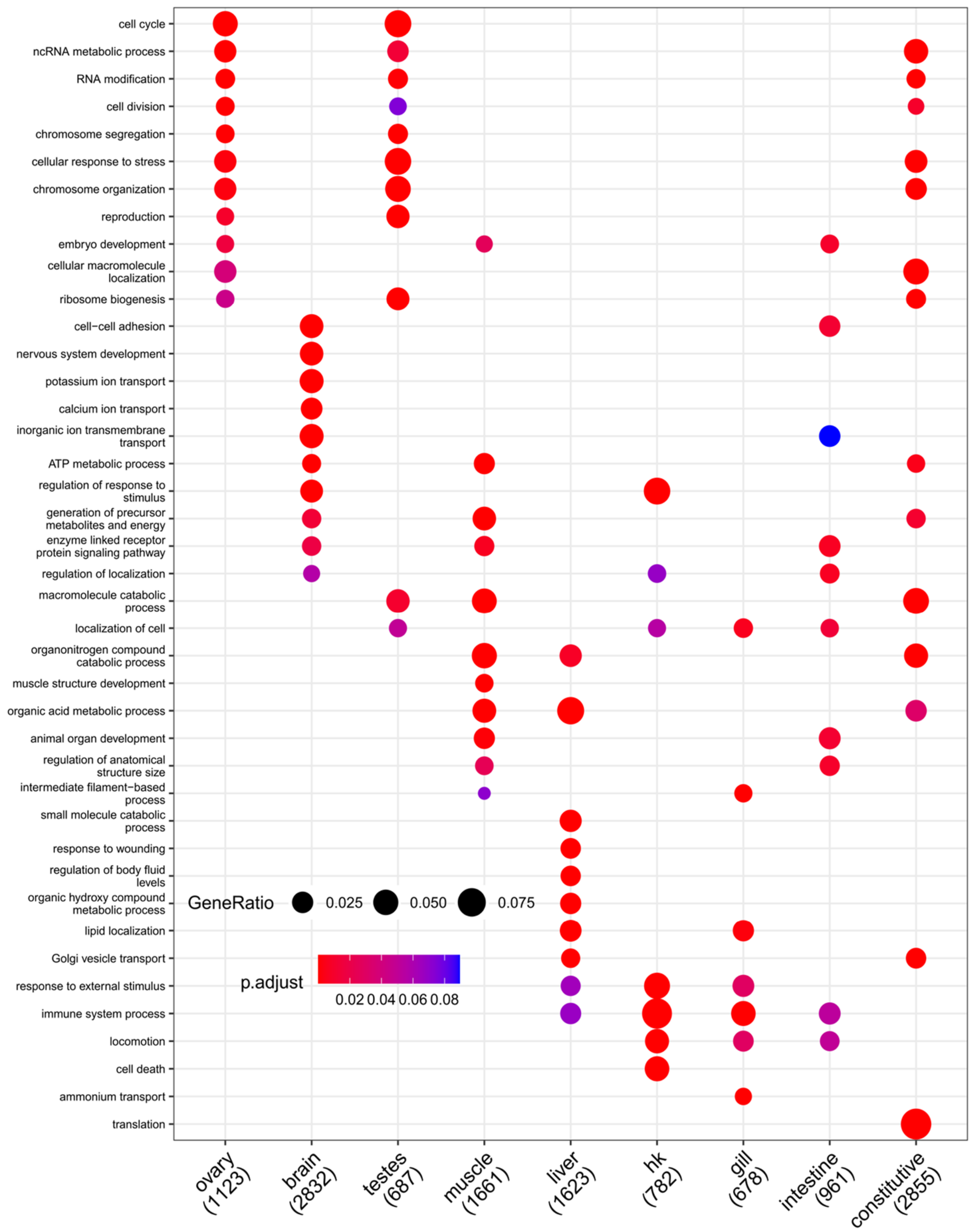

Supplementary Fig. 27. Enriched gene ontology (GO) terms for BodyMap transcriptome of trout. The dot plot compares the most enriched GO terms across SOM clusters shown on the x-axis (shown in Supplementary Fig. 23), restricted to 'Biological Process' terms. Each cluster shows a maximum of five distinct terms, based on FDR adjusted p-value, and additional terms also enriched in other clusters. The y-axis provides GO terms, with the size of each dot indicating gene ratio and colours indicating FDR adjusted p-value according to the shown scales. Numbers in labels indicate the number of genes in the SOM cluster matched to at least one GO term. 'hk' = head kidney, 'intestine' = distal intestine. GO term enrichment was tested by one-sided over-representation analysis (hypergeometric test, clusterProfiler enrichGO), with *p* values adjusted across terms by the Benjamini-Hochberg method.

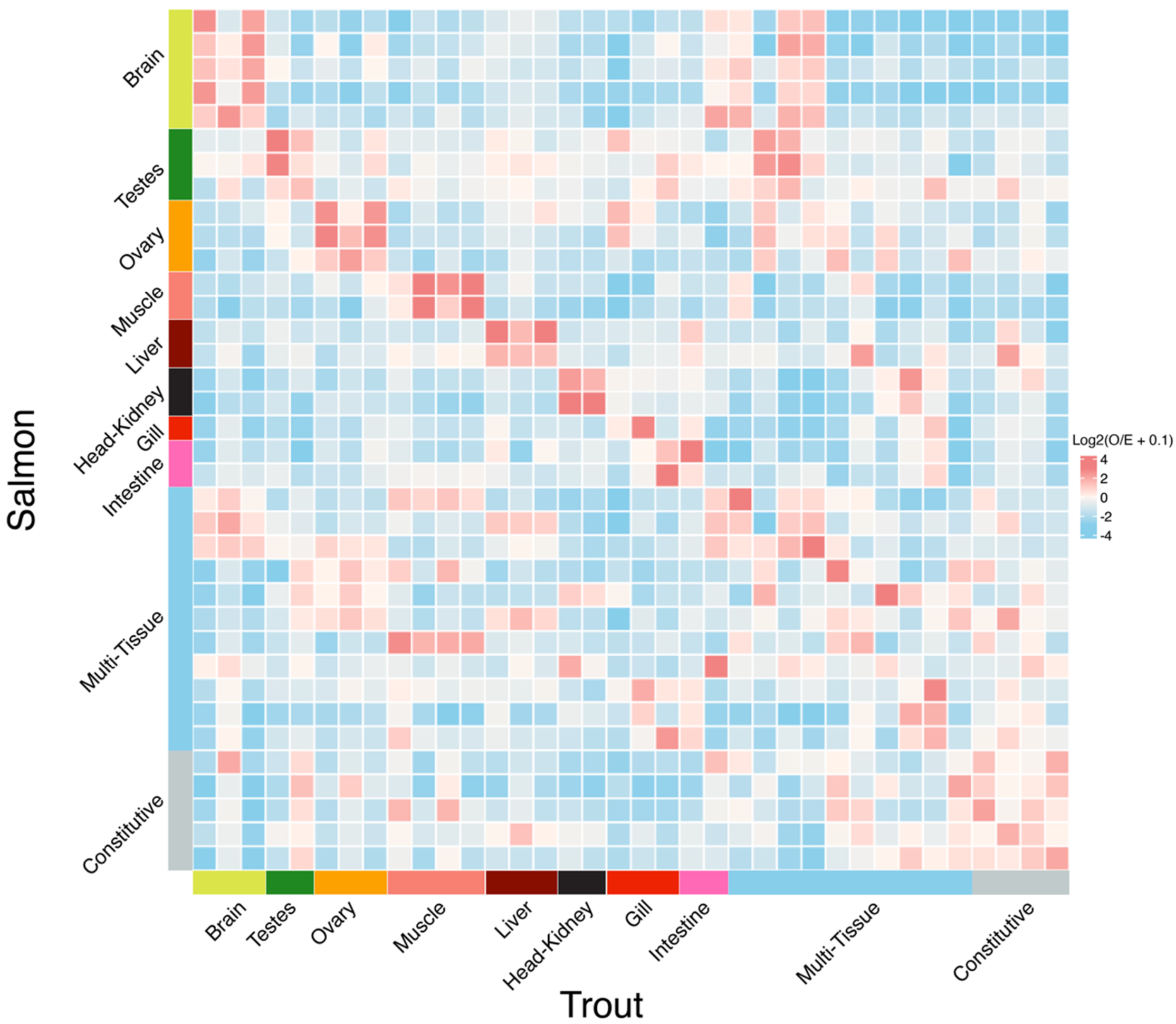

Supplementary Fig. 28. Matrix of observed to expected ratio of orthologs shared across all combinations of salmon and trout BodyMap SOM clusters. Expected values were calculated as the mean of 10,000 random distributions of trout genes across SOM clusters. Colours represent log-2 of observed to expected ratio, such that positive and negative values represent ortholog enrichment and depletion, respectively. Axis labels indicate SOM cluster names, as shown in Supplementary Fig. 22 (salmon) and Supplementary Fig. 23 (trout). 'Intestine' = distal intestine.

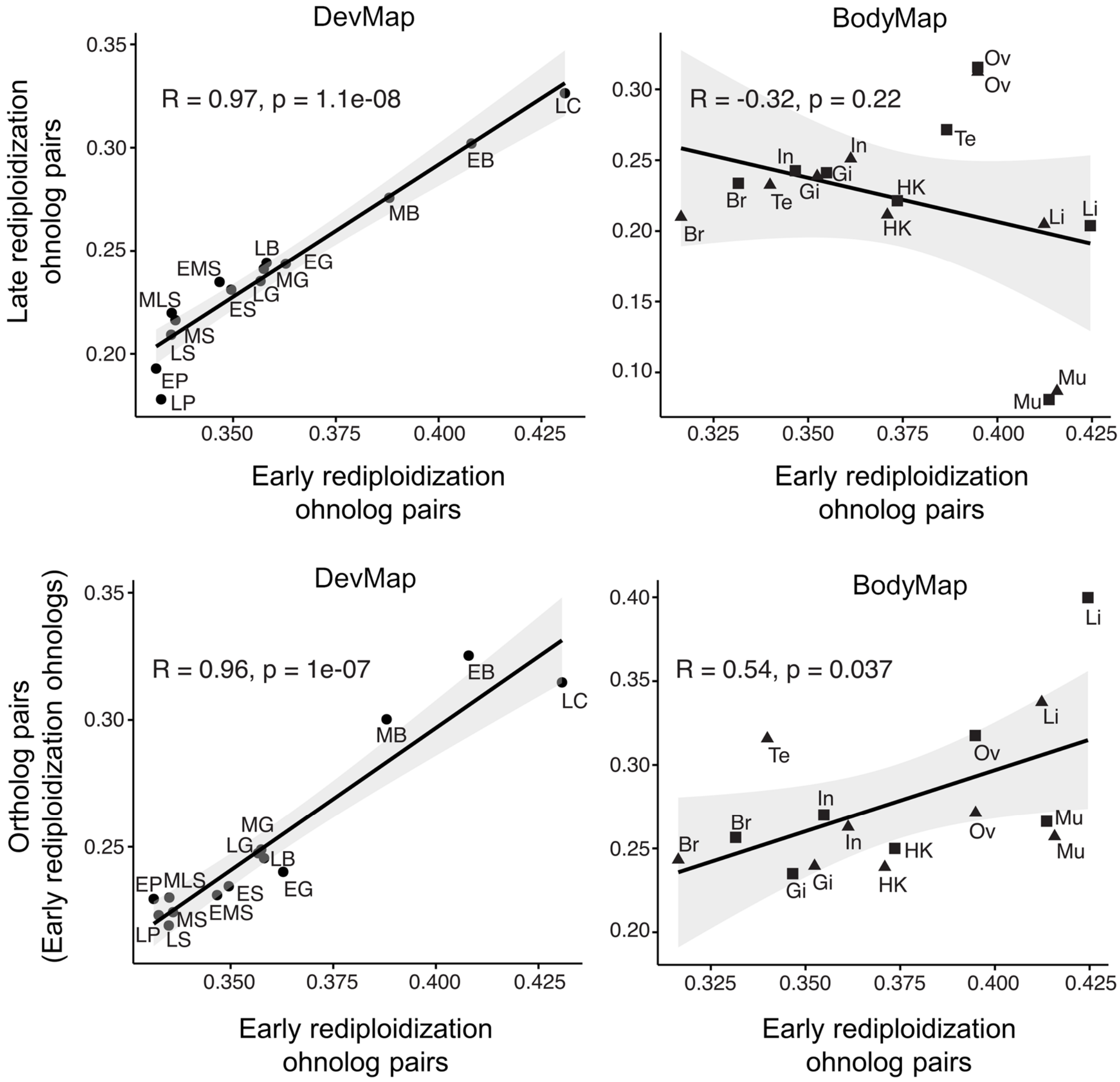

Supplementary Fig. 29. Scatterplots comparing mean JSD values between pairs of ohnologs and orthologs across ontogeny and tissues. The solid lines represent the least-squares fit and the shaded grey bands are 95 % confidence intervals. Pearson's R and *p* values are shown for each comparison. Correlation *p* values are two-sided. Each point is one developmental stage (DevMap panels, n = 14) or one tissue-maturity sample (BodyMap panels, n = 16 for the ohnolog comparison and n = 15 for the ortholog comparison).

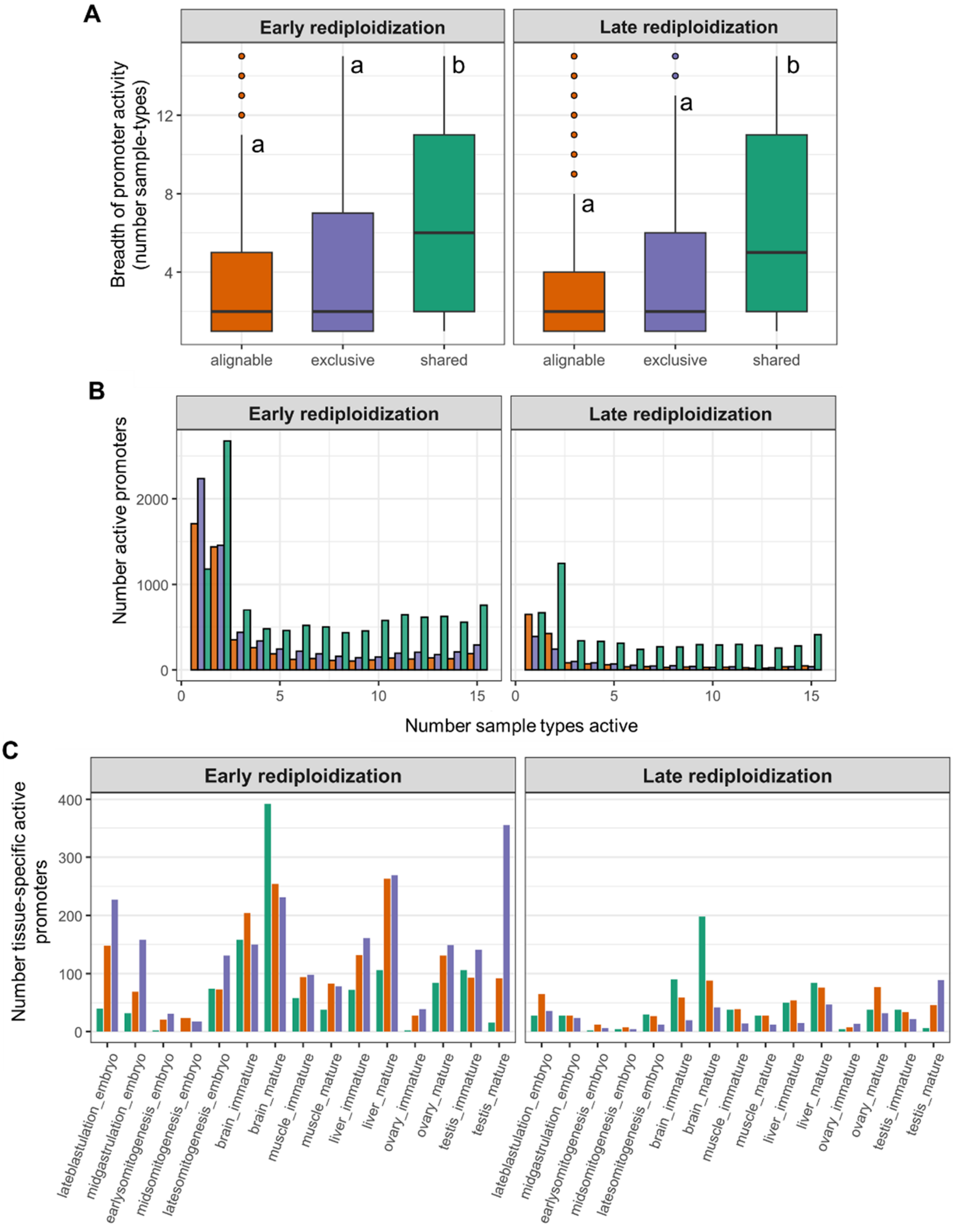
A
Early rediploidization
Late rediploidization
Breadth of promoter activity
(number sample-types)
4
8
12
a
a
b
a
a
b
alignable
exclusive
shared
alignable
exclusive
shared
B
Early rediploidization
Late rediploidization
Number active promoters
0
1000
2000
0
5
10
15
0
5
10
15
Number sample types active
C
Early rediploidization
Late rediploidization
Number tissue-specific active
promoters
0
100
200
300
400
lateblastulation_embryo
midgastrulation_embryo
earlysomitogenesis_embryo
midsomitogenesis_embryo
latesomitogenesis_embryo
brain_immature
brain_mature
muscle_immature
muscle_mature
liver_immature
liver_mature
ovary_immature
ovary_mature
testis_immature
testis_mature
lateblastulation_embryo
midgastrulation_embryo
earlysomitogenesis_embryo
midsomitogenesis_embryo
latesomitogenesis_embryo
brain_immature
brain_mature
muscle_immature
muscle_mature
liver_immature
liver_mature
ovary_immature
ovary_mature
testis_immature
testis_mature

Supplementary Fig. 30. Dynamics of salmon promoter activity across sample types, rediploidization regions and WGD alignment categories. (**A**) Breadth of promoter activity summarized across samples types and stages in the DevMap and BodyMap atlases (embryo stages, adult tissues at immature and mature stages) compared between the Exclusive, Alignable only and Shared categories. Shared promoters have the broadest activity across sample types (unique lowercase letters indicate significant differences according to Wilcoxon's rank test; [Left] Early regions: Alignable only (n = 5,245 unique promoters) vs. Exclusive (n = 6,635 unique promoters): *p*-adj=0.006; Alignable only (n = 5,245 unique promoters) vs. Shared (n = 11,166 unique promoters): *p*-adj=9.96e-287; and Exclusive (n = 6,635 unique promoters) vs. Shared (n=11,166 unique promoters): *p*-adj=4.64e-261; [right] Late regions: Alignable only (n = 1,600 unique promoters) vs. Exclusive (n = 1,238 unique promoters): *p*-adj=1.63e-12; Alignable only (1,600 unique promoters) vs. Shared (n = 5,772 unique promoters): *p*-adj=1.26e-157; and Exclusive (n = 1,238 unique promoters) vs. Shared (n = 5,772 unique promoters): *p*-adj=2.61e-61. (**B**) Consistent with part **A**, many more active promoters from the Shared category (retained as active ohnolog pairs) are active in a higher number of distinct sample types. (**C**) The number of tissue-specific active promoters varies extensively across sample types and alignment categories. For both Early and Late rediploidization regions, mature brain has the highest number of active ohnolog pairs that are inactive in other sample types, whereas mature testis has the highest number of sample-type unique Exclusive promoters.

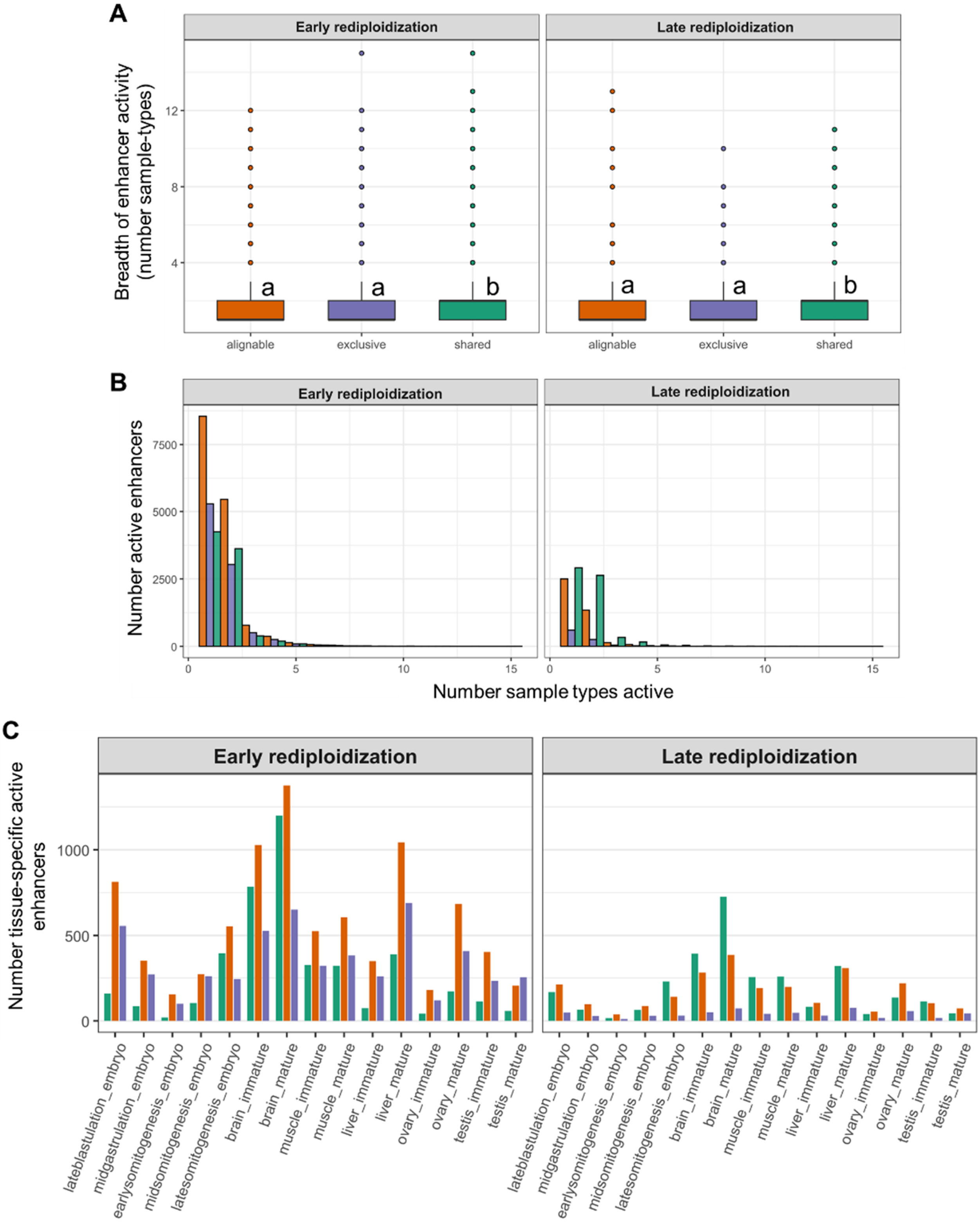

Supplementary Fig. 31. Dynamics of salmon enhancer activity across sample types, rediploidization regions and WGD alignment categories. (**A**) Breadth of enhancer activity summarized across samples types and stages in the DevMap and BodyMap atlases (embryo stages, adult tissues at immature and mature stages) compared between the Exclusive, Alignable only and Shared categories. There is no difference in the breadth of enhancer activity in different sample types across the Exclusive and Alignable only categories. However, the Shared enhancers retained as active ohnolog pairs show significantly higher activity breadth than both other alignment categories (unique lowercase letters indicate

significant differences according to Wilcoxon's rank test; [Left] Early regions: Alignable only (n = 15,443 unique enhancers) vs. Exclusive (n = 9,296 unique enhancers): *p*-adj=0.213; Alignable only (n = 15,443 unique enhancers) vs. Shared (n = 8,654 unique enhancers): *p*-adj=1.16e-15; and Exclusive (9,296 unique enhancers) vs. Shared (8,654 unique enhancers): *p*-adj=4.92e-16; [right] Late regions: Alignable only (n =4,103 unique enhancers) vs. Exclusive (n=936 unique enhancers): *p*-adj=0.260; Alignable only (n = 4,103 unique enhancers) vs. Shared (n = 6182 unique enhancers): *p*-adj=2.66e-43; and Exclusive (n = 9,296 unique enhancers) vs. Shared (n = 6,182 unique enhancers): *p*-adj=3.40e-18. (**B**) Consistent with **A**, the majority of enhancers from all alignment categories show activity in a small breadth of different tissue types. (**C**) The number of tissue-specific active enhancers varies across sample types and alignment categ

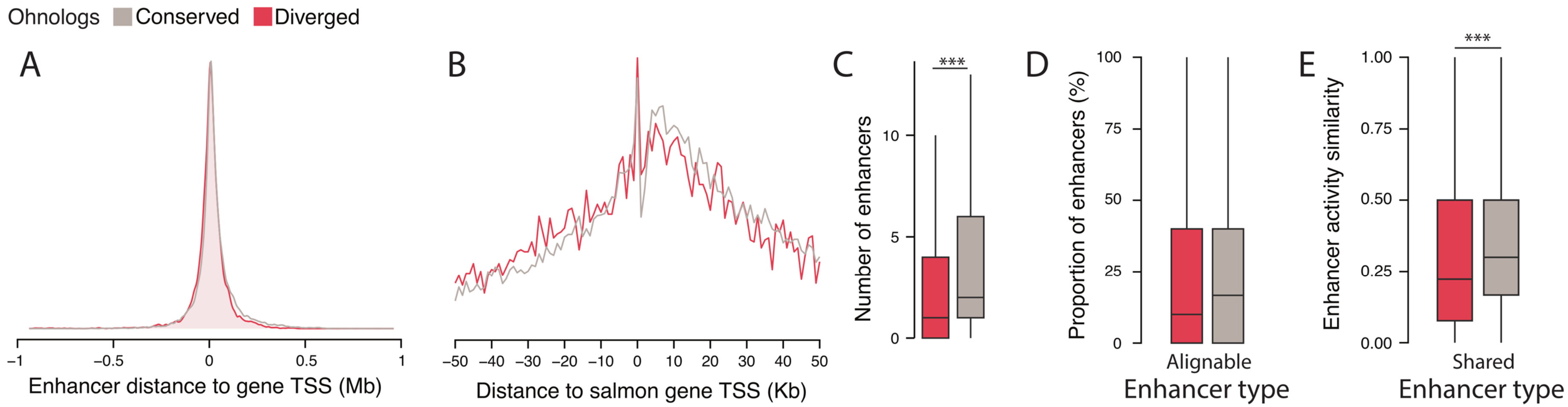

Supplementary Fig. 32. Comparing salmon ohnolog enhancers within the "conserved" (grey) and "diverged" (red) expression categories. (**A**) Density distribution of enhancer distances to gene TSS within regulatory domains. (**B**) Frequency of enhancer distances within +/–50 Kb of gene TSS. (**C**) We observed a difference in the number of enhancers assigned to regulatory domains of diverged and conserved ohnologs (two-sided Wilcoxon rank-sum test: rank-biserial r = 0.12, 95% confidence interval: 0.10–0.15, $p = 2.1 \times 10^{-}20$). (**D**) There was no significant difference in the proportion of enhancers belonging to the Alignable only category between diverged and conserved ohnologs (two-sided Wald test from a binomial generalized linear model (logit link) with robust standard errors clustered by ohnolog, Holm-adjusted across the three enhancer categories: odds ratio 0.92, 95% confidence interval: 0.85–1.00, $p = 0.058$). (**E**) We observed a difference in Shared enhancer activity similarity between diverged and conserved regulation classes, measured by the presence or absence of activity across all sample types (median similarity 0.22 (diverged) vs 0.30 (conserved); two-sided Wilcoxon rank-sum test: rank-biserial r = 0.18, 95% confidence interval: 0.11–0.25, $p = 1.2 \times 10^{-6}$). Box plots show: median (centre line), 25th–75th percentiles (box), 1.5 × interquartile ranges (whiskers); outliers not shown. n = 2,692 diverged and 6,292 conserved ohnolog genes in (**C**); 1,847 diverged and 4,720 conserved ohnolog genes in (**D**); 297 diverged and 1,204 conserved ohnolog pairs in (**E**). Each ohnolog pair, or individual ohnolog genes in (**D**), is an independent biological unit; comparisons are observational.

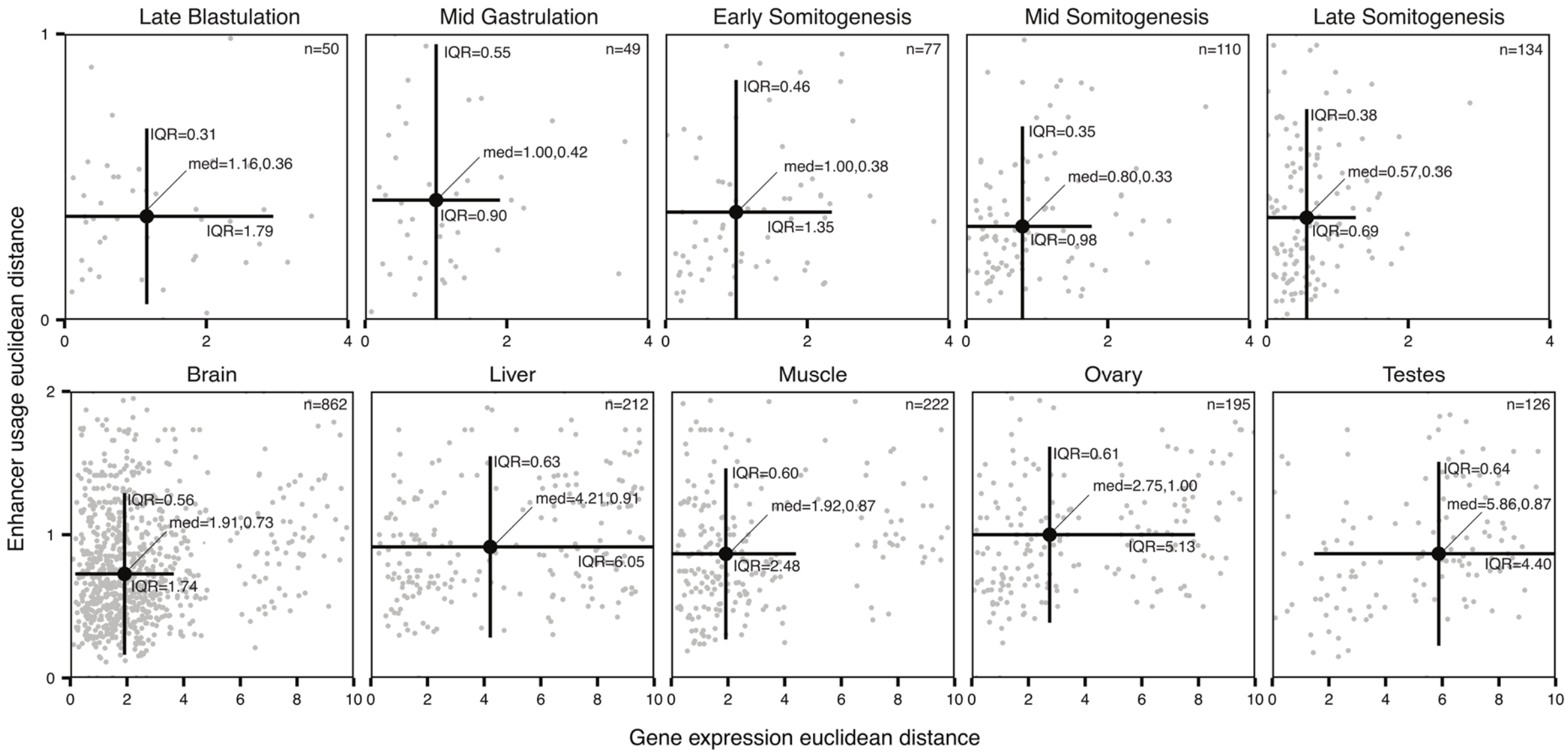

Supplementary Fig. 33. Ohnolog expression distances against enhancer distances in salmon. Euclidean distances between 2:2 ohnolog pairs are compared, for scaled gene expression profiles across sample types against enhancer-usage (proportions of total enhancers active in each sample type). Ohnologs were separated by stage of embryogenesis (top row, DevMap) or tissue type (bottom row, BodyMap) using SOM clustering (main text, Fig. 2). The number (n) of ohnolog pairs per cluster is shown in the top right. Median values are shown ('med') for expression and enhancer distances respectively, with lines showing upper and lower interquartile ranges (IQR).

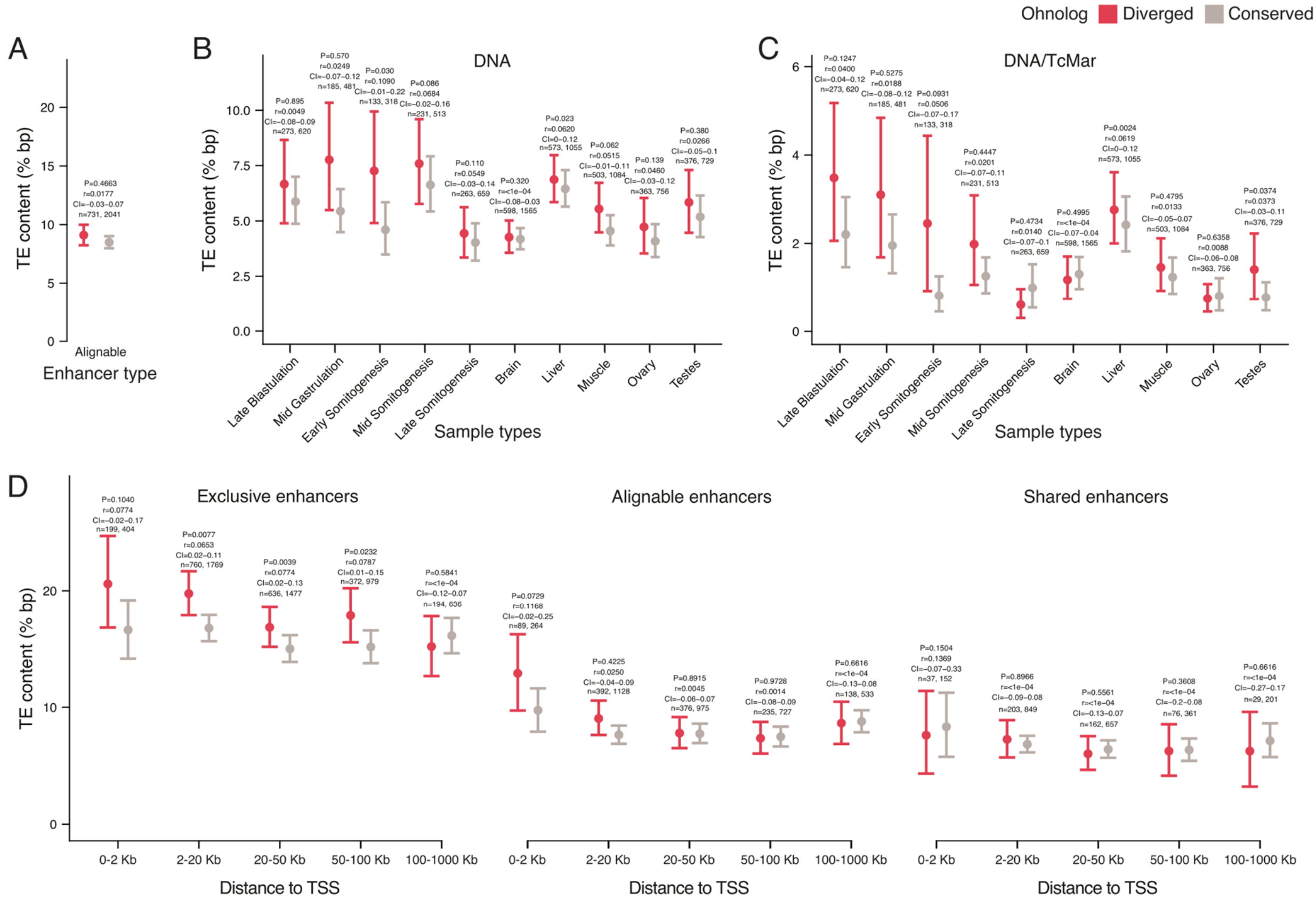

Supplementary Fig. 34. TE content in ohnolog enhancers in salmon. (**A**) Mean percentage of all TE bp overlapping Alignable only enhancers of diverged (red) and conserved (grey) ohnologs. Also shown is the mean content of: (**B**) DNA transposons and (**C**) TcMar class DNA transposons in the Exclusive enhancers active in specific sample types. (**D**) Mean percentage TE bp overlapping Exclusive, Alignable only, and Shared enhancers of ohnologs, stratified by physical distance (in Kb) of each enhancer to the gene transcription start site (TSS). Points show the mean; error bars are 95% bootstrap confidence intervals of the mean (percentile method, 10,000 replicates). Each on-plot annotation reports, for that comparison, the exact *p* value, the rank-biserial effect size (*r*) with its 95% confidence interval, and the two group sample sizes given as n = diverged, conserved ohnolog pairs; the interval beside *r* is the 95% CI of the effect size, distinct from the error bars (95% bootstrap CIs of the mean). All *p* values are from two-sided Wilcoxon rank-sum tests between conserved and diverged ohnologs, unadjusted for multiple comparisons. Each ohnolog pair is an independent biological unit; comparisons are observational.

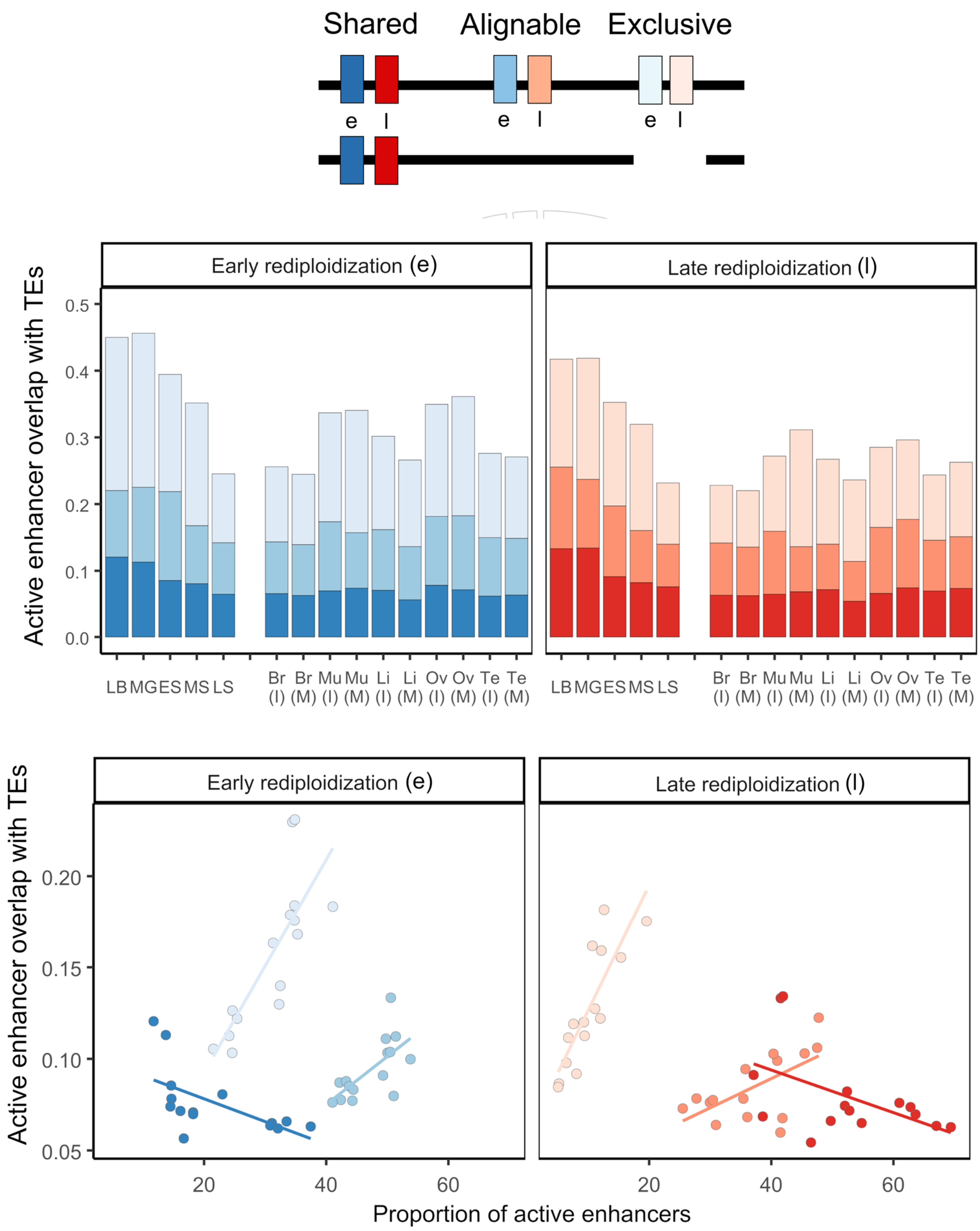

Supplementary Fig. 35. Ontogeny and tissue context constrain the overlap between TEs and active enhancers. The top graph shows the percentage overlap of TEs across different DevMap and BodyMap sample types for each alignment category, and both rediploidization regions. The bottom graph visualises the correlation between percentage overlap of TEs vs. proportion of active enhancers across different DevMap and BodyMap sample types. Associated statistics for the correlation plots are provided in Supplementary Table 16.

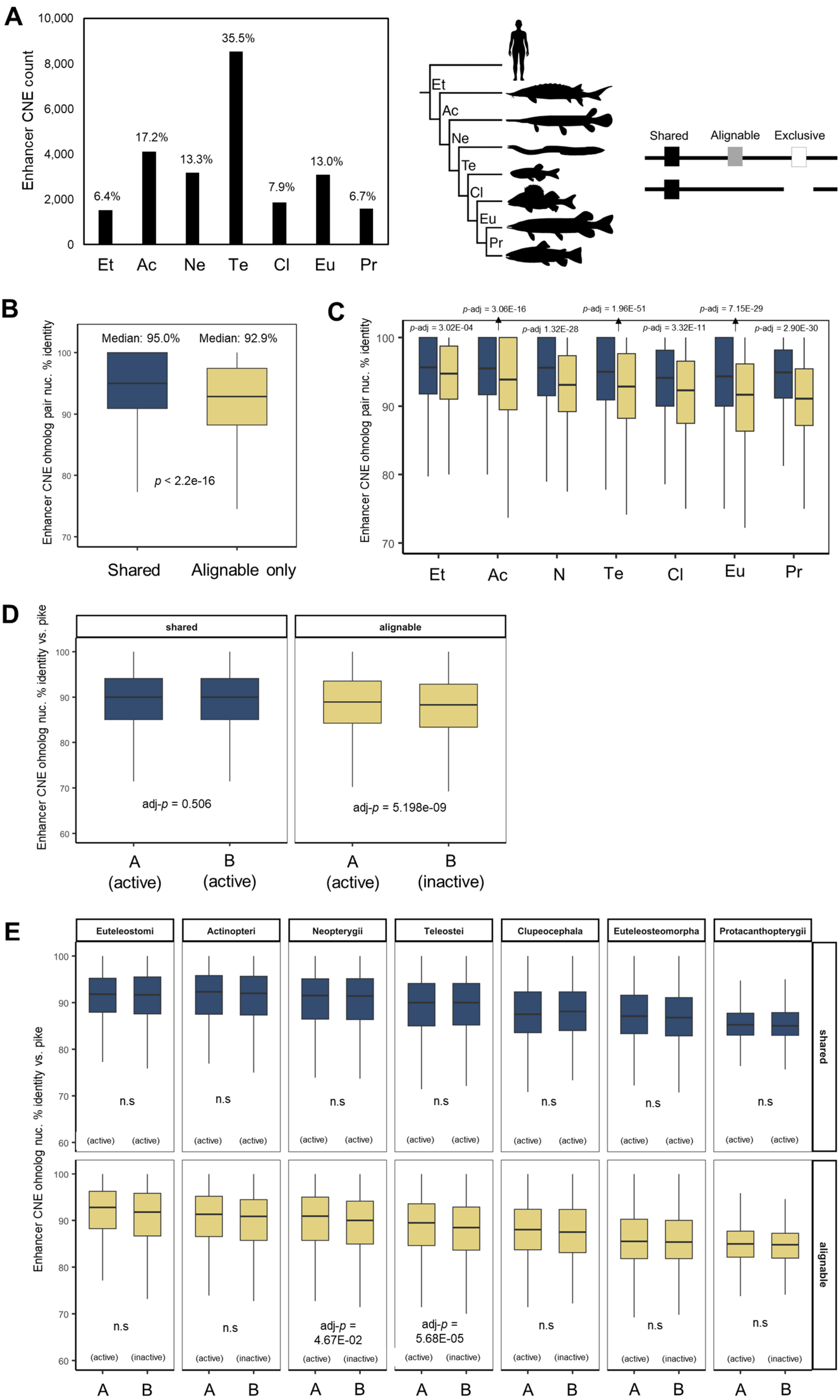
A
Enhancer CNE count
10,000
8,000
6,000
4,000
2,000
0
6.4%
17.2%
13.3%
35.5%
7.9%
13.0%
6.7%
Et
Ac
Ne
Te
Cl
Eu
Pr
Shared
Alignable
Exclusive
B
Enhancer CNE ohnolog pair nuc. % identity
Median: 95.0%
Median: 92.9%
p < 2.2e-16
Shared
Alignable only
C
Enhancer CNE ohnolog pair nuc. % identity
p-adj = 3.02E-04
p-adj = 3.06E-16
p-adj 1.32E-28
p-adj = 1.96E-51
p-adj = 3.32E-11
p-adj = 7.15E-29
p-adj = 2.90E-30
Et
Ac
N
Te
Cl
Eu
Pr
D
Enhancer CNE ohnolog nuc. % identity vs. pike
shared
alignable
adj-p = 0.506
adj-p = 5.198e-09
A (active)
B (active)
A (active)
B (inactive)
E
Enhancer CNE ohnolog nuc. % identity vs. pike
Euteleostomi
Actinopteri
Neopterygii
Teleostei
Clupeocephala
Euteleosteomorpha
Protacanthopterygii
shared
alignable
n.s
adj-p = 4.67E-02
adj-p = 5.68E-05
(active)
(inactive)
A
B

Supplementary Fig. 36. Overview of enhancer-CNEs evolution in the salmon genome. (A) Counts of enhancer-CNEs stratified by phylogenetic age. (**B** and **C**) Shared enhancer-CNEs (n=14,732), active at both ohnologs, show significantly higher sequence identity than Alignable only enhancer-CNEs (n=15,014), where enhancer activity is restricted to one ohnolog in a pair (Wilcoxon's rank test, done globally [**B**] and per CNE age category [**C**]). The total numbers of enhancer-CNEs across different phylogenetic ages are detailed in Supplementary Table 17. (**D** and **E**) Shared enhancer-CNE ohnologs from the same pairs show no difference in respective sequence-level divergence with their shared northern pike ortholog. However, for enhancer-CNEs in the Alignable category, the inactive enhancer-CNE ohnolog shows significantly greater sequence divergence with the pike ortholog, globally and in two CNE age categories (Wilcoxon's rank test, done globally [**D**] and per CNE age category [**E**]). The numbers for shared enhancer-CNEs for A (active) and B (active) are n = 7,366 and n = 7,366 respectively. The numbers for alignable enhancer-CNEs for A (active) and B (inactive) are n = 7507 and n = 7507, respectively. Silhouettes representing different vertebrate/fish lineages in panel a were obtained from PhyloPic under CC0 1.0 Universal Public Domain Dedication licenses.

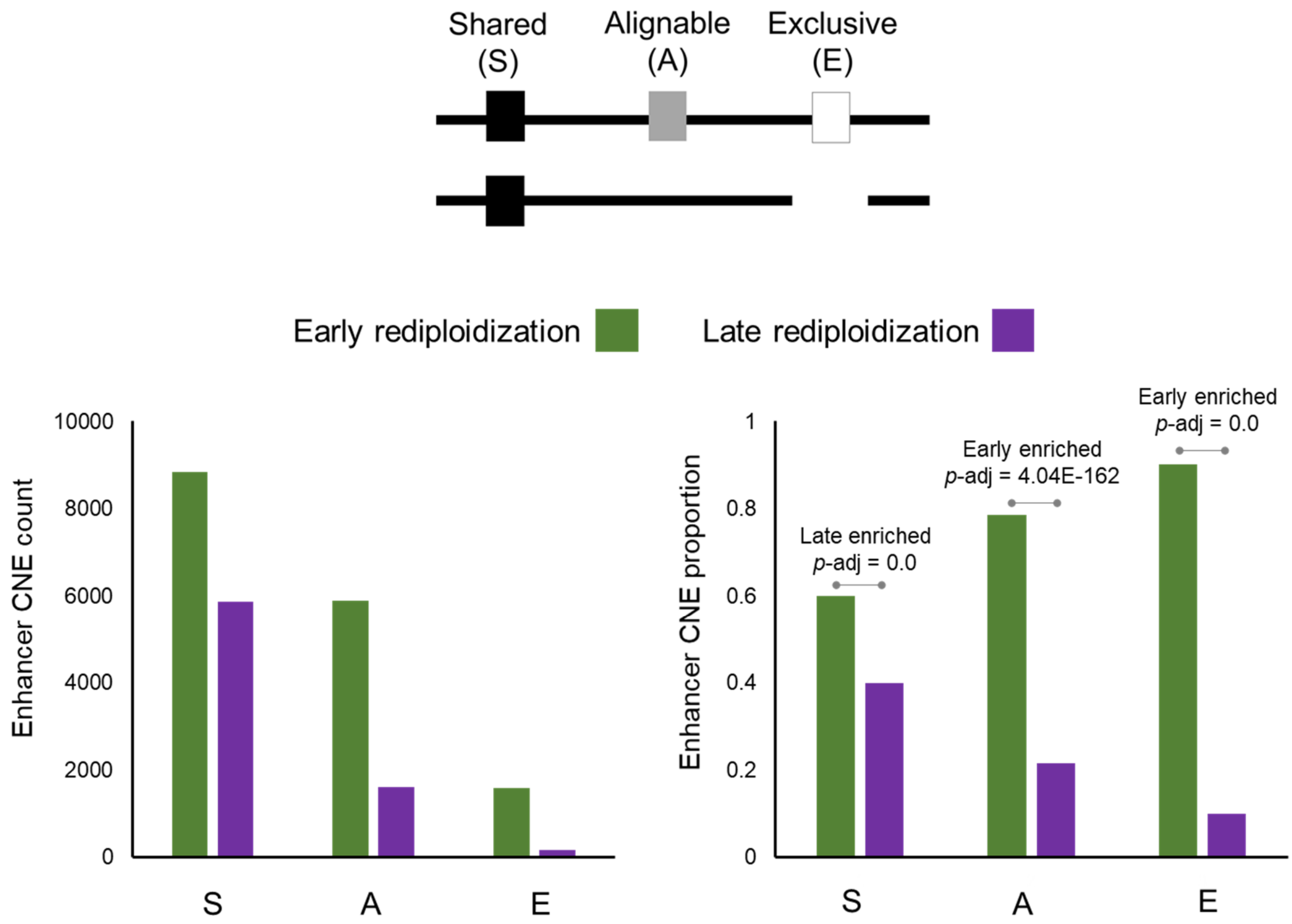

Supplementary Fig. 37. Enhancer-CNEs are differentially enriched in the salmon genome dependent on rediploidization history. Enhancer-CNE counts (left graph) and proportions (right graph) are shown for Early and Late rediploidization regions and each WGD-alignment category. The Shared enhancer-CNEs are significantly enriched in Late rediploidization regions, whereas the opposite is the case for Alignable only and Exclusive enhancer-CNEs.

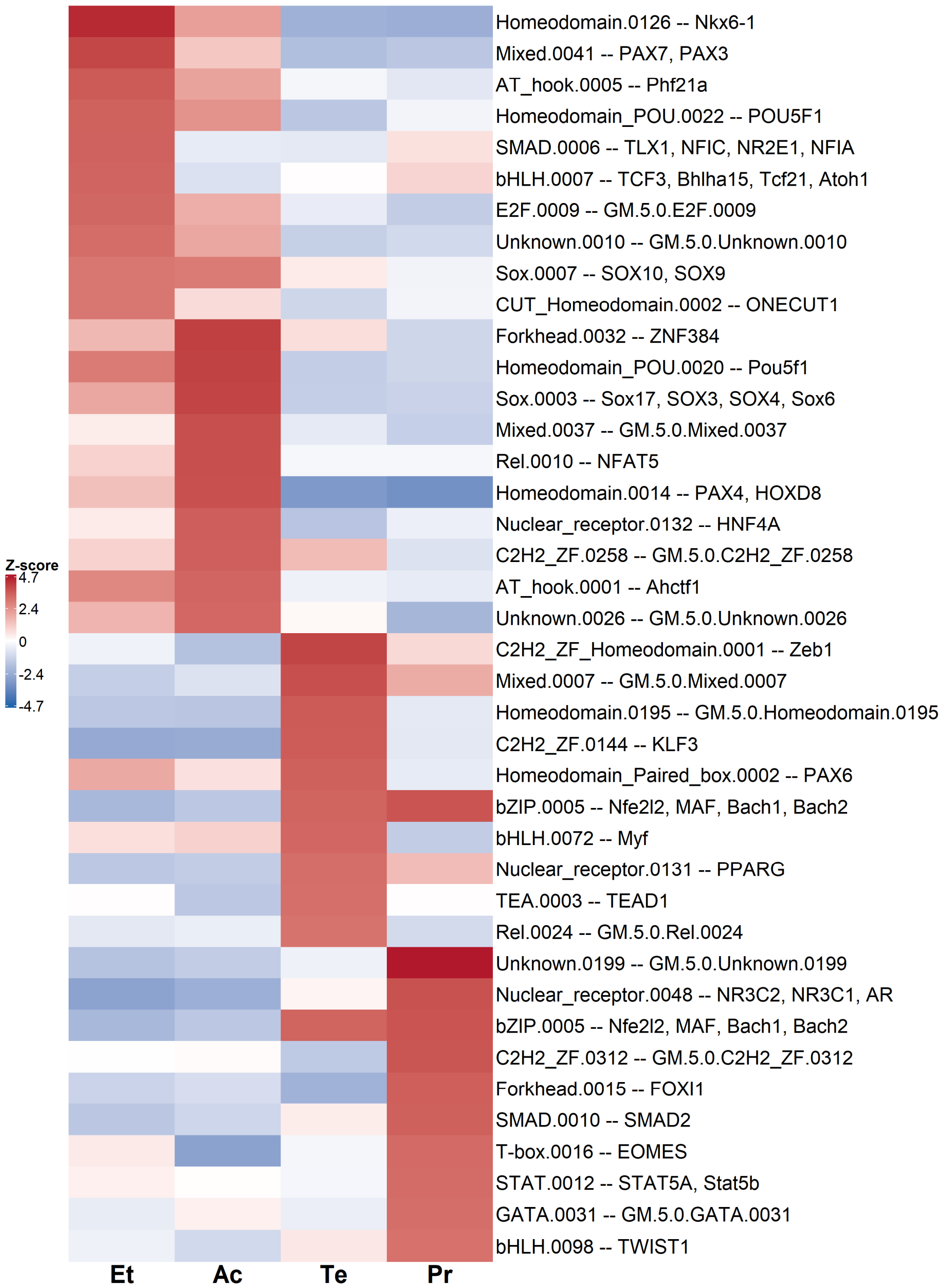

Supplementary Fig. 38. Differential enrichment of DNA motifs in enhancer-CNEs of distinct phylogenetic ages. The heatmap shows differential enrichment of motifs using Z-score values comparing four phylogenetic ages ('Et' =Euteleostomi; 'Ac' =Actinopteri, 'Te' =Teleostei, and 'Pr' = Protacanthopterygii). The top ten uniquely enriched motifs (gimme.vertebrate.v5.0 motif database) per category are included, with motif names provided along with predicted TFs (following '--' in each motif title).

Supplementary Text

Supplementary Text 1. Sampling Protocols for DevMap and BodyMap

Executive summary: This section provides the detailed protocols used for Atlantic salmon and rainbow trout samples taken: i) during embryogenesis (DevMap) and ii) from adult tissues of both fish sexes at two stages of maturation (BodyMap), for the purpose of RNA-seq ATAC-seq and ChIP-seq.

S1.1. DevMap sampling

S1.1.1. Materials and Reagents

- Petri dishes
- Watchmaker forceps
- 1.5 mL Eppendorf tubes
- Vortex
- Fixed angle centrifuge
- Ice tray
- Crushed ice
- Cold water from egg trays
- PBS

S1.1.2. Number of salmonid eggs required per stage

- Blastula: n=40
- Gastrula: n=30
- Early somitogenesis: n=20
- Mid-late somitogenesis: n=10
- Pharyngula stage: n=5

S1.1.3. Blastula to early gastrulation

Note: During blastulation to early stages of gastrulation, the cell mass is not tightly bound to the yolk and can be easily dissociated mechanically. However, it is also easy to lose or break cells. Because of this, the best approach is to dechorionate eggs under a dissection microscope and use a bore tip pipette to transfer each blastula to an Eppendorf tube.

1. Place the required number of eggs in a petri dish with de-chlorinated, aerated, cold water (from egg trays). Required number of eggs is stage dependent (above).
2. Place another petri dish with cold PBS under a dissection microscope.
3. Transfer one egg to the petri dish under the microscope.
4. Dechorionate using watchmaker forceps. Take care not to damage the blastula.
5. Use light mechanical pressure from the forceps to separate embryo material from yolk. For this step, a very careful and light scraping motion on the sides of the blastula works best.
6. Using a P1000 pipette set to 100 µL, with a bore pipette tip, transfer the embryo material to a 1.5 mL Eppendorf tube on ice.
7. Repeat for the required number of eggs for each replicate.
8. Let the embryo material decant to the bottom of the tube, then remove as much supernatant as possible and add 1000 µL of cold fresh PBS.
9. For samples meant for ATAC-seq library preparation, continue from step S3.1.3, for

samples meant for crosslinking and subsequent ChIP-seq library preparation, continue from step S4.1.

S1.1.4. Mid-gastrulation onwards

During gastrulation, normally at the onset of epiboly, cells will become more tightly bound to each other and to the yolk surface. This makes it more challenging to isolate embryos under the dissection microscope, but it also makes it easier to separate the gastrulas from the yolk without accidentally dissociating them. Because of this, eggs at this stage are dechorionated within a well of a six well plate, and vortexed to separate embryonic mass from yolk and oils.

1. Pre-chill swinging bucket centrifuge to 4ºC.
2. Place the required number of eggs for one biological replicate in the well of a six well plate, fill well with de-chlorinated, aerated, cold water (from egg trays). Place six well plate on ice.
3. Using watchmaker forceps, transfer one egg into another well, along with 1 mL of PBS.
4. Dechorionate egg and remove chorion, take care not to leave any embryonic material inside chorion.
5. Repeat for all eggs in one biological replicate.
6. Transfer contents of well into an 1.5 mL Eppendorf tube, on ice.
7. Vortex tube for ~10 secs.
8. Centrifuge on fixed angle centrifuge, 300g for 6 mins at 4ºC
9. Remove supernatant and add 1000 µL of fresh cold PBS.
10. Repeat the last centrifugation.
11. The samples are ready for cell isolation (S3.1.4) or crosslinking (S4.1), depending on downstream applications.

S1.2. BodyMap sampling

S1.2.1. Materials and Reagents

- One polystyrene box with ice, one with dry ice
- PBS plus (see buffers table)
- Labelled cryotubes or Eppendorf tubes
- Balance and weigh boats
- Scalpel and blades
- Tweezers and scissors
- Labelled Petri dishes
- 1 L of cold PBS
- Ethanol and distilled water to clean the tools
- Aluminum foil, kimwipes and absorbent paper
- Marker, pen and notepad
- Dietrich solution (histology)
- Air-tight containers (histology)

Note: Fish should be starved for 24 hr before sampling. Fish should be humanely killed using a Schedule 1 method before proceeding immediately with measurements and tissue sampling.

S1.2.2. Tissue sampling

1. Brain: make a transverse cut across the skull and lateral cuts (posterior to anterior) to create a flap which you can remove along with any skin, cartilage, or fat. Identify the

brain (A & B, directly below).

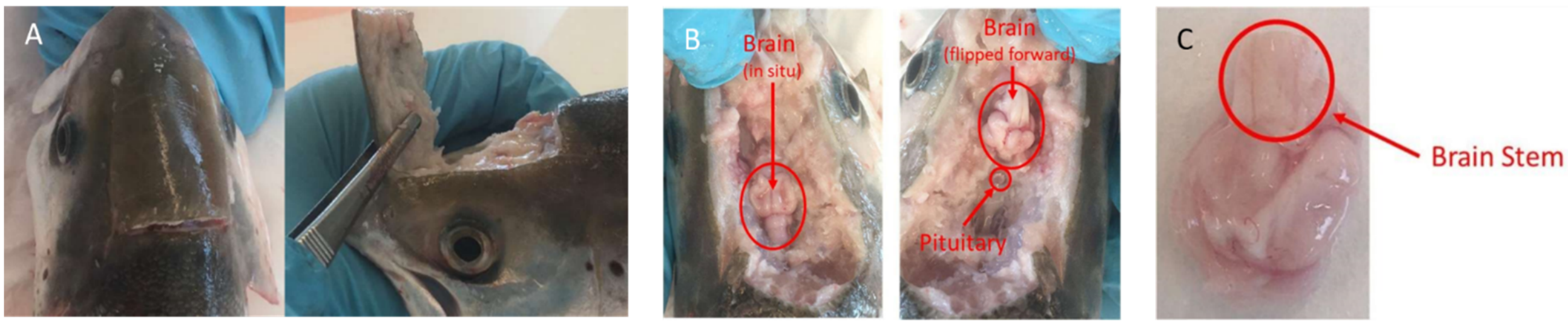

Brain sampling. (A) Access to the brain. (B) Sampling of the brain. (C) Removal of the brain stem

2. Carefully cut across the brain stem and transfer the whole brain to a petri dish placed on ice and containing sufficient PBS to rinse the tissue. Remove the brain stem and pituitary (B & C, directly above) and any contaminating tissue, then transfer to a clean petri dish.
3. Open the fish by using a shallow horizontal cut 5mm below the lateral line from the gill to the anal fin, and make two vertical cuts from the lateral incision to the belly. Cut through the skin and muscle but not so deep that you damage the internal organs (A, directly below).
4. Open the fish by flipping the muscle and skin layer down and stretching it to expose the internal organs (B, directly below).
5. Remove fat if necessary and expose the digestive organs (B, directly below). Then sample target organs in the following order: liver, gonad, distal intestine, head kidney, gill and skeletal muscle.

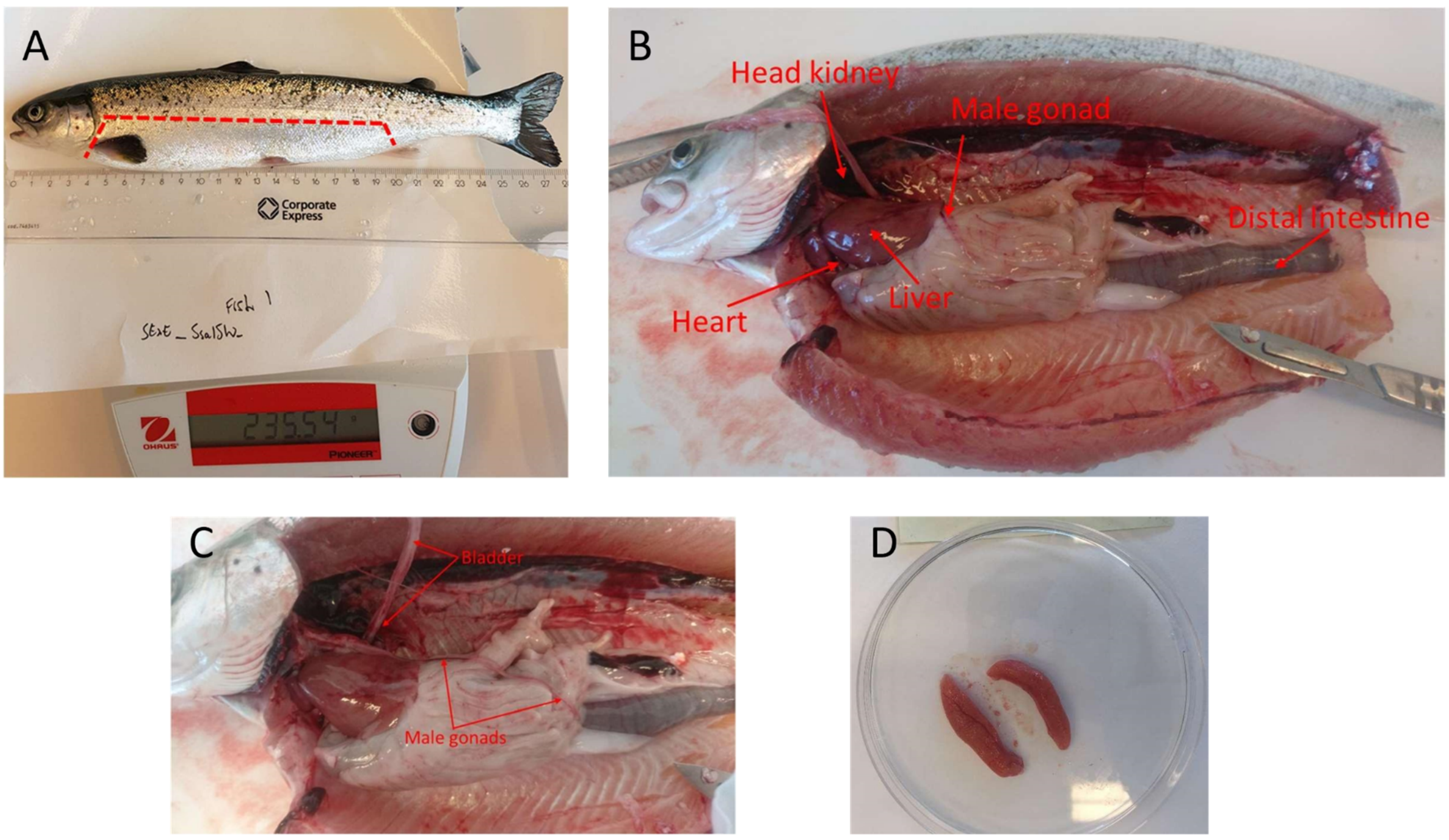

Tissue sampling in an immature Atlantic salmon. (A) lateral incision. (B) View of salmonid organs. (C) Immature male gonads. (D) Immature female gonads

6. Liver: Remove the intact liver after cutting the hepatic vein, and transfer to a clean petri dish on ice containing PBS. If necessary, remove a 1cm strip from the middle of the tissue

(in an anterior → posterior direction). Avoid or remove the vascular network and transfer the final trimmed sample to a sample petri dish.

Note: Because liver is an easy tissue to access and relatively large, it can be used as a source of DNA for whole genome sequencing of the individual. Collect at least 2 mg of tissue.

7. Gonads:

- In immature male_fish, testes are similar in appearance to the swim bladder (C, directly above), both are fine and delicate and look like sausage skins. Take a photo of the gonads *in situ*, then release the testes by cutting at the anterior end. The bladder lies over the testes and can be lifted to expose the underlying testes.
- In mature male fish, testes appear as large white sacks. Take a photo of the gonads *in situ*, then release the testes by cutting at the anterior end.
- In immature female fish, the ovaries are found under the digestive organs and appear as pinkish sacks (D, immediately above). Take a photo of the gonads *in situ*, then using tweezers, pull these away from the body cavity and cut at the point of attachment to liberate the organ.
- In mature female fish, the ovaries are large pink sacks and possibly contain eggs. Take a photo of the gonads *in situ*, then using tweezers, pull these away from the body cavity and cut at the point of attachment to liberate the organ.
- Gonad histology sampling: Remove sample(s) for histological analysis and place in appropriate histology petri dish ('H', directly below), then add PBS to remaining whole tissue (in collection dish; 'C') and histology sub-samples to prevent the tissue from drying out. If possible, wash away or press out and discard as many eggs/sperm as possible. The protocol for histology is provided in section S1.3 below.

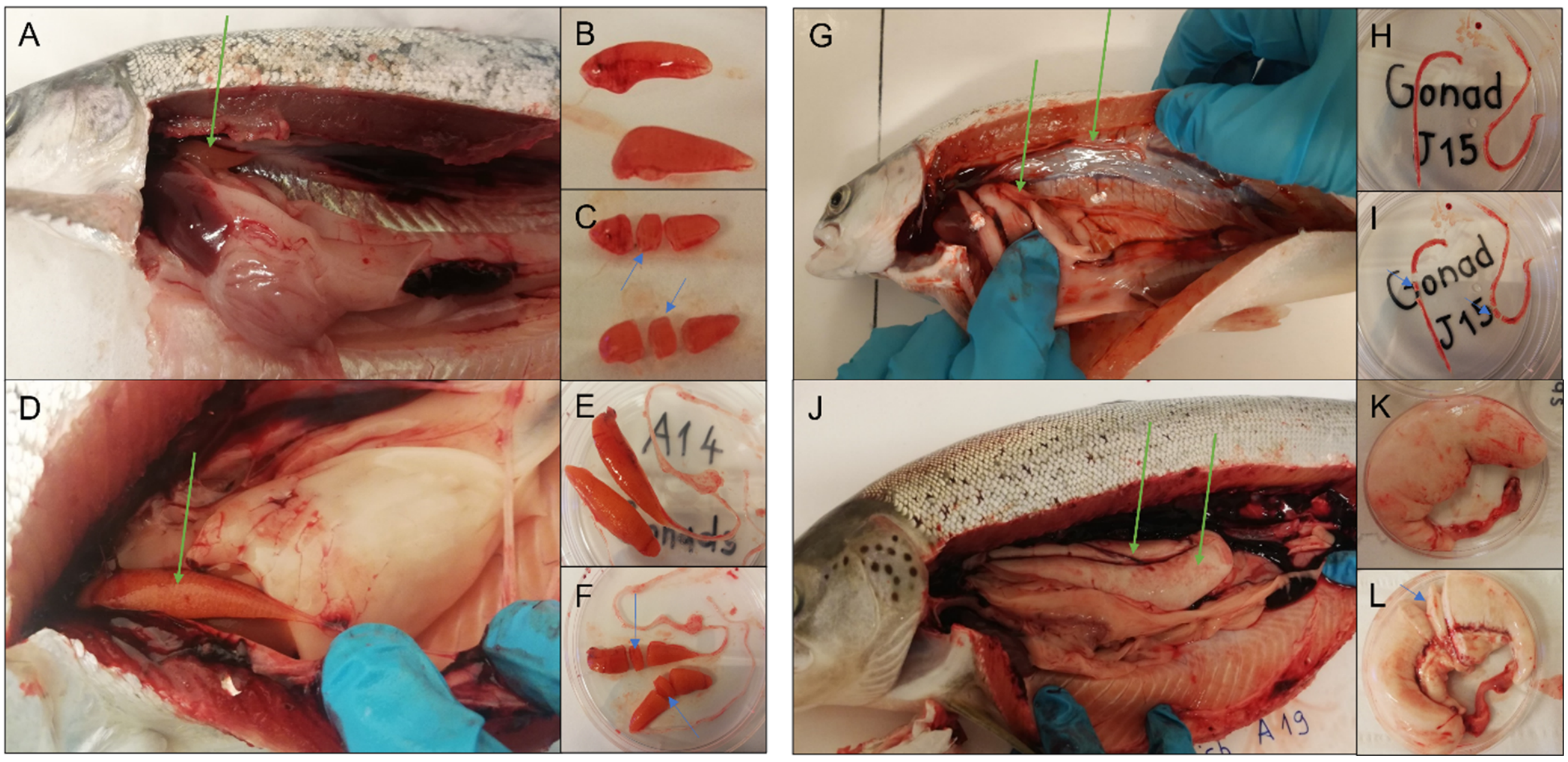

Gonad sampling and sampling for histology. (A to C) Immature female gonads. (D to F) Mature female gonads. (G to I) (A, D, G and J) green arrows show the location of the gonads. (B, E, H and K) Gonads after extraction. (C, F, I, L) blue arrows show the portion of the gonad sent for histology investigation in our study.

8. Distal intestine: Make a cut in the anterior part of the main digestive link (at the esophagus/ pharynx level) to release the beginning of the digestive tract. Lift the intestines out and locate the most terminal (distal) region proximal to the anus, cut to

release the tract completely.

9. Locate and isolate the distal intestine (DI). If feces are present, place the DI in a clean petri dish and gently use your finger to press them out. Transfer the DI to a petri dish containing PBS and cut it open along its length to expose its interior, then gently scrape with the back of a scalpel or spatula to remove mucus. Wash thoroughly in PBS, and transfer to the Sample petri dish (A & B, directly below).

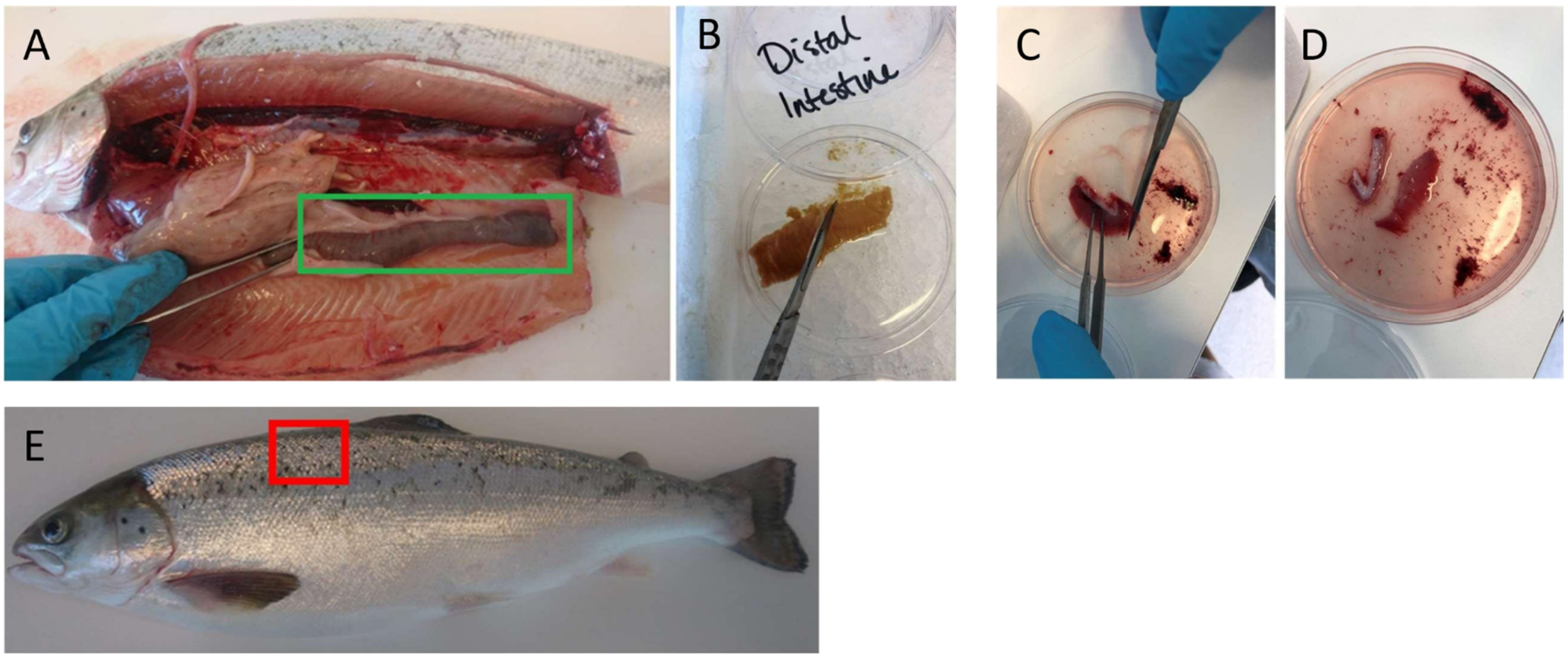

Sampling images. (A and B) Distal intestine sampling. (C and D) Gill sampling. (E) Localization of the muscle sampling

10. Head kidney: Remove and discard the heart to give better access to the head kidney, which is the most anterior portion of dark red tissue found under vertebral line and behind the head. The head/anterior kidney can be identified by its location and because it looks wider than the remaining more posterior kidney that follows the vertebral line along the body. The head kidney is fragile so care must be taken when extracting it. A small plastic spoon or spatula may be appropriate. Place directly into a sampling petri dish containing PBS.
11. Gill: Cut the gill at its root and immerse in a petri dish filled with PBS and scrape the surface to remove blood. Cut out and discard cartilaginous tissues (rakers) but retain the gill filaments and transfer these to a fresh petri dish with PBS (C & D, directly above).
12. Muscle: Sample a generous amount of fast-twitch myotomal muscle from the region anterior to the dorsal fin (E, directly above), avoiding the darker red muscle layer found close to the skin. Place into a corresponding petri dish and remove any skin and fat. Transfer cleaned muscle sample to petri dish and immerse in PBS.

Note: Frozen skeletal muscle is a relatively poor source of nuclei and the library protocols involve extensive filtering, which will return low yields of nuclei. For this reason, we recommend sampling at least 6g to ensure sufficient material.

S1.2.3. Preservation of tissues for RNA preservation

1. Place an unfolded piece of aluminum foil (no less than 10 x 10cm) or disposable weight-boat on a flat layer of dry ice ensuring good contact. At the same time, place two pairs of tweezers in dry ice, such that the ends of the tweezers become very cold. Then place pre-labelled cryotubes into dry ice so that they are cold when receiving samples.
2. Transfer tissue from sampling petri dishes to a new and dry petri dish. When possible, use

Kimwipes or equivalent to remove excess PBS. Then record the sample weight (subtracting the weight of the petri dish). Recommended sample collection weights (in mg) are shown for each tissue in the table immediately below.

Tissue	Mg to collect	Approx. µg total RNA per mg
Brain	15	1
Distal Intestine	15	1
Gill	15	1
Gonad	15	1
Muscle	100	0.25
Liver	15	3
Head kidney	15	2

3. With a sterile and clean scalpel, cut tissue samples into small pieces (approximately 2-3mm^3).
4. Using room temperature tweezers, drop tissue pieces of the recommended weights into 5-10 volumes RNAlater (or an equivalent reagent). See recommendations from the manufacturer, but we recommend storing the sample at 4°C overnight before transferring to -20°C for longer-term storage.
5. Next, using room temperature tweezers, drop all remaining tissue pieces onto the foil (if the tissue is sticky, this may require some aggressive flicking, or the use of a second pair of tweezers).
6. Tissue will freeze rapidly, try to place pieces separately on the foil and avoid clumping. After freezing the last tissue piece, use the chilled tweezers from step 2 to pluck each tissue piece from the foil and place in a pre-labeled chilled cryotube. If everything is kept cold, the small frozen pieces should not stick to each other or to the cryotube.

Note: the chilled tweezers will rapidly warm up once removed from dry ice. We recommend swapping between at least two pairs of tweezers to ensure only cold tweezers interact with samples.

7. Next, place the tubes in -80°C. This will allow you to later extract tissue without thawing the whole sample. Always work on dry ice and with cold tweezers for this later step.

S1.3. Gonadal sampling for histological analysis

S1.3.1. Materials & Reagents

- Dietrich's solution
- Small volume (15-20 mL) containers/tubes for storage of histological samples (e.g. https://www.fishersci.ca/shop/products/simport-scientific-securtainer-ii-tamper-evident-specimen-containers-5/p-4121042). These must be leak-proof.

S1.3.2. Buffer recipes

Dietrich's solution (1,020 mL, sufficient for approx. 50 samples):

- 600 mL ddH_2O
- 300 mL 95% ethanol
- 100 mL 40% formaldehyde
- 20 mL acetic acid

S1.3.3. Protocol

After excision, take a photo and weigh the entire gonads (section S1.2.2). If the gonad is large enough, prepare to sample three portions (anterior, middle and posterior), otherwise sample just the middle portion. Take another picture after sampling.

1. For histological sampling, make a clean cut using a sharp blade to ensure the integrity of the tissues and remove a section(s) not larger than $1cm^3$.
2. Place the samples in 10 volumes (at least) of Dietrich's solution for 10 days in sample containers. During this time, the samples should be kept cool and in the dark.
3. After 10 days, transfer the sample into new sample containers, then fill with 50% ethanol (at least 10 volumes) and let sit for 5 days.
4. After 5 days, samples are dehydrated through a graded series of ethanol and embedded in paraffin. Sections should be cut at a thickness of 4 µm using a microtome, mounted on slides, stained with haematoxylin and eosin and examined under photomicroscope with imaging capabilities (e.g. we used an Olympus Vanox, New York Microscope Company, Hicksville, NY, USA) to evaluate the maturity stage of the individuals (sexually immature or mature) by general morphology observation of the gametes development inside the slides.

Resulting histology images of each individual from our study is provided below for Atlantic salmon and rainbow trout.

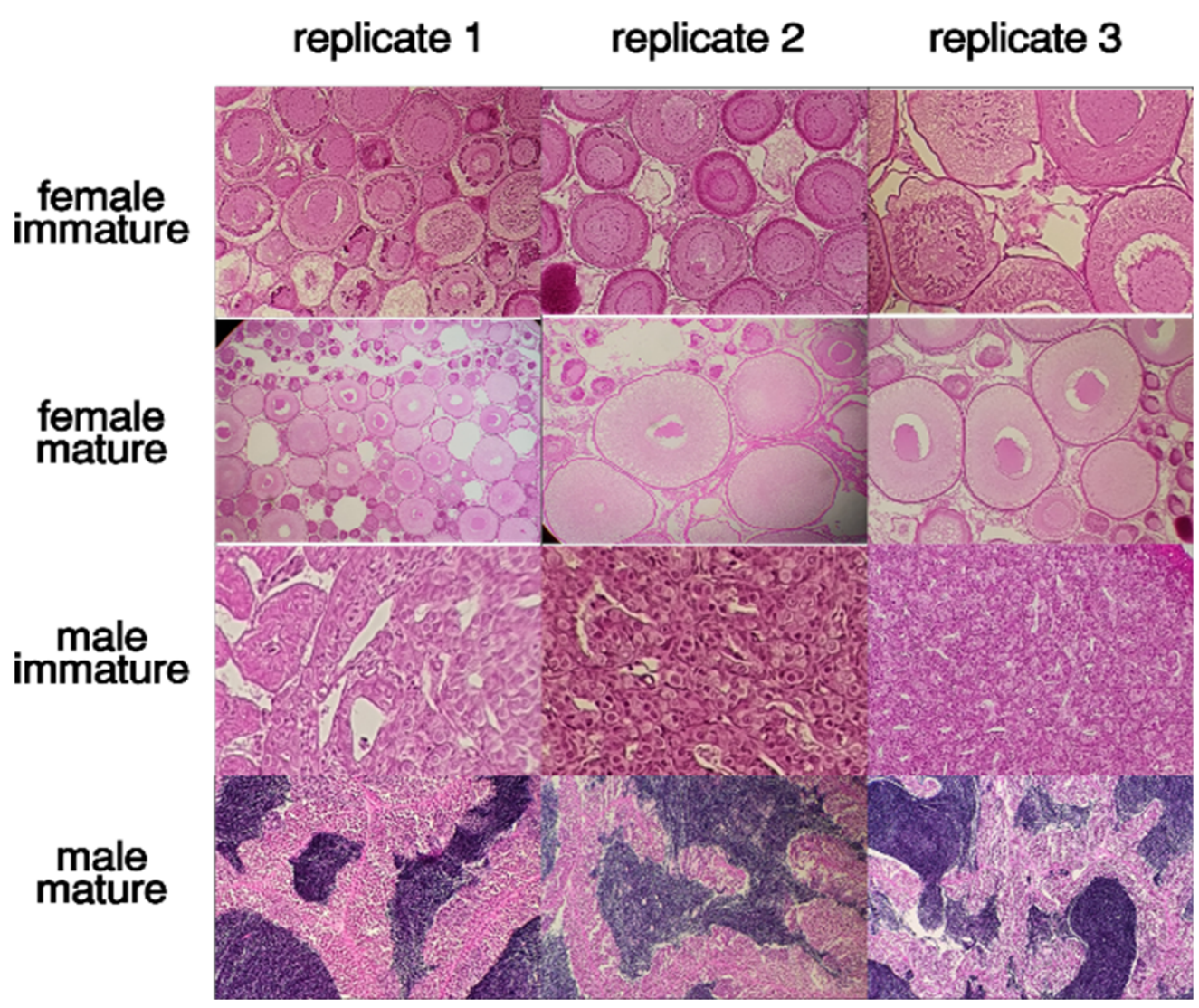

Histology images confirming the sexual maturation status of the 12 Atlantic salmon individuals used in our study.

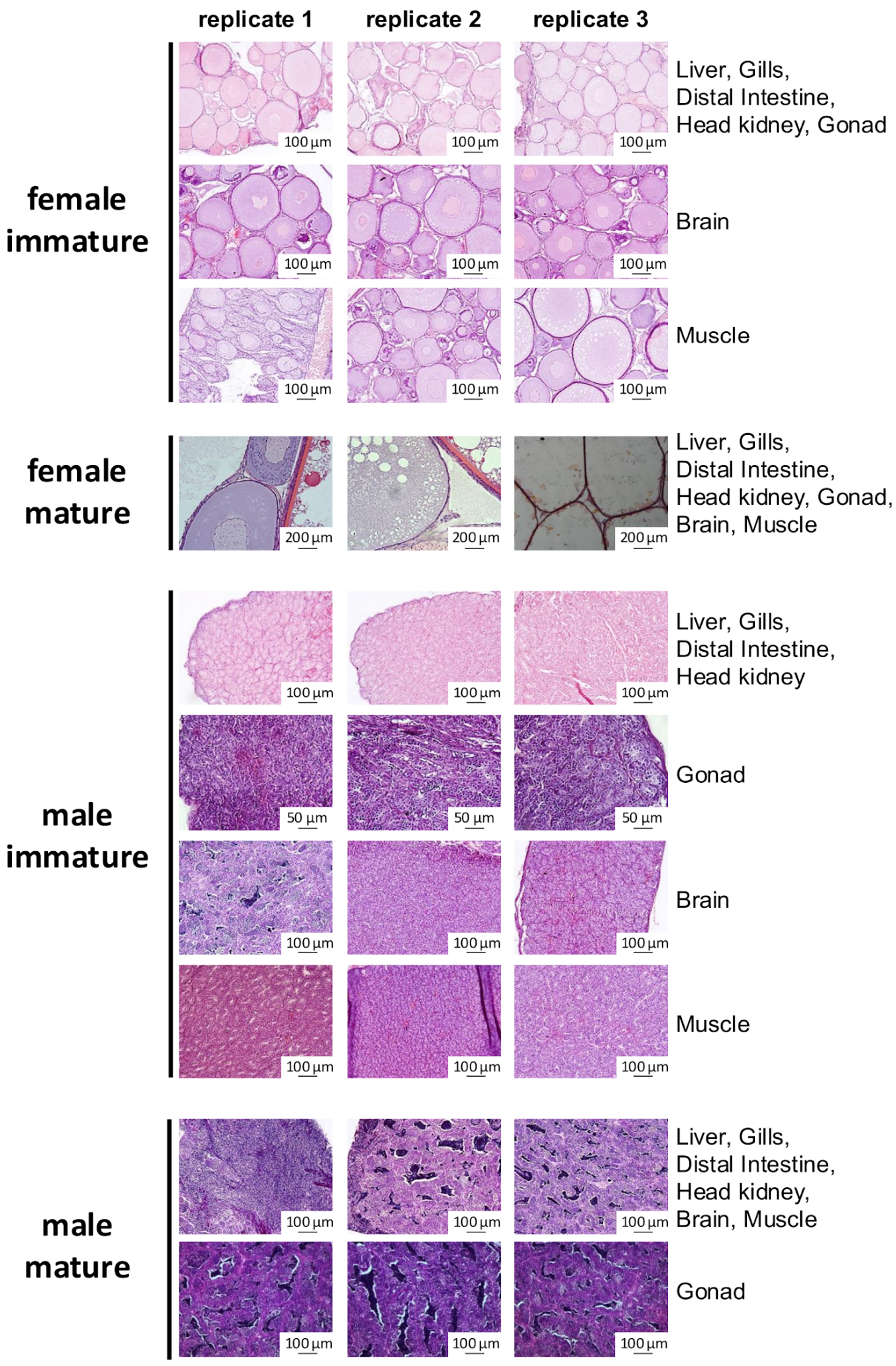

Histology images confirming the sexual maturation status of the 30 rainbow trout individuals used in our study.

Supplementary Text 2: DevMap and BodyMap RNA extraction protocols

Executive summary: This protocol describes the total RNA extraction methods used for Atlantic salmon and rainbow trout for DevMap and BodyMap studies.

S2.1. RNA extraction from salmonid embryos (DevMap)

S2.1.1. Materials and Reagents

- 1.5 mL Eppendorf tubes with safety lock
- Trizol (or equivalent)
- Sterile homogenization beads
- Tissuelyser or equivalent
- Water from egg trays
- Precipitation solution (recipe below)
- Isopropanol, anhydrous
- 1-Bromo-3-Chloropropane
- Nuclease-free water (NF-H2O)
- Ethanol 70%
- Sodium chloride (NaCl)
- Sodium citrate sesquihydrate
- RNAse-AWAY or equivalent
- MilliQ water

Precipitation solution: 100 mL of 1.2M NaCl and 0.8M sodium citrate sesquihydrate. To prepare, dissolve 7g NaCl in 50 mL NF-H_2O, then add 21.05 g sodium citrate sesquihydrate, then dissolve by heating and stirring. Filter through 0.2 µM syringe filter. Aliquot and store at -20ºC. Use only pre-autoclaved and RNA-AWAY washed bottles.

S2.1.2. Protocol:

1. Label required number of 1.5 mL Eppendorf tubes depending on the required number of biological replicates. No more than 3 eggs per tube should be used in salmonids.
2. Fill each tube with the required amount of homogenization beads.

Note: The amount of beads depends on the type of beads used. We successfully used 2x 3mm tungsten beads and 500 µL of both 1 mm Zirconium beads or 1 mm glass beads. It may be necessary to monitor the homogenate and use more beads/homogenization time if necessary.

3. In a flow hood, add 1 mL of TRIzol™ or equivalent to each tube. Store tubes in fridge while collecting embryos.

Note: The number of tubes used will depend on sampling strategy.

4. Place the required number of eggs for all biological replicates in water from egg trays within a Petri dish on ice.

Note: Using water from egg trays to temporarily keep the eggs while sampling allows for easy identification of dead/dying eggs, which will turn white and opaque and should not be sampled. If eggs are kept in PBS they will not turn white when they die, instead the chorion will turn more transparent and the yolk/embryo mass will clump into a ball. This makes it more difficult to tell between dead and alive eggs.

5. Using watchmaker forceps, transfer each egg to an empty petri dish, and puncture it with a pair of closed watchmaker forceps (pair 1), while holding the egg with a second pair of watchmaker forceps (pair 2). Do not remove forceps from the puncture site, instead, continue puncturing until pair 1 comes out through the other side.
6. Place egg over an open tube while still held by both pairs of forceps, and tear open the puncture by opening pair 1, dechorionating and depositing the egg in the tube at the same time.
7. Add no more than three punctured eggs per tube. Make sure tubes close tightly.

Note: this is to avoid overfilling of tubes due to the large egg size in salmonids. Egg puncturing is not necessary if using heavy 3mm tungsten beads.

8. Homogenize using a Tissuelyser at a setting of 30Hz for 6 mins.
9. Store tubes at -80ºC until RNA extraction or proceed with following steps.
10. Prepare (or thaw) the precipitation solution and label 3 additional Eppendorf tubes per sample.
11. Transfer the homogenate (without beads) to a new tube.
12. Centrifuge at 12,000g, for 10 mins at 4ºC.
13. Transfer supernatant to a new tube.

Note: A pellet will form at the bottom of the tube, while a layer of oil will form at the top. Extract only the middle layer between the pellet and the oil. If the oil is not visible, avoid aspirating trace amounts by keeping the pipette tip well below the surface.

14. Add 100 µL of 1-Bromo-3-Chloropropane.
15. Vortex vigorously for 15 sec. Incubate for 30 mins at room temperature.
16. Add 100 µL of previously prepared precipitation solution and 100 µL of anhydrous Isopropanol to each separate and empty tube.

Note: The precipitation solution and the isopropanol do not mix, hence one extra tube per sample is used here to avoid adding solution in incorrect proportions.

17. Centrifuge samples at 20,000g, for 15 mins at 4ºC.
18. Carefully place samples on ice without disturbing newly formed layers, and transfer 2x 200 µL of aqueous phase, slowly and carefully, into new empty 1.5 mL Eppendorf tubes. Use pipette tip with blunt end and keep tip in the middle, as far away from the interphase, the meniscus and the sides of the tube as possible. If there is debris stuck to the sides of the tube, stop pipetting before the meniscus level reaches the debris.
19. Add 200 µL of precipitation solution to each of the tubes where the aqueous layer was placed. Gently invert 6 times.
20. Incubate at room temperature for 10 mins or incubate overnight at -20ºC for increased RNA recovery.
21. Centrifuge samples at 20,000g for 10 mins at 4ºC.
22. Remove supernatant and resuspend in 1000 µL of 70% ethanol.
23. Incubate on rotation for 30 mins.
24. Repeat steps 21-23. Then, repeat step 21 again.
25. Remove supernatant, including remaining ethanol with a p-10 pipette.
26. Air dry pellets for 10 mins at room temperature.
27. Resuspend in 20-100 µL NF-H2O (depending on size of pellet).
28. Incubate at room temperature for 60 mins, flicking the tubes every 10 mins.
29. Assess RNA concentration using a Qubit fluorimeter
30. Dilute samples as necessary with NF-H2O, aiming for ~400 ng/µL.
31. Assess the quality of sample using Tapestation (to obtain RNA integrity numbers) and Nanodrop (to assess for purity).
32. Store your sample at -80ºC.

S2.2. RNA extraction from Atlantic salmon tissues (BodyMap)

A Qiagen column-based RNA extraction kit was used (miRNeasy Mini kit, #217004) following the manufacturer's recommendations, with an optional DNAse treatment and column drying as instructed by the manufacturer. Extracted RNA was stored at -80°C.

S2.2. RNA extraction from rainbow trout tissues (BodyMap)

Summary: In our study, rainbow trout RNA extraction followed the protocol of Chomczynski et al. [4]. The tissues are ground into the TRIzol™ solution containing guanidium isothiocyanate, phenol and sodium acetate. After addition of chloroform, the samples are centrifuged to separate the total RNA (upper aqueous phase). The aqueous phase is extracted, and the total RNA is reconcentrated by conventional alcoholic precipitation.

S2.2.1. Materials and Reagents

- 2 mL Eppendorf tubes
- Precellys (Bertin Instruments, # P000669-PR240-A)
- TRIzol™: TriReagent (Euromedex, #TR118)
- Chloroform (Carlo Erba, #438603)
- Ethanol (Carlo Erba, #4146012)
- Isopropanol (Sigma, #33539)
- CK14 ceramic beads 1.4 mm, tubes 2.0 mL (#P000912-LYSK0-A.0 OZYME)
- 75% EtOH

S2.2.1. Protocol:

1. Place the tissue in a Precllyus-specific 2 mL grind tube with ceramic beads.
2. Add 1 mL TRIzol™ for each 200 mg tissue.
3. Grind tissue with Precellys (2 times 15 sec with 30 sec break).
4. Transfer the homogenized tissue to a 2 mL Eppendorf tube.
5. Add 200 µL chloroform (for 1 mL TRIzol™) and quickly mix by manual inversion for 15 sec (without vortex).
6. Incubate for 5-10 min at room temperature.
7. Centrifuge at 12 000g, for 15-30 mins at 4°C.
8. Collect the colourless upper phase and transfer it in a new Eppendorf tube.
9. Add isopropanol: 0.5 mL per 1 mL TRIzol™ and vortex quickly.
10. Incubate for 2 h at -20°C.
11. Centrifuge at 12,000g, for 25 mins at 4°C.
12. Remove the supernatant.
13. Wash the pellet with 1 mL of 75% EtOH (per 1 mL TRIzol™).
    13.1. Add 75% EtOH at the opposite wall of the pellet.
    13.2. Centrifuge at 15 000g, for 15 mins at 4°C.
    13.3. Remove carefully the supernatant.
14. Resuspend the pellet in 75% EtOH (0.5 mL per 1 mL TRIzol™).
    14.1 Add 75% EtOH at the opposite wall of the pellet.

14.2. Centrifuge at 12 000g, for 15 mins at 4°C.

14.3. Carefully remove the supernatant.

14.4. Spin down the tubes to remove the remaining supernatant.

14.5. Leave the lid open to let the pellet dry (2-5 mins).

15. Dissolve the pellet in RNAse-free H_2O.

16. After quality check by Nanodrop and Bioanalyzer, store at -80°C.

Supplementary Text 3: DevMap & BodyMap Omni-ATAC-seq

Executive summary: This protocol describes how we performed the assay for transposase-accessible chromatin with sequencing (ATAC-seq) in embryos (DevMap) and frozen tissues (BodyMap), adapted from the Omni-ATAC method [5]. It is divided into 3 main steps: i) nuclear preparation, ii) transposition and iii) PCR with size selection. Due to a diversity of input material, the first step (nuclear preparation) is different between DevMap and BodyMap, but also contains several tissue-specific modifications within BodyMap. All protocol alterations have been added below the main reference protocol.

Nuclear preparation for BodyMap consists of tissue disruption, filtration and homogenization, release of nuclei from cells, then purification and concentration of nuclei suspension via density gradient centrifugation. A different method of nuclei extraction was used for embryos, with ST and TST buffers developed previously for nuclei preparations [4]. An aliquot of isolated nuclei is used to estimate nuclei concentration and to validate nuclei quality before transposition. The transposition reaction is sensitive to the ratio of transposase to nuclei used and requires prior optimizations depending on the sample type. Following transposition, the DNA is purified and frozen.

We performed the abovementioned steps in tissue-specific batches, then once a larger number of eluted DNA was accumulated, the samples were PCR-amplified and size-selected (180-700bp). It is important to assess by qPCR the optimal amount of PCR cycles for each tissue. Therefore, for all the PCR steps, we recommend using PCR tubes and not plates, then re-order them according to the amount of PCR cycles. Size selection is not required but often needed, either to remove high molecular weight fragments or left-over primers or primer dimers. The profile of each library was assessed by High Sensitivity Bioanalyzer or similar, with a recommended loading concentration of 1-2 ng/ µL.

S3.1. Modifications to Omni-ATAC for DevMap

Notes: The DevMap-specific modification starts with extracted embryos as input material, from which the cells are isolated (section S1.1). After the nuclei have been extracted from isolated cells (as described below), we recommend following the reference Omni-ATAC protocol from the transposition step onwards. There are also developmental-stage specific protocol adjustments, which have been divided into different sections for i) blastulation to early gastrulation stages, and ii) mid-gastrula to pharyngula stage. Additional comments within sections below describe any additional minor adjustments for closely related embryo stages.

3.1.1. Materials and Reagents

- 0.25% Trypsin-EDTA

- 15 mL Falcon tubes
- Rotator system
- Dissection microscope (optional)
- FBS (Heat inactivated)
- DMEM
- DMSO
- 40 µm filters (Thermo Fisher Scientific, catalog no. 08-771-2)
- 2 mL cryotubes
- Mr Frosty cooling chamber (Nalgene)
- Isopropyl alcohol

3.1.2. Buffer recipes

2x ST stock solution (Salt-Tris buffer, 10 mL)

- 146 mM of NaCl (292 µL)
- 10 mM of Tris-HCl pH 7.5 (100 µL)
- 1 mM of CaCl2 (10 µL)
- 21 mM of MgCl2 (210 µL)
- Nuclease-free water (9,388 µL)

Notes: Prepare the day before or fresh and keep on ice

1x ST solution (Salt-Tris buffer, 10 mL)

- 2x ST (5 mL)
- Nuclease-free water (5 mL)

TST working solution (10 mL)

5 mL of 2x ST stock solution

300 µL of 1% Tween-20

50 µL of 2% BSA

4.65 mL Nuclease-free water

1x proteinase inhibitor cocktail (PIC) tablet (Sigma-Aldrich, 11836170001)

Note: Prepare fresh and keep on ice

3.1.3. Cell isolation for Blastulation to early gastrulation (follows end of section S1.1 above).

1. Pipette the 900 µL suspension containing the extracted embryos up and down 10 times. Use a wide bore pipette tip and a p1000 pipette.
2. Vortex at a slow speed for 15 sec.

3. Pipette the 900 µL suspension containing the extracted embryos up and down 10 times. Use a wide bore pipette tip and a p1000 pipette.
4. Check solution is homogeneous with no clumps. If clumps are observed, repeat step 1.
5. Transfer solution to a 2 mL cryotube.
6. Add 10 µL of Trypan blue to a 1.5 mL Eppendorf tube.
7. Pipette sample up and down 3 times with a bore tip p1000 pipette set to 500 µL. Take a 10 µL aliquot for cell counting and add it to the tube with Trypan blue. Pipette up and down 3 times.

Note: This step is meant to homogenize the sample before taking an aliquot for cell counting, and only needs to be done with one replicate. Trypan blue stain needs an incubation period of 5 min; leave this tube for cell counting on ice while continuing to the next step with the sample tube.

8. Add 500ul of DMEM and 400ul of FBS to each sample tube.
9. Add 100 µL of DMSO. Invert tube 4 times. Perform this step as quickly as possible for all replicates at the same time to minimize cytotoxicity of DMSO.
10. Store in a pre-chilled Mr Frosty cooling chamber and transfer to a -80ºC freezer for up to 4h
11. Recover tube containing cells and Trypan blue. Assess cell numbers per mL and percentage of live cells using a hemocytometer.
12. Store cryopreserved cells at -80ºC.

3.1.4. Cell isolation for mid-gastrula to pharyngula stage (follows endpoint of section S1.1 above).

1. Remove supernatant from 900 µL PBS suspension containing extracted embryos and add required volume of cold Trypsin-EDTA indicated below. It may be required to use a 15 mL Falcon tube for the trypsin digestion when higher volumes of Trypsin EDTA are required:
    - Mid gastrula: 1,000 µL for 30 gastrulas (33 µL per gastrula)
    - Early somitogenesis: 1,000 µL for 20 embryos (50 µL per embryo)
    - Mid somitogenesis: 2,000 µL for 10 embryos (200 µL per embryo)
    - Late somitogenesis: 2,000 µL for 10 embryos (200 µL per embryo)
    - Pharyngula: 1,000 µL per embryo
2. Rotate for required time indicated below, 50RPM at room temperature:
    - Mid gastrula: 5 min
    - Early somitogenesis: 5 min, monitor digestion and increase time if necessary.
    - Mid somitogenesis: 10 min
    - Late somitogenesis 20 min; observe after 15min and decide if more time required.
    - Pharyngula: 30 min to 1h, with constant monitoring of digestion required. Observe tube against bright background to better observe clumps.
3. Use dissection microscope to estimate digestion progress. Go back to previous step if clumps are observed.

4. Add 500 µL of cold heat inactivated FBS per 1,000 µL of Trypsin EDTA

Note: If using a 15 mL Falcon tube for digestion, split homogenate into 1.5 mL Eppendorf tubes for fixed angle centrifugation.

5. Centrifuge 300g for 6 min at 4ºC.
6. Remove supernatant and resuspend in 1000 µL of PBS.
7. Centrifuge 300g for 6 min at 4ºC.
8. Remove supernatant and resuspend in 900 µL of cryopreservation solution without DMSO (45% DMEM, 55% FBS).
9. Pass homogenate through a 40 µm filter into a 2 mL cryotube.
10. Add 10 µL of Trypan blue to a 1.5 mL Eppendorf tube.
11. Pipette sample up and down 3 times with a bore tip p1000 pipette set to 500 µL. Take a 10 µL aliquot for cell counting and add it to the tube with Trypan blue. Pipette up and down 3 times.

Note: This step is meant to homogenize sample before taking an aliquot for cell counting and only needs to be done with one replicate. Trypan blue stain needs an incubation period of 5 min; leave this tube for cell counting on ice while continuing to the next step with the sample tube.

12. Add 100 µL of DMSO, close the cryotube and invert several times. Perform this step as quickly as possible for all replicates at the same time to minimize cytotoxicity of DMSO.
13. Place in Mr Frosty Cooling chamber and store at -80ºC for a minimum of 4 h.
14. Recover tube containing cells and Trypan blue. Assess cell numbers per mL and percentage of live cells using a haemocytometer.
15. Store cryopreserved samples at -80ºC.

3.1.5. Nuclei extraction from isolated cells (all stages)

1. To 400 µL of cryopreserved cells, add up to 1 mL, then pipette up and down until defrosted.
2. Centrifuge at 500g for 5 min at 4°C.
3. Discard supernatant and resuspend pellet in 1 mL ice cold PBS + PIC.
4. Centrifuge at 500g for 5 min at 4°C. Discard supernatant and resuspend pellet in 1 mL ice cold TST solution.
5. Leave on ice for 5 min, pipetting gently up and down.
6. Filter through a 40 µm Falcon cell strainer using the rubber end of a syringe to mash the top of the filter and add 1 mL of TST to wash the well and filter.
7. Bring the volume up to 5 mL with 3 mL of 1X ST buffer.
8. Transfer the sample to a 15 mL conical tube and centrifuge in a swinging bucket centrifuge at 500g for 5 min at 4°C.
9. Resuspend the pellet in 1X ST buffer. Resuspension volume is dependent on the size of the pellet, usually within the range of 100-200 µL resuspended to 1 mL.

10. If nuclei clumping is observed, refilter through 40μm filter.
11. Count nuclei with hemocytometer.
12. Put forward 50,000 to 75,000 nuclei for the next step. Make up the solution to 1 mL with 1X ST buffer.
13. Centrifuge the nuclei aliquot at 800g for 10 min at 4ºC, discard the supernatant and add transposase reaction mix to the nuclei. Follow the BodyMap reference protocol (section 3.2) from the transposition step onwards but use 1.25 μL of transposase instead of 2.5 μL.

S3.2. Omni-ATAC-seq on frozen tissue (BodyMap)

S3.2.1. Materials and Reagents

- 40-60 mg of snap-frozen tissue (section S1.2)
- Milli-Q water
- Homogenization Buffer Unstable Solution, 1x (see recipe)
- 50%, 29%, 40% Iodixanol solution (see recipe)
- ATAC-RSB-Tween-20 (see recipe)
- Phosphate-buffered Saline Solution (PBS) (Sigma-Aldrich, P5368)
- Hoescht Dye Solution (ThermoFisher, 62249) or Trypan Blue (Sigma-Aldrich, T8154)
- Illumina Tagment DNA Enzyme (transposase) and Buffer (Illumina, 20034197)
- 1% (v/v) Digitonin, diluted in purified water (Promega G944A)
- 10% Tween-20 (Sigma-Aldrich, 11332465001)
- Nuclease-Free water (ThermoFisher, AM9937)
- MinElute® PCR Purification Kit (Qiagen, 28004)
- Qubit™ ds DNA HS Kit (ThermoFisher, Q32854)
- NEBNext® Ultra™ II Q5® Master Mix (NEB, M0544S)
- IDT for Illumina DNA/RNA UD Indexes Set (Illumina, 20027213)
- SYBR™ Green I Nucleic Acid Gel Stain, 25x, diluted in water (Invitrogen, S7563)
- Agencourt AMPure XP beads (Beckman Coulter, A63880)
- 80% (v/v) Ethanol (VWR, 20821)
- TET Buffer (see recipe)
- Bioanalyzer High Sensitivity Kit (Agilent Technologies, 5067-4626)
- 2 mL Tissue douncer (VWR, KT885303-0002)
- Pestle A for 2 mL tissue douncer (VWR, KT885301-0002)
- Pestle B for 2 mL tissue douncer (VWR, KT885302-0002)
- 70 μm cell strainer (Corning, 431751)
- 40 μm cell strainer (Corning, 431750)

- 50 mL Falcon tube (Sarstedt, 62.547.254)
- 5 mL Eppendorf Tube snap cap (VWR, 89429-306)
- Centrifuge with swinging bucket for 5 mL tubes and cooling option (Eppendorf, 5810R)
- 1.5 mL DNA LoBind Tube (VWR, 525-0130)
- Microcentrifuge (Beckman Coulter, 20R)
- Counting chamber BLAUBRAND® Thoma pattern (Sigma-Aldrich, BR718020)
- Inverted Microscope (Zeiss, AXIO vert.A1)
- Thermomixer (Eppendorf, 5382000015)
- Qubit 4 Fluorometer (Invitrogen, Q33226)
- Qubit™ assays tubes (ThermoFisher, Q32856)
- 0.2 mL PCR Tubes with Flat Caps (ThermoScientific, AB-0620)
- GeneAmp® PCR Machine (Applied Biosystems, 9700)
- Hard-Shell PCR 96-well Plates (BioRad, HSP9655)
- Plate Microseals® 'B' (BioRad, MSB1001)
- qPCR machine (BioRad, CFX96)
- Vortex Mixer (Gilson, 36110750)
- Magnetic rack (Life Technologies, 12321D)
- BioAnalyzer (Agilent Technologies, 2100)
- Mortar and pestle (Sigma-Aldrich, Z247464-1EA) (muscle-specific)

S3.2.2. Buffer recipes

<u>Homogenization Buffer Unstable Solution, 1X (2 mL/sample)</u>

- 6X Homogenization Buffer Unstable Solution (see recipe) (333.33 µL)
- 320 mM Sucrose solution (see recipe) (640 µL)
- 0.1 mM EDTA (Sigma-Aldrich, 03690-100 mL) (0.4 µL)
- 0.30% (v/v) of Nonidet P40 Substitute 10% (Roche, 11332473001) (60 µL)
- 0.5 mM Spermidine (Sigma-Aldrich, S2626) (1 µL)
- 0.15 mM Spermine (Sigma-Aldrich, S3256) (2 µL)
- Water (963.27 µL)

Note: Prepare fresh and keep on ice.

<u>Homogenization Buffer Unstable Solution, 6X (910 µL/sample)</u>

- 12X Protease Inhibitor Cocktail Solution (see recipe) (454.19 µL)
- 6X Homogenization Buffer Stable (454.19 µL)
- 0.1 mM PMSF (Sigma-Aldrich, 93482-50 ML-F) (1.51 µL)

- 1mM beta-mercaptoethanol (Sigma-Aldrich,M6250) (0.11 µL)

Note: Prepare fresh under fume hood and keep on ice.

Sucrose solution, 1M

- Sucrose dissolved in water and sterilized by filtration using a 0.2 µm filter (Sarstedt)

Note: Store at 4°C for up to several months.

Protease Inhibitor Cocktail Solution, 12X (833 µL/sample)

- EDTA-Free Protease Inhibitor Cocktail (PIC) tablets (Sigma-Aldrich, 11836170001), dissolved in 6X Homogenization Buffer Stable (833 µL)

Note: Prepare fresh and keep on ice.

Homogenization Buffer Stable, 6X (100 mL)

- 30 mM $CaCl_2$ (Sigma-Aldrich, C5670) (3 mL)
- 18 mM $Mg(Ac)_2$ (Sigma-Aldrich, 63052) (1.8 mL)
- 60 mM Tris-HCl pH 7.8 (Sigma-Aldrich, T2569) (6 mL)
- Water (89.2 mL)

Note: Store at room temperature for up to several months

ATAC-RSB (50 mL)

- 10 mM Tris-HCl pH 7.4 (Sigma-Aldrich, T2194) (500 µL)
- 10 mM NaCl (Sigma-Aldrich, S3014-500G) (100 µL)
- 3 mM $MgCl_2$ (Sigma-Aldrich, M1028) (150 µL)
- Water (49.25 mL)

Note: Store at 4°C for up to several months.

TET Buffer (16.5 µL/sample, 100 samples)

- 10 mM Tris-HCL pH 8.0 (Sigma-Aldrich, T2694) (16.5 µL)
- 1 mM EDTA pH 8.0 (Sigma-Aldrich, 03690) (3.3 µL)
- 0.05% (v/v) Tween-20 (Sigma-Aldrich,11332465001) (8.25 µL)
- Water (1621.95 µL)

Note: Store at 4°C for up to several months.

50% Iodixanol solution (800 µL/sample)

- 6X Homogenization Buffer Unstable Solution 1X (133.3 µL)
- 50% (v/v) Iodixanol OptiPrep (Sigma-Aldrich, D1556) (666.6 µL)

Note: Prepare fresh and keep on ice.

29% Iodixanol solution (1200 µL/sample)

- 6X Homogenization Buffer Unstable Solution 1X (200 µL)

- 160 mM Sucrose (see recipe) (192 µL)
- 29% (v/v) Iodixanol OptiPrep (Sigma-Aldrich, D1556) (580 µL)
- Water (228 µL)

Note: Prepare fresh and keep on ice.

<u>40% Iodixanol solution (1200 µL/sample)</u>

- 6X Homogenization Buffer Unstable Solution 1X (200 µL)
- 160mM Sucrose (see recipe) (192 µL)
- 40% (v/v) Iodixanol OptiPrep (Sigma-Aldrich, D1556) (800 µL)
- Water (8 µL)

Note: Prepare fresh and keep on ice.

<u>ATAC-RSB-TWEEN (1 mL/sample)</u>

- ATAC-RSB (see recipe), 1X (990 µL)
- 0.1% (v/v) Tween-20 (1 0 µL of 10% Tween-20)

Note: Prepare fresh and keep on ice.

S3.2.3. Tissue disruption and nuclei isolation

Note: To start, pre-chill the swinging bucket centrifuge and micro-centrifuge to 4°C, pre-heat the thermomixer to 68°C and pre-chill the mortar on dry ice (for muscle and gill only). Keep samples on ice while working.

1. Place frozen tissue into ice-cold 2 mL douncer containing 1.2 mL cold 1X homogenization buffer (1x HB).

Note: Allow frozen tissue to thaw completely in buffer (max. 5 min). The volume of 1x HB can be adjusted according to the amount of tissue (0.8-2.0 mL).

2. Dounce the tissue with pestle A and B (up to 5 strokes each depending on tissue type) until obtaining a homogenized solution.

Note: It is important to visually control the level of homogenization. Different people may apply different strengths during the process, so try to minimise associated impacts.

3. Pre-clear the solution by filtering it through a 70µm (or 40µm) cell strainer suspended in a 50 mL Falcon tube.
4. Transfer 400 µL of filtrated suspension to each of two new 5 mL Eppendorf tubes.

Note: When tissue is limited, adjust the initial volume of 1x HB so there are no leftovers of homogenate after splitting it in two 400 µL volumes at this stage

5. Add an equal volume (400 µL) of 50% iodixanol solution and mix by pipetting (to create a 25% mixture).
6. Take 600 µL of 29% iodixanol solution and slowly dispense it under the 25% mixture.

Note: Kim-tech or other fine paper can absorb excess drops of solution from the pipette tip before dispensing. A reverse pipetting technique can help avoid bubbles and mixing of layers. Slow motion of the pipette in and out of the tube can avoid abrupt volume displacement and mixing of layers.

7. Take 600 µL of 40% iodixanol solution and slowly dispense it under the 29% mixture.
8. Transfer 5 mL tubes to a pre-cooled (4°C) swinging bucket centrifuge and centrifuge at 3,220g for 25 min with brakes set to 'off'.

Note: Density gradient centrifugation is performed in a swinging bucket centrifuge with brakes off to avoid collapse of the gradient layers. Without brakes, the centrifuge needs an additional 8-9 min to stop. If possible, centrifugation at higher speed is better, as it may improve the purity of nuclei.

9. Collect the nuclei band which should be visible at the interface between 29%-40% iodixanol layers and transfer it to a common 1.5 mL LoBind Eppendorf tube.

Note: This can be done by careful removal of volume from the top layer (approximately 1250 µL until the nuclei band) or targeting directly the nuclei band.

10. Add 1000 µL of ATAC-RSB-Tween buffer to the tube containing nuclei and mix.
11. Centrifuge in a pre-cooled centrifuge 500g for 10min at 4°C.

Note: Note the orientation of the tube so that you can predict the location of the pellet.

12. Remove and discard the supernatant.
13. Resuspend the nuclei pellet in 50 µL of cold PBS.
14. Take an aliquot of 2 µL from nuclei suspension, add 8 µL PBS and mix with 10 µL of dye (Hoescht or Trypan Blue). Count nuclei using hemocytometer and assess the integrity of sample (a majority of the nuclei should be rounded to slightly oval, with intact membrane, showing no shrinkage or dilation, and without blebbing).

Note: If there is too much material to accurately count, prepare a new dilution but maintain the ratio of 10 µL diluted nuclei suspension (in PBS): 10 µL dye. If you observe a lot of tissue debris, add 100 µL PBS to your nuclei suspension, filtrate through 40 µm strainer and recount.

15. Dilute the appropriate volume of your original nuclei suspension in a new 1.5 mL tube with cold PBS to obtain 16.5 µL at 3 000 nuclei/µL (approx. 50 000 nuclei).

S3.2.4. Transposase reaction and clean-up

1. Prepare the following transpose reaction mix in a new tube:

25.0 µL	2X TD buffer
2.5 µL	Illumina Transposase
0.5 µL	1% Digitonin

0.5 µL	10% Tween-20
5.0 µL	Nuclease-free water
33.5 µL	Total volume

Immediately add 33.5 µL transposase reaction mix to 16.5 µL of nuclei suspension and mix by pipetting up and down 6 times.

2. Incubate the reaction at 37ºC for 30 min in a Thermomixer with 1000 RPM mixing. Then, proceed immediately with the next step.
3. Purify the resulting DNA fragments with MinElute PCR purification kit (follow manufacturer instructions).

Note: Eluted DNA can be stored at -20ºC until ready to amplify.

4. Measure DNA concentration with HS Qubit or equivalent.

S3.2.5. Library amplification

1. Thaw transposed DNA, indexes, NEBNext® Ultra™ II Q5® Master Mix and SYBRGreen on ice.
2. Prepare the following PCR reaction in PCR tubes.

5.0 µL	IDT for Illumina DNA/RNA UD Indexes
25.0 µL	NEBNext® Ultra™ II Q5® Master Mix
20.0 µL	Sample (transposed DNA)
50.0 µL	Total volume

3. Short-spin the PCR tubes to collect all the liquid to the bottom.
4. Run the ‘Initial PCR amplification’ program (choose the pre-heat option for lid and set to 100ºC).

72ºC for 5 min	x 1
98ºC for 30 sec	x 1
98ºC for 10 sec	x 5

63ºC for 30 sec	
72ºC for 30 sec	
4ºC hold	x 1

5. Set up 15 µL qPCR reactions in a qPCR plate to determine the appropriate number of additional cycles needed. Prepare a pre-mix of nuclease-free water, NEBNext® UltraTM II Q5® Master Mix and 25X SYBRGreen for n+2 samples (n samples + 1 NTC + 1 additional), distribute 9 µL in each well and then add IDT for Illumina DNA/RNA UD Indexes and the sample as per below:

3.76 µL	Nuclease-free water
5.00 µL	NEBNext® Ultra™ II Q5® Master Mix
0.24 µL	25x SYBRGreen (diluted in water)
5.00 µL	Sample (Initially amplified DNA or water for NTC)
1.00 µL	IDT for Illumina DNA/RNA UD Indexes
15.00 µL	Total volume

6. Seal, vortex and centrifuge the qPCR plate 280g for 1 min at room temperature.
7. Run the qPCR as follows:

98ºC for 30 sec	x 1
98ºC for 10 sec	x 20
63ºC for 30 sec	
72ºC for 1 minute	
4ºC hold	x 1

8. Assess the amplification profiles and determine the required number of additional cycles to amplify. The number of cycles should equal 1⁄4 of the maximum achieved fluorescence. For each curve, subtract the baseline value from the end-point value. Then, divide by 4 and check the number of cycles corresponding to that value. If the 1⁄4 value is between two cycles, choose the lower one.

9. Short-spin the PCR tubes containing your Initially amplified DNA (45 µL) and run the 'Final PCR amplification' program (see below Table) without adding anything else.

Note: After final PCR amplification (done as per below table), keep samples at 4ºC until the size selection step. It is possible to assess the profile of the libraries before that. To do so, use High Sensitivity Qubit kit to measure the concentration then dilute a 1 µL aliquot of your PCR product for Bioanalyzer profiling.

98ºC for 30 sec	x 1
98ºC for 10 sec	# of cycles determined individually for each sample by qPCR
63ºC for 30 sec	
72ºC for 1 minute	
4ºC hold	x 1

3.2.6. Library size selection

1. Thoroughly resuspend Ampure XP beads by vortexing for at least 1 min.
2. Add 0.53x volume of beads (i.e. 23.9 µL assuming 45 µL reaction) to the sample and mix by pipetting (avoiding bubbles).
3. Incubate at room temperature for 10 min and mix gently every 5 min.
4. Touch-spin the tubes and place them on the magnetic rack for 5 min (until the supernatant is clear of beads).
5. Transfer the supernatant to a new tube and add another 1.3x original volume of Ampure XP beads (58.5 µL assuming 45 µL reaction).
6. Mix well by pipetting, avoiding bubbles.
7. Incubate at room temperature for 15 min while mixing gently every 5min.
8. Place on the magnetic rack for 5 min (until the supernatant is clear of beads).
9. Remove and discard supernatant.
10. While the tubes are still on the magnet, and without disturbing the pellet, wash the beads with 100 µL of freshly prepared 80% ethanol. Remove ethanol and discard. Repeat this step again.
11. Remove the tubes from the magnet, touch spin the samples, place back on the magnet and carefully remove the last traces of ethanol.
12. Remove the samples from the magnet and allow the tubes to air dry with caps open (30 sec to 2 min, do not wait until you see small cracks in the beads).
13. Add 16.5 µL TET Buffer. Resuspend beads by pipetting.
14. Rehydrate at room temperature for a minimum of 2 min.
15. Place on the magnetic rack for 5 min (until the supernatant is clear of beads).
16. Transfer the supernatant (eluted DNA) to a LoBind Eppendorf tube or 96 well plate.

17. Measure DNA library concentration with Qubit High Sensitivity Kit.

18. Validate DNA fragment size with Bioanalyzer or Tapestation High Sensitivity DNA kit

Note: The DNA fragment size distribution should follow a nucleosome pattern (ca. 200bp-350bp-500bp peaks for libraries), like in the example provided immediately to the right. If you have an excess of primer dimers (narrow peak around 120 bp), do an additional clean up with MinElute PCR purification kit and elute in 10 µL. If you have an excess of long fragments in your samples (>1000 bp), do an additional round of bead purification.

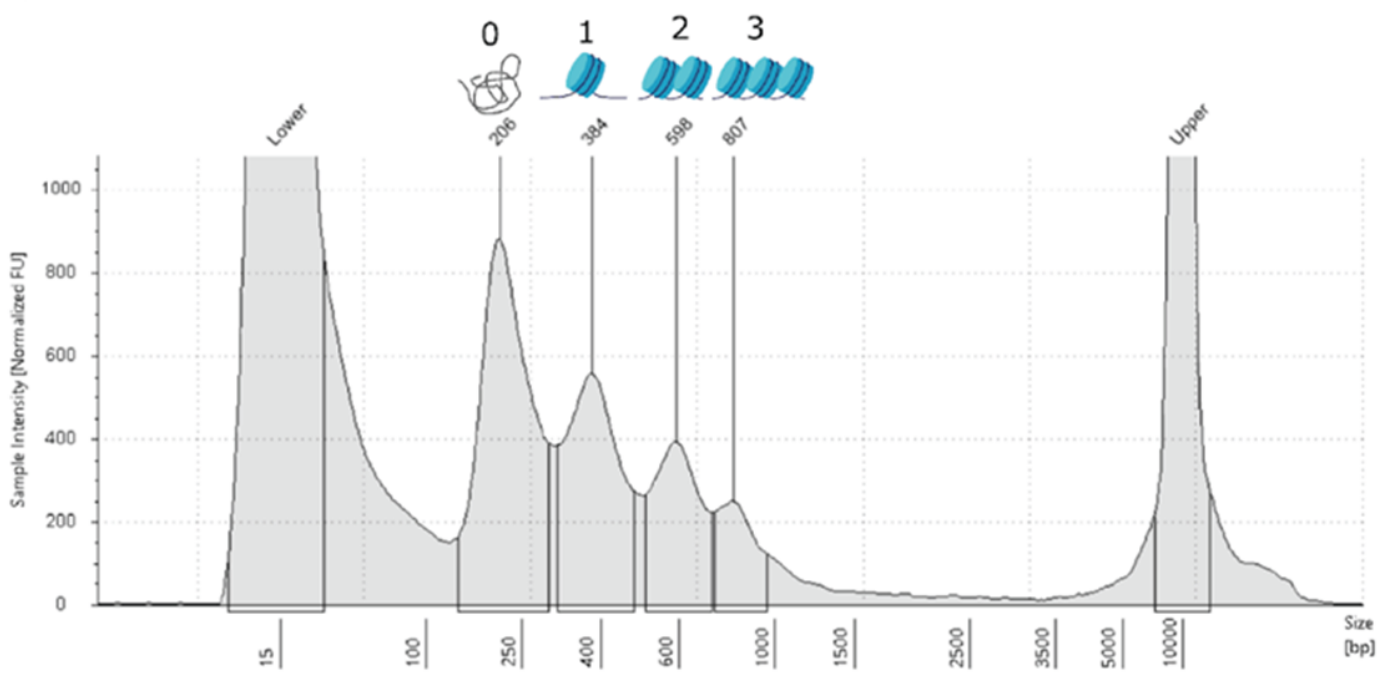

S3.2.6. Tissue-specific modifications

- *Brain, liver and immature gonads*: Follow reference protocol. Recommended to use 40-50mg total of pieces for liver or immature gonad and 20-30 mg for brain. No filtering is needed after homogenization.
- *Muscle*: Follow the reference protocol. The initial amount of tissue should be larger than for other tissues. Start with minimum 100 mg total of pieces. Larger amount of tissue also requires a larger volume of homogenization buffer (1xHB). Start with minimum 1.6 mL of 1xHB. Grind the tissue on a mortar previously chilled on dry ice until it is powdery or 'corn-flake' looking. Filter through 70 µm cell strainer and/or through 40µm if debris are still visible.
- *Mature male gonad*: Buffer preparation needs larger volumes of 1x HB (recommendation to prepare 9 mL more per sample) and 50% iodixanol solution (500µL more per sample) than reference protocol, due to additional washing steps and need for a second iodixanol centrifugation. 150-200 mg is a suitable weight of tissue. Recommended modifications as follows:
  1. Place the tissue in a 5 mL tube and add 1 mL of 1x HB. Gently squeeze the tissue with a pestle against the tube side to release the sperm remains, but do not break the tissue into smaller pieces.
  2. Vortex for at least 30 sec and pass through a 40 µm cell strainer suspended in a 50 mL Falcon tube. Add 200 µL of 1x HB over the strainer to rinse the tissue and place the tissue back to the 5 mL tube. Discard the filtrate containing mostly sperm (you can control the content of the filtrate by microscopy with Trypan blue coloration).
  3. Repeat steps 2 and 3 until the solution containing the tissue is clear.
  4. Cut the tissue into pieces and place it into an ice-cold 2 mL douncer containing 1 mL cold 1x HB. Homogenize as per reference protocol (step 2, section S3.2.3).
  5. Filter through a 70 µm cell strainer suspended in a 50 mL Falcon tube.
  6. Perform gradient centrifugation and recover nuclei band as per reference protocol (step 3-9, section S3.2.3).

7. Prepare a second gradient centrifugation adding an equal volume of 1x HB to the collected nuclei band (400 µL) resulting in a 15-20% iodixanol suspension and add carefully (avoid mixing layers) 500 µL of 50% iodixanol solution to the base of the 2 mL tube.
8. Centrifuge samples at 4ºC at 12,000g for 40 min (using soft stop).
9. Collect the nuclei band (around 100-150 µL) found between the top (15-20% iodixanol) and bottom (50% iodixanol) layers. Do not collect the pellet found at the bottom of the tube, which mostly contains sperm.
10. Continue as per reference protocol

Supplementary Text 4. DevMap & BodyMap ChIP-seq

Executive summary: This protocol describes how we performed Chromatin Immunoprecipitation with Sequencing (ChIP-seq) in embryos and frozen tissues in Atlantic salmon and rainbow trout.

Due to a diversity of input material, two methods were required. For all samples with low input material (DevMap samples and brain samples from BodyMap of rainbow trout), a µChIPmentation Kit for Histones (Diagenode #C01011011) was used together with Chromatin EasyShear Kit-High SDS (Diagenode # C01020012) with a few modifications to the manufacturer's recommended steps (as described below). For all the remaining BodyMap samples, a classic custom ChIP protocol was developed along with a V3 MicroPlex Library Preparation Kit (Diagenode; #C05010002). This protocol was optimized for each tissue separately. Hence, a reference BodyMap protocol is described below (as applied for liver tissue), followed by a list of the tissue-specific modifications applied. The same antibodies were used across all assays for the immunoprecipitation step. For both ChIP methods used, the input controls (i.e. lacking immunoprecipitation steps) from embryos from the same developmental stage (DevMap) or from the same tissue/sex/maturity (BodyMap) were equimolarly pooled before library preparation for sequencing.

S4.1. Crosslinking of embryos (DevMap)

This protocol describes a method used to crosslink proteins to DNA of whole salmonid embryos, with the purpose of subsequent ChIP-seq library preparation.

S4.1.1. Materials and Reagents

- 4% PFA solution in PBS (Thermo-Fisher #30525-89-4)
- Rotator wheel with 1.5ml conical tube inserts (such as IKA™ 0004015000)
- 1M glycine solution (Sigma Aldrich #67419)
- PBS (Gibco™ 10010023)
- Fixed angle centrifuge

Steps:

1. Pre-chill fixed angle centrifuge
2. Prepare chilled 1% PFA solution in PBS by diluting 4% stock PFA solution 1:3 in PBS. 875µL per tube will be required.

2. Starting from S1.1.3 (step 9) or S1.1.4 (step 11). Remove supernatant and resuspend in 875 µL of 1% PFA.

3. Put tubes on a rotator at 50 RPM for X min, at room temperature.

Note: X min = 8 mins for blastula and gastrula, 10 mins during segmentation stages, 15 mins post-segmentation stages.

5. Add 125 µL of 1 M glycine. Continue rotating for an additional 10 mins at 50 RPM, at room temperature.
6. Centrifuge at 300 RCF, for 6 min at 4ºC.
7. Discard supernatant and resuspend in PBS, then store at -80ºC

S4.2. ChIP-seq on crosslinked embryos using µChIPmentation (DevMap)

S4.2.1. Materials and Reagents

- µChIPmentation Kit for Histones (Diagenode #C01011011)
- Chromatin EasyShear Kit - High SDS (Diagenode # C01020012
- 24 unique dual indexes (UDI) for tagmented libraries Set I and Set II from Diagenode (#C0101134 and #C0101135)

Steps:

1. Thaw crosslinked embryo material on ice.
2. Wash once with 1 mL of ice-cold PBS.
3. Spin down. Remove PBS. Homogenize using 2 mL dounce with 1 mL homogenization buffer (PBS + 1x PIC, 20-30 pestle strokes).
4. Perform the cell lysis with Chromatin EasyShear Kit-High SDS, using 500,000 homogenized cells for 315 µL of tL1 lysis buffer with 1x PIC on ice for 20-30min.
5. Follow the manufacturer's recommendations of µChIPmentation Kit for Histones kit with following parameters of sonication: 500 µL, 3-7 cycles (1 cycle = 30sec ON & 30sec OFF), 0.65 mL Bioruptor® Microtubes (#C30010011) and with following modifications:
    - immunoprecipitation step performed with 100 ng of chromatin (37,000 cells) and 0.8-1 µg of antibody per IP
    - Tagmentation of immunoprecipitated samples: 37°C for 12 min, PCR amplification: 14-15 cycles

S4.2.2. Modifications of the manufacturer's protocol that apply only to the input sample:

6. Use 6 µL for input sample + 21 µL of ChIPmentation mix (1µL Tn5 + 19 µL tagmentation buffer + 1µL $MgCl_2$)
7. Mix with Protein A beads pellet (equivalent to 10 µL of washed Protein A beads)
8. Tagment for 15-17 min

9. Add 25 µL of 2x High Fidelity mix, continue with repair and reverse crosslinking as in the protocol
10. Magnetise beads and transfer supernatant in a new tube
11. Add 2 µL of diluted UDI primer pair
12. PCR using 14-15 cycles and purify
13. Purify with 1x volume AMPure beads (to remove the excess of adapters)

S4.3. ChIP-seq on frozen tissue (BodyMap)

S4.3.1. Materials and Reagents

- Snap frozen tissue (see tissue-specific protocol modifications)
- PBS Plus (see recipe below)
- Hoescht Dye Solution (ThermoFisher, 62249) or Trypan Blue (Sigma-Aldrich, T8154)
- 37% (v/v) Formaldehyde solution (Sigma-Aldrich, 252549)
- 1M Glycine (w.v) (Sigma-Aldrich, 50048)
- Complete Sonication Buffer (see recipe below)
- Complete IP Buffer (see recipe below)
- Elution Buffer (see recipe below)
- RNase A (Qiagen, 19101)
- Proteinase K (Qiagen, 19133)
- 5 M NaCl (Sigma-Aldrich, S3014)
- MinElute® PCR Purification Kit (Qiagen, 28004)
- Qubit™ ds DNA BR kit (ThermoFisher, Q32853)
- Bioanalyzer DNA 1000 kit (Agilent Technologies, 5067-1504)
- Protein A Dynabeads (Life Technologies, 10004D)
- Protein G Dynabeads (Life Technologies, 100002D)
- Antibodies H3K4me1(Diagenode, C15410194)
- Antibodies H3K4me3 (Diagenode, C15410003)
- Antibodies H2K27ac (Diagenode, C15410196)
- Antibodies H3K27me3 (Diagenode, C15410195)
- Recovery Buffer (see recipe below)
- Complete Low Salt Buffer (see recipe below)
- Complete High Salt Buffer (see recipe below)
- 20% SDS (Fluka Analytical, 05030-1L-F)
- Low TE buffer (see recipe below)

- Qubit™ ds DNA HS Kit (ThermoFisher, Q32854)
- Microplex Library Preparation Kit v3 (Diagenode, C05010001)
- 60% Iodixanol OptiPrep (Sigma-Aldrich, D1556)
- 50%, 29%, 40% Iodixanol solution (see recipe below)
- Dounce Tissue Grinder Tube of 15 mL and Pestles (A & B) (VWR, 102095-542)
- 5 mL Eppendorf Tube snap cap (VWR, 89429-306)
- Counting chamber BLAUBRAND® Thoma pattern (Sigma-Aldrich, BR718020)
- Inverted Microscope (Zeiss, AXIO vert.A1)
- Centrifuge with swinging bucket for 5 mL tubes and cooling option (Eppendorf, 5810R)
- HulaMixer™ (Sigma-Aldrich, 15920D)
- Vortex Mixer (Gilson, 36110750)
- Micro-centrifuge (Beckman Coulter, 20R)
- 1.5 mL DNA LoBind Tube (VWR, 525-0130)
- Corning™ CoolRack™ (Fisher Scientific, C432034)
- EpiShear Probe Sonicator (Active Motif, 53052)
- Thermomixer (Eppendorf, 5382000015)
- Qubit 4 Fluorometer (Invitrogen, Q33226)
- Qubit™ assays tubes (ThermoFisher, Q32856)
- BioAnalyzer (Agilent Technologies, 2100)
- Magnetic rack (Life Technologies, 12321D)
- 2.0 mL DNA LoBind Tube (VWR, 525-0131)
- Mortar and pestle (Sigma-Aldrich, Z247464-1EA)

S4.3.2. Buffer recipes

<u>PBS Plus</u>

- PBS (Sigma-Aldrich, P5368), 1x
- PIC (Sigma-Aldrich, 11836170001), 1x
- 0.1 mM PMSF
- 5 mM Sodium Butyrate

Note: Prepare fresh under fume hood and keep on ice

<u>Sonication Buffer</u>

- 50 mM Tris-HCl pH 8.0
- 10 mM EDTA
- 1% (v/v) SDS

- Water

Note: Store at room temperature for up to 1 month. Complete the buffer for use with 7x PIC, 1 mM PMSF and 12.5 mM Sodium Butyrate (under a fume hood, while keeping on ice). If precipitation of SDS occurs, warm and agitate a little.

IP Buffer

- 20 mM Tris-HCl pH 8.0 (Sigma-Aldrich
- 2 mM EDTA pH 8.0 (Sigma-Aldrich, 03690)
- 0.1% (v/v) Triton X-100
- 150 mM NaCl
- Water

Note: Store at room temperature for up to 3 months. Complete the buffer for use with 1x PIC, 1 mM PMSF and 1 mM Sodium Butyrate (under a fume hood, while keeping on ice).

Elution Buffer

- 10 mM Tris-HCl pH 8.0 (Sigma-Aldrich
- Water

Note: Store at room temperature

Recovery Buffer

- 50 mM Tris-HCl pH 7.5 (Sigma-Aldrich
- 5 mM EDTA pH 8.0 (Sigma-Aldrich, 03690)
- 1% (v/v) SDS
- 50 mM NaCl
- Water

Note: Store at room temperature for up to 3 months. Complete the buffer for use with 0.1 mM PMSF (under a fume hood, while keeping on ice).

Low Salt Wash Solution

- 20 mM Tris-HCl pH 8.0 (Sigma-Aldrich
- 2 mM EDTA pH 8.0 (Sigma-Aldrich, 03690)
- 150 mM NaCl
- 1% (v/v) Triton X-100
- 0.1% (v/v) SDS
- Water

Note: Store at room temperature for up to 3 months. Complete the buffer for use with 1 mM Sodium Butyrate (under a fume hood, while keeping on ice).

High Salt Wash Solution

- 20 mM Tris-HCl pH 8.0 (Sigma-Aldrich
- 2 mM EDTA pH 8.0 (Sigma-Aldrich, 03690)
- 500 mM NaCl
- 1% (v/v) Triton X-100
- 0.1% (v/v) SDS
- Water

Note: Store at 4°C temperature for up to 3 months Complete the buffer for use with 1 mM Sodium Butyrate (under a fume hood, while keeping on ice).

<u>Low TE Buffer</u>

- 10 mM Tris-HCl pH 8.0 (Sigma-Aldrich
- 1 mM EDTA pH 8.0 (Sigma-Aldrich, 03690)
- Water

Note: Store at room temperature.

S4.3.3. Tissue disruption, crosslinking and sonication

Note: Pre-chill swinging bucket centrifuge and microcentrifuge to 4°C. Keep samples on ice

1. Place the frozen tissue (here assumes liver, but see tissue-specific modifications, section S4.3) into an ice-cold 15 mL douncer containing 5 mL cold PBS Plus.

Note: Allow the frozen tissue to thaw completely in the buffer.

2. Dounce the tissue with pestle A and B (approx. 5 strokes each) until obtaining a homogenized solution.

Note: It is important to carefully control the level of homogenization. Note that different people may apply different strength during the process.

3. Transfer the homogenate into a 5 mL Eppendorf tube.
4. Rinse the dounce and pestles with 0.5 mL of PBS Plus and add to the previous solution.

Note: A pipette tip cut at the bottom can be used to facilitate the aspiration.

5. Take an aliquot of 10 µL from the suspension and mix with 10 µL of dye (Hoescht or Trypan Blue). Count nuclei manually using a hemocytometer.
6. Spin in a pre-cooled centrifuge at 2,500g for 3 min at 4°C.
7. Remove and discard the supernatant.
8. Resuspend the pellet in 2.43 mL of PBS Plus buffer and add 67.5 µL of 37% formaldehyde solution at room temperature. Mix well.

Note: If more than 24 million nuclei, resuspend in 4.86 mL of PBS Plus buffer then add 135 µL of 37% formaldehyde solution and split into two tubes.

9. Rotate (360° rotation, 40 rpm) at room temperature for 5 min.

10. Quench the reaction with 360 µL of glycine 1M (0.125M final) and place back in the mixer for 10 min.
11. Centrifuge at 800g for 5 min at 4°C.
12. Remove the supernatant containing formaldehyde.
13. Resuspend with 4 mL of PBS Plus buffer by pipetting (applying brief vortex if necessary).

Note: If more than 24 million nuclei, resuspend each pellet in 2 mL of PBS Plus and then merge.

14. Centrifuge 800g for 5 min at 4°C.
15. Remove and eliminate the supernatant.
16. Resuspend with 5 mL of PBS Plus buffer by pipetting (applying brief vortex if necessary).
17. Centrifuge 800g for 5 min at 4°C.
18. Remove and eliminate the supernatant. Resuspend with 1 mL of PBS Plus buffer and transfer to a clean 1.5 mL Eppendorf tube.
19. Centrifuge on a pre-cooled micro-centrifuge 3000g for 10 min at 4°C.
20. Remove the supernatant.

Note: The nuclei pellet can be snap frozen and stored in -80ºC for up to 2 months.

21. Resuspend the pellet by pipetting in a ratio of 100 µL of complete sonication buffer per 1 million nuclei. Incubate on ice for 5 min.
22. Sonicate the chromatin while keeping the samples cold with the following settings:

Number of cycles	16
Amplitude	55 %
Cycle interval	1 second ‘ON’ / 1 second ‘OFF’
Cycle time	30 sec

Note: Each cycle = 30 sec total, composed by a repetition of 1 sec ‘ON’ / 1 sec ‘OFF’. The total ‘ON’ period is 15 sec. These parameters are specific to the sonication device EpiShear Probe Sonicator (Active Motif, 53052), used for Atlantic salmon.

23. Centrifuge the sonicated chromatin 8,000g for 10 min at 4°C.
24. Transfer the supernatant into a new 1.5 mL Eppendorf tube. Record the volume.

Note: If sonication done in multiple tubes, keep separate until quality control for each is performed.

25. Use an aliquot of 20 µL per tube for quality control.
26. Add 3x volumes of complete IP buffer to the remaining chromatin.

27. Store the diluted chromatin at -80°C (safe stop point).

S4.3.4. Quality control of sonication

28. Add 67 µL of elution buffer, 2 µL of RNase A, 5 µL of proteinase K and 6 µL of NaCl to the 20 µL aliquot.
29. Incubate in thermomixer at 500 rpm for 1h 30 min at 68℃.
30. Purify using MinElute purification kit and elute in 20 µL of elution buffer.
31. Record chromatin concentration using Qubit Broad Range kit using 1µL of eluate.
32. Validate the fragments size distribution with Bioanalyzer DNA 1000 kit or equivalent (e.g. Tapestation)

Note: A good quality sonication profile has a minimum of 70% of the chromatin in a size distribution of 100-600 bp centered around 350 bp. Later, you can pool together sample tubes with this expected fragment distribution to run the immunoprecipitation.

S4.3.5. Beads preparation

Note: The workflow below is intended for 4 immunoprecipitations with 5 µg of material each and 50 ng for input control, from sonicated chromatin sample with the following parameters: 400 µL volume before 3x dilution (step 25), Qubit-based concentration of aliquot (step 30) = 60 ng/µL.

33. Clean the beads by mixing 528µL of protein A beads, 528µL of protein G beads and 4576µL of complete IP buffer and split by 1276 µL in four LoBind 2 mL tubes.

Note: ratio 1:1 of protein A beads and protein B beads, and ratio 60 µL of beads for 260 µL of IP buffer. 10 % is added to avoid repetition of the beads cleaning in case of loss during washing and pipetting.

34. Mix by pipetting and/or vortexing and touch spin the tubes.
35. Place the tubes in the magnetic rack for a couple of min (or until the supernatant is clear of beads) and discard the supernatant.
36. Remove the tubes from the magnetic rack and add 1144 µL of complete IP buffer.
37. Repeat 2 more times the steps 34-36.

S4.3.6. Coupling of beads and antibodies

38. As per below table, mix the cleaned beads with each antibody in a separate 1.5 mL tube and rotate (360° rotation, 40 rpm for 30 min at room temperature).

130 µl of beads with	0.7µl (1.05µg) of H3K4me1
130 µl of beads with	0.8µl (1.12µg) of H3K4me3
130 µl of beads with	0.8µl (2.24µg) of H3K27ac
130 µl of beads with	1.9µl (2.09µg) of H3K27me3

S4.3.7. Chromatin pre-clearing

39. Place 520 µL of remaining cleaned beads in a 2 mL tube in the magnetic rack for 2 min and discard supernatant.

Note: If the sonicated chromatin sample is more diluted, adjust the size of the tubes used for pre-clearing from 2 mL capacity to 5 mL. As 5 mL tubes do not fit the magnetic rack, holding the tube and the rack close together by hand is recommended

40. Add 1364 µL of the diluted chromatin and rotate (360° rotation, 40 rpm) for 30 min at room temperature.

Note: 2% of the actual volume of diluted chromatin is added to avoid repetition of the pre-clearing in case of loss during washing and pipetting.

S4.3.8. Immunoprecipitation

41. Touch spin the tubes containing beads coupled with antibodies from step 38, then place them in the magnetic rack for 2 min and discard the supernatant.
42. Touch spin the tube of cleaned beads with chromatin from step 40, and place it in the magnetic rack for 2 min and transfer the supernatant to a new 2 mL tube.

Note: The new 5 mL tube contains pre-cleared chromatin.

43. Separate 3.34 µL (50 ng) of pre-cleared chromatin as 'input control', add recovery buffer to a total volume of 82 µL and store at 4°C.
44. Place 333.34 µL of pre-cleared chromatin into each of the tubes with beads coupled with antibodies from step 38, then add 166.67 µL of complete IP buffer to each.
45. Rotate (360° rotation, 40 rpm) overnight (12-16 hr) at 4°C.

S4.3.9. Bead washing, decrosslinking and DNA recovery

46. Touch spin the tubes, place them on the magnetic rack for 2 min and discard supernatant.
47. Resuspend the beads in 200 µL of complete IP buffer.
48. Touch spin the tubes, place them on the magnetic rack for 5 min and discard supernatant.
49. Resuspend the beads in 200 µL of complete low salt wash buffer.
50. Touch spin the tubes, place them on the magnetic rack for 5 min and discard supernatant.
51. Repeat the washing with low salt wash buffer.
52. Resuspend the beads in 200 µL of complete high salt wash buffer.
53. Touch spin the tubes, place them on the magnetic rack for 5 min and discard supernatant.
54. Repeat the washing with high salt wash buffer.
55. Resuspend the beads in 57 µL of recovery buffer.
56. Elute the DNA from the beads in a Thermomixer at 1,000 rpm for 1h 30 min at 65ºC.
57. Touch spin the tubes and place them on the magnetic rack for 5 min.
58. Transfer the eluted chromatin to new clean LoBind 1.5 mL Eppendorf tubes.
59. Wash the beads with 30 µL of recovery buffer. Mix and touch spin the tubes.

60. Place the tubes on the magnetic rack. Transfer into the full volume of eluted chromatin from step 55 (i.e. 87 µL final).
61. Take out the 'input control' tube stored in the fridge.
62. Add 5 µL 20% SDS, 2 µL of RNase A, 5µL of proteinase K and 6 µL of NaCl to the 'input control' tube.
63. Add 2 µL of RNase A, 5 µL of proteinase K and 6 µL of NaCl to the tubes containing immunoprecipitated chromatin.
64. Incubate in a Thermomixer at 500 rpm for 1h 30 min at 68°C.
65. Purify the samples using MinElute PCR purification kit and elute in 32 µL of low TE buffer.

Note: DNA from the input and immunoprecipitated chromatin can be stored at - 20°C until library preparation.

66. Quantify samples with Qubit High Sensitivity kit as follows:

Input	H3K4me1	H3K4me3	H3K27ac	H3K27me3
1 µl of 1/10 dilution	6µl	6µl	2µl	2µl

Note: Different volumes of immunoprecipitated DNA are recommended for Qubit quantifications to ensure that the detected concentration is not below Qubit sensitivity limits.

67. Prepare the library using the Microplex Library Preparation Kit v3 following the manufacturer's instructions.

Note: Recommended minimum input DNA 0.2 ng. It should be started from a volume of 10 µL.

DNA input (ng)	Number of cycles
50	5
20	6
10	7
5	8
3	9
2	10
1	11
0.5	12
0.3-0.2	13

S4.4. Modifications to BodyMap reference protocol

S4.4.1. Atlantic salmon brain

Follow the reference protocol, but use 50-75mg of initial tissue. Crosslink for 5 min. Centrifugate at 2,500g (step 11) and 1,500g (steps 14 and 17). Sonicate for 15 cycles (step 22).

S4.4.2. Immature gonads

Follow the reference protocol, but use 30-100mg of initial tissue. Sonicate for 18 cycles (step 22).

S4.4.3. Mature female gonads

Follow the reference protocol, but use 200mg of initial tissue. Sonicate for 18 cycles (step 22).

S4.4.4. Mature male gonads

More modifications were needed to perform ChIP from mature male gonads material due to sperm content. This protocol may need to be performed 2-4 times to ensure sufficient amount of generated chromatin from the gonad excluding contamination from sperm. This is important for successful immunoprecipitation. Please follow these instructions, in a similar way as for the ATAC-seq procedure;

S4.3.4.1. Buffer recipes

Iodixanol, Gradient 1, Bottom layer solution

- 40% (v/v) Iodixanol OptiPrep (Sigma-Aldrich, D1556)
- PBS Plus, 1x

Note: Prepare fresh and keep on ice

Iodixanol, Gradient 1, Middle layer solution

- 29% (v/v) Iodixanol OptiPrep (Sigma-Aldrich, D1556)
- PBS Plus, 1x
- 0.1% (v/v) Nonidet P40 Substitute (Roche, 11332473001)
- 0.15 mM Spermine (Sigma-Aldrich, S3256)
- 0.5 mM Spermidine (Sigma-Aldrich, S2626)

Note: *Prepare fresh and keep on ice*

Iodixanol, Gradient 2 solution

- 50% (v/v) Iodixanol OptiPrep (Sigma-Aldrich, D1556)
- PBS Plus, 1x

Note: Prepare fresh and keep on ice

S4.3.4.2. Modifications for mature male gonad

68. Transfer the tissue (400-600 mg) in a 5 mL tube containing 1 mL of PBS Plus buffer. Gently squeeze the tissue with a pestle against the tube to release the sperm, but do not disrupt the tissue into smaller pieces.
69. Vortex for at least 30 sec and filter through a 40 µm cell strainer suspended in a 50 mL Falcon tube. Add 500 µL of PBS Plus buffer over the cell strainer to rinse the tissue and place the tissue back into the 5 mL tube. Discard the filtrate containing mostly sperm.
70. Repeat step 1 and 2 to release sperm from the tissue as much as possible.
71. Transfer the tissue into an ice-cold dounce containing 5 mL cold PBS Plus and homogenize with pestle A and B.
72. Transfer the homogenate into a 5 mL Eppendorf tube. Avoid transferring pieces that did not effectively homogenize.
73. Centrifuge on a pre-cooled micro-centrifuge at 3,000g for 10 min at 4°C.
74. Discard the supernatant and add 800 µL of fresh PBS Plus buffer.
75. Add 560 µL 60% iodixanol and mix to obtain 1,400µL to achieve a solution containing 25% iodixanol.
76. Prepare a 5 mL tube for the first gradient centrifugation: place 1200 µL of bottom layer buffer (40% iodixanol) and gently add 1200 µL of middle layer buffer (29% iodixanol). Avoid mixing the layers.
77. Add solution from step 8 to the 5 mL tube over the middle layer solution.
78. Centrifuge in a pre-cooled swinging bucket centrifuge with brakes set to 'off' at 3,200g for 30 min at 4°C. This will result in a visible band between the 29% and 40% iodixanol layers.
79. Recover the nuclei band (200-400 µL) into a clean 1.5 mL Eppendorf tube. This can be done by careful removal of the first two layers (25% and 29% iodixanol) or by targeting the nuclei band.
80. Add and mix 0.7x volume of PBS Plus buffer to obtain a 15-20% iodixanol solution with nuclei.
81. Let the solution sediment for 5-10 min on ice.
82. Carefully transfer supernatant into another clean 1.5 mL Eppendorf tube. Avoid transferring pieces of tissue or big aggregates.
83. Prepare two 2 mL tubes for the second gradient centrifugation: place 300 µL of bottom layer buffer (50% iodixanol) and gently add to it half of the recovered nuclei solution from the previous step.
84. Centrifuge in a pre-cooled micro-centrifuge with brakes set to 'off' at 12,000g for 40 min at 4°C. A band should be visible between the 15-20% and 50% iodixanol layers.
85. Remove and discard the first gradient layer containing 15-20% iodixanol solution in both tubes.
86. Recover the nuclei layers and transfer them into a common 1.5 mL Eppendorf tube (100-200µL).
87. Dilute the nuclei layer 10 times with PBS plus to obtain a clean solution with nuclei.

88. Centrifuge at 5,000g for 10 min at 4°C.
89. Discard the supernatant and resuspend the nuclei pellet with 2.5 mL of PBS Plus buffer.
90. Take an aliquot of 10 µL from the suspension and mix with 10 µLof dye (Hoescht or Trypan Blue). Count the nuclei and sperm using hemocytometer. Gonad cell nuclei to sperm nuclei ratio between 1 to 2 and 1 to 5 is expected.
91. Add 67.5 µL of 37% formaldehyde solution at room temperature and mix by pipetting.

Note: When there are more than 24 million nuclei, add 2.43 mL more of PBS Plus buffer, then add 135 µL of 37% formaldehyde solution and split into two tubes.

92. Continue the protocol as stated in reference protocol (steps 9-20).
93. Resuspend the pellet and sonicate using the settings of the reference protocol as follows:

Gonad cell nuclei to sperm nuclei ratio	Nuclei per 300µl sonication buffer	Sonication cycles
1 to 5	1.5 million	18
1 to 4	2 million	18
1 to 3	2.5 million	20
1 to 2	3 million	20

94. Continue protocol as stated in reference protocol (step 23 onwards).

S4.4.5. Muscle

S4.3.5.1 Buffer recipes

<u>Hypotonic Buffer</u>

- 10 mM HEPES pH 7.4
- 10 mM KCl
- 5 mM $MgCl_2$
- 0.1% (v/v) Nonidet P40 Substitute (Roche, 11332473001)
- Water

Note: Store at 4°C temperature for up to 1 month. Complete the buffer for use with 1x PIC, 0.1 mM PMSF and 1.25 mM Sodium Butyrate (done under fume hood and keeping on ice)

S4.3.5.2. Modifications for muscle

95. Grind 1g of tissue using a mortar chilled on dry ice until it is powdery
96. Transfer the grinded material into a 5 mL tube containing 5 mL of cold complete hypotonic buffer.
97. Rotate (360° rotation, 40 rpm) for 10 min at 4°C.

98. Transfer to a douncer and homogenate using pestle A (approx. 5 strokes).

99. Transfer homogenate back to a 5 mL tube and add hypotonic buffer to make 5 mL volume if needed.

100. Take an aliquot of 10 µL from the suspension and mix with 10 µL of dye (Hoescht or Trypan Blue). Count nuclei using hemocytometer.

101. Add 135 µL of 37% formaldehyde solution at room temperature.

102. Rotate (360° rotation, 40 rpm) for 10min at 4°C

103. Quench the reaction with 720 µL of glycine 1M (0.125M final) and place it back in rotation for 10 min.

104. Transfer crosslinked tissue back to douncer and homogenize with pestle A (5 strokes).

105. Transfer the homogenate to a 5 mL tube.

106. Centrifuge at 2,500g for 10 min at 4°C.

107. Remove and eliminate the supernatant.

108. Resuspend with 5 mL of complete hypotonic buffer.

109. Transfer solution back to the douncer and homogenize with pestle A (5 strokes).

110. Filter using a 40 µm strainer placed over a 50 mL Falcon tube.

111. Transfer the filtrate into a new 5 mL tube.

112. Centrifuge at 1,000g for 10 min at 4°C.

113. Remove the supernatant.

114. Resuspend pellet in 4 mL of hypotonic buffer. Split the solution in order to obtain 3 million nuclei per 1.5mL Eppendorf tube.

115. Centrifuge at 3,000g for 10 min at 4°C.

116. Remove the supernatant.

Note: The nuclei pellet can be snap frozen and stored in -80ºC for up to 2 months.

117. Resuspend the pellet in a ratio of 300 µL of complete sonication buffer per 3 million of nuclei. Incubate on ice for 5min.

118. Sonicate the chromatin for 18 cycles, keeping the samples ice cold and using settings of the reference protocol (step 22).

119. Continue the protocol as stated in reference protocol from step 23 onwards.

Supplementary Text 5. Transcription factor binding site (TFBS) enrichment results

Transcription factors (TF) are nuclear proteins that regulate transcription to modulate cell identity or tissue homeostasis by binding specific DNA sequence motifs, i.e., TF binding sites (TFBS). As explained in the main text, we used the robust open chromatin regions annotated as active promoters or enhancers by ChromHMM in TFBS enrichment analyses to seek evidence

for enrichment of vertebrate TFs according to the known biology of embryogenesis (DevMaps) or adult tissue differentiation (BodyMaps). This was done separately for both salmonid species (Supplementary Figs. 10-11), with the results compared to establish evidence in support of shared TFBS usage across species (Supplementary Fig. 12). Though not exhaustive, examples of enriched TFBS are provided below, consistent with expected functions of vertebrate TFs, providing evidence for the high quality of our ChromHMM predictions.

DevMap enriched TFBS

The Sox2::Pou5f1 complex is a critical regulator of pluripotency in vertebrates including zebrafish [6,7]. In our study, the Sox2::Pou5f1 TFBS was most enriched at the late blastula and mid-gastrulation stages in active promoters and enhancers of both species (Supplementary Fig. 10), with activity sharply declining from somitogenesis onwards. In zebrafish, Sox2 family and Pouf51 genes, alongside Nanog, are essential to initiate zygotic genome activation (ZGA) and the termination of the maternal programme that dominates before the maternal-to-zygotic transition (MZT) [7]. The enrichment of this motif at these stages of salmonid embryogenesis suggests a conserved role in the MZT. Another TFBS showing the strongest enrichment in the late blastula, followed by mid-gastrulation, was MGA::EVX1, enriched in active promoters and enhancers in both salmonid species (Supplementary Fig. 10). MGA is a bHLH TF that in zebrafish is highly expressed as a maternal transcript, before showing zygotic expression during gastrulation, and ongoing expression in developing brain, heart and gut during organogenesis, which is essential to the development of these organs [8]. The enrichment of this TFBS in active regulatory elements at the earliest profiled stages of salmonid development is consistent with this TF being a maternal factor, with a role in early organogenesis.

In salmon, we identified enrichment of the TFBS for Sox10 at mid-gastrulation and early somitogenesis in both active enhancers and promoters (Supplementary Fig. 10). The same TFBS was most enriched at early somitogenesis in active enhancers of trout (Supplementary Fig. 10). Sox10 plays a key role in the formation and differentiation of neural crest cell lineages including neural cells and melanocytes [9,10]. The enrichment of Sox10 TFBS in our data is consistent with neural crest development being captured during salmonid embryogenesis. The TFBS for IRF6 similarly showed enrichment at mid-gastrulation of active promoters for both species. IRF6 is a maternal TF that causes severe gastrulation defects when ablated in *Xenopus* or zebrafish [11]. TP63 binding sites were enriched in active regulatory elements of salmon at early somitogenesis, which in zebrafish is a master regulator of ectoderm development, with ectoderm tissues specified during early somitogenesis [12]. We also identified a strong enrichment of the TFBS for Hoxd10 specifically for active enhancers of both species at early somitogenesis, consistent with the established importance of Hox10 genes in the presomitic mesoderm [13] a structure that appears during the earliest stage of segmentation, leading into somitogenesis.

At later stages of embryogenesis, we identified enriched TFBSs consistent with the functions of conserved TFs. For example, the TFBS for MEF2B was enriched in both mid- to late-somitogenesis stage for both active enhancers and promoters of both species (Supplementary Fig. 10). MEF2 family members are key regulators of cardiac and skeletal myogenic differentiation in mammals and teleosts [14,15], which are both highly active processes during the segmentation period. NRF1 (Nuclear respiratory factor 1) was an interesting TFBS enriched specifically in active promoters of both species at late somitogenesis [16], recognized as a master regulator of mitochondrial biogenesis, which may reflect the growing energy demands of late stage embryos, e.g. for coordinated muscle movement and brain activity.

BodyMap enriched TFBS

Hepatocyte nuclear factors (HNFs) are a group of transcription factors with master functions in transcriptional regulation of liver-specific genes, promoting hepatocyte development [17]. In our analysis, TFBS for two distinct HNFs, HNF1 and HNF4, were enriched in liver for both active enhancers and promoters of both species (across sexes and maturation stages) (Supplementary Fig. 11). Both HNF1 and HNF4 play critical roles in maintaining hepatocyte function [18,19]. HNF4 is a conserved nuclear receptor superfamily member indispensable for liver development in mammals [19]. TFBS for IRF6 and TP63 were also enriched within active enhancer and promoters from liver samples of both species (Supplementary Fig. 11). In mammals, IRF6 protects hepatocytes against liver steatosis by binding regulatory elements in peroxisome proliferator-activated receptor γ [20]. TP63 is a transcription factor that binds the promoter of *SIRT1* in mammals to regulate hepatic glucose production and insulin sensitivity [21].

In brain-active regulatory elements of both species, the most enriched TFBS, particularly in active enhancers, was Neurod1/2, representing master TFs for central nervous system and brain development [22]. Specific to promoters of both species, the binding site for NRF1 was highly enriched in brain samples (Supplementary Fig. 11). In mammals, NRF1 is expressed broadly across tissues and plays a key role in regulating mitochondrial biogenesis, respiration and cell redox homeostasis by binding the promoter of target genes [23,24]. Hence, the enrichment of NRF1 binding sites in salmonid brains is logical, given the extensive metabolic demands of this tissue. Consistent with this, NRF1 binding sites were also highly enriched in regulatory elements activated during late stages of embryogenesis in both species (Supplementary Fig. 11), where the brain is developing, and energy demands of the embryo are greatly increasing (e.g., according to our GO transcriptome analysis; Supplementary Text 6). RFX2 was interesting an TFBS enriched in active brain enhancers and promoters in both salmonid species; this TF is considered essential for vertebrate ciliogenesis and is highly expressed in brain, where it regulates neuronal cilia differentiation [25]. The enrichment of RFX2 binding sites in salmonid brain regulatory elements likely represents the proportionate greater importance of cilia differentiation in abundant brains neurons, compared to other tested tissues in the analysis (i.e., muscle, liver). Similar to NRF1, binding sites for RFX2 were also enriched in active enhancers of both salmonids at the latest stage of embryogenesis (Supplementary Fig. 11), where brain and neural differentiation is highly active.

In muscle samples, we observed enrichment of binding sites for MEF2B in active regulatory elements, particularly in salmon. As noted above, MEF2B binding sites were also enriched in active regulatory elements of both salmonid species at late stages of embryogenesis (Supplementary Fig. 11), when muscle development is highly active. Six2 binding sites were enriched in active muscle regulatory elements of both species (Supplementary Fig. 11). This TFBS was predicted to be an important regulator of skeletal muscle development in cattle, including for both myoblasts and differentiated muscle fibres [26].

Overview of enriched TFBS shared between salmonid species

Overall, we identified 672 enriched TFBS across the four enrichment analyses performed, of which 58% were common between the two species (Supplementary Fig. 12). We found more enriched TFBS shared across the two species in DevMap samples, 71% for active promoters and 56% for active promoters, which compared to 51% and 48% for the equivalent BodyMap regulatory elements, respectively. It was observed that trout had a larger proportion of unique rather than shared enriched TFBS compared to salmon specifically for BodyMap samples

(Supplementary Fig. 12). Specifically, for trout BodyMap samples, 45% and 42% of active promoters and enhancers were not shared with salmon, whereas the reciprocal values for salmon were 12% and 27%, respectively (Supplementary Fig. 12). Conversely, for DevMap samples, the number of species-specific enriched TFBS in both active enhancers and promoters was comparable in both species. These results may reflect the fact that the DevMap ATAC-seq and ChIP-seq experiments were performed at the same site, whereas for BodyMap, different collaborators performed these analyses independently, albeit with standardized consortium protocols.

Supplementary Text 6. Transcriptome dynamics during embryogenesis

DevMap transcriptome dataset overview

For both salmon and trout, our DevMap transcriptome datasets comprise 14 matched stages of embryogenesis, determined by relative age [3] and anatomical features observed during sampling. Below we report processes captured across each major sampled stage based on the genes expressed in SOM clusters (Fig. 2a; Supplementary Figs. 15-16), including GO enrichment analysis (Supplementary Figs. 19-20; Supplementary Tables 8-9).

Maternal to zygotic transition clusters

The earliest embryonic stage sampled was late cleavage, closely followed by early, mid and late blastulation stages. In salmon (SOM clusters: 'Maternal 1', 'Maternal 2', 'Maternal 3' and 'Maternal 4') and trout (SOM clusters: 'Maternal 1', 'Maternal 2', 'Maternal 3'), SOM captured genes showing highest expression before or during blastulation, with a subsequent decrease in expression. This pattern is consistent with maternal transcripts, or a combination of maternal transcripts and genes expressed during early zygotic genome activation (ZGA). Salmon cluster 'Maternal 2' and trout cluster 'Maternal 1' were enriched for GO terms linked to cell division, i.e. '*cell cycle*', '*chromosome segregation*', '*centrosome cycle*', '*cytokinesis*' and '*DNA replication*', consistent with cell cleavages (Supplementary Figs. 19-20). These terms are also associated with housekeeping roles, which may explain why salmon 'Maternal 2' cluster genes maintain some expression throughout embryogenesis (Fig. 2a; Supplementary Fig. 15; Supplementary Fig. 19). Salmon cluster 'Maternal 1' strongly resembled a maternal transcript degradation pattern, and was enriched for GO term GO:0048511 '*rhythmic process*', explained by genes including *nocta*, which contributes to 3'-5' exonuclease activity and RNA deadenylation [27] and *dbpa*, encoding an RNA helicase involved in RNA structural rearrangements [28], suggesting that maternal or early zygotic transcripts are performing roles related to RNA modification and translational regulation. Other terms present in salmon cluster 'Maternal 1' , and trout clusters 'Maternal 1' and 'Maternal 2' included GO:0045892 '*negative regulation of transcription, DNA-templated*' and GO:0046578 '*regulation of Ras protein signal transduction*' (Supplementary Figs. 19-20); these terms are involved in cellular differentiation, suggesting the pre-blastulation stage represents a transition between cleavage and early blastulation, with maternal transcripts still present and the zygotic genome not yet fully activated. Other terms found in maternal SOM clusters, including GO:0051090 '*regulation of DNA-binding transcription factor activity*', GO:0006406 '*mRNA export from nucleus*' (trout 'Maternal 2') are however consistent with some degree of ZGA in late cleavage stages (Supplementary Figs. 19-20).

Salmon SOM clusters showing up-expression during blastulation include ‘Maternal 4’, capturing genes also highly expressed in pre-blastulation, and ‘Blastula 1’, a highly specific mid-blastulation cluster (Fig. 2a; Supplementary Fig. 15). Salmon SOM cluster ‘Blastula 2’ spans the transition from late blastulation to early gastrulation. The most closely related trout SOM clusters showed less overlap between pre-blastulation and blastulation, with no obvious equivalent to salmon ‘Maternal 4’ (Fig. 2a; Supplementary Fig. 16). Trout SOM cluster ‘Blastula 1’ with mid-blastulation specific expression, showed no enriched GO terms, despite being comprised of 1,261 genes, potentially highlighting issues in the annotation of early development genes. Enriched GO terms for salmon ‘Blastula 1’ included GO:0007399 ‘*nervous system* development’. However, the genes explaining this term were associated with endoderm development, such as *her5* [29] as well as genes associated with microtubule stabilization, such as *campsap2a* [30] (Supplementary Fig. 19). Other terms enriched in both blastulation-specific clusters were GO:0043408 ‘regulation of *MAPK* cascade’, GO:0007049 ‘cell cycle’, GO:0030178 ‘negative regulation of *Wnt* signalling pathway’ and several terms associated with RNA modification and metabolism (Supplementary Fig. 19-20).

Gastrulation clusters

Salmon had two SOM clusters capturing genes showing up-regulation during gastrulation (Fig. 2a; Supplementary Fig. 15). As noted above, salmon cluster ‘Blastula 2’ spans the transition from blastulation to gastrulation, whereas ‘Gastrula 1’ showed gastrulation-specific expression, with highest expression in the late gastrula stage. In trout (Fig. 2a; Supplementary Fig. 16), cluster ‘Blastula’ spanned the transition from blastulation to gastrulation, while cluster ‘Gastrula 1’ had gastrulation-specific expression, with the highest expression found towards late gastrulation. Salmon cluster ‘Gastrula 1’ was enriched for two main processes: GO:0072330 ‘monocarboxylic acid biosynthesis’ (explained by eight genes, including *acaca*, *elovl6* and *ptgs2v*), and GO:0042981 ‘regulation of apoptosis’ (explained by eighteen genes, including *dedd*, *mdm2*, *socs3* and *diabloa*) (Supplementary Fig. 19). Several remaining terms below the employed significance threshold were associated with gastrulation, including GO:0007369 ‘Gastrulation’, GO:0060027 ‘Convergent extension during gastrulation’, GO:0098609 ‘cell-cell adhesion’, as well as terms associated with morphogenesis, cell differentiation and Wnt signalling. Gastrulation clusters in both species were highly enriched in terms associated with Wnt signalling (Supplementary Figs. 19-20), explained by genes including *wnt8a*, *wnt3a*, *wnt10b* among others. Other enriched cell-cell signalling pathways were Jak/Stat (salmon), as well as Hippo, Ras and Notch (both species). Additionally, both SOM clusters were enriched for terms associated with nervous system development, explained by genes of the Brinp, Gata and Fox families. Both SOM clusters were also enriched in terms associated with cell-cell adhesion, explained by genes of the cadherin superfamily among others (Supplementary Figs. 19-20).

Somitogenesis clusters

For salmon, SOM clusters ‘Somitogenesis 1’ and ‘Somitogenesis 2’ correspond to genes showing up-regulation during early-to-mid segmentation and mid-to-late segmentation, respectively (Fig. 2a; Supplementary Fig. 15). Cluster ‘Pharyngula 1’ included genes that were relatively up-regulated during late segmentation, but showed highest expression in pharyngula stages. Cluster ‘Somitogenesis 1’ was enriched for GO:0016055 ‘Wnt signalling pathway’ (twenty-three genes), as well as GO:0048513 ‘animal organ development’ (thirty-seven genes) (Supplementary Fig. 19). Enriched GO terms for this cluster are broadly linked with tissue development, organogenesis, cell signalling and, importantly, GO:0007389 ‘pattern specification

process'. Cluster 'Somitogenesis 2' showed similar GO enrichment, including 'Wnt signalling pathway', but also terms associated with muscle development (Supplementary Fig. 19). Additionally, GO:0001568 'blood vessel development' was enriched in salmon 'Somitogenesis 1'. For trout, SOM clusters 'Somitogenesis 1' and 'Somitogenesis 2' captured genes showing highest expression during early-to-mid segmentation and mid-to-late segmentation respectively (Fig. 2a; Supplementary Fig. 16), mirroring results in salmon. While the enriched GO terms in 'Somitogenesis 1' clusters were almost identical, trout cluster 'Somitogenesis 2' also showed enrichment for GO:0033014 'tetrapyrrole biosynthetic process', and terms associated with the circulatory system, including GO:0015671 'gas transport' and GO:0008015 'blood circulation' (Supplementary Fig. 20). Despite this difference, other terms associated with morphogenesis and tissue development were shared between salmon and trout (Supplementary Figs. 19-20).

Pharyngula clusters

Genes comprising the salmon SOM clusters 'Pharyngula 1' and 'Pharyngula 2' showed highest expression during the pharyngula period, with 'Pharyngula 1' having the highest normalized expression. A third cluster ('Early/late') had high expression in both late cleavage and pharyngula (Fig. 2a; Supplementary Fig. 15). A similar pattern was observed for trout (Fig. 2a; Supplementary Fig. 16), with cluster two pharyngula specific clusters and one Early/late cluster. Enriched GO terms for the pharyngula clusters reflect upregulation of processes associated with metabolism, such as ATP production, glucose, carbohydrate and fatty acid metabolism, consistent with increased energy production demands of the developing fish (Figs S22-23). Pharyngula-specific clusters were particularly enriched in metabolic processes in both species (Supplementary Figs. 19-20). Terms associated with brain function (e.g., synaptic signalling or regulation of neurotransmitter levels) and nervous system development were also prevalent in all pharyngula clusters. Moreover, all clusters were highly enriched for GO terms associated with development, especially organ and tissue development, including urogenital system development, renal development, eye development and epithelium development, among others (Supplementary Figs. 19-20).

Constitutive embryonic gene expression

The largest SOM clusters in the salmon and trout embryonic datasets were represented by genes with low normalized expression variability across developmental stages, capturing ~40% and 50% of all genes in salmon and trout, respectively (Fig. 2a; Figs S15-16). Enriched GO terms for these clusters were composed almost exclusively of housekeeping functions linked to DNA repair, the cell cycle, nucleic acid metabolism, ribosome production and maintenance, among others (Supplementary Figs. 19-20). Although these constitutive SOM clusters did not display stage-specific expression, there were small variations in terms of expression pattern. For example, cluster 'Constitutive 1' in salmon and cluster 'Constitutive 3' in trout both display an increase in expression towards the pharyngula period. Interestingly, the most highly enriched terms in these clusters were associated with cellular respiration (salmon) and ATP metabolism (trout) (Supplementary Figs. 19-20), mirroring the prevalence of metabolic genes in pharyngula clusters, consistent with the increased energy requirements in late embryonic stages, likely due to the demands of brain and muscle tissue, which together comprise a large fraction of the embryo mass.

Supplementary Text 7. Transcriptome dynamics across tissues at different maturation stages

BodyMap transcriptome dataset overview

BodyMap datasets are composed of eight tissues in adult male and female salmon and trout at two stages of ontogeny (sexually immature; sexually mature), with samples matched across species. SOM analysis captured a highly heterogeneous landscape of tissue expression, with most clusters displaying tissue-specific expression (23/36 in salmon and 21/36 in trout) (Extended Data Fig. 4; Supplementary Figs. 22-23). Moreover, 63% (29,787 out of 47,109) and 60% (23,037 out of 38,230) of Ensembl annotated coding genes were captured by tissue-specific SOM clusters in salmon and trout, respectively. An additional type of SOM cluster (hereafter 'multi-expression') was observed capturing genes showing up-regulation in more than one tissue. Below we explore the different SOM clusters based on tissue expression, along with enriched GO terms (Supplementary Tables S8-9).

Ovary clusters

In salmon, three out of thirty-six SOM clusters showed highly ovary-specific expression (Extended Data Fig. 4; Supplementary Fig. 22). Combined, these clusters were composed of 5,024 genes, representing 10.6% of the tissue transcriptome. Trout similarly had three SOM clusters in this category (Extended Data Fig. 4; Supplementary Fig. 23), comprising 3,022 genes, or 7.9% of the tissue transcriptome. The most strongly enriched GO terms for both species were associated with egg production, including GO:0048477 'oogenesis' (Supplementary Figs. 26-27), explained by genes including *bmp15*, which acts upstream of female sex differentiation and regulates oocyte maturation [31] and *buc*, which plays a role in the specification of the oocyte animal-vegetal axis, and is present within the blastomeres [32], suggesting maternally deposited genes are captured in these clusters. Other GO terms enriched in ovary clusters included GO:0051321 'meiotic cell cycle', explained by genes such as *rnf212*, a dosage sensitive ring finger protein with a role in crossing over during meiosis [33]. Additional enriched GO terms included those involved in fatty acid metabolism, sperm-egg recognition and cytoskeleton organisation. While similar GO terms were enriched in ovary SOM clusters in both species (Supplementary Figs. 26-27), trout showed more terms associated with non-coding RNA processing and RNA methylation, as well as embryonic development, including GO:0007369 'gastrulation' (Supplementary Fig. 27), which included genes such as *bbs12*, *mkks* and *desi2*, which are each involved in convergent extension during gastrulation, as well as *msgn1*, involved in mesoderm differentiation in the transition from gastrulation to somitogenesis [34].

Brain clusters

In salmon and trout four and three respective SOM clusters captured brain-specific genes, which together represent 14% and 15% of all expressed genes in the tissue transcriptome (Extended Data Fig. 4; Supplementary Figs. 22-23), respectively. Most enriched GO terms in these clusters (Supplementary Figs. 26-27) were associated with brain and nervous system functions in both species, including GO:0007399 'nervous system development', GO:0099536 'synaptic signalling' and GO:0006813 'potassium ion transport'. Additionally, GO terms such as GO:0008015 'blood circulation' and GO:0006939 'smooth muscle contraction', among others, highlight the role of vascularisation and oxygenation for brain function.

Testes clusters

In salmon, four SOM clusters composed of 3,910 genes (6.5% of the tissue transcriptome)

capture testes-specific gene expression (Extended Data Fig. 4; Supplementary Figs. 22-23). These clusters could be demarcated into immature and mature testes specific gene expression, the strongest observed effect of maturity across all tissues sampled. The trout tissue panel dataset was missing mature testes samples due to sampling, so the same effect cannot be compared across species. The missing mature testis samples also explain the lower number (two) of testes-specific SOM clusters in trout, which captured 1,593 genes (4% of the tissue transcriptome). Enriched GO terms for trout SOM clusters showing testes-specific expression were associated with meiosis and cell division (Supplementary Fig. 27), highlighting the high rate of cell division in testes. This included GO terms such as GO:0051321 'meiotic cell cycle', with most other enriched terms associated with DNA repair and rRNA production. Other enriched terms were associated with sperm production and function, such as GO:0007283 'spermatogenesis' and GO:0001539 'cilium or flagellum-dependent cell motility'. Finally, terms such as GO:0034470 'ncRNA processing' suggest a role of ncRNA in sperm production. Enriched GO terms in salmon testes-specific SOM clusters were very similar to trout (Supplementary Figs. 26-27), with notable differences linked to terms associated with development, including embryo, head, brain, neural tube, kidney and eye development, as well as pathways commonly associated with early development, such as Notch and Wnt.

Muscle clusters

In salmon, three SOM clusters captured muscle-specific genes (Extended Data Fig. 4; Supplementary Figs. 22-23), composed of 3,183 genes (7% of the tissue transcriptome). This includes a small (292 genes) cluster expressed almost exclusively in immature females. Enriched GO terms for these clusters (Supplementary Fig. 26) are exclusively associated with muscle structural proteins, including GO:0061061 'muscle contraction', GO:0051146 'striated muscle cell differentiation', GO:0048741 'skeletal muscle fibre development' and GO:0006936 'muscle contraction'. The other enriched GO terms were largely associated with metabolism and energy production/storage, including GO:0006757 'ATP generation', GO:0005977 'glycogen metabolic process', GO:0006603 'phosphocreatine metabolic process', GO:0005975 'carbohydrate metabolic process' and GO:0006112 'energy reserve metabolic process'. Enriched GO terms in a female muscle specific cluster for salmon were mainly linked to metabolism, including triglyceride, carbohydrate, fructose and proline and folic-acid metabolism, but also the cellular response to insulin (Supplementary Fig. 26).

The four trout muscle-specific SOM clusters (Extended Data Fig. 4; Supplementary Fig. 23) captured 3,486 genes (9% of the tissue transcriptome) were strongly conserved with salmon, including a small immature female-specific cluster. Enriched GO terms extensively overlapped for muscle-specific SOM clusters across species (Supplementary Figs. 26-27), with the exception that trout clusters were specifically enriched for embryonic development associated terms. The trout immature female-specific muscle SOM cluster comprised 494 genes and enriched for GO terms associated with metabolism. Other enriched trout GO terms not present in salmon included T-cell activation, endocytosis and adaptive immune response.

Liver clusters

In salmon, three liver-specific SOM clusters captured 4,057 genes (8.5% of the tissue transcriptome) (Extended Data Fig. 4; Supplementary Fig. 22). One of these clusters captured 314 genes specifically up-regulated in mature males. Trout had four liver-specific SOM clusters (Extended Data Fig. 4; Supplementary Fig. 23), which displayed higher heterogeneity, including clusters showing up-regulation in mature males, mature females, immature fish of both sexes,

and one showing more homogeneous up-regulation. Enriched GO terms in the liver clusters for both species included diverse terms associated with metabolism, highlighting the important metabolic role played by the liver (Supplementary Figs. 26-27; Supplementary Tables 8-9). Enriched GO terms associated with hemostasis in both species highlight the liver's role in coagulation (Supplementary Figs. 26-27). Liver immune functions are represented by enriched GO terms in both species (Supplementary Figs. 26-27), including GO:0006956 'complement activation' and GO:0002449 'lymphocyte mediated immunity'

Head kidney clusters

In salmon, two SOM clusters were head kidney specific (Extended Data Fig. 4; Supplementary Fig. 22), capturing 2,261 genes (4.7% of the tissue transcriptome). Trout also had two head kidney specific SOM clusters comprised of 1,783 genes (4.6% of the tissue transcriptome) (Extended Data Fig. 4; Supplementary Fig. 23). In both species, enriched GO terms (Supplementary Figs. 26-27) were consistent with the head kidney's primary role in immunity, for example, GO:0050776 'regulation of immune response', GO:0006935 'chemotaxis', GO:0019221 'cytokine mediated signalling pathway', GO:0050863 'regulation of T-cell activation', GO:0002250 'adaptive immune response' and GO:0002250 'phagocytosis', reflecting both primary and secondary immune functions. The primary hematopoietic function of the head kidney is reflected in the enriched term GO:0060218 'hematopoietic stem cell differentiation'.

Gill clusters

In salmon, two SOM clusters showed gill-specific expression (Extended Data Fig. 4; Supplementary Fig. 22), capturing 3,169 genes (6.7% of the tissue transcriptome). Trout similarly had two gill-specific SOM clusters (Extended Data Fig. 4; Supplementary Fig. 23), capturing 1,662 genes (4.3% the tissue transcriptome). In both species, enriched GO terms for these gill clusters (Supplementary Figs. 26-27) captured many immune terms, including GO:0006955 'immune response', GO:0060326 'cell chemotaxis', GO:0019882 'antigen processing and presentation' and GO:0031640 'killing of cells of other organism', highlighting the gill's key immune functions. Enriched GO terms associated with waste excretion and homeostatic function were also shared across both species (Supplementary Figs. 26-27), including GO:0072488 'ammonium transmembrane transport' and GO:0098655 'cation transmembrane transport'. Other enriched GO terms associated with development and regulation of the circulatory system reflect the highly vascularized nature of the gill as the key respiration site.

Distal intestine clusters

In salmon and trout, two SOM clusters showed distal intestine-specific expression, comprising 2,249 and 2,079 genes, respectively (Extended Data Fig. 4; Supplementary Figs. 22-23). One notable species difference was evidence for higher gill expression heterogeneity in trout, with one SOM cluster presenting lower expression in mature females, and the other presenting lower expression in mature males, while remaining at constant levels in immature individuals of both sexes (Extended Data Fig. 4; Supplementary Figs. 22-23). This observation is consistent with sexual dimorphism in intestine physiology linked to sexual maturation in trout. Enriched GO terms for salmonid gill SOM clusters captured the expected physiological roles of the distal intestine, including metabolism, ion transport and extracellular matrix organisation, as well as terms associated with muscle and circulatory system function.

Constitutive clusters

Several SOM clusters captured genes showing homogenous expression across tissues, sexes and maturation stages in both species. In salmon, four such 'constitutive' SOM clusters captured 9,458 genes (20% of the tissue transcriptome) (Supplementary Fig. 22). In trout, three constitutive SOM clusters captured 6,179 genes (16% of the tissue transcriptome) were captured (Supplementary Fig. 23). Enriched GO terms in the constitutive SOM clusters for both species largely represent housekeeping functions shared across cells types, including translation, histone modification, protein modification and protein catabolism, ncRNA metabolism, DNA repair and biosynthesis, ribosome biogenesis, ATP biosynthesis, and GO terms associated with mRNA processing, modification and transport, along with other functions associated with cell viability (Supplementary Tables 8-9).

Multi-expression clusters

For both the salmon and trout datasets, an interesting group of SOM clusters showed upregulation in more than one tissue, potentially highlighting shared biological processes among different groups of tissues. In salmon, nine multi-expression SOM clusters were captured (Supplementary Fig. 22). Four were up-regulated in ovary and either brain, muscle, liver or head kidney. Three further salmon SOM clusters showed up-regulation in brain and one each of testes, muscle or distal intestine. The remaining two multi-expression SOM clusters were up-regulated specifically in head-kidney and liver, or specifically in head-kidney and gill. Eleven trout multi-expression SOM clusters were observed (Supplementary Fig. 23). Four showed upregulation in ovary and either brain, testes, muscle or head kidney, with an ovary plus head-kidney cluster also showing up-regulation in testes. Three multi-expression clusters were expressed in brain and either testes, muscle or head kidney. The remaining multi-expression clusters were gill plus intestine, muscle plus liver, muscle plus liver plus head kidney, and head kidney plus gill/intestine.

Supplementary Text 8. Additional text on regulatory element evolution (supporting Fig. 4)

72.8% of active promoters were found in Early rediploidization regions, with 48.5%, 22.8% and 28.8% belonging to the Shared, Alignable only, and Exclusive categories, respectively (Supplementary Table 12). A similar proportion of active enhancers (74.8%) were located in Early regions, with 25.9%, 46.2% and 27.8% belonging to the same respective categories. A larger proportion of active promoters (67.0%, $p < 0.0001$, Fisher's Exact Test) and enhancers (55.1%, $p < 0.00001$, Fisher's Exact Test) belonged to the Shared category in Late compared to Early regions (Supplementary Table 12). Reciprocally, fewer active promoters (14.4%, $p < 0.0001$, Fisher's Exact Test) and enhancers (8.34%, $p < 0.0001$, Fisher's Exact Test) belonged to the Exclusive category in Late regions. For active promoters and enhancers, 22.8% vs. 18.6% ($p < 0.0001$, Fisher's Exact Test) and 46.2 % vs. 36.6% ($p = <0.0001$, Fisher's Exact Test) belonged to the Alignable only category in Early vs. Late regions, respectively.

While a smaller proportion of active promoters and enhancers were located in Late regions, they show increased density compared to Early regions (one promoter every 74.5 kb vs. 116 kb, and one enhancer every 57.9 kb vs. 80.1 kb, respectively) when normalised to the size of each region.

Supplementary Text 9. Differential enrichment of motifs in enhancer-CNEs across different CNE phylogenetic age categories.

The oldest CNEs are highly enriched for Homeodomain-related motifs and TFs, comprising 45% and 38.3% of the top-60 uniquely enriched motifs for Euteleostomi and Actinopteri, compared to 6.7% and 5% for Teleostei and Protacanthopterygii, respectively (Supplementary Fig. 38). The most strongly enriched motifs for Euteleostomi CNEs implicates master regulators of development, cell-type specification, pluripotency, embryonic patterning and organogenesis e.g. Nkx6-1, Tgif1, Barhl1/2, Alx1, Hesx1, Pbx1, Pbx2, Hoxb, Cux1/2, Pax7/3, Sox9/10 and Nanog/Sox2/Pouf5f. The Actinopteri CNEs were also highly enriched for developmental regulators, but with strongest enrichment for a greater proportion of Sox and C2H2 zinc finger motifs (Supplementary Table 21). Most development-associated motifs and TFs enriched in the oldest CNEs are highly depleted in the younger CNE age categories. The Teleostei age category was more enriched for C2H2 zinc finger-associated and bZIP motifs (25% and 13.3% of top 60 enriched motifs), with the top motif being for Zeb1, a master regulator of cell-cell adhesion [35]. The most enriched motifs in this CNE age category implicate regulators of stress and adaptive environmental responses (EGRs, FosB, TEADs, RREB1), chromatin architecture (CTCF), metabolism (PPARG), muscle development (Myf5, MEF2) and eye/neural development (Pax6). The youngest CNE category (Protacanthopterygii) was dominated by bZIP and Nuclear Receptor motifs (23% and 20 % of top 60 enriched motifs), uniquely implicating regulators of signal/hormone responsive processes impacting metabolism, sexual development, detoxification and immune responses and immune cell function (e.g., NR3C1/Glucocorticoid Receptor; AR/Androgen receptor; PR/Progesterone Receptor; NFE2L2, MAFK, Stat5, Batf/Jun, EBF1).

Supplementary References

1. Lien, S. *et al.* The Atlantic salmon genome provides insights into rediploidization. *Nature* **533**, 200–205 (2016).

2. Robertson, F. M. *et al.* Lineage-specific rediploidization is a mechanism to explain time-lags between genome duplication and evolutionary diversification. *Genome Biol.* **18**, (2017).

3. Gorodilov, Y. N. Description of the early ontogeny of the Atlantic salmon, Salmo salar, with a novel system of interval (state) identification. *Environ. Biol. Fishes* **47**, 109–127 (1996).

4. Chomczynski, P. & Sacchi, N. The single-step method of RNA isolation by acid guanidinium thiocyanate-phenol-chloroform extraction: twenty-something years on. *Nat. Protoc.* **1**, 581–585 (2006).

5. Corces, M. R. *et al.* An improved ATAC-seq protocol reduces background and enables interrogation of frozen tissues. *Nat. Methods* **14**, 959–962 (2017).

6. Chew, J.-L. *et al.* Reciprocal transcriptional regulation of Pou5f1 and Sox2 via the Oct4/Sox2 complex in embryonic stem cells. *Mol. Cell. Biol.* **25**, 6031–6046 (2005).

7. Lee, M. T. *et al.* Nanog, Pou5f1 and SoxB1 activate zygotic gene expression during the maternal-to-zygotic transition. *Nature* **503**, 360–364 (2013).

8. Rikin, A. & Evans, T. The tbx/bHLH transcription factor mga regulates gata4 and organogenesis. *Dev. Dyn.* **239**, 535–547 (2010).

9. Howard, A. G., 4th *et al.* An atlas of neural crest lineages along the posterior developing zebrafish at single-cell resolution. *Elife* **10**, (2021).

10. Kim, J., Lo, L., Dormand, E. & Anderson, D. J. SOX10 maintains multipotency and inhibits neuronal differentiation of neural crest stem cells. *Neuron* **38**, 17–31 (2003).

11. Sabel, J. L. *et al.* Maternal Interferon Regulatory Factor 6 is required for the differentiation of primary superficial epithelia in Danio and Xenopus embryos. *Dev. Biol.* **325**, 249–262 (2009).

12. Santos-Pereira, J. M., Gallardo-Fuentes, L., Neto, A., Acemel, R. D. & Tena, J. J. Pioneer and repressive functions of p63 during zebrafish embryonic ectoderm specification. *Nat. Commun.* **10**, 3049 (2019).

13. Carapuço, M., Nóvoa, A., Bobola, N. & Mallo, M. Hox genes specify vertebral types in the presomitic mesoderm. *Genes Dev.* **19**, 2116–2121 (2005).

14. Molkentin, J. D. *et al.* MEF2B is a potent transactivator expressed in early myogenic lineages. *Mol. Cell. Biol.* **16**, 3814–3824 (1996).

15. Hinits, Y. *et al.* Zebrafish Mef2ca and Mef2cb are essential for both first and second heart field cardiomyocyte differentiation. *Dev. Biol.* **369**, 199–210 (2012).

16. Kiyama, T. *et al.* Essential roles of mitochondrial biogenesis regulator Nrf1 in retinal development and homeostasis. *Mol. Neurodegener.* **13**, 56 (2018).

17. Lau, H. H., Ng, N. H. J., Loo, L. S. W., Jasmen, J. B. & Teo, A. K. K. The molecular functions of hepatocyte nuclear factors - In and beyond the liver. *J. Hepatol.* **68**, 1033–1048 (2018).

18. Ktistaki, E. & Talianidis, I. Modulation of hepatic gene expression by hepatocyte nuclear factor 1. *Science* **277**, 109–112 (1997).

19. Kotulkar, M., Robarts, D. R. & Apte, U. HNF4α in hepatocyte health and disease. *Semin. Liver Dis.* **43**, 234–244 (2023).

20. Tong, J. *et al.* Hepatic interferon regulatory factor 6 alleviates liver steatosis and metabolic disorder by transcriptionally suppressing peroxisome proliferator-activated receptor γ in mice. *Hepatology* **69**, 2471–2488 (2019).

21. Gonzalez-Rellan, M. J. *et al.* Hepatic p63 regulates glucose metabolism by repressing SIRT1. *Gut* **72**, 472–483 (2023).

22. Tutukova, S., Tarabykin, V. & Hernandez-Miranda, L. R. The role of Neurod genes in brain development, function, and disease. *Front. Mol. Neurosci.* **14**, 662774 (2021).

23. Hu, S. *et al.* Nrf1 is an indispensable redox-determining factor for mitochondrial homeostasis by integrating multi-hierarchical regulatory networks. *Redox Biol.* **57**, 102470

(2022).

24. Liu, K. *et al.* Molecular mechanism of specific DNA sequence recognition by NRF1. *Nucleic Acids Res.* **52**, 953–966 (2024).

25. Coschiera, A. *et al.* Primary cilia promote the differentiation of human neurons through the WNT signaling pathway. *BMC Biol.* **22**, 48 (2024).

26. Cai, C. *et al.* Transcriptional and open chromatin analysis of bovine skeletal muscle development by single-cell sequencing. *Cell Prolif.* **56**, e13430 (2023).

27. Hughes, K. L., Abshire, E. T. & Goldstrohm, A. C. Regulatory roles of vertebrate Nocturnin: insights and remaining mysteries. *RNA Biol.* **15**, 1255–1267 (2018).

28. Moore, A. F. T., Gentry, R. C. & Koculi, E. DbpA is a region-specific RNA helicase. *Biopolymers* **107**, e23001 (2017).

29. Bally-Cuif, L., Goutel, C., Wassef, M., Wurst, W. & Rosa, F. Coregulation of anterior and posterior mesendodermal development by a hairy-related transcriptional repressor. *Genes Dev.* **14**, 1664–1677 (2000).

30. Martin, M. & Akhmanova, A. Coming into focus: Mechanisms of microtubule minus-end organization. *Trends Cell Biol.* **28**, 574–588 (2018).

31. Dranow, D. B. *et al.* Bmp15 is an oocyte-produced signal required for maintenance of the adult female sexual phenotype in zebrafish. *PLoS Genet.* **12**, e1006323 (2016).

32. Escobar-Aguirre, M., Elkouby, Y. M. & Mullins, M. C. Localization in oogenesis of maternal regulators of embryonic development. *Adv. Exp. Med. Biol.* **953**, 173–207 (2017).

33. Reynolds, A. *et al.* RNF212 is a dosage-sensitive regulator of crossing-over during mammalian meiosis. *Nat. Genet.* **45**, 269–278 (2013).

34. Chalamalasetty, R. B. *et al.* Mesogenin 1 is a master regulator of paraxial presomitic mesoderm differentiation. *Development* **141**, 4285–4297 (2014).

35. Vannier, C., Mock, K., Brabletz, T. & Driever, W. Zeb1 regulates E-cadherin and Epcam (epithelial cell adhesion molecule) expression to control cell behavior in early zebrafish development. *J. Biol. Chem.* **288**, 18643–18659 (2013).
