# Supplementary material for: Developmental contexts of gene-regulatory evolution following whole genome duplication in salmonids
Source: Nat Ecol Evol. 2026 Sep 29;10(10):1959–81. doi: 10.1038/s41559-026-03171-6 (PMC13642036; doi:10.1038/s41559-026-03171-6)
Supplement: Supplementary file 2 [file 41559_2026_3171_MOESM2_ESM.pdf]

nature portfolio

Corresponding author(s): Daniel J Macqueen

Last updated by author(s): 18/07/2026

Reporting Summary

Nature Portfolio wishes to improve the reproducibility of the work that we publish. This form provides structure for consistency and transparency in reporting. For further information on Nature Portfolio policies, see our Editorial Policies and the Editorial Policy Checklist.

Statistics

For all statistical analyses, confirm that the following items are present in the figure legend, table legend, main text, or Methods section.

n/a	Confirmed	
☐	☒	The exact sample size (*n*) for each experimental group/condition, given as a discrete number and unit of measurement
☐	☒	A statement on whether measurements were taken from distinct samples or whether the same sample was measured repeatedly
☐	☒	The statistical test(s) used AND whether they are one- or two-sided<br>*Only common tests should be described solely by name; describe more complex techniques in the Methods section.*
☐	☒	A description of all covariates tested
☐	☒	A description of any assumptions or corrections, such as tests of normality and adjustment for multiple comparisons
☐	☒	A full description of the statistical parameters including central tendency (e.g. means) or other basic estimates (e.g. regression coefficient) AND variation (e.g. standard deviation) or associated estimates of uncertainty (e.g. confidence intervals)
☐	☒	For null hypothesis testing, the test statistic (e.g. *F*, *t*, *r*) with confidence intervals, effect sizes, degrees of freedom and *P* value noted<br>*Give P values as exact values whenever suitable.*
☒	☐	For Bayesian analysis, information on the choice of priors and Markov chain Monte Carlo settings
☐	☒	For hierarchical and complex designs, identification of the appropriate level for tests and full reporting of outcomes
☐	☒	Estimates of effect sizes (e.g. Cohen's *d*, Pearson's *r*), indicating how they were calculated

Our web collection on statistics for biologists contains articles on many of the points above.

Software and code

Policy information about availability of computer code

Data collection	No software was used for the purpose of data collection
Data analysis	All code required to reproduce the analyses and result reported is provided at: https://github.com/AQUA-FAANG/AQUA-FAANG-salmonids (https://doi.org/10.5281/zenodo.21410850 - see reference 70 in article).<br><br>Used for data analysis:<br>- nf-core pipeline rnaseq v3.8.1 (https://nf-co.re/rnaseq/3.8.1/)<br>- nf-core pipeline atacseq v1.2.1 (https://nf-co.re/atacseq/1.2.1/)<br>- nf-core pipeline chipseq v1.2.2 (https://nf-co.re/chipseq/1.2.2/)<br>- Umap (single version, https://bismap.hoffmanlab.org/)<br>- Blacklist (single version, https://github.com/Boyle-Lab/Blacklist/)<br>- ChromHMM v1.27, https://compbio.mit.edu/ChromHMM/)<br>- bedtools v2.30.0 (https://bedtools.readthedocs.io/en/latest/)<br>- GimmeMotifs v0.17.1 (https://gimmemotifs.readthedocs.io/en/master/reference.html)<br>- Progressive Cactus (https://github.com/ComparativeGenomicsToolkit/cactus/releases)<br>- minimap2 v 2.18-784r1015 (https://github.com/lh3/minimap2/releases)<br>- ComparativeGenomicsToolkit v2.2.1. (https://github.com/ComparativeGenomicsToolkit/Comparative-Annotation-Toolkit)<br>- preprocessCore v1.72.0 (https://www.bioconductor.org/packages/release/bioc/html/preprocessCore.html)<br>- kohonen R package v3.0.13 (https://cran.r-project.org/web/packages/kohonen/index.html)<br>- uwot v0.2.4 (https://cran.r-project.org/web/packages/uwot/index.html)<br>- complexHeatmap R package v2.26.0 (https://bioconductor.org/packages/release/bioc/html/ComplexHeatmap.html)

- ggalluvial R package v 0.12.5 (https://cran.r-project.org/web/packages/ggalluvial/index.html)
- clusterProfiler v4.18.4 (https://bioconductor.org/packages/release/bioc/html/clusterProfiler.html)
- AnnotationForge v1.52 (https://www.bioconductor.org/packages/release/bioc/html/AnnotationForge.html)
- biomaRt v2.66 (https://bioconductor.org/packages/release/bioc/html/biomaRt.html)
- GO.db v3.22 (https://www.bioconductor.org/packages/release/data/annotation/html/GO.db.html
- Bowtie2 v2.5.0 (https://github.com/BenLangmead/bowtie2/releases)
- SAMtools v1.5 (https://www.htslib.org/doc/1.5/samtools.html)
- CAGEr v2.11.4 (https://www.bioconductor.org/packages/release/bioc/html/CAGEr.html)
- phylentropy R v0.1.0 (https://cran.r-project.org/web/packages/philentropy/index.html)
- ggpubr R package v0.6.2 (https://cran.r-project.org/web/packages/ggpubr/index.html)
- mafTools (https://bioconductor.org/packages/release/bioc/vignettes/maftools/inst/doc/maftools.html; https://github.com/PoisonAlien/maftools)
- RepeatMasker v4.1.5 (https://github.com/Dfam-consortium/RepeatMasker)
- Gerp++ (https://github.com/tvkent/GERPplusplus?tab=readme-ov-file)
- Mclust R package v6.1.2 (https://cran.r-project.org/web/packages/mclust/index.html)
- tidyverse 2.0.0 R package – https://tidyverse.org/
- fuzzyjoin 0.1.6 R package – https://rpubs.com/hellojohncruz/fuzzyjoin
- openxlsx 4.2.8 R package – https://cran.r-project.org/web/packages/openxlsx/index.html
- RcolorBrewer 1.1.3 R package – https://cran.r-project.org/web/packages/RColorBrewer/index.html
- UpSetR - 1.4.1. R package - https://cran.r-project.org/web/packages/UpSetR/index.html

For manuscripts utilizing custom algorithms or software that are central to the research but not yet described in published literature, software must be made available to editors and reviewers. We strongly encourage code deposition in a community repository (e.g. GitHub). See the Nature Portfolio guidelines for submitting code & software for further information.

Data

Policy information about availability of data

All manuscripts must include a data availability statement. This statement should provide the following information, where applicable:

- Accession codes, unique identifiers, or web links for publicly available datasets
- A description of any restrictions on data availability
- For clinical datasets or third party data, please ensure that the statement adheres to our policy

All data required to reproduce and expand the results reported in this study have been shared. The raw sequencing datasets are available through the FAANG data portal (https://data.faang.org/projects/AQUA-FAANG). ENA accession numbers are shared in Supplementary Table 2, and as follows: PRJEB51855, PRJEB51854 and PRJEB53399 (salmon DevMap: RNA-seq, ATAC-seq and ChIP-seq, respectively); PRJEB51857, PRJEB51856 and PRJEB55010 (trout DevMap: RNA-seq, ATAC-seq and ChIP-seq, respectively); PRJEB47409, PRJEB47408 and PRJEB55063 (salmon BodyMap: RNA-seq, ATAC-seq and ChIP-seq, respectively); PRJEB57191, PRJEB57190 and PRJEB57956 (trout BodyMap: RNA-seq, ATAC-seq and ChIP-seq, respectively). Processed datasets are also provided via salmobase.org 34, including RNA-seq TPM values, ATAC-seq and ChIP-seq peak files, robust open chromatin regions, ChromHMM-annotated robust open chromatin regions, whole genome alignments, and our homology prediction. Supplementary data files and source data for all figures and extended data figures are shared at https://doi.org/10.5281/zenodo.21415252 111.

Research involving human participants, their data, or biological material

Policy information about studies with human participants or human data. See also policy information about sex, gender (identity/presentation), and sexual orientation and race, ethnicity and racism.

Reporting on sex and gender	Not applicable to study
Reporting on race, ethnicity, or other socially relevant groupings	Not applicable to study
Population characteristics	Not applicable to study
Recruitment	Not applicable to study
Ethics oversight	Not applicable to study

Note that full information on the approval of the study protocol must also be provided in the manuscript.

Field-specific reporting

Please select the one below that is the best fit for your research. If you are not sure, read the appropriate sections before making your selection.

☒ Life sciences ☐ Behavioural & social sciences ☐ Ecological, evolutionary & environmental sciences

For a reference copy of the document with all sections, see nature.com/documents/nr-reporting-summary-flat.pdf

Life sciences study design

All studies must disclose on these points even when the disclosure is negative.

Sample size	Sample sizes were not defined using power analysis. We aimed to have a minimum of n=3 per sample type per each performed sequencing assay, which aligns with best practise (e.g. ENCODE, FAANG functional annotation initiatives).
Data exclusions	None
Replication	Biological replication was used as per the above described sample size, and accounted for through robust statistical procedures described in the methods, which vary per analysis.
Randomization	Samples were not from different experimental conditions - fish were sampled at baseline status at different life-stages without any experimental manipulations. Sampling was done in each case randomly with respect to a larger population of fish or fish embryos. At embryonic stages, embryos were randomly sampled from a larger pool of available embryos. At adult stages, fish were sampled randomly from a larger population.
Blinding	Blinding was not relevant to this study, as we were sampling fish at baseline status without any experimental manipulations being tested that would require a blinding approach to influence the outcomes.

Reporting for specific materials, systems and methods

We require information from authors about some types of materials, experimental systems and methods used in many studies. Here, indicate whether each material, system or method listed is relevant to your study. If you are not sure if a list item applies to your research, read the appropriate section before selecting a response.

Materials & experimental systems

n/a	Involved in the study	
☐	☒	Antibodies
☐	☐	Eukaryotic cell lines
☐	☐	Palaeontology and archaeology
☐	☒	Animals and other organisms
☐	☐	Clinical data
☐	☐	Dual use research of concern
☐	☐	Plants

Methods

n/a	Involved in the study	
☐	☒	ChIP-seq
☐	☐	Flow cytometry
☐	☐	MRI-based neuroimaging

Antibodies

Antibodies used	All antibodies used were from Diagenode: H3K27ac: C15410196; H3K4me1: C15410194; H3K4me3: C1541000; H3K27me3: C15410195.
Validation	Our efforts to validate each antibody for the target salmonid species are provided in the supplementary information. Extensive validation data for each antibody used is provided on the Diagenode website. This includes evidence of use applications in diverse species including associated peer reviewed publications. See: https://www.diagenode.com/en/categories/histone-antibodies

Eukaryotic cell lines

Policy information about cell lines and Sex and Gender in Research

Cell line source(s)	Not applicable
Authentication	Not applicable
Mycoplasma contamination	Not applicable
Commonly misidentified lines (See ICLAC register)	Not applicable

Palaeontology and Archaeology

Specimen provenance	Not applicable
Specimen deposition	Not applicable

Dating methods	Not applicable

☐ Tick this box to confirm that the raw and calibrated dates are available in the paper or in Supplementary Information.

Ethics oversight	Not applicable

Note that full information on the approval of the study protocol must also be provided in the manuscript.

Animals and other research organisms

Policy information about studies involving animals; ARRIVE guidelines recommended for reporting animal research, and Sex and Gender in Research

Laboratory animals	Farmed Atlantic salmon and farmed rainbow trout were sampled either as embryos or at two adult life stages (sexually immature and sexually matured - maturation defined using histology as described in supplementary information)
Wild animals	No wild animals were used
Reporting on sex	Sex was explicitly considered in the sampling. For the two adult life stages, an equal number of male and female fish were sampled and used to produce the reported functional genomics datasets.
Field-collected samples	Not applicable
Ethics oversight	This research complies with relevant ethical regulations. Sampling of fish embryos (DevMap) was done prior to protected life stages. For tissue sampling in rainbow trout adults (BodyMap), the experiment was conducted at the INRAE Fish Physiology and Genomics Laboratory (LPGP) experimental facilities (IE LPGP, permit number C35-238-6, Rennes, France), and approved by the animal welfare committee for the animal experimentation of LPGP under the authorization number T-2020-16-YG-AL. Tissue sampling in Atlantic salmon adults (BodyMap) was conducted at the Center for Fish Trials (NMBU, Ås Norway), an approved facility that is registered with the Norwegian Food Safety Authority (Mattilsynet) under locality number 10362. Animal treatment and experiments were conducted according to institutional and national animal guidelines, as well as laws that regulate scientific use of experimental animals in Norway (EU directive 2010/63/EU, FOR-2017-04-05-451).

Note that full information on the approval of the study protocol must also be provided in the manuscript.

Clinical data

Policy information about clinical studies

All manuscripts should comply with the ICMJE guidelines for publication of clinical research and a completed CONSORT checklist must be included with all submissions.

Clinical trial registration	Not applicable
Study protocol	Not applicable
Data collection	Not applicable
Outcomes	Not applicable

Dual use research of concern

Policy information about dual use research of concern

Hazards

Could the accidental, deliberate or reckless misuse of agents or technologies generated in the work, or the application of information presented in the manuscript, pose a threat to:

No	Yes	
☒	☐	Public health
☒	☐	National security
☒	☐	Crops and/or livestock
☒	☐	Ecosystems
☒	☐	Any other significant area

Experiments of concern

Does the work involve any of these experiments of concern:

No	Yes	
☒	☐	Demonstrate how to render a vaccine ineffective
☒	☐	Confer resistance to therapeutically useful antibiotics or antiviral agents
☒	☐	Enhance the virulence of a pathogen or render a nonpathogen virulent
☒	☐	Increase transmissibility of a pathogen
☒	☐	Alter the host range of a pathogen
☒	☐	Enable evasion of diagnostic/detection modalities
☒	☐	Enable the weaponization of a biological agent or toxin
☒	☐	Any other potentially harmful combination of experiments and agents

Plants

Seed stocks	Not applicable
Novel plant genotypes	Not applicable
Authentication	Not applicable

ChIP-seq

Data deposition

☒ Confirm that both raw and final processed data have been deposited in a public database such as GEO.

☒ Confirm that you have deposited or provided access to graph files (e.g. BED files) for the called peaks.

Data access links<br>*May remain private before publication.*	All ChIP-seq datasets are provided in final processed form on salmobase.org. Accession numbers for the raw data have been provided above.
Files in database submission	No applicable
Genome browser session<br>(e.g. UCSC)	Unclear what is needed here.

Methodology

Replicates	Please see Table S1 (details of all replicates) and Figures S3-S4
Sequencing depth	Please see Table S2 for per sample information
Antibodies	Provided in a section above.
Peak calling parameters	Please see: https://github.com/AQUA-FAANG/AQUA-FAANG-salmonids/blob/main/nfcore-pipelines/AtlanticSalmon/scripts/chipseq.sh
Data quality	Irreproducible Discovery Rate was used to empirically define open chromatin regions based on reproducible signal biological replicates, which were then annotated using an integration of ChIP-seq datasets (4 histone marks) using ChromHMM. The quality of annotations was tested using an independent method (CAGE) and by detailed analyses of transcription factor motif enrichment per sample type.
Software	BWA (alignment), MACS2 (peak calling), Umap and Blacklist (blacklist definition), ChromHMM (integration and definiton of chromatin state)

Flow Cytometry

Plots

Confirm that:

- [ ] The axis labels state the marker and fluorochrome used (e.g. CD4-FITC).
- [ ] The axis scales are clearly visible. Include numbers along axes only for bottom left plot of group (a 'group' is an analysis of identical markers).
- [ ] All plots are contour plots with outliers or pseudocolor plots.
- [ ] A numerical value for number of cells or percentage (with statistics) is provided.

Methodology

Sample preparation	Not applicable
Instrument	Not applicable
Software	Not applicable
Cell population abundance	Not applicable
Gating strategy	Not applicable

- [ ] Tick this box to confirm that a figure exemplifying the gating strategy is provided in the Supplementary Information.

Magnetic resonance imaging

Experimental design

Design type	Not applicable
Design specifications	Not applicable
Behavioral performance measures	Not applicable

Acquisition

Imaging type(s)	Not applicable
Field strength	Not applicable
Sequence & imaging parameters	Not applicable
Area of acquisition	Not applicable

Diffusion MRI ☐ Used ☐ Not used

Preprocessing

Preprocessing software	Not applicable
Normalization	Not applicable
Normalization template	Not applicable
Noise and artifact removal	Not applicable
Volume censoring	Not applicable

Statistical modeling & inference

Model type and settings	Not applicable
Effect(s) tested	Not applicable

Specify type of analysis: ☐ Whole brain ☐ ROI-based ☐ Both

Statistic type for inference

(See Eklund et al. 2016)

Not applicable

Correction

Not applicable

Models & analysis

n/a	Involved in the study
☐	☐ Functional and/or effective connectivity
☐	☐ Graph analysis
☐	☐ Multivariate modeling or predictive analysis

Functional and/or effective connectivity

Not applicable

Graph analysis

Not applicable

Multivariate modeling and predictive analysis

Not applicable
