# Supplementary material for: Developmental contexts of gene-regulatory evolution following whole genome duplication in salmonids
Source: Nat Ecol Evol. 2026 Sep 29;10(10):1959–81. doi: 10.1038/s41559-026-03171-6 (PMC13642036; doi:10.1038/s41559-026-03171-6)
Supplement: Supplementary file 3 [file 41559_2026_3171_MOESM3_ESM.pdf]

nature portfolio

Peer Review File

Developmental contexts of gene-regulatory evolution following whole genome duplication

Corresponding Author: Professor Daniel Macqueen

Version 0:

Decision Letter:

10th March 2026

*Please ensure you delete the link to your author homepage in this e-mail if you wish to forward it to your co-authors.

Dear Dan,

Your manuscript entitled "Salmonids reveal principles of regulatory evolution following autotetraploidization" has now been seen by 3 reviewers, whose comments are attached. The reviewers have raised a number of concerns which will need to be addressed before we can offer publication in Nature Ecology & Evolution. We will therefore need to see your responses to the criticisms raised and to some editorial concerns, along with a revised manuscript, before we can reach a final decision regarding publication.

We therefore invite you to revise your manuscript taking into account all reviewer and editor comments. Please highlight all changes in the manuscript text file in Microsoft Word format.

We are committed to providing a fair and constructive peer-review process. Do not hesitate to contact us if there are specific requests from the reviewers that you believe are technically impossible or unlikely to yield a meaningful outcome.

When revising your manuscript:

* Include a “Response to reviewers” document detailing, point-by-point, how you addressed each reviewer comment. If no action was taken to address a point, you must provide a compelling argument. This response will be sent back to the reviewers along with the revised manuscript.

* If you have not done so already please begin to revise your manuscript so that it conforms to our Article format instructions at http://www.nature.com/natecolevol/info/final-submission. Refer also to any guidelines provided in this letter.

* Include a revised version of any required reporting checklist. It will be available to referees (and, potentially, statisticians) to aid in their evaluation if the manuscript goes back for peer review. A revised checklist is essential for re-review of the paper.

* Extended Data Figures - please ensure that any supplementary figures and tables that are crucial to the manuscript’s conclusions are converted into Extended Data figures and tables to increase visibility of these data. Extended Data figures and tables are online-only (present in the online PDF and full-text HTML versions of the paper), peer-reviewed display items that provide essential background to the article but are not included in the main article due to space constraints. A maximum of ten Extended Data display items (figures and tables) is permitted.

Please use the link below to submit your revised manuscript and related files:

Link Redacted

Note: This URL links to your confidential home page and associated information about manuscripts you may have submitted, or that you are reviewing for us. If you wish to forward this email to co-authors, please delete the link to your homepage.

We hope to receive your revised manuscript within four to eight weeks. If you cannot send it within this time, please let us know. We will be happy to consider your revision so long as nothing similar has been accepted for publication at Nature Ecology & Evolution or published elsewhere.

Nature Ecology & Evolution is committed to improving transparency in authorship. As part of our efforts in this direction, we are now requesting that all authors identified as 'corresponding author' on published papers create and link their Open Researcher and Contributor Identifier (ORCID) with their account on the Manuscript Tracking System (MTS), prior to acceptance. ORCID helps the scientific community achieve unambiguous attribution of all scholarly contributions. You can create and link your ORCID from the home page of the MTS by clicking on 'Modify my Springer Nature account'. For more information please visit please visit www.springernature.com/orcid.

Please do not hesitate to contact me if you have any questions or would like to discuss these revisions further.

We look forward to seeing the revised manuscript and thank you for the opportunity to review your work.

[redacted]

Reviewer expertise:

Reviewer #1: polyploidy, gene regulation

Reviewer #2: gene regulatory evolution, salmonids

Reviewer #3: polyploidy, gene regulation

Reviewers' comments:

Reviewer #1 (Remarks to the Author):

This manuscript presents an incredible resource from the AQUA-FAANG consortium, including 771 high-throughput sequencing libraries (RNA-seq, ATAC-seq, ChIP-seq) across development and adult tissues in two salmonid species. The integrative analysis provides unprecedented insights into the regulatory evolution of ohnologs following the salmonid-specific 4R whole-genome duplication and the subsequent two-wave rediploidization process.

By characterizing the highly dynamic and variable profiles of ohnolog expression divergence, this study found the highest constraint of expression divergence at the late somitogenesis/segmentation stages shared between early and late rediploidzation resolved ohnolog pairs across two species, implicating the same strong purifying selection to constrain the divergence of duplicated genes. Delving into the mechanistic how, the following analyses convincingly link the ohnolog expression divergence pattern with the cis-regulatory control of enhancers, as well as the role TE insertion to evolve new enhancer sequences.

The manuscript is well-written given the complexity of the data and analyses. My comments are primarily aimed at increasing clarity, transparency, and reproducibility. They should be straightforward to address.

1. Regarding the authors' claim of 'high-quality sequencing datasets', I would recommend more rigorous technical validation, such as adding quality control metrics in Table S3 for the RNA-seq, ATAC-seq, and ChIP-seq libraries. Specifically, this should include mapping rates, duplication rates, and—for the epigenomic datasets—FRiP (Fraction of Reads in Peaks) scores.

- For replication conditions, a BAM coverage correlation matrix (Pearson or Spearman) or a heatmap would greatly strengthen the evidence for the reproducibility and technical integrity of their replicates. This is particularly important for the ChIP-seq and ATAC-seq data, where signal reproducibility is a ENCODE standard.

- Fig S5-S9: ATAC and ChIP peaks with genomic annotation were showed for replicated samples per specific sample condition, which showed comparable portions of annotated features. However, certain samples did stand out (e.g. trout_LS_1 in FigS5, salmon_brain_2 in FigS9). Please also include the IDR merged peak number for each set of replicates.

2. The experimental design is largely consistent between Atlantic salmon and rainbow trout, except for the lacking of Gonad ChIP-seq in trout.

- Could this explain the higher number of open chromatin regions and the higher CAGE support rate observed in salmon?

- Could these differences affect downstream analyses between species? Actually later analyses involving ohnolog pairs and cis-regulatory elements were mainly on Salmon (Fig 3-5), without equivalent data in trout.

3. The gene and regulatory element datasets defined are central to the analyses of duplicated gene regulation evolution: A total 10,521 high-confidence ohnolog pairs conserved in both species and 5,832 'singletons', and 26,157 genes and 194,528 promoters or enhancers. Are those the same set used for the DevMap and BodyMap analyses in the result section "Dynamic genome regulation across ontogeny and tissues"? If so, does that mean at least one third of genes in each genome were excluded? Please clarify and discuss any potential biases would be introduced due to limited

representativeness.

4. While SOM is an effective tool for visualizing high-dimensional expression and chromatin accessibility patterns, the manuscript would benefit from a more detailed justification of the parameters used and quality control.

- Please specify how the grid dimensions (e.g., x-y size, topology) were determined. Was an optimization criterion used (e.g., minimal quantization error)?

- For the distinction between "constitutive" and "condition-specific" SOM clusters (lines 848–852, Figs. S18–21): beyond visual inspection, were any quantitative thresholds applied (e.g., coefficient of variation, proportion of samples showing activity)? Explicitly stating the criteria would improve reproducibility.

5. About cross-species SOM comparison, Figure 2 is rich in information but quite difficult to read, particularly the Sankey-style diagrams linking salmon and trout SOM clusters. I was confused about how the Sankey Diagrams correspond to heatmap rows (not really I suppose) and how the flow colors correspond to orthology.

- I recommend splitting the heatmap and the Sankey/band plots into separate panels with clear labels.

- The color legend for the orthologous gene ratio (light red bands) and syntenic region counts (brown bands) should be placed directly adjacent to the corresponding plots.

- Regarding the UMAP plots: the caption states that "dots represent mean normalised TPM values of individual genes (A) or mean normalised ATAC-seq read intensity values for individual robust open chromatin regions (B)". This phrasing is ambiguous. I assume the UMAP embeddings are calculated from the normalized expression/accessibility profiles across stages, and each dot corresponds to a gene or a chromatin region. Please rephrase to clarify that the UMAP visualizes the multivariate profile of each feature, not a single mean value.

6. The division of ohnolog pairs into "Early" and "Late" rediploidization classes is central to the study. It would be very informative to know whether these two classes are enriched in specific SOM clusters from the DevMap or BodyMap analyses. E.g., are Early-resolved ohnologs overrepresented in clusters associated with later development or specific adult tissues?

- Can the authors trace how individual ohnolog pairs have partitioned into different SOM clusters (e.g., one copy in cluster A, the other in cluster B) and whether this partitioning differs between Early and Late rediploidization pairs? Such an analysis would directly link the rediploidization timeline to regulatory diversification.

7. Lines 307–309 and Fig. 5F show that "Exclusive" enhancers overlap TEs to a much greater extent than "Shared" enhancers. One potential confounding factor is that exclusive enhancers might be located farther from transcription start sites on average, and TE content is known to increase with distance from genes. To strengthen the claim that TE insertions actively contribute to enhancer innovation/divergence (rather than merely reflecting a lack of constraint in distal regions), I suggest repeating the TE-overlap analysis after binning enhancers by distance to the nearest TSS (e.g., 0–10 kb, 10–50 kb, >50 kb). This would control for the genomic context and provide a more rigorous test.

Reviewer #1 (Remarks on code availability):

I confirm that the repository is live and contains the scripts and workflows used for the major analyses presented.

Reviewer #2 (Remarks to the Author):

Baudement and colleagues report a large and impressive collaborative dataset of gene expression and gene regulatory regions in different developmental stages and tissues in two salmonid species. They use this vast dataset to understand regulatory element evolution across genome regions experiencing different rediploidization histories following whole genome duplication. The dataset could (will) be used to address many other questions of gene regulatory evolution and I think the paper's focus on rediploidization and ohnolog evolution is justified and original. This is the most impressive dataset to address these questions I have yet seen. The vast bioinformatic analyses are nearly immaculate and a massive undertaking that has produced results of high uniqueness and value for the wider research community. The fact that results, data, and protocols are shared in extensive detail is exemplary and will be highly appreciated by researchers not only studying salmonids, but also other vertebrates.

The vast scale of data and results also poses challenges in their presentation. Although I agree with the focus on regulatory evolution following WGD, there are some loose ends in the interpretation of results from analyses of ohnolog expression divergence, which I point out below. I also think that all sections need to be revised for consistency of motivation (of the study) and use of language, which is quite variable. In a large collaboration like this I think this is the responsibility of the last authors.

Main comments:

1. Embryonic development follows very different rates in Atlantic salmon and rainbow trout following from their autumn vs spring spawning life cycle. How the developmental rate is taken into account in the analyses needs to be better explained and put this into perspective when discussing the results. For example, is the regulatory architecture of stage-specific expression and regulatory activity conserved (and therefore simply accelerated in trout compared to salmon)? Can you identify architecture elements (e.g. gene modules, ohnologs, SOMs, regulatory elements) that are responding to developmental time (rate) rather than stage?

2. Lines 66-67: I suggest making this motivation statement more specific. How does knowing the functional outcomes of rediploidizations help to understand species radiations?

3. Lines 77-78: I understand this argument of having one “genetic background” to look at the functional divergence of ohnologs in different stages of rediploidization - but could you articulate here how do you take into account that rediploidization of some genes may influence further selection (“evolutionary degrees of freedom”) of other genes. Can we claim that evolution of some ohnologs is independent of evolutionary endpoints in other ohnologs? My concern here is that if ohnolog evolution is interdependent on evolution of other ohnologs, evolutionary trends in, say, late rediploidization ohnologs becomes dependent on rediploidization of early rediploidization ohnologs, and the “stratification of ohnolog divergence times” is not independent of which ohnologs reverted to diploidy first. In this case you would not be looking at different evolutionary “strata” but an interdependent trajectory, which are not the same.

4. Section entitled “Ontogeny governs the evolution of enhancer-CNEs following WDG”: While I find the results of this section interesting in describing general tendencies of enhancer-CNE conservation across phylogenies and rediploidization states, many of the results here describe general statistics without much biological interpretation. Could you not do a sequence motif analysis for the CNEs? This could potentially bring a lot of biological insight into what signaling pathways are conserved across WGD/phylogeny/ontogeny and what pathways are evolvable.

5. Section entitled “Enhancer evolution is coupled to ohnolog expression regulation”: This section is clearly in need of rephrasing and clarification of sentences. It is hard to follow what are the exact results and what is being compared. The style in this section is also markedly different from the rest of the manuscript and I recommend editing for consistency by the last authors (e.g. word use, post-hoc justifications of analyses, etc.). The results here are also reported in a more list-like manner and I recommend balancing this section with more interpretation of the results. Specific comments below.

Lines 249-250: The motivation for this analysis is weak and unspecific – please elaborate in more specific terms what new knowledge of the evolutionary process this analysis creates.

Lines 250-252: I suggest rephrasing this sentence because it is not immediately clear what is the message here.

Lines 264-274: This paragraph is more wordy than other paragraphs and the message could be condensed here without elaborate justification of the analyses.

Lines 265-268: How do you take into account total expression level in this analysis? My concern here is that lower expression correlation may be driven by lower expression, so can these classes simply reflect groups of genes with higher or lower expression instead of variable or conserved regulation?

Lines 275-281: How do you take into account the effect of expression noise in this analysis? My concern here is that if you define the regulatory classes based on expression correlation, genes with lower correlation may be those with lower overall expression - in that case finding more active enhancers in "conserved regulation" class may simply reflect the higher expression of genes in that class.

Lines 284-288: It is not clear how these two analyses (questions) are different, please rephrase.

Lines 288-289: Please quantify these statements of "decreased" and "more variable" in some way.

Lines 291-293: Please state stronger than *what*.

Lines 293-295: It is not clear what is being compared here.

Lines 296-297: This is not a strong conclusion and it's self-evident that regulatory elements will evolve across time - this conclusion is simply saying regulatory elements evolve, it tells very little *how* regulatory element evolution influences ohnolog expression evolution. Please elaborate.

– Section end –

6. Lines 397-399: In this context it would be helpful if you could succinctly explain how this perspective is novel from previous knowledge.

Additional comments:

7. Line 101: Please briefly explain what is “robust” in this context.

8. Line 129: “Closely related” is a bit misleading in this context because you have not analyzed associated changes in SOM cluster genes and e.g. their proximate ATAC-seq peaks. I think "similar" would be more appropriate here.

9. Lines 162-163: Could you please elaborate briefly on the reasons/logic on why ohnolog expression divergence decreases across embryogenesis. How does this reflect on some more general observation?

10. Line 176: Can you please elaborate briefly why do you think the difference in divergence between early and late rediploidization genes is smallest in ovaries and markedly different from other tissues.

11. Lines 193-194: Can you please briefly elaborate what does this mean in terms of gene (regulatory) evolution in general. Why is expression evolution more constrained in ohnologs vs singletons during later stages of embryogenesis? What does this tell us about the functional roles of (genes retained as) ohnologs vs singletons in development?

12. Lines 197-198: Why is this pattern conserved across these main osmoregulatory organs? Is it driven by a specific set of transcripts that have conserved roles (e.g. in osmoregulation)?

13. Lines 204-205: Could you elaborate on this conclusion/argument in a more explicit manner. For example, what is meant with “pleiotropic constraints” in this context?

14. Line 242: Please specify female or male gonads (or both).

15. Lines 302-303: It is not clear what is meant with "constraints" in this context.

16. Lines 299-309: I’d like to point out that all but two sentences in this paragraph start with a subordinate clause (indeed… on the other hand… yet… to explore these questions… overall…). My suggestion is to use more direct sentences and to minimize the use of such style.

17. Line 339: "Influences" indicates a causal relationship so I think "associates" would be more appropriate here.

18. Line 367: I think "reflect" is more suiting than "shape" because you have not argued/showed whether the causality goes from sequence conservation -> regulatory activity or vice versa.

19. Line 403: Is the inference of "selection" really justified here? You have not measured selection (in this case it seems to be implied as relaxed purifying selection), and increased variation could in fact be beneficial and promoted by natural selection.

20. Line 413: I would rephrase “ideal” here - ideal means that there is no better model system, and many arguments could be made pointing to the contrary.

Reviewer #3 (Remarks to the Author):

The authors reported results from analysis of multiple sets of genomic, chromatin, and gene expression data and tested evolutionary consequences of tetraploidization in salmonids. They found the changes were largely dependent on developmental stages and tissue-specificity. Salmonids are among vertebrate species for the study of polyploidization, which occurred 80-100 Mya. Despite a large scope of the work, the conclusions are ambiguous with respect to the conceptual advance and novelty on polyploid genome evolution. Specific comments are outlined below.

(1) Functional annotation (Figure 1 and Lines 101-107). What are the criteria for the definition of “active promoters” and “active enhancer”? The diagram in Fig. 1B was simulated and did not corelate with biological functions. For example, ATAC-seq peaks should overlap with those of active histone marks such as H3K27ac and H3K4me1, which should be anti-correlated with repressive marks such as H3K27me3. In Fig. 1G, it’s hard to interpret variation of the enhancer (repeat) length and presence/absence. In addition. Each horizontal line should be labeled.

(2) Gene expression across tissues (Figure 2). If the purpose is for comparative analysis between species, it would be better to compare them within each of individual stages of development. The y-axis should be explained. The current maps are less informative.

(3) Ohnology expression divergence (Figure 3). The gene “regulatory” evolution should be changed to gene “expression” evolution in the text and figure. They used both ohnology and orthology in the analysis, which caused conceptual confusion. Please clarify them. In C and D, how were the two sets of ohnologs (red) computed to gain divergence values?

(4) Regulatory elements (Figure 4). This part should come early in the Results to help understand and interpret functional annotation data.

(5) Enhancer evolution (Figure 5). Enhancer sequences tend to diverge more than promoter sequences. As such, it’s better to focus on conserved ohnology/orthologs for this part of analysis. The presence of TEs in these sequences is interesting. In plants, TEs can serve as regulatory function via transcription and RNA-directed DNA methylation (RdDM). The role of TEs

should be discussed since animals don’t have RdDM pathway.

(6) Enhancer-CNEs (Figure 6). Enhancers are DNA sequences that are present or absent regardless of developmental stages and tissue specificity. It’s unclear how enhancers could be present in one developmental stage but absent in other stages? One possibility is that tissue-specific transcription factor(s) could discriminate enhancer function but not the sequence itself. This confusion exists in the entire manuscript and should be clarified.

Minor: citation problems such as in lines 301, 306, 312 (52?), etc., probably from a previous format?

In summary, the data are useful for the polyploid research community, but the description is broad across 1R-4R WGD events, while the most interesting findings were related to the 4R WGD. Perhaps the authors should focus on the conservation and divergence of gene expression and regulatory elements after 4R WGD and clarify the data interpretation and conclusions.

********************END********************

Version 1:

Decision Letter:

24th June 2026

Dear Dan,

Thank you for submitting your revised manuscript "Salmonids reveal principles of regulatory evolution following autotetraploidization" (NATECOLEVOL-26010236A). It has now been seen again by the original reviewers and their comments are below. The reviewers find that the paper has improved in revision, and therefore we'll be happy in principle to publish it in Nature Ecology & Evolution, pending minor revisions to comply with our editorial and formatting guidelines.

Reviewer #1 has checked your responses to Reviewer #3 and is satisfied with your revisions, so there is no need to respond.

We are now performing detailed checks on your paper and will send you a checklist detailing our editorial and formatting requirements in a couple of weeks. Please do not upload the final materials and make any revisions until you receive this additional information from us.

Thank you again for your interest in Nature Ecology & Evolution. Please do not hesitate to contact me if you have any questions.

[redacted]

Reviewer #1 (Remarks to the Author):

The authors have adequately addressed all my previous concerns and I am happy to endorse this manuscript for publication.

Reviewer #2 (Remarks to the Author):

I would like to thank the authors for their detailed responses to my comments on the first version of this manuscript. I found the replies and the modifications very satisfactory and I believe the additional analyses included in this revised version have made a very positive impact on the paper.

I have no further comments and would like to congratulate the authors on this highly impactful and thorough work.

With best regards,
Jukka-Pekka Verta

Reviewer #2 (Remarks on code availability):

The repo and the code are very well documented and organized, no further suggestions.

Reviewer #3 (Remarks to the Author):

The authors have tried to address my comments; however, many of their responses raise additional questions rather than provide satisfactory clarification. Here are a couple of examples.

For example, regarding the functional annotation and the definitions of active promoters and active enhancers, the authors responded with statements such as, “Perhaps the reviewer was not familiar with the approach...” and “Please refer to the Methods section...”. For a paper intended for publication in NEE, general readers would reasonably expect a short and concise definition in the MAIN text. Directing reviewers and readers to the Methods section, and reproducing large portions of that section in the response letter, does not adequately address the concern. I find this approach disappointing.

Another example concerns the use of orthology and ohnology in the same context. The commonly accepted terminology is orthologs (genes diverged by speciation) and paralogs (genes duplicated within a species). The term ohnology is used much more narrowly and is less common. In response, the authors stated, “This comment is so short on details, we found it impossible to understand why the reviewer is conceptually confused,” and “We don’t understand what change would help the reviewer here without them providing more explicit and detailed information.” If the authors consider themselves experts in genome duplication and polyploidy, yet are unable to understand the concern being raised, they should ask whether the presentation in the manuscript itself may be contributing to the confusion.

I shall discontinue the list from their individual responses. They should read the comments and corresponding answers and examine themselves.

In summary, while the authors have made some revisions, many of the substantive concerns raised in the previous review remain insufficiently addressed. I strongly encourage the authors to carefully reread the reviewer comments and provide more direct and constructive responses.

INTERNAL USE ONLY AIP modules required:["Life Science"]

Open Access This Peer Review File is licensed under a Creative Commons Attribution 4.0 International License, which permits use, sharing, adaptation, distribution and reproduction in any medium or format, as long as you give appropriate credit to the original author(s) and the source, provide a link to the Creative Commons license, and indicate if changes were made.
In cases where reviewers are anonymous, credit should be given to 'Anonymous Referee' and the source.
The images or other third party material in this Peer Review File are included in the article’s Creative Commons license, unless indicated otherwise in a credit line to the material. If material is not included in the article’s Creative Commons license and your intended use is not permitted by statutory regulation or exceeds the permitted use, you will need to obtain permission directly from the copyright holder.
To view a copy of this license, visit https://creativecommons.org/licenses/by/4.0/

Response to reviewers

Reviewer #1

This manuscript presents an incredible resource from the AQUA FAANG consortium, including 771 high throughput sequencing libraries (RNA seq, ATAC seq, ChIP seq) across development and adult tissues in two salmonid species. The integrative analysis provides unprecedented insights into the regulatory evolution of ohnologs following the salmonid specific 4R whole genome duplication and the subsequent two wave rediploidization process.

By characterizing the highly dynamic and variable profiles of ohnolog expression divergence, this study found the highest constraint of expression divergence at the late somitogenesis/segmentation stages shared between early and late rediploidzation resolved ohnolog pairs across two species, implicating the same strong purifying selection to constrain the divergence of duplicated genes. Delving into the mechanistic how, the following analyses convincingly link the ohnolog expression divergence pattern with the cis-regulatory control of enhancers, as well as the role TE insertion to evolve new enhancer sequences.

The manuscript is well written given the complexity of the data and analyses. My comments are primarily aimed at increasing clarity, transparency, and reproducibility. They should be straightforward to address.

Reply: We greatly appreciate the time you have spent reviewing our work and are happy to receive your positive appraisal. Thanks for acknowledging the value of the resource, as well as the insights derived from our analyses.

1. Regarding the authors' claim of 'high-quality sequencing datasets', I would recommend more rigorous technical validation, such as adding quality control metrics in Table S3 for the RNA-seq, ATAC-seq, and ChIP-seq libraries. Specifically, this should include mapping rates, duplication rates, and—for the epigenomic datasets—FRiP (Fraction of Reads in Peaks) scores.

Reply: As recommended, Table S3 (Supplementary Table 3) now includes mapping rates and duplication rates for all datasets and FRiP scores the ATAC-seq and ChIP-seq datasets. We also removed the claim of "high quality" from the text, allowing readers to judge data quality for themselves.

For replication conditions, a BAM coverage correlation matrix (Pearson or Spearman) or a heatmap would greatly strengthen the evidence for the reproducibility and technical integrity of their replicates.

Reply: Thanks for this suggestion. We provided BAM coverage PCA analyses for all datasets in Supplementary Figs. 2-4 in the original submission, which were formatted to provide the required information on the grouping of biological replicates. However, extensive quality control metrics, including BAM coverage sample correlation matrices, were generated via MultiQC reports, which spans our complete dataset.

In line with your recommendation, we now share all MultiQC reports as a supplementary dataset. We prefer not to include additional QC plots as supplementary figures, because the supplementary information is already very long and comprehensive. By sharing all MultiQC reports, the requested information is available, alongside other indicators of quality.

This is particularly important for the ChIP seq and ATAC seq data, where signal reproducibility is a ENCODE standard.

We agree signal reproducibility is crucial for these epigenomic datasets. For our core analyses of active promoters and enhancers (used to explore regulatory evolution following WGD), this was

achieved using the irreproducible discovery rate (IDR) with our ATAC-seq datasets (please, also see next reply), capturing signals from the most biologically-reproducible open chromatin regions, following ENCODE best practise. As a general point on ENCODE, while we agree on the value of alignment with this human- and model-oriented initiative, in our view, the standards for equivalent data for non-model fish species should not be dogmatically fixed for a number of valid reasons. Nonetheless, AQUA-FAANG in almost all cases achieved at least 3 biological replicates per epigenomic assay, above the ENCODE recommended minimum.

Fig S5-S9: ATAC and ChIP peaks with genomic annotation were showed for replicated samples per specific sample condition, which showed comparable portions of annotated features. However, certain samples did stand out (e.g. trout_LS_1 in FigS5, salmon_brain_2 in FigS9). Please also include the IDR merged peak number for each set of replicates.

Reply: We are happy to share the IDR merged peak numbers. But first, please note our analyses on active promoters and enhancers follows Baranasic et al. 2022 (Nature Genetics 54: 1037–1050. https://doi.org/10.1038/s41588-022-01089-w), where IDR-passing open chromatin regions (defined by ATAC-seq biological replicates) were functionally annotated using the integration of signals from four ChIP-seq histone marks, modelled in ChromHMM. Consequently, IDR was used for ATAC-seq, but not our ChIP-seq datasets. As requested, we provide IDR merged peak numbers across all possible pairwise comparisons within each set of replicates, including the number and proportion of IDR-passed peaks (see new Supplementary Table 4).

2. The experimental design is largely consistent between Atlantic salmon and rainbow trout, except for the lacking of Gonad ChIP-seq in trout.

Reply: Every effort was made to standardise experimental design between salmon and trout, from sampling, to library generation, to sequencing, to bioinformatics. Below we explain this comprehensively, along with any deviations due to challenges faced during the project.

i) Sample acquisition was standardised across species to the maximal possible extent:

- For embryo stages (DevMaps), both species were sampled using identical methods and staging criteria, at the same institute, by the same co-author.

- For adult tissues (BodyMaps), the two species were sampled at different institutes (NMBU, salmon; INRAe, trout) by different co-authors, using identical methods outlined in the methods/protocols. This species division was a product of the split in funding across partners in the AQUA-FAANG consortium and hence unavoidable.

ii) For RNA-seq, our approach was fully standardised, with matched samples for each species processed for library prep and sequencing using the same service provider and sequencing platform.

iii) For ATAC- and ChIP-seq library preps and sequencing:

- Our approach was fully standardised across species for embryo stages (DevMap). Library prep was performed by the same co-authors using identical protocols.

- For adult tissues (BodyMap), library preparations were done using standardised protocols described in the methods/protocols, but at different institutes (NMBU, salmon; INRAe, trout). NMBU was the lead on developing these protocols, and had more available staff resources. For trout, we faced challenges that led to a failure to produce high quality ChIP-seq data for gonads. Also, the quality of ATAC- and ChIP-seq data for muscle was of lower quality in trout, due to challenges faced with obtaining sufficient high-quality nuclei. A lack of person resources and funding meant it became impossible to overcome these challenges. However, the impacts were

limited and do not dilute the overall achievements of the project, nor the quality of novel insights on genome regulatory evolution made possible using the available 771 datasets.

iv) Sequencing was standardised for ATAC-seq and ChIP-seq libraries in both species, as was done by a single service provider on the same platform.

v) Bioinformatic analyses were standardised across species using identical code, which has been openly shared.

Could this ["lacking of Gonad ChIP-seq in trout"] explain the higher number of open chromatin regions and the higher CAGE support rate observed in salmon?

Reply: The higher number of open chromatin regions in salmon cannot be explained by the absence of gonad ChIP-seq data, as the open chromatin regions are based on ATAC-seq data, which was available for all tissues in trout. However, as noted above, the quality of ATAC-seq data in trout muscle, judged by the number of peaks across samples (Figure S5 in the original submission), was lower than salmon muscle. This likely contributed to the differences observed.

Another potential contributor is genome size. The Atlantic salmon genome (~3.1Gb) is approximately 25% larger than the rainbow trout genome (~2.3Gb). These differences are driven by variation in repeat content. In light of the association between salmon TEs and active enhancers among our "Exclusive" active enhancer category, which by definition are open chromatin regions, the additional genome size in salmon could be a factor.

Because CAGE was restricted to DevMap samples, the lack of gonad ChIP-seq in trout has no impact on the CAGE support rate. Given that the methods used to obtain ATAC-seq and ChIP-seq data for DevMap analyses were identical in both species (done by the same co-authors, at the same location, with the same protocols), we cannot otherwise readily explain species differences in overlap between CAGE and promoter annotations.

Could these differences affect downstream analyses between species? Actually later analyses involving ohnolog pairs and cis-regulatory elements were mainly on Salmon (Fig 3-5), without equivalent data in trout.

Reply: Importantly, these differences across salmon and trout have no impact on key conclusions presented on regulatory element evolution after WGD (Figs. 4-6) for the reason identified by the reviewer. We deliberately focus those inferences on salmon, because this species captures the broadest panel of high-quality regulatory data from ATAC- and ChIP-seq. Cross-species comparisons of ATAC-seq data are presented in Fig. 2b (and Extended Data Fig. 1), which shows that open chromatin regions from similar SOM clusters are enriched in syntenic regions (between salmon and trout) defined using genome alignments. We also provide cross-species comparisons of transcription factor binding motifs in active promoter and enhancer elements, but these are limited to sample types with data available in both species, and were provided to evidence the overall quality of our regulatory element atlases. In Fig. 3, trout RNA-seq data was however used for comparisons between ohnolog and ortholog transcriptome evolution.

3. The gene and regulatory element datasets defined are central to the analyses of duplicated gene regulation evolution: A total 10,521 high-confidence ohnolog pairs conserved in both species and 5,832 'singletons', and 26,157 genes and 194,528 promoters or enhancers. Are those the same set used for the DevMap and BodyMap analyses in the result section "Dynamic genome regulation across ontogeny and tissues"? If so, does that mean at least one third of genes in each genome were excluded? Please clarify and discuss any potential biases would be introduced due to limited representativeness.

Reply: The manuscript section "*Dynamic genome regulation across ontogeny and tissues*" is based on the global RNA-seq and ATAC-seq datasets, not the comparative dataset presented in the preceding section. Therefore, all genes and reproducible (robust) open chromatin regions were considered and no data was excluded, meaning no biases were introduced.

We realise now that presenting our comparative dataset of genes/regulatory elements immediately before a section using the global dataset led to the reviewer's question. To clarify the text, we made some changes to explain that the results described in the section "*Dynamic genome regulation across ontogeny and tissues*", are based on global datasets, not the subset used for comparative analysis, which are now clearly flagged to form the basis for downstream evolutionary inferences.

4. While SOM is an effective tool for visualizing high-dimensional expression and chromatin accessibility patterns, the manuscript would benefit from a more detailed justification of the parameters used and quality control.

Reply: As recommended, we expanded the methods to provide a more detailed justification of the parameters used. We summarise this information below and revised the Methods accordingly.

Regarding grid dimensions, these were selected to exceed the number of distinct experimental conditions in each dataset, ensuring sufficient resolution to capture dynamic expression or accessibility profiles. A 4×4 grid (16 clusters) was used for DevMap RNA-seq (14 developmental stages), a 6×6 grid (36 clusters) for BodyMap RNA-seq (~20 tissue/sex/maturation combinations), and a 5×5 grid (25 clusters) for both ATAC-seq datasets. Alternative configurations were systematically evaluated by inspecting cluster profile redundancy and population sizes, following standard practice (Wehrens and Kruisselbrink, 2018. Journal of Statistical Software, 87, 1-18). SOM clustering is an exploratory dimensionality reduction technique, where grid size selection involves informed heuristic choices rather than a single optimisation criterion. This is analogous to the choice of cluster number in k-means or resolution parameter in Leiden clustering.

To expand on the criteria used to define clusters, we formalised a classification between constitutive and tissue-specific SOM clusters through explicit quantitative thresholds applied to each cluster's profile. Clusters were classified as constitutive when the standard deviation of mean values across all conditions was below 0.4, indicating a flat expression profile. For BodyMap datasets, non-constitutive clusters were further classified as tissue-specific when the highest per-condition mean normalized values exceeded 2.165, with the remaining non-constitutive clusters classified as multi-tissue. We acknowledge these thresholds are inherently empirical: they were determined by visual inspection of cluster expression profiles and iteratively adjusted until the resulting classifications were consistent with the observed patterns (e.g., the tissue-specific threshold was selected as the lowest value that did not misclassify any clear multi-tissue cluster). While the choice of threshold values requires an initial manual assessment of the data, formalising the classification in this way limits subjective bias in the assignment of individual clusters, automated the process for reproducibility, and made the criteria transparent and auditable. These thresholds were supplemented by manual curation for a small number of edge cases (e.g., clusters with intermediate profiles). All criteria, threshold values and manual adjustments are documented in the shared analysis code.

5. About cross species SOM comparison, Figure 2 is rich in information but quite difficult to read, particularly the Sankey style diagrams linking salmon and trout SOM clusters. I was confused about how the Sankey Diagrams correspond to heatmap rows (not really I suppose) and how the flow colors correspond to orthology.

Reply: Thank you for these helpful suggestions to improve Fig. 2. We have addressed all points raised and revised the figure accordingly.

I recommend splitting the heatmap and the Sankey/band plots into separate panels with clear labels.

As recommended, we split the heatmaps and Sankey/band plots into separate panels with clear labels. Revised Fig. 2 comprises four panels: (a) RNA-seq SOM heatmaps with UMAP plots, (b) ATAC-seq SOM heatmaps with UMAP plots, (c) RNA-seq cross-species Sankey diagram, and (d) ATAC-seq cross-species Sankey diagram. This improves readability and makes the correspondence between cluster labels and Sankey flows much clearer.

The color legend for the orthologous gene ratio (light red bands) and syntenic region counts (brown bands) should be placed directly adjacent to the corresponding plots.

Reply: Regarding the colour legends for the observed-to-expected orthologous gene ratio (panel c) and the syntenic open chromatin region counts (panel d), they are now placed directly adjacent to their corresponding plots, as recommended.

Regarding the UMAP plots: the caption states that "dots represent mean normalised TPM values of individual genes (A) or mean normalised ATAC seq read intensity values for individual robust open chromatin regions (B)". This phrasing is ambiguous. I assume the UMAP embeddings are calculated from the normalized expression/accessibility profiles across stages, and each dot corresponds to a gene or a chromatin region. Please rephrase to clarify that the UMAP visualizes the multivariate profile of each feature, not a single mean value.

Reply: We have clarified the UMAP description in the legend. The revised text states that UMAP embeddings were calculated from multivariate normalised expression (RNA-seq) or accessibility (ATAC-seq) profiles across all embryonic stages, and that each point represents an individual gene or open chromatin region positioned by to its profile similarity to other features. This replaces the ambiguous phrasing flagged, which wrongly implied that a single summary value was plotted.

Finally, because an equivalent figure and results are presented for our BodyMap atlases, we also updated the equivalent figure/legend (Extended Data Fig. 4 in revised submission; previously Fig. S25).

6. The division of ohnolog pairs into "Early" and "Late" rediploidization classes is central to the study. It would be very informative to know whether these two classes are enriched in specific SOM clusters from the DevMap or BodyMap analyses. E.g., are Early resolved ohnologs overrepresented in clusters associated with later development or specific adult tissues?

Reply: Thanks for this great suggestion to better connect our SOM-based expression clustering to the rediploidization history of ohnolog pairs. We addressed this comment by performing comprehensive new analyses, with results provided in Extended Data Figs. 5 (DevMap) and 6 (BodyMap), along with expansions to the revised results and methods sections. All data analysis code is shared in our GitHub.

- Can the authors trace how individual ohnolog pairs have partitioned into different SOM clusters (e.g., one copy in cluster A, the other in cluster B) and whether this partitioning differs between Early and Late rediploidization pairs? Such an analysis would directly link the rediploidization timeline to regulatory diversification.

Reply: Yes, and we have done so in the revision, as described above. The results reinforce the key messages highlighted in this section of the manuscript, and indeed strengthen our findings connecting rediploidization age to regulatory divergence. We have tried to keep the description and interpretation of the findings concise, and aligned with prior conclusions. It would have been possible to present a much deeper analysis, but we believe this falls outside the scope of this study, where it is difficult to warrant major expansions in text. Such a deeper investigation would be better presented elsewhere.

7. Lines 307–309 and Fig. 5F show that "Exclusive" enhancers overlap TEs to a much greater extent than "Shared" enhancers. One potential confounding factor is that exclusive enhancers might be located farther from transcription start sites on average, and TE content is known to increase with distance from genes. To strengthen the claim that TE insertions actively contribute to enhancer innovation/divergence (rather than merely reflecting a lack of constraint in distal regions), I suggest repeating the TE overlap analysis after binning enhancers by distance to the nearest TSS (e.g., 0–10 kb, 10–50 kb, >50 kb). This would control for the genomic context and provide a more rigorous test.

Reply: We greatly appreciate the suggestion, and agree that testing enhancer distance to TSS is useful to distinguish TE-driven enhancer functional divergence from reduced constraint resulting from a greater physical distance from genes. We therefore repeated the TE-enhancer-overlap analysis stratified by distance to the nearest TSS (bins of 0-2, 2-20, 20-50, 50-100 and 100-1000 Kb), separately for Exclusive, Alignable only and Shared enhancers, and present the results as a new panel within Supplementary Fig. 34. We identified significantly elevated TE content in Exclusive enhancers of diverged (compared to conserved) ohnologs in the 2-20, 20-50, and 50-100 Kb bins, with the strongest effects at the 2-20 and 20-50 Kb bins. For more distal enhancers (>100 Kb from TSS), TE content was not different between diverged and conserved ohnologs. We also found that the increased TE content in Exclusive compared to Shared enhancers was present across all tested TSS distance bins. These results are the opposite of the pattern expected if the signals were driven by Exclusive enhancers being more distal and less selectively constrained. This more rigorous test has been incorporated into the revised results, strengthening our interpretations.

Remarks on code availability:

I confirm that the repository is live and contains the scripts and workflows used for the major analyses presented.

Reply: Thank you for your diligence in checking our code. We confirm at the point of submitting the revision, all code used for the major analyses presented have been incorporated.

Reviewer #2

Baudement and colleagues report a large and impressive collaborative dataset of gene expression and gene regulatory regions in different developmental stages and tissues in two salmonid species. They use this vast dataset to understand regulatory element evolution across genome regions experiencing different rediploidization histories following whole genome duplication. The dataset could (will) be used to address many other questions of gene regulatory evolution and I think the paper's focus on rediploidization and ohnolog evolution is justified and original. This is the most impressive dataset to address these questions I have yet seen. The vast bioinformatic analyses are nearly immaculate and a massive undertaking that has produced results of high uniqueness and value for the wider research community. The fact that results, data, and protocols are shared in extensive detail is exemplary and will be highly appreciated by researchers not only studying salmonids, but also other vertebrates.

Reply: Thank you for your positive appraisal and the time spent reviewing our work. We are glad you found our datasets impressive and saw the novelty of our work. We were most happy to receive your praise for the efforts made to share our work as a resource for the community.

The vast scale of data and results also poses challenges in their presentation. Although I agree with the focus on regulatory evolution following WGD, there are some loose ends in the interpretation of results from analyses of ohnolog expression divergence, which I point out

below. I also think that all sections need to be revised for consistency of motivation (of the study) and use of language, which is quite variable. In a large collaboration like this I think this is the responsibility of the last authors.

Reply: Thank you for your detailed suggestions. We indeed went to efforts to standardise the writing style in the original submission, which was coordinated by the last author. However, we accept there is an inherent challenge in this type of large-scale collaborative effort, and welcome your constructive suggestions, which have helped us strengthen the clarity and logic of the manuscript's presentation.

1. Embryonic development follows very different rates in Atlantic salmon and rainbow trout following from their autumn vs spring spawning life cycle. How the developmental rate is taken into account in the analyses needs to be better explained and put this into perspective when discussing the results. For example, is the regulatory architecture of stage-specific expression and regulatory activity conserved (and therefore simply accelerated in trout compared to salmon)? Can you identify architecture elements (e.g. gene modules, ohnologs, SOMs, regulatory elements) that are responding to developmental time (rate) rather than stage?

Reply: Thanks for sharing these interesting thoughts, which while interesting, are challenging to address using our study design. Embryonic developmental rate in salmonids is of course driven by temperature regime during embryogenesis, which is a product of environmental conditions around spawning time in wild fish. While there exist differences in spawning time between Atlantic salmon and rainbow trout, embryonic temperature was closely matched for both species (see Methods). Moreover, the trout strain we used was from Troutlodge, representing a Steelhead population, which can naturally spawn in the late winter (and indeed, this was the case for our study population, which was spawned in winter). We found that the two species developed at similar rates in our study, reaching the eyed stage in a similar number of days. Furthermore, the populations used are domesticated, not wild, so have been reared at controlled temperatures for many generations, detached from selection occurring in the wild. These factors combine to make it challenging to meaningfully compare gene expression or genomic regulation in the context of developmental rate, as our design minimises such effects across species. The overwhelming across-species similarity in gene expression across 14 sampled matched stages of embryogenesis (Fig. 2-b) suggests our strategy was successful. We consequently don't feel changes in the manuscript are required.

2. Lines 66-67: I suggest making this motivation statement more specific. How does knowing the functional outcomes of rediploidizations help to understand species radiations?

Reply: Thanks for highlighting this issue. On reflection we agree the original statement lacks clarity. For reference, the original sentence (alongside its proceeding sentence for context) were as follows:

"*Understanding the functional outcomes of rediploidization is necessary to unravel the genomic basis for major vertebrate radiations. However, studies of regulatory changes following the 1R-3R WGDs are challenged by the vast evolutionary time separating these events from living species*."

In the revised manuscript, we decided to delete the text entirely, now merging two paragraphs, and shortening the introduction. This serves to remove ambiguous links between the functional outcomes of rediploidisation and species radiations (which would demand additional text to explain; clearly unwarranted), without losing any key rationale for our study. The impact of this change is minimised by the fact that we return to the broader relevance of our findings for understanding vertebrate genetic evolution in the Discussion.

3. Lines 77-78: I understand this argument of having one "genetic background" to look at the functional divergence of ohnologs in different stages of rediploidization - but could you articulate here how do you take into account that rediploidization of some genes may influence further selection ("evolutionary degrees of freedom") of other genes. Can we claim that

evolution of some ohnologs is independent of evolutionary endpoints in other ohnologs? My concern here is that if ohnolog evolution is interdependent on evolution of other ohnologs, evolutionary trends in, say, late rediploidization ohnologs becomes dependent on rediploidization of early rediploidization ohnologs, and the "stratification of ohnolog divergence times" is not independent of which ohnologs reverted to diploidy first. In this case you would not be looking at different evolutionary "strata" but an interdependent trajectory, which are not the same.

Reply: For context, the sentence flagged was: "*Asynchronous rediploidization creates a stratification of ohnolog divergence times within affected genomes, providing an ideal study system to explore the function and regulation of ohnologs at distinct stages of evolution.*".

The points you raise are relevant for attempts to fully dissect the intricacies of ohnolog evolution in a genome showing asynchronous rediploidization. But your stated concern on the non-independence of ohnolog evolutionary trajectories, while important and unresolved, is rather detached from the simple purpose of the original statement, and sits beyond the scope of this study. The statement in the introduction stands as correct, irrespective of the selective pressures governing the co-evolution of ohnologs across the genome, mainly because it is foundationally a product of the absolute timing of rediploidisation, which varies across the genome (Gundappa et al. 2022. Mol. Biol. Evol. 39: msab310. https://doi.org/10.1093/molbev/msab310). This is shown in Fig. 4b, where the percentage divergence between large genomic regions retained from the salmonid WGD is shown, highlighting the variation (stratum) of ohnolog sequence divergence in the genome. The original statement does not attempt to make claims about non-independence of ohnolog co-evolution with respect to rediploidisation timing. It simply reflects that the transition from tetrasomic inheritance to two unlinked disomic ohnolog pairs occurred at different times in different genomic locations, leading to a "strata" of divergence times. We do not feel any changes are required in the manuscript, and we stand by the accuracy of the original statement.

As a related thought, your questions on ohnolog co-evolutionary trajectories, how this relates to rediploidisation timing, and the role of selection vs. drift in this context, represent an area of active research in the ongoing WGDip project (https://www.rediploidisation.org/), which is using salmonids as a study system, alongside other WGD events followed by asynchronous rediploidization. We consider this as one of the frontiers in the field and have posed similar questions ourselves in a forward-looking perspective article that is currently under review in Nature Reviews Genetics.

4. Section entitled "Ontogeny governs the evolution of enhancer-CNEs following WDG": While I find the results of this section interesting in describing general tendencies of enhancer-CNE conservation across phylogenies and rediploidization states, many of the results here describe general statistics without much biological interpretation. Could you not do a sequence motif analysis for the CNEs? This could potentially bring a lot of biological insight into what signaling pathways are conserved across WGD/phylogeny/ontogeny and what pathways are evolvable.

Reply: Thanks for this great suggestion. We originally didn't include such analyses on the grounds of the existing length of the manuscript. To address your suggestion, we have incorporated comprehensive new motif enrichment analyses for enhancer-CNEs of different phylogenetic ages from the Shared and Exclusive categories. The results are very interesting, and have been incorporated into the Results, Methods, Discussion, as well as in an Extended Data Figure, Supplementary Figure, Supplementary Text, and two new Supplementary tables.

5. Section entitled "Enhancer evolution is coupled to ohnolog expression regulation": This section is clearly in need of rephrasing and clarification of sentences. It is hard to follow what are the exact results and what is being compared. The style in this section is also markedly different from the rest of the manuscript and I recommend editing for consistency by the last

authors (e.g. word use, post-hoc justifications of analyses, etc.). The results here are also reported in a more list-like manner and I recommend balancing this section with more interpretation of the results.

Reply: Thanks for highlighting that you found this section difficult to follow, and in a distinct style to other parts of the manuscript. We have made a significant effort to rephrase and clarify according to your specific suggestion, adding more interpretation of the results, while editing for greater consistency with other sections.

Lines 249-250: The motivation for this analysis is weak and unspecific – please elaborate in more specific terms what new knowledge of the evolutionary process this analysis creates.

Reply: For context, the original statement (underlined) and its wrapping sentences: "*Cis-regulatory elements govern transcriptional regulation* [47,49–51]*, and in mammals, enhancer evolution is linked to rates of gene expression evolution* [46]*. <u>However, the association between enhancer evolution and ohnolog expression divergence following WGD remains poorly understood.</u> We addressed this knowledge gap, using analyses that did not split genes by rediploidization history to exploit the stratification of ohnolog sequence divergence when making inferences on regulatory changes*".

We have expanded and strengthened the motivation for this analysis, posing outstanding scientific questions and explaining how the work that follows extends beyond past analyses:

"Enhancers shape transcriptional regulation [52,54–56] *and their conservation is strongly linked to gene expression evolution across species* [51]*. Does the loss, retention, or gain of enhancer activity similarly explain changes in ohnolog expression evolution following WGD? While a past study proposed that enhancers are involved in ohnolog expression divergence following the salmonid 4R WGD* [57]*, its conclusions were based on a single histone mark and one tissue (testis; showing rapid regulatory evolution in this study), while the retention of enhancer sequences from WGD was not assessed. We used our broad sample panel, regulatory atlases, and whole genome alignment to comprehensively dissect how enhancer activity and sequence conservation shaped ohnolog expression evolution, combining complementary approaches that exploit the full range of ohnolog divergence across the salmon genome"*.

Lines 250-252: I suggest rephrasing this sentence because it is not immediately clear what is the message here.

Reply: The original sentence was "*We addressed this knowledge gap, using analyses that did not split genes by rediploidization history to exploit the stratification of ohnolog sequence divergence when making inferences on regulatory changes*."

We rephrased the sentence as presented above. The point we sought to convey is that we didn't deliberately separate Early and Late rediploidization ohnologs for this analysis, rather we used the full spectrum of variation in ohnolog expression and sequence divergence to give most power to inform on the relationship between enhancer activity/conservation and ohnolog expression evolution.

Lines 264-274: This paragraph is more wordy than other paragraphs and the message could be condensed here without elaborate justification of the analyses.

Reply: We have condensed the message markedly in the revised text.

Lines 265-268: How do you take into account total expression level in this analysis? My concern here is that lower expression correlation may be driven by lower expression, so can these classes simply reflect groups of genes with higher or lower expression instead of variable or conserved regulation?

Reply: This point is the same as your next comment (covered in next reply).

Lines 275-281: How do you take into account the effect of expression noise in this analysis? My concern here is that if you define the regulatory classes based on expression correlation, genes with lower correlation may be those with lower overall expression - in that case finding more active enhancers in "conserved regulation" class may simply reflect the higher expression of genes in that class.

Reply: We agree it's important to take expression level into consideration in this analysis. We have added in a new analysis to show that ohnolog pairs from the two defined expression classes do not show clear differences in expression level. Please see Extended Data Fig. 7c in the revised submission. The result is briefly mentioned in the Results section.

Lines 284-288: It is not clear how these two analyses (questions) are different, please rephrase.

Reply: For context, the original sentence was "*Ohnolog pairs showing divergent expression had higher enhancer-usage distances than pairs showing conserved expression (Fig. 5D; respective medians = 1.15 vs 0.89; $p = 1.6 \times 10^{-28}$). We further asked if enhancer usage distance is associated with ohnolog pair expression distance (see Methods; Fig. 5E, Fig. S38).*"

In the revised submission, we have rephrased as recommended, emphasising the distinction between the two analyses by splitting the paragraph and explaining the second step now compares enhancer-usage distance against expression distance on a continuous scale, as opposed to the categorical analysis previous using "conserved" and "diverged" ohnolog sets.

Lines 288-289: Please quantify these statements of "decreased" and "more variable" in some way.

Reply: Done. We backed up these statements with the addition of Kruskal-Wallis tests.

Lines 291-293: Please state stronger than *what*.

Reply: We have rephrased this text, replacing 'stronger' with 'significant' based on Kruskal-Wallis tests, and making it clear we're comparing differences between tissues.

Lines 293-295: It is not clear what is being compared here.

Reply: For context, the original sentence was "*Brain showed the lowest distance and dispersion in both cases, whereas liver, ovary, and testis displayed the broadest range of values*". We have rephrased this section to make the intended comparison explicit.

Lines 296-297: This is not a strong conclusion and it's self-evident that regulatory elements will evolve across time - this conclusion is simply saying regulatory elements evolve, it tells very little *how* regulatory element evolution influences ohnolog expression evolution. Please elaborate.

Reply: Thank you for flagging your concern. For context, the original sentence was "*Overall, these results reveal that the evolution of ohnolog expression is associated with the loss of ancestral enhancer sequences or the gain of new enhancer sequences post-WGD*."

We have elaborated the conclusion as recommended, more explicitly referencing our findings:

"Taken together, these analyses reveal an overall coupling between enhancer evolution and ohnolog expression divergence following the salmonid 4R WGD, with clear differences between embryogenesis and adult tissues. Ohnolog expression divergence strongly tracks the loss of Shared enhancers and gain of Exclusive enhancers. Moreover, the overall relationship between enhancer evolution and ohnolog expression is consistent with the ontogenetic and tissue-specific constraints shown earlier (Figs. 3, 4)."

6. Lines 397-399: In this context it would be helpful if you could succinctly explain how this perspective is novel from previous knowledge.

Reply: Thank you for this suggestion. For context, the original statement, which opened the discussion section, was: "*This study paints a novel perspective of genome evolution following autotetraploidization, where ohnolog regulatory evolution is governed by the same ontogenetic and tissue-specific constraints acting between species*".

As recommended, we revised the opening paragraph of the discussion considerably, in an effort to more specifically highlight the novelty of our findings in relation to the most relevant past knowledge. In doing so, we have focussed the paragraph more strongly on the salmonid 4R WGD:

"Our findings reveal that ohnolog regulatory evolution following the salmonid 4R WGD was strongly shaped by ontogenetic and tissue-specific constraints shown elsewhere to be acting across vertebrate species [42,45,47]. Multiple lines of evidence imply a period of high 'within-genome' i.e. post-WGD constraint on regulatory evolution at late embryogenesis, which likely reflects the complex pleiotropic regulatory networks governing organogenesis at this life stage in all vertebrates [42]. A past study in salmon profiled the active histone mark H3K27ac using testis samples, concluding that the evolution of regulatory elements following the 4R WGD was driven by neutral processes [57]. While consistent with our findings for testis, broader profiling of sample types revealed extensive variation in the rate of regulatory element evolution across tissue types, with brain representing the maxima of post-WGD constraint, and gonads and liver experiencing rapid regulatory evolution, which may reflect either/both relaxed purifying selection and positive selection. Our study also provides complementary evidence that the 4R WGD created opportunities for regulatory diversification, via loss of duplicated enhancer-CNEs created during rediploidization. Future studies should further dissect these findings, and would benefit from additional data types to penetrate regulation at the level of 3D chromatin architecture and specific cell types [63]."

Please note, in the last paragraph of the discussion, we also added a new citation to a tissue regulatory atlas published elsewhere in rainbow trout, noting its value as a resource to the research community, alongside the datasets shared in our work.

Additional comments:

7. Line 101: Please briefly explain what is "robust" in this context.

Reply: We revised the sentence to explicitly explain the meaning of robust in this context.

"*We identified 319,718 'robust' open chromatin regions in salmon and 194,002 in trout, defined as strongly supported ATAC-seq peaks using the irreproducible discovery rate (IDR) approach [36] with our biological replicates (Supplementary Table 4). These regions are expected to harbour regulatory elements bound by transcription factors (TFs) and protein complexes associated with RNA polymerase II [33].*"

Please note, additional information on the IDR peaks used to define these open chromatin regions has been provided at the request of Reviewer 1.

8. Line 129: "Closely related" is a bit misleading in this context because you have not analyzed associated changes in SOM cluster genes and e.g. their proximate ATAC-seq peaks. I think "similar" would be more appropriate here.

Reply: A fair point. We revised the sentence as suggested.

"Across embryogenesis, self-organizing maps (SOM) identified clusters of genes (Fig. 2a; Supplementary Figs. 15-16) or genomic regions (Fig. 2b; Supplementary Figs. 17-18) showing similar stage-specific changes in expression or chromatin openness."

9. Lines 162-163: Could you please elaborate briefly on the reasons/logic on why ohnolog expression divergence decreases across embryogenesis. How does this reflect on some more general observation?

Reply: We thank the reviewer for this suggestion. We have added text to this part of the results section, connecting the observed pattern to the vertebrate phylotypic period:

"*Ohnolog expression divergence decreased across embryogenesis, being highest at the earliest cleavage stage (pre-MZT), and lowest at the most advanced segmentation and pharyngula stages (Fig. 3a), thought to represent the vertebrate phylotypic period* [45]*, when transcriptomic profiles in different species show the highest level of conservation across ontogeny* [42,45,47]*. Our data therefore support that post-WGD regulatory divergence is subject to the same developmental constraints that shape gene expression evolution between species*."

10. Line 176: Can you please elaborate briefly why do you think the difference in divergence between early and late rediploidization genes is smallest in ovaries and markedly different from other tissues.

Reply: The original statement was: "*Unlike embryogenesis, expression divergence across tissues was not significantly correlated between Early and Late rediploidization ohnolog pairs (Fig. S33). This was explained by increased expression divergence in ovaries, alongside reduced divergence in muscle and liver, for the Late rediploidization ohnolog pairs*".

The result can be explained by an unusually elevated rate of regulatory evolution for ovary-expressed ohnolog pairs, specifically for late rediploidisation regions, when compared to other tissues. This is consistent with the least strong purifying selection to maintain dosage of the youngest ovary ohnolog pairs, allowing these gene pairs to diverge more freely in expression level compared to Late rediploidizing ohnologs expressed in the other profiled tissues. *Why* this might be the case remains the subject of speculation, but it is something we are exploring in ongoing work aiming to dissect the role of the post-WGD evolutionary constraint on gonads. We would prefer not to expand the manuscript with too much speculation, but have revised the statement to point towards differences in the extent of purifying selection to explain the observation:

"Unlike embryogenesis, expression divergence across tissues was not significantly correlated between Early and Late rediploidization ohnolog pairs (Supplementary Fig. 29). This was explained by increased expression divergence in ovaries alongside reduced divergence in muscle for the Late rediploidization pairs, indicative of the weakest and strongest levels of purifying selection on the dosage of young ohnolog pairs, respectively, among the profiled tissues."

11. Lines 193-194: Can you please briefly elaborate what does this mean in terms of gene (regulatory) evolution in general. Why is expression evolution more constrained in ohnologs vs singletons during later stages of embryogenesis? What does this tell us about the functional roles of (genes retained as) ohnologs vs singletons in development?

Reply: The original statement was: "*This result indicates that orthologs evolving under the highest constraints on expression during mid-to-late embryogenesis are overrepresented as ohnolog pairs*."

We are happy to elaborate on the broader relevance of the observation. As ohnologs are well-established (including after the salmonid 4R) to be highly enriched in dosage-sensitive roles linked to development (e.g., transcriptional regulation), our results suggest that maintaining the ancestral

dosage of genes expressed during mid-to-late embryogenesis is an important selective pressure for ohnolog retention and expression evolution.

We revised the text to expand on this argument, including citations to relevant literature:

"*As observed for many WGD events, genes retained as ohnolog pairs following the salmonid 4R WGD are enriched for dosage sensitive functions* [49,50] *such as development, transcriptional regulation and signalling, while singletons are weakly enriched for metabolic roles* [20]*. Taken alongside the strong correlation between expression divergence observed for ohnolog pairs (post-WGD) vs. orthologs of the same genes (post-speciation), our results suggest that genes expressed during mid-to-late segmentation are not only generally dosage sensitive, but that this property selects for retention as duplicates following WGD*."

12. Lines 197-198: Why is this pattern conserved across these main osmoregulatory organs? Is it driven by a specific set of transcripts that have conserved roles (e.g. in osmoregulation)?

Reply: The original statement was "*For gill, head kidney and distal intestine ortholog pairs from the ohnolog group showed significantly less expression divergence than singletons (Fig. 3D).*".

In short, this question is challenging to answer without extensive additional analysis, which lies outside the scope of this study. While the general idea that the expression of the same genes could contribute to similar patterns of ohnolog or singleton expression across tissues is sound, the picture is complicated. Firstly, just because the orthologs that represent ohnologs showed less expression divergence than singletons, the absolute JSD values are not the same when comparing each group of genes across the three mentioned tissues - this makes it likely that different genes are contributing. Secondly, each of these organs has multiple functions and a mixture of both shared and unique cell types with roles extending far beyond osmoregulation, including innate and adaptive immunity. Consequently, linking the evolution of genes to specific functions is challenging, minimally requiring single cell RNA-seq to help separate the sub-functions of cell types comprising the bulk tissue samples used in this work. Thus, the identified link with osmoregulation may be coincidental, or only part of a more complex picture.

13. Lines 204-205: Could you elaborate on this conclusion/argument in a more explicit manner. For example, what is meant with "pleiotropic constraints" in this context?

Reply: The original sentence was: "*In sum, these results reveal the nature of post-WGD regulatory evolution across ontogeny and tissues, implicating pleiotropic constraints during a period of rapid organ development at mid-to-late embryogenesis as one strong factor selecting for the long-term retention of ohnologs, while also highlighting ohnolog retention as a driver of tissue regulatory evolution both within and across species, but with highly variable effects across tissue types*"

We have expanded and rephrased this wrap-up statement in an effort to make the text clearer and more explicit, while explaining what was meant by "pleiotropic constraints" in appropriate context:

"*Overall, these results reveal that the 4R WGD has played a central role in regulatory evolution in salmonids, shaping variation in the extent of gene expression divergence across ontogeny and tissue types, both within the same genome for ohnolog pairs, but also for different classes of orthologs following speciation. In both contexts, a period of maximal constraint on regulatory evolution is inferred during the period spanning the late stages of embryogenesis. During this period, pleiotropic genes are intensively recruited across multiple developing cell and organ types, creating a highly interconnected regulatory environment that is proposed to limit evolvability across species* [42]*. Our results suggest the same pleiotropic constraints act on duplicated gene networks retained following 4R autopolyploidisation and likely represent a critical driver for ohnolog retention and expression evolution".*

14. Line 242: Please specify female or male gonads (or both).

Reply: Updated to clarify: "*Mature testis showed the highest proportion of Exclusive enhancers, while liver, ovary and testis showed the lowest proportion of Shared enhancers (Fig. 4d).*"

15. Lines 302-303: It is not clear what is meant with "constraints" in this context.

Reply: For context, the original statement was "*On the other hand, TEs also disable functional elements within genomes, meaning their overlap with regulatory elements provides insights into constraints on gene regulation across ontogeny or tissues*"

We have rephrased this sentence effort to clarify the intended meaning. Please see the change in the context of our response to your next comment.

16. Lines 299-309: I'd like to point out that all but two sentences in this paragraph start with a subordinate clause (indeed… on the other hand… yet… to explore these questions… overall…). My suggestion is to use more direct sentences and to minimize the use of such style.

Reply: While we strongly value your opinion, the original style was clearly a matter of personal preference - and we felt it added impact in the context of the paragraph in question.

As a compromise, we reworked the text to remove two of the subordinate clauses in the paragraph:

"*How TEs contribute to enhancer functional evolution following WGD remains poorly understood. One mechanism to evolve new enhancer sequences is through transposable element (TE) activity, as observed following the salmonid 4R WGD [59]. However, TEs also disable functional regions within genomes, meaning their overlap with pre-existing regulatory elements also reflects the strength of purifying selection acting on regulatory functions. To explore these questions, we identified all active enhancers within cis-regulatory domains from salmon that overlapped with a high-quality TE annotation [59]. We found that Exclusive enhancers overlapped TEs to a much larger extent (>2 fold) than Shared enhancers (Fig. 5f), which could be explained by TE insertions either acting to create de novo enhancers [57,59], or by reduced constraint on the functions of Exclusive enhancers.*"

17. Line 339: "Influences" indicates a causal relationship so I think "associates" would be more appropriate here.

Reply: In the context of the sentence, the indication of a causal relationship is intended, so we prefer to keep the original phrasing.

18. Line 367: I think "reflect" is more suiting than "shape" because you have not argued/showed whether the causality goes from sequence conservation -> regulatory activity or vice versa.

Reply: We have changed to "reflects", as recommended.

19. Line 403: Is the inference of "selection" really justified here? You have not measured selection (in this case it seems to be implied as relaxed purifying selection), and increased variation could in fact be beneficial and promoted by natural selection.

Reply: We agree that the increased regulatory variation observed in these tissues could indeed be promoted by selection, and not just a product of relaxed purifying selection. The statement has been rephrased and made more explicit during the revision to the relevant Discussion paragraph (explained in our reply to major comment 6).

20. Line 413: I would rephrase "ideal" here - ideal means that there is no better model system, and many arguments could be made pointing to the contrary.

Reply: We disagree that the original statement (i.e. "an ideal vertebrate system") implied there is no better model system. We don't feel strongly on the phrasing, so replaced "ideal" with "excellent".

Reviewer #3 (polyploidy, gene regulation):

The authors reported results from analysis of multiple sets of genomic, chromatin, and gene expression data and tested evolutionary consequences of tetraploidization in salmonids. They found the changes were largely dependent on developmental stages and tissue-specificity. Salmonids are among vertebrate species for the study of polyploidization, which occurred 80-100 Mya. Despite a large scope of the work, the conclusions are ambiguous with respect to the conceptual advance and novelty on polyploid genome evolution.

Reply: Thank you for taking the time to review our work, and acknowledging its large scope. We have revised the paper in response to the comprehensive suggestions of Reviewer 1 and 2, while also addressing your comments in turn below.

(1) Functional annotation (Figure 1 and Lines 101-107). What are the criteria for the definition of "active promoters" and "active enhancer"? The diagram in Fig. 1B was simulated and did not corelate with biological functions. For example, ATAC-seq peaks should overlap with those of active histone marks such as H3K27ac and H3K4me1, which should be anti-correlated with repressive marks such as H3K27me3. In Fig. 1G, it's hard to interpret variation of the enhancer (repeat) length and presence/absence. In addition. Each horizontal line should be labeled.

Reply: We feel all information queried in this comment was fully specified in the original manuscript. Perhaps the reviewer was not familiar with the approach we have taken, but it has been used extensively elsewhere and follows best practise in the field. We breakdown our reply sentence by sentence below.

- What are the criteria for the definition of "active promoters" and "active enhancer"?

Reply: Please refer to the Methods section, which clearly describes how promoter and enhancer states were defined, as outlined in the main Results section and Fig. 1.

"Chromatin state annotations

Chromatin states were annotated across the salmon and trout genomes using ChromHMM Input signal data were BAM alignments for the ATAC-seq and ChIP-seq (H3K27ac, H3K4me1, H3K4me3, and H3K27me3 marks) data generated as described above. Sampled matched input controls were given to estimate background signals for the ChIP-seq data. Prior to ChromHMM, BAM alignments were converted to BED format and filtered to keep reads only in chromosomes and exclude blacklist regions. The alignments were binarized using the ChromHMM BinarizeBed function with default bin size (200 bp), before running the ChromHMM LearnModel function. After testing models with various state numbers, with the aim to capture the most biologically relevant chromatin states 37 we proceeded with: two separate 12-state models for salmon DevMaps (i.e. embryo stages) and BodyMaps (i.e. tissue samples; brain, gonad, liver, muscle); and for trout, a 12-state model for DevMaps, and three separate 10-state models for each included tissue (brain, liver, muscle) (Extended Data Fig. 1). Note that each trout tissue was treated separately, as data quality was more variable between tissues compared to salmon. After generating the ChromHMM models, the non-blacklisted BAM files representing ATAC-seq and multiple histone mark ChIP-seq data were binarized using the ChromHMM BinarizeBam function with default bin size (200 bp). The combination of binarized signals from ATAC-seq and ChIP-seq marks were used together with the ChromHMM model for the respective species and condition to annotate regions across the genome representing different chromatin states at 200 bp resolution (see Results, main article).

Robust open chromatin definition and functional annotation

A set of robust open chromatin regions, defined as those that are highly reproducible across biological replicates per condition, was generated using the ATAC-seq narrow peak files resulting from the analyses described above, post-filtering to remove peaks either not found on chromosome sequences, or completely overlapping blacklist regions. The reproducibility of peaks between all possible pairs of biological replicates per condition was determined using the IDR approach [36] as done by the ENCODE project. IDR measures the reproducibility of the scoring of peaks between replicates to determine an optimal cut-off for peak significance. IDR was run using options --rank='p.value' and --soft-idr-threshold=0.1. This gave a set of significantly reproducible peaks between pairs of replicates. To deal with having three biological replicates per condition, the resulting reproducible peaks from each pair were merged together with bedtools merge (=>1bp overlap), taking the maximum IDR score for the resulting peak. With one set of reproducible peaks per condition, the summits of those peaks were re-calculated using the MACS2 refinepeak function and all replicate ATAC-seq BAM alignments were merged together.

Finally, the reproducible ATAC-seq peaks with summits were assigned with chromatin states per condition by intersecting ChromHMM annotations using bedtools intersect. When a peak overlapped more than one ChromHMM annotation in a given condition, the assigned chromatin state was chosen in order of importance, based on how confidently we could call the state given the combination of assay signals required. This order is the same order of the chromatin states shown in Fig. 1B (see main text) from top to bottom. A single unified set of robust open chromatin regions (reproducible ATAC-seq peaks) was generated per species by merging all overlapping peak regions (=>1bp overlap) across all conditions. Read counts from each ATAC-seq and ChIP-seq assays were counted with featureCounts using the BAM alignments for each biological replicate against the unified peak set of each species. This table of assay counts within robust open chromatin peaks for all samples was used for downstream analyses."

- The diagram in Fig. 1B was simulated and did not corelate with biological functions. For example, ATAC-seq peaks should overlap with those of active histone marks such as H3K27ac and H3K4me1, which should be anti-correlated with repressive marks such as H3K27me3

Reply: We agree Fig. 1b is a schematic, as stated in the legend. It was included for the benefit of readers that are not experts in chromatin and epigenomics. We are confused by the purpose of the follow up comment regarding ATAC-seq peaks and their overlap with histone marks. As described in the results and supported by extensive methods (shown above), our analysis explicitly captures these overlaps through a ChromHMM model, shown in Fig. 1c, where, for example, active enhancer regions are defined by the co-presence of open chromatin, H3K27ac and H3K4me1 signal and absence of H3K27me3. However, the reviewer should be aware that not all open chromatin regions overlap histone marks, as captured in our data and previous analyses (Baranasic et al. 2022. Nat. Genet. 54, 1037–1050)

- In Fig. 1G, it's hard to interpret variation of the enhancer (repeat) length and presence/absence. In addition. Each horizontal line should be labeled.

Reply: The phrasing here is impossible to penetrate, and we don't understand the issue being flagged. It's unclear what is meant by "enhancer (repeat) length" and "presence/absence". We have already provided labelling to explain the data shown on each horizontal line presented in Fig. 1g, which is explained in a comprehensive figure legend.

(2) Gene expression across tissues (Figure 2). If the purpose is for comparative analysis between species, it would be better to compare them within each of individual stages of development. The y-axis should be explained. The current maps are less informative.

Reply: Figure 2 does not show gene expression "across tissues", leaving us confused. It is unclear what the reviewer means by "*it would be better to compare them within each of individuals stages of development*". The Y-axis has been explained clearly in the legend. Please note, Figure 2 has been greatly improved in response to comments from another reviewer, where comparative analyses between species have been presented with greater clarity. Here, comparisons between individual stages of development are included both for orthologous gene expression and syntenic open chromatin regions.

(3) Ohnology expression divergence (Figure 3). The gene "regulatory" evolution should be changed to gene "expression" evolution in the text and figure. They used both ohnology and orthology in the analysis, which caused conceptual confusion. Please clarify them. In C and D, how were the two sets of ohnologs (red) computed to gain divergence values?

Reply: We breakdown our reply sentence by sentence as follows:

- gene "regulatory" evolution should be changed to gene "expression" evolution in the text and figure

Reply: We changed all cases of "regulatory evolution" or "regulatory divergence" to "expression evolution" or "expression divergence", including for the Fig. 3 legend, and in the relevant text describing Fig. 3, along with other places in the manuscript where the change is relevant.

- They used both ohnology and orthology in the analysis, which caused conceptual confusion. Please clarify them.

Reply: This comment is so short on details, we found it impossible to understand why the reviewer is conceptually confused. We carefully distinguished these analyses in the text and Fig. 3, including conceptually. We don't understand what change would help the reviewer here without them providing more explicit and detailed information.

- In C and D, how were the two sets of ohnologs (red) computed to gain divergence values?

Reply: Taking this comment alongside an earlier comment about how active regulatory elements were defined, we question if this reviewer looked at the Methods, where this information is explicitly explained. Moreover, the code is available in our GitHub repository, meaning other researchers can use our approach and reproduce the findings. For information, below is the relevant Methods section:

"Ohnolog expression divergence analysis

We calculated pairwise expression distances for defined classes of ohnolog and ortholog pairs (data shown in Fig. 3). We first calculated the mean of biological replicates of TPM values for each gene, separately for different samples. We then independently performed quantile normalization across DevMap and BodyMap samples using the preprocessCore R package [103]. Next, the standard error across samples was divided by the gene expression values. Ohnologs and orthologs were extracted from our homology prediction (Supplementary Table 6) for comparison. Specifically, we compared expression data four non-overlapping gene-pair categories: (i) 8,757 2:2 AORe (Early rediploidization) pairs, which started diverging in the salmon and trout ancestor; ii) 1,103 2:2 LORe (Late) rediploidization pairs, where two ohnolog pairs are conserved in both salmon and trout, but diverged independently in each species; (iv) 8,661 ortholog pairs representing AORe 2:2 ohnologs and (v) 5,099 ortholog pairs that represent singletons from AORe regions. Within each category, the orthogroup ID was used to segregate gene pairs into separate columns (named geneA and geneB). Expression divergence between the two members of a pair was measured using Jensen-Shannon distance (JSD). Prior to distance calculation, the two normalized TPM profiles were jointly quantile-normalized using the preprocessCore R package and re-scaled by dividing each value by the sum of all values. JSD values were then obtained using the JSD function of the phylentropy R package [104]. To

capture uncertainty, 100 bootstrap replicates were drawn for every embryonic stage (DevMaps) or tissue/maturity (BodyMaps) combination by resampling gene-pair indices with replacement. Within each replicate we recalculated JSD for the sampled set and subsequently recorded the mean and standard deviation across replicates. To assess the correlation of divergence patterns between categories, we calculated Pearson's correlation between the sample-wise mean JSD values; scatter plots, regression lines and 95% confidence intervals were produced with the ggpubr R package [105]."

(4) Regulatory elements (Figure 4). This part should come early in the Results to help understand and interpret functional annotation data.

Reply: We don't understand the logic behind this suggestion. An entire paragraph within the first section of the results is dedicated to introducing and defining the regulatory elements, along with associated quality metrics. The results are centrally presented in Fig. 1. Fig. 4 later offers a deeper dive into the conservation of regulatory element activity following the salmonid 4R WGD, which fits best in its current position within the manuscript, following the results on ohnolog expression evolution, which contextualises expectations for regulatory elements.

Hence, no changes are necessary in the manuscript.

(5) Enhancer evolution (Figure 5). Enhancer sequences tend to diverge more than promoter sequences. As such, it's better to focus on conserved ohnology/orthologs for this part of analysis. The presence of TEs in these sequences is interesting. In plants, TEs can serve as regulatory function via transcription and RNA-directed DNA methylation (RdDM). The role of TEs should be discussed since animals don't have RdDM pathway.

Reply: We breakdown our reply sentence by sentence.

- Enhancer evolution (Figure 5). Enhancer sequences tend to diverge more than promoter sequences. As such, it's better to focus on conserved ohnology/orthologs for this part of analysis.

Reply: This brief comment impossible for us to penetrate, meaning there are no meaningful changes we can make to improve the analysis. We agree enhancer sequences evolve more rapidly than promoter sequences, this is clearly explained in our manuscript - both in the context of our own results and citations to the work of others. Regarding the second part "*it's better to focus on conserved ohnology/orthologs for this part of analysis*", we don't understand what is being suggested and how it differs from what we have already done.

- The presence of TEs in these sequences is interesting. In plants, TEs can serve as regulatory function via transcription and RNA-directed DNA methylation (RdDM). The role of TEs should be discussed since animals don't have RdDM pathway.

Reply: We already dedicated extensive text to presenting and interpreting findings on the potential role of TEs in affecting regulatory functions in salmonids. We are unclear how referring to the RdDM pathway from plants will add anything relevant to existing text. This seems a tangential observation from the reviewer – if more explicit guidance and suggestions had been provided, it might have been possible to make meaningful changes, but the comment is so short on detail, we don't feel any improvements will arise by attempting discussing "the role of TEs" on the basis that "animals don't have RdDM pathway".

(6) Enhancer-CNEs (Figure 6). Enhancers are DNA sequences that are present or absent regardless of developmental stages and tissue specificity. It's unclear how enhancers could be present in one developmental stage but absent in other stages? One possibility is that tissue-specific transcription factor(s) could discriminate enhancer function but not the sequence itself. This confusion exists in the entire manuscript and should be clarified.

Reply: We don't believe there is any confusion on our part. The reviewer however seems confused, missing the point that we are profiling epigenomic activity of enhancer sequences (which we agree are fixed DNA sequences) across stages of ontogeny and different tissues. This was achieved by integrating ATAC-Seq and ChIP-seq (for four histone marks), to predict the epigenomic activity of regulatory elements according to stages and tissues (summarised in Fig. 1). In the analyses presented in Fig. 6 (as for Fig. 4 and Fig. 5), we focussed on active enhancers – so any variation present across stages/tissues relates to activity, not to the enhancer sequence itself. In Fig. 6, we further classified active enhancers in the salmon genome based on their overlap with CNEs of different phylogenetic ages, allowing us to explore how enhancer activity across sample types relates to cross-species conservation of enhancer sequences. All this information is clearly explained in the manuscript.

Minor: citation problems such as in lines 301, 306, 312 (52?), etc., probably from a previous format?

Reply: Thanks for spotting this typo, which was a standalone carry-over issue with the reference management software. The issue has been fixed in the revised submission, and the formatting carefully checked throughout.

In summary, the data are useful for the polyploid research community, but the description is broad across 1R-4R WGD events, while the most interesting findings were related to the 4R WGD. Perhaps the authors should focus on the conservation and divergence of gene expression and regulatory elements after 4R WGD and clarify the data interpretation and conclusions.

Reply: Thanks for noting the value of our research to the research community. We agree the most interesting findings relate to the 4R WGD, which arises from the fact that this was the event we studied in detail. We believe we have done exactly as you recommend, by focussing on "*the conservation and divergence of gene expression and regulatory elements after 4R WGD*". We in fact only refer to other vertebrate WGD events either when framing our study in the Introduction, or when concluding on the broader relevance and transferability of our findings in the Discussion. This broader framing is justified, because it expands the appeal and relevance to a larger audience, including researchers interested in how rediploidization has shaped genome evolution following WGD events beyond the salmonids.
